# Supplementary material for: Benefits of specialist palliative care by identifying active ingredients of service composition, structure, and delivery model: A systematic review with meta-analysis and meta-regression
Source: PLoS Med. 2024 Aug 2;21(8):e1004436. doi: 10.1371/journal.pmed.1004436 (PMC11329153; doi:10.1371/journal.pmed.1004436)
Supplement: S1 Appendix — (DOCX) [file pmed.1004436.s001.docx]

**Benefits of specialist palliative care by identifying active ingredients of service composition, structure, and delivery model: A systematic review with meta-analysis and meta-regression**

**S1 Appendix**

Miriam J. Johnson, Leah Rutherford, Anisha Sunny, Sophie Pask, Susanne de Wolf-Linder, Fliss E. M. Murtagh, Christina Ramsenthaler

[hycr22@hyms.ac.uk](mailto:hycr22@hyms.ac.uk)

# Review of publications presenting a classification system of models of specialist palliative care

To develop a classification system of service components and models, a literature search identified eight publications of frameworks or classification systems of effective specialist palliative care services.^1-8^ The following publications have been reviewed:

The proposed classification systems and frameworks were extracted from these publications into the following table. A content analysis across all seven publications identified common elements. To develop the final scoring system for classifying core components of specialist palliative care, we originally used the five categories proposed in Bainbridge *et al* (2016)^1^ – types of services offered, availability, characteristics of the care model, linkages to other resources, and process interventions as a higher-level schematic for categories. This initial system was revised two times in our expert group into a classification system classifying core components of palliative care into three higher categories of types of multi-disciplinary team (MDT) members involved, types of services provided, and availability. In category 1, types of MDT members involved, differential scoring was given to team members which are seen as essential team members in policy and guidance documents (Seow & Bainbridge, 2018)^7^. As the members of the MDT do not necessarily reflect the range of services they provide, as individuals may have additional training allowing them to provide extra services, each member of the MDT was scored according to their importance within SPC programmes. Psychologists and volunteers were given a score of 1 because their classification as core team members varies widely across countries and funding models of specialist palliative care (Bainbridge *et al*, 2016)^1^.

**Table A. Revised classification of core service components of specialist palliative care**

| **Category** | **Components** | **Scoring** |
| --- | --- | --- |
| **a) Type of multidisciplinary team members involved** | Physician | 3 |
|  | Nurse | 3 |
|  | Psychologist | 1 |
|  | Physiotherapist | 2 |
|  | Occupational health therapist | 2 |
|  | Social worker / social care | 2 |
|  | Spiritual support | 2 |
|  | Volunteers | 1 |
|  | ***Subtotal*** | ***16*** |
| **b) Type of service** | Symptom assessment (including psycho-social-spiritual) and management | 1 |
|  | Advance Care Planning | 1 |
|  | Carer support (prior to patient’s death) | 1 |
|  | Bereavement Care | 1 |
|  | Rehabilitation | 1 |
|  | Education / liaison of/with usual care team | 1 |
|  | ***Subtotal*** | ***6*** |
| **c) Availability** | Initial assessment | 1 |
|  | Planned follow-up | 1 |
|  | Patient or clinician-initiated (as needed) follow-up | 1 |
|  | Out of hours | 1 |
|  | ***Subtotal*** | ***4*** |
|  | **Total score** | **26** |

**Table B. Description of classification systems for core components of specialist palliative care services as presented in reviews to date**

| **Bainbridge *et al* (2016)^1^** | - Meta-review of specialist palliative care reviews - 30 components identified through content analysis of systematic reviews - Later classified in larger groups | **Group 1: Types of services offered**   - Bereavement support - Medical intervention - Pain and symptom management - Practical support - Patient and family education - Professional psycho-social support - Spiritual support | **Group 2: Availability**   - On call 24/7 - Physician home visits - 24/7 physician - 24/7 home visits | | | **Group 3: Characteristics of the care model**   - Case management (nurse navigation) - Expert consultation with other providers - Holistic care - Ongoing assessment - Patient directed care/joint decision making - Tailored care plan/needs-driven care - EOL expertise/ training - Multidisciplinary team | | **Group 4: Linkages to other resources**   - Access to dedicated inpatient beds - Contact established with primary care - Linkage between community services - Linkage with residential hospice - Visiting volunteer service | | | **Group 5: Process interventions**   - Advance care planning - Chart in the home - Emergency response plan - One contact - Team case rounds |
| --- | --- | --- | --- | --- | --- | --- | --- | --- | --- | --- | --- |
| **Bayly *et al* (2021)^2^** | - Meta-review of systematic reviews in geriatric or palliative care - 78 elements identified through content analysis using CATWOE health systems analysis model | **Group 1: Method of supporting integrated working**   - Collaborative working - Case management - Comprehensive assessment   **Group 2: Actors-workforce**   - Professional education - MDT care - Rehabilitation expertise training - EOL expertise training | **Group 3: Transformation – service model elements and components**   - Patient family education - Medication review - Self-management - Systematic risk screening - Contact with GP / attending doctor - Practical support - Medical intervention - Individualised MDT plan - Complex / medication management - Discharge planning - Professional psychosocial support - Team case rounds - Early rehabilitation assessment - Advance care planning - Emergency response plan - Spiritual support - Bereavement support | | | **Group 4: Transformation – mode of delivery**   - Ongoing assessment - Face-to-face and telephone - Face-to-face interaction - Access to inpatient beds - Physician home visits - 24-hour physician access - Telephone only - 24-hour home visits - Online only   **Group 5: Transformation – operational tools and guidance to support practice**   - Standard comprehensive assessment | | **Group 6: Worldview – methods of integrated working**   - Link to hospital - Expert consult with other providers - Link between community services - Joint provision – health and social care - Link to residential hospice | | | **Group 7: Worldview – conceptual model**   - Patient engagement - Active patient participation - Centrality of patient needs - Patient goal-driven care - Ongoing/continuous care - Joint decision making - Service-driven care planning - Needs and benefit-driven care planning - Caregiver engagement |
| **Brereton *et al* (2016)^3^** | - Meta-review of narrative or systematic reviews - Definition of model of palliative care: any structured care model involving multiple components including who delivers, where, to whom, when, how and for what purpose - Aim to explain which models were effective for which patient groups and which settings | **Models were clustered according to setting**  1. Home-based palliative care  2. Models delivered across multiple settings.  No further taxonomy was developed.  **Group 1: Palliative care or a palliative care approach including outpatient palliative care**  **Group 2: Specialised or specialist palliative care including models of hospice care**  **Group 3: Palliative care teams** | | | | | | | | | |
| **Evans *et al* (2019)^4^** | - Meta-review of systematic reviews in palliative care - Three typologies: - 1) Service delivery models - 2) Key components and subcategories - 3) Classification system for target outcomes | **Group 1: Service delivery models**   - Integrated geriatric care: person-centred care, services working together, accessed earlier at functional decline, focusing on quality of life with maintaining function - Integrated palliative care: person-centred care, services working together, accessed later trajectory of functional decline and dying, focusing on quality of life, emphasis on symptom distress and concerns - Specialist palliative care: complex problems - Generalist palliative care: basic knowledge of palliative care of staff treating patients with life-limiting conditions | | **Group 2: Key components and subcategories**   - Overarching methods to integrate and manage the continuum of care - Comprehensive assessment: person-centred, needs across physical psychological social and spiritual domains - Case management: coordinating care for patients, caregivers by assessing case as individual or team - Collaborative working: working across disciplines and organisations to plan and deliver services to meet the needs of individual and those close to them | | | **Group 3: Shared key components**   - Person-centered care: multi or single component (physical psychosocial spiritual) - Education (patients, caregivers, staff) - Workforce | | | **Classification for target outcomes**   - 1. Quality of life: quality of life, psychosocial symptoms, pain, symptom control, psychological/spiritual wellbeing, perception of health - 2. Function: functional status, ADL, cognitive function, falls, social support / participation, frailty - 3. Dignified end-of-life care: satisfaction with care, living will/DNR, quality of care, goals of care/preferences, quality of communication, quality of death - 4. Health service use: hospital admission / readmission, place of residence, death, hospital length of stay, appropriate treatments, ED attendance - 5. Survival: survival | |
| **Firth *et al* (2019)^5^** | - Empirical analysis of core specialist palliative care components via semi-structured interviews, Delphi study and structured interviews with UK service leads - 20 criteria, 16 reached consensus after Delphi rounds, 4 additional criteria added after final testing | **No grouping provided**   - Setting of care (inpatient hospital inpatient hospice, home based etc) - Type of care (advisory, hand-on) - Size – number of referrals annually - Number of disciplines delivering the care - Mode of care (face to face, telephone, other remote delivery) - Number of interventions available - Whether out-of-hours referrals accepted - Whether out of hours care available through service - Time when out of hours care is available - Out of hours mode (face to face or advisory) | | | | | - Extent of education/training provided to external professionals - Whether outcome and experience measures are used in the service - Whether standard bereavement follow-up care given - Complex grief follow-up provided - Primary diagnosis of patient   **4 additional criteria after final review:**   - Service publicly funded or voluntary funded - Patient or family self-referral - Standard discharge criteria   Purpose of care provided | | | | |
| **Luckett *et al* (2014)^6^** | - Review of policy documents, systematic reviews, RCTs and non-randomised studies - Variation in the content of models - Model classification system - Elements of palliative care models, ordered by country | **Model classification system:**   - Case management - Consultation model - Health or clinical networks - Integrated care - Liaison model - Managed clinical networks - Pop-up model - Shared-care model: 1) Approach to care using skills and knowledge of a range of health care professionals with joint responsibility of a patient’s care; 2) Narrow approach of joint participation of general practitioners and specialist in the planned delivery of care; 3) Systematic cooperation across systems for mental health care | | | **Elements of effective models:**   - Case management: holistic needs, coordination of services across health and social care, patient-centred care - Shared care: identifiable lead clinician working together with other disciplines, communication and coordination, rapid needs-based response and navigational strategies - Specialist outreach services: hard-to-reach or underserved communities, collaboration with primary care, education, other health services - Managed health networks: formal linking of groups and disciplines, across funding boundaries - Integrated care: coordination of disparate services, needs-based and patient-centred, patients and family involved in decision making and goal setting - Volunteers: use of volunteers where informal caregivers are lacking | | | | **Policy document analysis**  Varying list of goals of how services should be configured | | |
| **Seow & Bainbridge (2018)^7^ and Seow *et al* (2020)^8^** | - Rapid review (narrative) of five publications focusing on palliative home care (systematic reviews to surveys and qualitative studies) to identify common elements across studies - Qualitative study with 78 providers from 11 community-based specialist palliative care services | **Essential elements of palliative home care:**   - Teamwork within and across settings: transitions, communication, primary care involvement - Management of pain and physical symptoms - Holistic management of nonphysical and physical symptoms, interprofessional handling - Having the right people on the team - Timely and responsive: access in times of crisis, proactive symptom management, early identification, 24/7 as ideal - Patient and family preparedness: caregiver empowerment through education, knowledge and expectation handling | | | **Main models of care**   - Substitution model - Mixed model - Shared-care model - Consultation model | | | | **Seven common care practice themes from qualitative study:**   - Specialist expertise 24/7 - Intrateam communication - Timeliness - Physical symptom and psychosocial-spiritual management - Education and preparedness - Peace and fulfilment - Advocates for patient preferences | | |

*Abbreviations*: ADL = activities of daily living, CATWOE = Customers, actors, transformation process, worldview, owners, environmental constraints, DNR = do-not-resuscitate order, ED = emergency department, EOL = end of life care, GP = general practitioner, MDT = multi-disciplinary team, RCT = randomized controlled trial, UK = United Kingdom

**References to S1 Appendix**

1. Bainbridge D, Seow H, Sussman J. Common components of efficacious in-home end-of-life care programs: a review of systematic review. J Am Geriatr Soc. 2016;64:632–639.

2. Bayly J, Bone AE, Ellis-Smith C, et al. Common elements of service delivery models that optimize quality of life and health service use among older people with advanced progressive conditions: a tertiary systematic review. BMJ Open. 2021;11:e048417.

3. Brereton L, Clark J, Ingleton C, et al. What do we know about different models of providing palliative care? Findings from a systematic review of reviews. Palliat Med. 2017;31:781–797.

4. Evans CJ, Ison L, Ellis‐Smith C, et al. Service delivery models to maximize quality of life for older people at the end of life: a rapid review. Milbank Q. 2019;97:113–175.

5. Firth AM, O’Brien SM, Guo P, et al. Establishing key criteria to define and compare models of specialist palliative care: A mixed-methods study using qualitative interviews and Delphi survey. Palliat Med. 2019;33:1114–1124.

6. Luckett T, Phillips J, Agar M, Virdun C, Green A, Davidson PM. Elements of effective palliative care models: a rapid review. BMC Health Serv Res. 2014;14:136.

7. Seow H, Bainbridge D. A Review of the Essential Components of Quality Palliative Care in the Home. J Palliat Med. 2018;21:S37–S44.

8. Seow H, Bainbridge D, Brouwers M, et al. Common care practices among effective community-based specialist palliative care teams: a qualitative study. BMJ Supp Palliat Care. 2020;10:e3.
