# Supplementary material for: Benefits of specialist palliative care by identifying active ingredients of service composition, structure, and delivery model: A systematic review with meta-analysis and meta-regression
Source: PLoS Med. 2024 Aug 2;21(8):e1004436. doi: 10.1371/journal.pmed.1004436 (PMC11329153; doi:10.1371/journal.pmed.1004436)
Supplement: S2 Appendix — (DOCX) [file pmed.1004436.s002.docx]

**Benefits of specialist palliative care by identifying active ingredients of service composition, structure, and delivery model: A systematic review with meta-analysis and meta-regression**

**S2 Appendix**

Miriam J. Johnson, Leah Rutherford, Anisha Sunny, Sophie Pask, Susanne de Wolf-Linder, Fliss E. M. Murtagh, Christina Ramsenthaler

[hycr22@hyms.ac.uk](mailto:hycr22@hyms.ac.uk)

# Overview of all relevant systematic reviews and meta-analyses of effectiveness of specialist palliative care on quality of life and emotional wellbeing

We pursued a systematic review of systematic reviews and meta-analyses published in Pubmed using the search terms “palliative care” AND (“systematic review” OR “meta-analysis”) AND “model” and searching from inception to 02/03/2023.

We only included systematic reviews and/or meta-analysis that had been published determining the effectiveness of specialist palliative care using multidisciplinary, multi-component interventions in patients with advanced cancer or non-cancer conditions. Reviews of mixed cancer or noncancer populations were included as long as they described the sample as having advanced or life-limiting/life-threatening disease. Reviews of specialist palliative care in single conditions were included. Review were included if they provided a narrative synthesis, estimate of effect size or meta-analysis for patient-reported or patient-centred health-related quality of life (QoL), symptom burden (single symptom or multi-symptom scales), or an emotional wellbeing outcome such as psychological distress, depression or anxiety. Reviews were included if they synthesised quantitative evidence (experimental or quasi-experimental studies, observational studies). There was no minimum number of included studies per review and empty reviews were included as well.

Excluded were all reviews of interventions not described or characterised as specialist palliative care or being single component (e.g., advance care planning, telehealth only, generalist healthcare providers, generalist palliative care). Further excluded were all reviews in pediatric populations or in populations not comprising individuals with advanced disease with the explicit exception of reviews of early palliative care. Reviews were excluded if they summarized qualitative studies only.

Results of systematic reviews presenting a narrative synthesis of findings only are presented in S2 Table A. Twelve meta-analyses were found (see S2 Table B). There was large variation regarding setting, service models, and patient populations studied. Meta-analyses were often based on one quarter of eligible trials only because of heterogeneous outcome measures. Two meta-analyses attempted to address this problem by converting QoL results into Functional Assessment of Therapy-Palliative Care units (FACIT-PAL) or other common QoL measures.

(a) Effect sizes: Meta-analyses of QoL outcome found the point estimate of the standardized mean differences (SMD) ranged from 0.04 to 0.60 (moderate effect size) with the median SMD across all reviews for QoL being at 0.24 (small effect size, see eTable5). The pooled SMD was often not statistically significant due to low power. The maximum SMD was consistently observed at the 3 months follow-up time point. Eleven reviews reported effects for symptom burden with a median reduction in symptom burden of SMD 0.20. Only three reviews reported an emotional outcome measure (depression or anxiety) finding negligible (SMD 0.09) or small (0.33) effect sizes, respectively.

(b) Service composition: Eight reviews (see S2 Table B) investigated service composition as an effect modifier for SPC effectiveness. Narrative syntheses resulted in seven classification frameworks to logic models for the SPC intervention. Subgroup analyses based on at most five RCTs suggested that a more comprehensive multidisciplinary team composition as well as higher level of integration or collaboration between settings and providers yielded higher, but imprecise effect sizes or more statistically significant benefits. A consistent pattern of a possible “dose” relationship of service composition was not seen.

Below, the identified systematic reviews providing a narrative synthesis only are summarised first, followed by those reviews that reported effect sizes or derived a summary effect via meta-analysis. Finally, in S2 Table C we report all effect sizes from meta-analyses per outcome to give a better overview of results to date.

**Table A. Overview of systematic reviews using narrative synthesis of findings**

| **Autor, Year** | **Type of synthesis** | **Objective** | **Time frame** | **PICO/eligibility** | **# included studies** | **Results** |
| --- | --- | --- | --- | --- | --- | --- |
| Bakitas *et al* 2015^1^ | Narrative synthesis | To synthesize empirical evidence on persons with cancer to describe the current practice and state of research relating to PC in rural settings | January 1990 to February 2014 | Research study or systematic reviews  Rural focus  PC intervention | 39 studies included  4 studies reporting 2 RCTs evaluating an intervention of relevance | Both RCTs offered early PC approaches to patients with advanced cancer or their caregivers, settings were rural New Hampshire and Vermont  **Quality of life**: improved quality of life over time, statistically significant  **Symptom burden**: statistically nonsignificant improved patient symptoms  **Emotional wellbeing**: improved depression over time, statistically significant |
| Bradley *et al* 2018^2^ | Narrative synthesis | To summarise existing quantitative evidence on PC interventions that facilitate social support | Inception to January 2017 | Social support interventions: PC interventions included that offered opportunities for social support if they facilitated face-to-face interactions with other people outside the person’s home  Adult outpatients, diagnosis of life-limiting illness | 16 RCTs, quasi-experimental studies with control group, prospective studies without control group  12 advanced cancer  4 mixed diagnoses | Results for palliative day care: statistically significant effect on symptoms in two studies, on hope in one study.  Difficult to synthesise results due to attrition and acquiring a baseline prior to day care  No results regarding **quality of life** outcome reported. |
| Cunningham *et al* 2016^3^ | Narrative synthesis | Published evidence of higher quality comparative studies of PC interventions delivered outside the hospital in an outpatient setting | Not stated | Published evidence of higher quality comparative studies of PC interventions delivered outside the hospital in an outpatient setting and directed at both physical and psychosocial patient care for advanced disease | 14 good/fair quality studies  Specialist interventions described in 10 studies  Generalist interventions in 4 studies | Most interventions described as “generalist” appeared to be multi-disciplinary, followed core principles of PC, and were delivered by experienced personnel, making the distinction between specialist and generalist PC problematic.  **Quality of life**: 7 studies overall, 5 studies specialist PC, 2 studies generalist PC  Cancer and mixed populations – PC either improved patient quality of life or had comparable improvements in quality of life relative to usual care  **Symptom burden**: 7 studies overall, 5 studies specialist PC, 2 studies generalist PC, the same 7 RCTs as for quality of life; 5/7 studies found no statistically significant difference in any symptoms between groups; 3/7 studies showed reductions in symptom burden for some symptoms  **Emotional wellbeing**: 6 studies, 4 studies in specialist PC, 2 studies generalist PC, anxiety and depression, 4/6 studies found statistically significant results in favor of the intervention |
| Datla *et al* 2019^4^ | Narrative synthesis | To identify the evidence in relation to PC for people with symptomatic heart failure | 1995 to December 2017 | Experimental or quasi-experimental observational and service evaluations, national audits  Symptomatic heart failure (New York Heart Association Classes III and IV)  Specialist or generalist PC | 23 studies  8 manuscripts/7 studies RCTs  4 feasibility or pilot RCTs/studies  1 quasi-experimental study  7 cohort studies  2 case-control studies  1 cross-sectional study | 16 studies community-based, 2 extended across community and hospitals, 3 hospital alone, 2 hospice  Range of sample size from 13 to 16,613 patients  **Quality of life**: 7/11 studies statistically significant improvement in quality of life, 1 study noted improvement in only HrQoL but no disease-specific quality of life  **Symptom burden**: 5/9 studies statistically significant improvement in breathlessness, pain, and fatigue, 2 studies found more symptom improvement in the control arm  **Emotional wellbeing**: 5/9 studies statistically significant improvement in depression in the intervention arm |
| Davies & Higginson, 2005^5^ | Narrative synthesis | To systematically review how the structure and process of specialist palliative day-care relate to outcomes for adults with cancer | Inception to December 2003 | Studies reporting information on palliative day-care service structure, care processes or outcomes  Qualitative or quantitative studies  Adults with cancer | 15 manuscripts with 12 observational studies included | **Quality of life**: 1 prospective comparative study found no difference in quality of life scores over time.  **Symptom control**: Two observational studies; 1 mortality follow-back survey in which 71% of 65 bereaved caregivers reported the patient had obtained pain relief from symptoms; one comparative study showed lower symptom control (p = 0.03). Both studies were limited by baseline differences between the groups. |
| Davis *et al* 2015^6^ | Narrative synthesis | To review and discuss randomized control trials examining the integration of PC earlier in the disease trajectory for patients with serious illnesses as outpatients or living at home | Not stated | RCTs examining the integration of PC earlier in the disease trajectory for patients with serious illnesses as outpatients or living at home | 28 studies  15 RCTs of outpatient PC  13 RCTs of palliative home care | Early PC interventions  Improvement in certain symptoms (depression), quality of life mentioned; in some included RCT quality of life was not improved. No further synthesis or vote-counting. |
| Diop *et al* 2017^7^ | Narrative synthesis | To systematically characterize interventions and effectiveness of PC for advanced heart failure patients | Inception to June 2016 | Primary diagnosis of heart failure who underwent PC or hospice interventions  Mixed populations allowed as long as heart failure was quantified  All non-qualitative study designs | 15 studies included  8 RCTs, 2 prospective non-randomized trials, 5 retrospective controlled studies  Maximum of 16,613 patients included | **Quality of life**: patient quality of life improved significantly in 5/6 studies  **Symptom burden**: improvement for dyspnea and sleep quality reported in 5/6 studies  **Emotional wellbeing:** statistically significant improvement seen for depression in 3/6 studies, anxiety in 3/6 studies |
| Ferrell *et al* 2017^8^ | Narrative synthesis | To provide evidence-based recommendations to oncology clinicians, patients, family and friend caregivers, and PC specialists on PC in advanced cancer | March 2010 to January 2016 | Phase III RCTs or secondary analyses of RCTs  Patients with cancer | 9 RCTs, two publications reporting on 1 large quasi-experimental trial, five secondary publications based on prior published RCTs | No synthesis of findings provided, only discussion of individual studies |
| Gilbertson-White *et al* 2017^9^ | Narrative synthesis | To examine the effect of PC and supportive oncology interventions on quality of life in people with advanced cancer living in rural areas | 1991 to 2016 | Rural residents as all or part of the sample  PC or supportive care intervention  Patients with advanced cancer  All research study types, including mixed-method studies and program evaluations | 9 studies included, describing 8 projects  1 RCT  4 feasibility/pilot studies  3 program evaluations | **Quality of life:** 2 studies – 1 pilot study and 1 RCT; both studies showed statistically significant improvement in quality of life  **Symptom burden**: 3 articles (2 pilot studies, 1 RCT), decreased physical symptom burden but not statistically significant, also only trends observed in the non-randomized trials  **Emotional wellbeing:** 5 articles – 4 pilot/feasibility studies, 1 RCT, decrease in anxiety and depression across all 5 studies, but results were not statistically significant; 1 study reported a statistically significant decrease in anxiety in the intervention group. |
| Gomes *et al* 2013^10^ | Narrative synthesis | To quantify the effect of home PC services for adult patients with advanced illness regarding the odds of dying at home and outcomes such as symptom control, quality of life, caregiver distress, satisfaction with care, and costs | Inception to November 2012 | RCTs, controlled clinical trials, controlled before-after studies, interrupted time series  Impact of home PC services  Adults with advanced illness or their family caregivers, or both | 23 studies  16 randomized controlled rials  37,561 participants and 4042 family caregivers | Mostly advanced cancer included, also chronic heart failure, chronic obstructive pulmonary disease, HIV/AIDS, multiple sclerosis  23 studies, 16 RCTs (6 with high quality)  Meta-analysis only for odds of dying at home  **Symptom burden**: 3 trials and 1 before-after study with 2107 participants overall showed a statistically significant effect of reducing symptom burden |
| Hall *et al* 2011^11^ | Narrative synthesis | To determine the effectiveness of multi-component PC service delivery interventions for residents of care homes for older people, and to describe the range and quality of outcome measures | Inception to February 2010 | RCTs, controlled clinical trials, controlled before-and-after studies, interrupted time series of multi-component PC service delivery interventions  Residents of care homes for older people  Only multi-component interventions | Two RCTs, 1 before-after study  3 studies included | No studies included that assessed **quality of life** outcomes, status of specialist PC debatable  Included studies only reported on outcomes satisfaction with care, discomfort or process measures  **Symptom burden**: 1 study reported lower observed discomfort in residents with end-stage dementia |
| Health Quality Ontario, 2014^12^ | Narrative synthesis | To determine whether an optimal team-based model of care exists for service delivery at the end of life | January 2000 to October 2013 | Studies evaluating a team model of care compared with usual care in an end-of-life adult populations; a team had to represent at least 2 disciplines | 10 RCTs | 6 team-based models of care were evaluated: hospital with direct contact, home with direct contact, home with indirect contact, comprehensive with indirect contact, comprehensive with direct contact, and comprehensive with direct and early contact.  Moderate-quality evidence that the use of a comprehensive, direct, and early (up to 24 months before death) contact model improved patient quality of life, and symptom management.  **Quality of life**: 6 studies, a statistically significant improvement in patient QoL was seen in 3/6 studies  **Symptom burden**: 4 studies, 2/4 studies showed statistically significant improvements in symptom burden, but in one study a higher distress score was observed in one patient group in the mixed patient group study. |
| Higginson & Evans 2010^13^ | Narrative synthesis | To determine whether specialist PC teams achieve their aims and improve outcomes for patients with advanced cancer and their caregivers, in terms of improving symptoms and quality of life and/or emotional concerns | 2000 to end of 2009 | Comparison of specialist PC team with usual care with 2 or more health workers and at least 1 having specialist training  Community, hospital (inpatient or outpatient), or hospice setting  Advanced cancer or their caregivers, also mixed populations included | 8 RCTs and 32 observational or quasi-experimental studies | Overall, the evidence demonstrated that home, hospital and inpatient specialist PC  significantly improved patient outcomes in the domains of pain and symptom control, anxiety, and reduced hospital admissions.  No meta-analysis, just based on vote counting, no overall synthesis of findings per outcome given.  **Quality of life:** Quality of life, when measured, was less often different between groups and tended to deteriorate over time. |
| Holmenlund *et al* 2017^14^ | Narrative synthesis | To review the existing literature about specialist PC and its effects on quality of life, symptoms and survival in advanced cancer | Inception to October 2016 | Adult patients with advanced, metastatic, incurable or life-limiting cancer diagnosis  RCTs  Specialist PC interventions | 8 RCTs  6 parallel trials, 2 cluster-randomized trials | **Quality of life:** 5/8 studies reported a statistically significant greater positive effect on quality of life in the intervention group  **Symptom burden**: 6/8 studies reported on symptom burden, 2/6 studies found a decrease in symptom burden in the intervention group, 4/6 found a statistically significant reduction in the intervention group compared to the control group over time  **Emotional wellbeing:** 7/8 studies reported on emotional wellbeing, 5/7 study observed a statistically significant improvement in the intervention group compared to the control group |
| Johnston *et al* 2020^15^ | Narrative synthesis | To review the evidence on the effect of out-of-hours specialist or generalist PC for adults on patient and caregiver outcomes, and costs and cost-effectiveness | January 2000 to 12^th^ November 2019 | Studies comparatively evaluating the effect of out-of-hours specialist or generalist PC  Adult patients  No restrictions on study design | 1 study identified through full-text search but not included | No study included due to quality assessment  Empty review, no synthesis |
| Leclerc *et al* 2014^16^ | Narrative synthesis | To determine the effectiveness and the harm and adverse effects of the interdisciplinary team approach to providing end-of-life PC to adult patients and their caregivers | 1995 to February 2013 | Comparative studies of any qualitative or quantitative designs  Community-dwelling or adult inpatients with a terminal condition  Received PC from a team with members of at least 3 professions  Team described as inter-disciplinary  Comparison group | 4 quantitative studies included  1,321 participants | **Symptom burden:** 2/4 studies reported symptom burden, 1 study found no difference for symptom severity with a p = 0.91, 1 study reported statistically significant improvement in physical comfort based on an after-death interview  **Emotional wellbeing:** 2/4 studies reported emotional wellbeing, 1 study found no statistically significant difference in emotional burden with p = 0.07, 1 study reported statistically significant improvement in anxiety or depression based on an after-death interview |
| Lorenz *et al* 2008^17^ | Narrative synthesis | To assess evidence about interventions to improve palliative and end-of-life care | January 1990 to November 2005 | All study types  Adult populations  Included all high-quality evidence for specialist end-of-life or PC and interventions | Varying numbers of included studies and systematic reviews for guideline development | No outcomes-based presentation of findings  Strong evidence to support multi-component interventions to improve continuity in heart failure |
| Phongtankuel *et al* 2018^18^ | Narrative synthesis | To (1) describe the delivery of multicomponent PC interventions, (2) characterize the disciplines, (3) identify the components being implemented, and (4) analyze whether the number of disciplines or components is associated with positive outcomes | January 1980 to December 1, 2015 | Multicomponent PC interventions in adult cancer and noncancer conditions  Care model includes at least 2 domains  Not only studies focusing on the end of life  All study designs | 71 articles detailing 64 multicomponent PC interventions | No synthesis regarding outcomes, solely noted the proportion of studies reporting a statistically significant result in association with different disciplines involved or components.  For **quality of life**, there was a higher proportion of statistically significant results with more disciplines involved, particularly with involvement of a physician or physical/occupational therapy |
| Salisbury *et al* 1999^19^ | Narrative synthesis | To systematically review the research evidence about the impact of alternative models of specialist PC on the quality of life of patients | Inception to June 1998 | Research studies of different designs  Quality of life measured by a validated scale  Specialist PC not defined | 27 comparative studies | No synthesis of findings, results for individual studies based on p values presented in a table |
| Sampson *et al* 2005^20^ | Narrative synthesis | To perform a systematic review regarding the efficacy of a PC model in patients with dementia | Inception to October, 1 2003 | Dementia patients that received PC interventions according to the WHO definition  No systematic reviews | 4 studies included  1 RCT  1 prospective cohort study  2 descriptive studies of programs | **Symptom burden:** 1/4 studies reported lower observed physical discomfort (prospective cohort study, statistically significant) |
| Shepperd *et al* 2021^21^ | Narrative synthesis | To determine if providing home-based end-of-life care reduces the likelihood of dying in hospital and what effect this has on patients' symptoms, quality of life, health service costs and caregivers compared with inpatient hospital or hospice care. | Inception to March 2020 | Update of Cochrane review  Hospital at home: home-based end-of-life care  Comparator: inpatient hospital or hospice care versus home-based PC  exclude since no comparator usual care | 4 RCTs | The effect on patient outcomes and control of symptoms was uncertain.  **Symptom burden:** 1 RCT/4 studies assessed pain control of patient via proxy-report from caregivers (n = 168). There may be a small difference in participants pain control assessed by caregivers (4-point scale: MD –0.48 points, 95% CI –0.93 to –0.03).  **Emotional wellbeing:** There was no difference on psychological wellbeing outcomes in 1 RCT. |
| Singer *et al* 2016^22^ | Narrative synthesis | To identify (1) individuals appropriate for PC and (2) elements of health service interventions (personnel involved, use of multidisciplinary teams, and settings of care) effective in achieving better outcomes for patients, caregivers, and the healthcare system | January 2001 to August 2015 | Adults with advanced illness  Health service interventions addressing the patient or the caregiver  Cancer and non-cancer conditions  RCTs | 124 RCTs | **Quality of life:** 42/96 studies reported statistically significant results in the majority of quality of life domains.  **Symptom burden:** Approximately 20%–30% of studies that addressed pain (9 of 37) or dyspnea (3 of 14) had a majority of significant results in each of those domains  **Emotional wellbeing:** 40-50% of studies assessing depressive symptoms or anxiety reported statistically significant findings. |
| Stevens *et al* 2010^23^ | Narrative synthesis | To determine whether the provision of palliative day care services has a measurable effect on attendees’ wellbeing | 1980 to 2009 | Primary research and audit/service improvement studies  Only generalist or specialist palliative day care services | 35 individual studies | 17/35 studies explored the impact on wellbeing qualitative, all 17 studies reported that attendance of palliative day care had a positive outcome on attendees’ wellbeing |
| Tassinari *et al* 2016^24^ | Narrative synthesis | To assess the role of early PC in patients with advanced oncologic and non-oncologic chronic diseases | Not stated | Randomized phase III trials of early, simultaneous PC compared to standard care  Cancer and non-cancer advanced diseases | 12 articles describing 9 RCTs  2 nonrandomized trials included | **Quality of life**: 2/7 RCTs report a statistically significant improvement in the early PC group compared to usual care  **Symptom control:** 1/5 RCTs report a statistically significant improvement in symptom control. |
| Thomas *et al* 2006^25^ | Narrative synthesis | To identify and analyze all published RCTs that focus on the organization of end of life care provided to persons who are terminally ill, near death, or dying | Not stated | A literature review of RCTs of the organization of care at the end of life | 23 RCTs | Vote counting  **Quality of life and symptom burden:** Six studies found some improvement in ratings of the quality of life and perceived management of symptoms of patients through the provision of care by PC teams. 3 studies found no statistically significant improvement in symptoms |
| Yang *et al* 2016^26^ | Narrative synthesis | To evaluate the effectiveness of PC for cancer patients in the acute inpatient hospital setting | January 2005 to May 2015 | Hospital-based PC teams on adult cancer patient outcomes  All study designs included  Mixed cancer populations included | 21 studies with pre-post designs, process evaluations, economic evaluations | **Symptom burden:** 2 pre-post studies with control groups showed a statistically significant improvement in symptom control  12 pre-post studies without control groups demonstrated that PC consultation improved symptom control. |
| Zimmermann *et al* 2008^27^ | Narrative synthesis | To systematically review the evidence for effectiveness of specialized PC | Inception to January 2008 | RCTs evaluating SPC interventions  Definition SPC: service of professionals that provides or coordinates comprehensive care for patients with terminal illness  Excluded: only 1 component of SPC or 1 aspect of quality of life as outcome | 22 RCTs | **Quality of life:** 13 RCTs, 9 no statistically significant differences but lacked power, 2 home PC programs significant differences on SF-36 in 6 of 9 domains, 2 of 8 domains;  1 study nurse-led follow up EORTC QLQ-C30 emotional functioning improvement, dyspnea with lung module  1 study with structured multidisciplinary intervention for radiation therapy outpatients, at 4 weeks significant difference in QOL but not at 8 or 27 weeks  **Symptom burden**: 14 RCTs, 1 trial statistically significant difference in individual symptoms, 3 studies in outpatients (cancer, COPD, CHF) assessed dyspnea; statistically significant improvement only in symptom distress (not severity) |

*Abbreviations*: CHF = chronic heart failure, CI = confidence interval, COPD = chronic obstructive pulmonary disease, EORTC QLQ-C30 = European Organization for the Research and Treatment of Cancer Quality of Life Questionnaires-C30, MD = mean difference, HrQoL = health-related quality of life, PC = palliative care, PICO = population, intervention, control, outcome, QOL = quality of life, RCT = randomized controlle trial, SF-36 = Shortform 36 quality of life questionnaire, SPC = specialist palliative care

**Table B. Overview of systematic reviews using meta-analyses**

| **Autor, Year** | **Type of synthesis** | **Objective** | **Time frame** | **PICO/eligibility** | **# included studies** | **Results** |
| --- | --- | --- | --- | --- | --- | --- |
| Bajwah *et al* 2020^28^  Oluyase *et al* 2021^29^ | Meta-analysis | To assess the effectiveness and cost-effectiveness of hospital specialist PC compared to usual care for adults with advanced illness and their caregivers | Inception to August 2019 | RCTs  hospital specialist PC  usual care  PC patients and their unpaid caregivers | 42 RCTs  7779 participants  6678 patients  1101 caregivers  21 in cancer  14 in non-cancer  7 mixed diagnoses | 40 studies high risk of bias in at least one domain  Patient HrQoL showed a small effect size based on 10 studies (SMD 0.24 over usual care, 95% CI 0.15 to 0.37; I^2^ = 3%, 1344 participants, low-quality evidence).  Hospital-based specialist PC improved other person-centered outcomes: **patient symptom burden**: small effect SMD = -0.26 (95% CI -0.41 to -0.12; I^2^ = 0%, 6 studies, 761 participants, very low-quality evidence) |
| Chan *et al* 2023^30^ | Meta-analysis | To determine the association of PC for progressive neurologic diseases with patient and caregiver-centered outcomes | Inception to September 2021 | RCTs, quasi-experimental studies, pilot studies of adult populations with a diagnosis of Parkinson disease, multiple sclerosis, motor neuron disease, dementia, multiple system atrophy, progressive supranuclear palsy, PC interventions with 2 or more domains of the 2018 National Consensus Project for Quality PC | 15 studies in narrative synthesis  12 RCTs included in meta-analysis  3431 patients  1776 caregivers | **Quality of life:** 7 studies (n = 1812 participants), 3 reported statistically significant improvements in the PC groups compared with usual care  6 studies pooled in meta-analysis, PC was not statistically significantly associated with better QoL (SMD 0.19; 95% CI -0.07 to 0.44, I² = 76%, p = 0.150). No significant group differences in subgroup meta-analysis.  **Symptom burden:** 11 trials (n = 1984), 4 reported statistically significant improvements in the PC group compared to the usual care group  Meta-analysis with 9 trials (n = 1117) and statistically significant association of PC with reduced symptom burden (SMD -0.34, 95% CI -0.59 to -0.09, I² = 72%, p = 0.008)  Subgroup analysis showed statistically significant differences in trials with and without an interdisciplinary team, home visits, PC physicians, spiritual care, 12-months duration of trial, mixed neurodegenerative diseases |
| Chyr *et al* 2022^31^ | Meta-analysis | To perform a mixed methods review to evaluate the effectiveness and implementation of models for integrating PC into ambulatory care for US adults with noncancer serious chronic illness | January 2000 to May 2020 | Both RCTs and non RCTs, qualitative, mixed methods, process evaluations  Adult patients with serious life-threatening chronic illness  SPC in the ambulatory setting, integration  US-based studies | 22 articles included  17 quantitative, 5 qualitative  14 quantitative studies: 9 RCTs, 5 controlled trials or prospective studies  All non-cancer conditions, mainly heart failure | 12 integrating PC, 4 shared care, 4 care coordinators or social workers in care delivery, 4 consulting care model  Two models of integrating PC identified: shared care model, consultative care model  Meta-analysis of 4 RCTs: **Quality of life** outcome – no difference compared to usual care, SMD 0.19, range -0.03 to 0.41 for 2 RCTs, other 2 RCTs did not report follow-up data, model of integrating PC did not improve quality of life  **Overall symptom burden**: 2 RCTs, 1 study reported an improvement of MD 0.1 (95% CI: -0.5 to 0.8, not significant, 1 study reported an improvement on the Edmonton Symptom Assessment Scale-Parkinson’s Disease with MD -8.26 (95% CI: -13.9 to -2.6, p < 0.05), but likely not clinically meaningful and inconsistent results.  **Depressive symptom score**: 6 RCTs, 1 CT, 2 prospective cohort, meta-analysis of 3 RCTs: SMD -0.09 (95% CI: -0.35 to 0.16, p > 0.05), failure to include more studies due to missing variability data, statistically significant results in studies not included in meta-analysis, but probably not clinically meaningful |
| Cui *et al* 2019^32^ | Meta-analysis | To provide a comprehensive assessment of the efficacy of collaborative care intervention in patients with chronic heart failure | Inception to September 2018 | Collaborative care interventions  Patients with chronic heart failure  RCTs | 21 RCTs  2999 patients | Significant improvement in collaborative care interventions:  **Quality of life:** SMD = 0.60 (95% CI: 0.27-0.94, I² = 94%, 21 studies with 2999 patients)  Subgroup analyses for exploring heterogeneity:   - multidisciplinary interventions (SMD 0.63, 95% CI 0.11-1.11), lower in non-multidisciplinary interventions (SMD 0.59, 95% CI 0.11-1.06) - Patients administered face-to-face interventions SMD 0.54 (95% CI 0.24-0.85) compared to telephone-only interventions   **Anxiety level**: 4 RCTs, significantly improved anxiety levels in collaborative care group with SMD 0.33 (95% CI 0.12-0.55, 203 intervention patients, 205 control patients)  Outcome measures not specified or described, likely incorrect pooling of different measures |
| Finlay *et al* 2002^33^, Higginson *et al* 2002^34^ | Narrative synthesis | To determine whether hospital-based PC teams improve the process or outcomes of care for patients and families at the end of life | Inception to December 1998 | Evaluations of PC teams working in hospitals with 2 or more healthcare workers of which at least 1 had specialist training in PC  Life-threatening illness or their unpaid caregivers  Broad range of outcomes and designs (experimental and quasi-experimental studies) | 43 studies, all quantitative and qualitative study designs  9 studies in hospital-based PC teams  Two of these result sets analyzed separately | 43 studies evaluating the effectiveness of hospital-based PC in general:  **Pain**:20 studies, mean SMD: 0.41, minimum -0.14, maximum 2.04  **Other symptoms**: 14 studies, mean SMD: 0.32, minimum -0.11, maximum 1.76  **Quality of life**: 17 studies, mean SMD: 0.18, minimum -0.62, maximum 0.20  9 studies evaluating hospital-based PC teams:  nature of interventions varied, teams sometimes individual nurses with unclear training, other individual doctors, some were multi-professional, insufficient data on the intensity of the interventions, time periods of care (few days to weeks), out of hours/night work unclear  No pooled effect sizes reported, only individual standardized mean differences:  **Quality of life**: individual SMDs are 0.04, 0.36, 0.53, 0.50, 0.34 reported in 5 studies including solely hospital-based PC or broader interventions  **Pain**: individual per-study SMDs are 2.04, 0.67, 0.03, 0.08, 0.32, 0.34, 0.48, 0.60 (8 studies)  **Other symptoms**: individual per-study SMDs are 1.76, 0.27, 0.09, -0.05, 0.66 (5 studies) |
| Fulton *et al* 2019^35^ | Meta-analysis | To evaluate the effects of integrated outpatient palliative and oncology care for advanced cancer on patient and caregiver outcomes | Inception to 21 November 2016, update to July 2018 | Trial or quasi-experimental design, adults with advanced cancer, interventions delivered in outpatient settings, evidence of integration between PC and oncology services, comparator usual oncology care | 8 parallel RCTs, two cluster-RCTs for 10 studies included | All interventions included a multidisciplinary team, were classified as “moderately integrated,” and addressed physical and psychological symptoms. In a meta-analysis, short-term quality of life improved, symptom burden improved, and all-cause mortality decreased.  Ratings of the levels of integration and impact for integrated palliative & oncology care with 8 domains: care teams co-located, written or electronic information exchanged, care teams communication bidirectional information exchanged as standard and routine practice, care providers with equal roles in decision-making, care standardized across all patients, one joint treatment plan for cancer patients  **Quality of life**: Integrated PC improved short-term quality of life (n = 9; SMD 0.24; 95% CI 0.13 to 0.35; I^2^ = 0%). Positive effects were consistent, ranging from small to moderate in all but one study.  At 6–12 months, quality of life was not improved (n = 6; SMD 0.15; 95% CI –0.12 to 0.43; I^2^ = 28%). One study found an interaction effect by cancer type; patients with lung cancer benefitted greater than those with gastrointestinal cancer.  **Symptom burden**: At 1–3 months post-randomization, patients assigned to integrated PC showed small but statistically nonsignificant improvements in symptom burden (n = 6; SMD –0.17; 95% CI –0.45 to 0.11, I^2^ = 62%).  **Psychological symptom outcomes**: 6 studies reported effects on depression symptoms. There was no short-term effect on depressive symptoms reporting severity as a continuous outcome (n = 4; SMD –0.09; 95% CI –0.32 to 0.13; I^2^ = 0%) |
| Gaertner *et al* 2017^36^ | Meta-analysis | To assess the effect of specialist PC on quality of life and additional outcomes relevant to patients in those with advanced illness | Inception till July 2016 | RCTs  Adult in-/ outpatients in hospital hospice, community  advanced illness  receiving specialist PC with a multi-professional team approach | 12 publications included  10 RCTs with 2454 patients, 1766 patients with cancer | Results re-expressed on the global health/quality of life scale of the EORTC QLQ-C30  Advanced cancer: 6 studies, 4 early PC, 10 hospital 7 inpatient, 4 outpatient  **Quality of life:** A small effect in favor of specialist PC (SMD 0.16, 95% CI 0.01 to 0.31; QLQ-C30 global health/QoL 4.1, 0.3 to 8.2; n=1218 patients, 6 trials).  Sensitivity analysis showed an SMD of 0.57 (−0.02 to 1.15; global health/QoL 14.6, −0.5 to 29.4; n=1385, 7 trials). The effect was marginally larger for patients with cancer (0.20, 0.01 to 0.38; global health/QoL 5.1, 0.3 to 9.7; n=828, 5 trials), for those who received specialist PC early (0.33, 0.05 to 0.61, global health/QoL 8.5, 1.3 to 15.6; n=388, 2 trials).  **Symptom burden**: The results for pain and other secondary outcomes were inconclusive.  Some methodological problems (such as lack of blinding).  Pain secondary outcome: SMD negative due to small studies SMD -0.21 (-1.35 to 0..94)  Cancer versus non-cancer studies: cancer SMD 0.20, non-cancer (1 trial) SMD 0.04 |
| Haun *et al* 2017^37^ | Meta-analysis | To compare effects of early PC interventions versus treatment as usual/standard cancer care on health-related quality of life, depression, symptom intensity, and survival among adults with a diagnosis of cancer | Inception to October 2016 | RCTs and cluster randomized trials on professional PC services providing or co-coordinating comprehensive care for adults at early stages of cancer | 7 studies included  RCTs and cluster randomized trials  1614 participants | No study followed the solo practice model, 3 studies used the collaborative care model, 4 studies the integrated care model  Quality of life: FACIT and FACIT-Sp, FACT-L, FACT-Hep, McGill QOL, follow-up range 12 to 52 weeks, standardized mean differences were mostly combined on the Functional Assessment in Cancer Therapy measures and re-expressed in natural units converted to the the FACT-G.  **Quality of life**: early PC improved HrQoL with a small effect size of SMD 0.27 (95% CI 0.15 to 0.38, 1028 participants, low GRADE of evidence); re-expressed in natural units of the FACT-G: average increase by 4.59 (95% CI: 2.55 to 6.46) points  Subgroup analysis in two service models:  Coordinated care model: SMD 0.21 (95% CI: 0.03 to 0.39)  integrated care model: SMD 0.31 (95% CI: 0.15 to 0.46)  **Symptom burden**: early PC resulted in improved symptom burden with lower symptom intensity with a small effect size of SMD -0.23 (95% CI -0.35 to -0.10; 1054 participants, evidence of low certainty)  **Emotional wellbeing:** Levels of depressive symptoms among those receiving early palliative  care did not differ significantly from levels among those receiving usual/standard cancer care (5 studies; SMD -0.11, 95% CI -0.26 to 0.03; participants analyzed at post treatment = 762; evidence of very low certainty). |
| Kassianos *et al* 2018^38^ | Meta-analysis | To critically evaluate the impact of SPC on patients’ HrQoL | Inception to June 2016 | Adult cancer patients  Evaluating interventions aiming to provide SPC to cancer patients by SPC service  All non-specialist interventions excluded  Randomized and non-RCTs including prospective and retrospective studies with pre-post assessment | 11 studies  2939 patients  5 RCTs  6 prospective before-after studies | **Quality of life:** Moderate positive impact of SPC on HrQOL (SMD 0.28, 0.16 to 0.41, statistically significant) (but RCTs and non-RCTs pooled, I² for RCTs 51%, I² for non-RCTs 86%)  Effect modifiers: not shown for RCTs versus non-RCTs, types of cancer, but SMD 0.55 in inpatients or both SMD 0.18, but not significant SMD 0.20 in outpatients (-0.03 to 0.44)  Meta-regression: age and treatment duration not significant predictors of overall effect size  Improved HRQOL in patients with cancer following SPC especially in symptoms like pain, nausea, fatigue, improvement in physical and psychosocial functioning, less or no improvement in social and spiritual domains  Studies of inpatients larger benefit than outpatients  Age, treatment duration did not moderate impact of SPC  SPC intervention clearly described in 2 studies, rest did not report SPC delivery, SPC provided by multi-professional team with specialist training in SPC mostly specified, half of studies reported on which guidelines SPC was based  Almost all studies reported multidisciplinary team  Control groups reported in 4 RCTS as usual care, 5^th^ RCT no information |
| Kavalieratos *et al* 2016^39^ | Meta-analysis | To determine the association of PC with quality of life, symptom burden, survival, and other outcomes for people with life-limiting illness and for their caregivers | Inception to July 2016 | RCTs investigating PC interventions for adult patients with life-threatening illness and 1 of 9 outcomes  Interventions targeting on symptom or one PC domain and not targeting patients were excluded | 43 RCTs  12731 patients  2479 caregivers  Meta-analysis done on 23 trials  35 trials had usual care as control | **Quality of life**: results in the meta-analysis were re-expressed as FACIT-Pal or ESAS units; 24 RCTs reported QoL (12 with high risk of bias); 17 trials reported statistically significant results  1-3 month follow-up with 15 studies for meta-analysis: clinically significant improvements in QOL: SMD 0.46 (95% CI 0.08-0.83, I² = 95%), FACIT-Pal mean difference 11.36  **Symptom burden**: 1-3 month follow up, SMD -0.66 (95% CI -1.25 to -0.07, 1813 participants, I² = 96%), ESAS mean difference -10.30  At the 4- to 6-month follow-up, PC was associated with improved symptom burden (SMD, −0.18; 95% CI, −0.31 to−0.05; I² = 0.0%)  Analysis limited to trials at low risk of bias (n = 5), association between PC and QOL attenuated but remained statistically significant (SMD 0.20 (95% CI 0.06-0.34), mean difference FACIT-Pal 4.94, symptom burden did not remain statistically significant (ESAS SMD -0.21, 95% CI -0.42 to 0.00), ESAS MD -3.28 |
| Latorraca et al 2019^40^ | Narrative synthesis | To assess the effects (benefits and harms) of PC interventions compared to usual care for people with any form of multiple sclerosis | Inception to November 2018 | RCTs and cluster randomized trials or first phase of cross-over trials  Compared PC interventions versus usual care or versus another type of intervention  Adults with diagnosis of multiple sclerosis | 3 studies  146 participants | Two studies compared multidisciplinary, fast-track PC versus multidisciplinary standard care while on a waiting-list control, and one study compared a multidisciplinary palliative approach versus multidisciplinary standard care at different time points (12, 16, and 24 weeks). Two were RCTs with parallel design (total 94 participants) and one was a cross-over design (52 participants). The three studies assessed PC as a home-based intervention.  **Quality of life:** In long-time follow up (> 6 months), no statistically significant difference between intervention and control group on HRQOL SEIQOL with MD 4.80 (95% CI: -12.32 to 21.92) based on 1 study with 58 participants at 24 weeks, very low evidence |
| Quinn *et al* 2020^41^ | Meta-analysis | Is receipt of PC interventions associated with lower acute health care use and better patient-centered outcomes in adults with noncancer illness | Inception to April 2020 | RCTs of PC interventions in adults with noncancer conditions (50% cancer patients – studies excluded)  trials of PC regardless of SPC status, if contained at least 2 of 8 domains outlined in 2018 National Consensus Project on Clinical Practice Guidelines for Quality PC | 28 RCTs  13 664 patients | 28 RCTs data on 13 664 patients (mean age 74 years, 46% women), heart failure, 11 mixed disease, 4 dementia, 3 COPD.  **Quality of life:** Results re-expressed in FACIT-Pal units, 6 studies pooled, PC not associated with disease-generic QOL (6 trials, n = 1334, SMD 0.18 (95% CI -0.24 to 0.61, I² = 87%), FACIT-Pal MD 4.7 (-6.3 to 15.9)) or disease-specific QOL (11 trials, n = 2204, SMD 0.07 (95% CI: -0.09 to 0.23, I² = 68%))  **Symptom burden:** 14 trials, 11 studies (n = 2598) pooled in the meta-analysis; PC was statistically significantly associated with modestly lower symptom burden (11 trials, n = 2598, SMD -0.12 (95% CI -0.20 to -0.03), I² = 0%, ESAS MD -1.6 (95% CI -2.6 to -0.4), translates into an average of a 0.2 point decrease across all subdomains on the ESAS |

*Abbreviations*: CI = confidence interval, CT = controlled clinical trial, EORTC QLQ-C30 = European Organization for the Research and Treatment of Cancer Quality of Life Questionnaire-C30, ESAS = Edmonton Symptom Assessment Scale, FACT-G = Functional Assessment in Cancer Therapy-General, FACT-Hep = Functional Assessment in Cancer Therapy-Hepatobiliary, FACIT-PAl = Functional Assessment in Cancer Therapy-Palliative care, FACIT-Sp = Functional Assessment in Cancer Therapy-Spiritual, FACT-L = Functional Assessment in Cancer Therapy-Lung, GRADE = Grading of Recommendations, Assessment, Development and Evaluation, HrQoL = health-related quality of life, McGill QOL = McGill quality of life questionnaire, MD = mean difference, PC = palliative care, PICO = population, intervention, control, outcome, QoL = quality of life, RCT = randomized controlled trial, SMD = standardized mean difference, SPC = specialist palliative care, US = United States of America

**Table C. Summary of findings from meta-analysis/systematic reviews SPC QOL/symptom burden/psychosocial outcomes – Research in context**

| **Study** | **setting** | **disease group** | **# studies** | **design** | **QOL SMD (95% CI)** | **#n QOL** | **Symptoms SMD** | **#n Symp** | **Psychosocial** | **#n Psych** |
| --- | --- | --- | --- | --- | --- | --- | --- | --- | --- | --- |
| Bajwah *et al* 2020,^28^ Oluyase *et al* 2021^29^ | hospital SPC | all | 42 | RCTs | 0.26 (0.15 to 0.37) | 10 | -0.26  (-0.41 to 0.12) | 6 |  |  |
| Chan *et al* 2023^30^ | SPC interventions | neurodegenera-tive diseases | 12 | All quantita-tive designs | 0.19 (-0.07 to 0.44) | 6 | -0.34 (-0.59 to -0.09) | 9 |  |  |
| Chyr *et al* 2022^31^ | integrated PC home care | noncancer | 14 | All quantita-tive designs | 0.19 (-0.03 to 0.41) | 4 | MD 0.1,  MD -8.26 on ESAS | 2 | Depression: -0.09 (-0.35, 0.16) | 3 RCTs |
| Cui *et al* 2019^32^ | collaborative | CHF | 21 | RCTs | 0.60 (0.27 to 0.94) | 21 |  |  | Anxiety: 0.33 (0.12-0.55) | 4 RCTs |
| Finlay *et al* 2002,^33^ Higginson *et al* 2002^34^ | HSPC | all | 9 | All quantita-tive designs | range 0.04 to 0.53 | 5 | range 0.03 to 2.04 | 8 |  |  |
| Fulton *et al* 2019^35^ | integrated PC outpatient | cancer | 9 | RCTs | 0.24 (0.13-0.35) at 3 months, at 6-12 months not improved | 9 | -0.17  (-0.45, 0.11) | 6 | Depression: -0.09 (-0.32 to 0.13) | 4 |
| Gaertner *et al* 2017^36^ | SPC | all | 10 | RCTs | 0.16 (0.01 to 0.31) | 6 | -0.21  (-1.35 to 0.94) | 1 |  |  |
| Haun *et al* 2017^37^ | early PC | cancer | 7 | RCTs | 0.27 (0.15 to 0.38) integrated care | 4 |  |  |  |  |
| Kassianos *et al* 2018^38^ | SPC | cancer | 11 | All quantita-tive designs | 0.28 (0.16 to 0.41) | 11 (5 RCT, 6 BAS) |  |  |  |  |
| Kavalieratos *et al* 2016^39^ | SPC | all | 23 | RCTs | 1-3 mths: 0.46 (0.08 to 0.83) 4-6 mths: 0.12 (-0.03 to 0.28) | 15, 12 | 1-3 mths: -0.66  (-1.25 to -0.07)  4-6 mths: -0.18  (-0.31 to -0.05) | 1-3: 10  4-6: 6 |  |  |
| Latorraca *et al* 2019^40^ | SPC | multiple sclerosis | 3 | RCTs | MD 4.8 (-12.32 to 21.92) on SEIQOL at 24 weeks | 1 |  |  |  |  |
| Quinn *et al* 2020^41^ | SPC | noncancer | 28 | RCTs | generic QOL 0.18 (-0.24 to 0.61),  disease-specific QOL 0.07 (-0.09 to 0.23) | 6, 11 | -0.12  (-0.20 to -0.03) | 11 |  |  |

*Abbreviations*: BAS = before-after study, CHF = chronic heart failure, CI = confidence interval, ESAS = Edmonton Symptom Assessment Scale, HSPC = hospital-based specialist palliative care, MD = mean difference, PC = palliative care, Psych = psychological/emotional wellbeing, QOL = quality of life, RCT = randomized controlled trial, SEIQOL = Schedule for the Evaluation of Individual Quality of Life, SMD = standardized mean difference, SPC = specialist palliative care, Symp = symptom burden

**References to S2 Appendix**

1. Bakitas MA, Elk R, Astin M, et al. Systematic Review of Palliative Care in the Rural Setting. Cancer Control. 2015;22:450–464.

2. Bradley N, Lloyd-Williams M, Dowrick C. Effectiveness of palliative care interventions offering social support to people with life-limiting illness – a systematic review. Eur J Cancer Care. 2018;27:e12837.

3. Cunningham C, Travers K, Chapman R, et al. Palliative care in the outpatient setting. A comprehensive effectiveness report. Institute for Clinical and Economic Review; London. 2016.

4. Datla S, Verberkt CA, Hoye A, Janssen DJA, Johnson MJ. Multi-disciplinary palliative care is effective in people with symptomatic heart failure: a systematic review and narrative synthesis. Palliat Med. 2019;33:1003–1016.

5. Davies E, Higginson IJ. Systematic review of specialist palliative day-care for adults with cancer. Support Care Cancer. 2005;13:607–627.

6. Davis MP, Temel JS, Balboni T, Glare P. A review of the trials which examine early integration of outpatient and home palliative care for patients with serious illnesses. Ann Palliat Med. 2015;4:99–121.

7. Diop MS, Rudolph JL, Zimmerman KM, Richter MA, Skarf LM. Palliative Care Interventions for Patients with Heart Failure: A Systematic Review and Meta-Analysis. J Palliat Med. 2017;20:84–92.

8. Ferrell BR, Temel JS, Temin S, et al. Integration of Palliative Care Into Standard Oncology Care: American Society of Clinical Oncology Clinical Practice Guideline Update. J Clin Oncol. 2017;35:96–112.

9. Gilbertson-White S, Saeidzadeh S, Yeung CW, Tykol H, Vikas P. Palliative and supportive interventions to improve patient-reported outcomes in rural residents with cancer. J Community Support Oncol. 2017;15:e248–e255.

10. Gomes B, Calanzani N, Curiale V, McCrone P, Higginson IJ. Effectiveness and cost-effectiveness of home palliative care services for adults with advanced illness and their caregivers. Cochrane Database Syst Rev. 2013;6:CD007760.

11. Hall S, Kolliakou A, Petkova H, Froggatt K, Higginson IJ. Interventions for improving palliative care for older people living in nursing care homes. Cochrane Database Syst Rev. 2011;3:CD007132.

12. Health Quality Ontario. Team-Based Models for End-of-Life Care: An Evidence-Based Analysis. Ont Health Technol Assess Ser. 2014;14:1–49.

13. Higginson IJ, Evans CJ. What is the evidence that palliative care teams improve outcomes for cancer patients and their families? Cancer J. 2010;16:423–435.

14. Holmenlund K, Sjøgren P, Nordly M. Specialized palliative care in advanced cancer: What is the efficacy? A systematic review. Palliat Support Care. 2017;15:724–740.

15. Johnston BM, McCauley R, McQuillan R, et al. Effectiveness and cost-effectiveness of out-of-hours palliative care: a systematic review. HRB Open Res. 2020;3:9.

16. Leclerc BS, Blanchard L, Cantinotti M, et al. The effectiveness of interdisciplinary teams in end-of-life palliative care: a systematic review of comparative studies. J Palliat Care. 2014;30:44–54.

17. Lorenz KA, Lynn J, Dy SM, et al. Evidence for improving palliative care at the end of life: a systematic review. Ann Intern Med. 2008;148:147–159.

18. Phongtankuel V, Meador L, Adelman RD, et al. Multicomponent Palliative Care Interventions in Advanced Chronic Diseases: A Systematic Review. Am J Hosp Palliat Care. 2018;35:173–183.

19. Salisbury C, Bosanquet N, Wilkinson EK, et al. The impact of different models of specialist palliative care on patients' quality of life: a systematic literature review. Palliat Med. 1999;13:3–17.

20. Sampson EL, Ritchie CW, Lai R, Raven PW, Blanchard MR. A systematic review of the scientific evidence for the efficacy of a palliative care approach in advanced dementia. Int Psychogeriatr. 2005;17:31–40.

21. Shepperd S, Gonçalves-Bradley DC, Straus SE, Wee B. Hospital at home: home-based end-of-life care. Cochrane Database Syst Rev. 2021;3:CD009231.

22. Singer AE, Goebel JR, Kim YS, et al. Populations and Interventions for Palliative and End-of-Life Care: A Systematic Review. J Palliat Med. 2016;19:995–1008.

23. Stevens E, Martin CR, White CA. The outcomes of palliative care day services: a systematic review. Palliat Med. 2011;25:153–169.

24. Tassinari D, Drudi F, Monterubbianesi MC, et al. Early Palliative Care in Advanced Oncologic and Non-Oncologic Chronic Diseases: A Systematic Review of Literature. Rev Recent Clin Trials. 2016;11:63–71.

25. Thomas RE, Wilson D, Sheps S. A literature review of randomized controlled trials of the organization of care at the end of life. Can J Aging. 2006;25:271–293.

26. Yang GM, Neo SH, Lim SZ, Krishna LK. Effectiveness of Hospital Palliative Care Teams for Cancer Inpatients: A Systematic Review. J Palliat Med. 2016;19:1156–1165.

27. Zimmermann C, Riechelmann R, Krzyzanowska M, Rodin G, Tannock I. Effectiveness of specialized palliative care: a systematic review. JAMA. 2008;299:1698–1709.

28. Bajwah S, Oluyase AO, Yi D, et al. The effectiveness and cost-effectiveness of hospital-based specialist palliative care for adults with advanced illness and their caregivers. Cochrane Database Syst Rev. 2020;9:CD012780.

29. Oluyase AO, Higginson IJ, Yi D, et al. Hospital-based specialist palliative care compared with usual care for adults with advanced illness and their caregivers: a systematic review. Southampton (UK): NIHR Journals Library; 2021.

30. Chan LML, Yan OY, Lee JJJ, et al. Effects of Palliative Care for Progressive Neurologic Diseases: A Systematic Review and Meta-Analysis. J Am Med Dir Assoc. 2023;24:171–184.

31. Chyr LC, DeGroot L, Waldfogel JM, et al. Implementation and effectiveness of integrating palliative care into ambulatory care of noncancer serious chronic illness: mixed methods review and meta-analysis. Ann Fam Med. 2022;20:77–83.

32. Cui X, Dong W, Zheng H, Li H. Collaborative care intervention for patients with chronic heart failure. A systematic review and meta-analysis. Medicine (Baltimore). 2019;98:e14867.

33. Finlay IG, Higginson IJ, Goodwin DM, et al. Palliative care in hospital, hospice, at home: results from a systematic review. Ann Oncol. 2002;13 Suppl 4:257–264.

34. Higginson IJ, Finlay I, Goodwin DM, et al. Do hospital-based palliative teams improve care for patients or families at the end of life? J Pain Symptom Manage. 2002;23:96–106.

35. Fulton JJ, LeBlanc TW, Cutson TM, et al. Integrated outpatient palliative care for patients with advanced cancer: a systematic review and meta-analysis. Palliat Med. 2019;33:123–134.

36. Gaertner J, Siemens W, Meerpohl JJ, et al. Effect of specialist palliative care services on quality of life in adults with advanced incurable illness in hospital, hospice, or community settings: systematic review and meta-analysis. BMJ. 2017;358:j2925.

37. Haun MW, Estel S, Rücker G, et al. Early palliative care for adults with advanced cancer. Cochrane Database Syst Rev. 2017;6:CD011129.

38. Kassianos AP, Ioannou M, Koutsantoni M, Charalambous H. The impact of specialized palliative care on cancer patients' health-related quality of life: a systematic review and meta-analysis. Support Care Cancer. 2018;26:61–79.

39. Kavalieratos D, Corbelli J, Zhang D, et al. Association Between Palliative Care and Patient and Caregiver Outcomes: A Systematic Review and Meta-analysis. JAMA. 2016;316:2104–2114.

40. Latorraca CO, Martimbianco ALC, Pachito DV, et al. Palliative care interventions for people with multiple sclerosis. Cochrane Database Syst Rev. 2019;10:CD012936.

41. Quinn KL, Shurrab M, Gitau K, et al. Association of Receipt of Palliative Care Interventions With Health Care Use, Quality of Life, and Symptom Burden Among Adults With Chronic Noncancer Illness: A Systematic Review and Meta-analysis. JAMA. 2020;324:1439–1450.
