# Supplementary material for: Benefits of specialist palliative care by identifying active ingredients of service composition, structure, and delivery model: A systematic review with meta-analysis and meta-regression
Source: PLoS Med. 2024 Aug 2;21(8):e1004436. doi: 10.1371/journal.pmed.1004436 (PMC11329153; doi:10.1371/journal.pmed.1004436)
Supplement: S3 Appendix — (DOCX) [file pmed.1004436.s003.docx]

**Benefits of specialist palliative care by identifying active ingredients of service composition, structure, and delivery model: A systematic review with meta-analysis and meta-regression**

**S3 Appendix**

Miriam J. Johnson, Leah Rutherford, Anisha Sunny, Sophie Pask, Susanne de Wolf-Linder, Fliss E. M. Murtagh, Christina Ramsenthaler

[hycr22@hyms.ac.uk](mailto:hycr22@hyms.ac.uk)

**Search strategy for database search in MEDLINE**

Below, we present the search strategy for OVID MEDLINE database searches. This search strategy was adapted to each database using medical subject headings and synonyms as per the database’s thesaurus. The search was run on Dec 23, 2022, and updated on Dec 23, 2023.

## Search strategy in OVID MEDLINE

1. exp Palliative Care/

2. palliat*.tw.

3. "advanced disease*".tw.

4. ("end-stage disease*" or "end stage disease* or end-stage illness" or "end stage").tw.

5. ("end of life"):ti,ab,kw

6. Terminally Ill/

7. Terminal Care/

8. (terminal* adj6 care*).tw.

9. ((terminal* adj6 ill*) or terminal-stage* or dying or (close adj6 death)).tw.

10. (terminal* adj6 disease*).tw.

11. (end adj6 life).tw.

12. hospice*.tw.

13. dying.tw.

14. incurable.tw.

15. or/1-14

16. intervention*.tw.

17. care.tw.

18. therap*.tw.

19. support*.tw.

20. service*.tw.

21. or/16-20

22. randomized controlled trial.pt.

23. controlled clinical trial.pt.

24. randomized.ab.

25. placebo.ab.

26. clinical trials as topic.sh.

27. randomly.ab.

28. trial.ti.

29. or/22-28

30. 15 AND 21 AND 29

31. (animal/ or nonhuman/) not human/

32. 30 not 31
