# Supplementary material for: Benefits of specialist palliative care by identifying active ingredients of service composition, structure, and delivery model: A systematic review with meta-analysis and meta-regression
Source: PLoS Med. 2024 Aug 2;21(8):e1004436. doi: 10.1371/journal.pmed.1004436 (PMC11329153; doi:10.1371/journal.pmed.1004436)
Supplement: S4 Appendix — (DOCX) [file pmed.1004436.s004.docx]

**Benefits of specialist palliative care by identifying active ingredients of service composition, structure, and delivery model: A systematic review with meta-analysis and meta-regression**

**S4 Appendix**

Miriam J. Johnson, Leah Rutherford, Anisha Sunny, Sophie Pask, Susanne de Wolf-Linder, Fliss E. M. Murtagh, Christina Ramsenthaler

[hycr22@hyms.ac.uk](mailto:hycr22@hyms.ac.uk)

# Protocol for systematic review/meta-analysis

The full protocol for the systematic review can be viewed in the PROSPERO registration brief under CRD42021292371. Further detail is given in the paper. In this S4 appendix, we detail the methods for the meta-analysis.

**Statistical analysis plan**

For each study, we noted the intervention duration and time points of outcome information. We prespecified the following analyses per time point:

| **Quality of life outcome** | **Emotional wellbeing** |
| --- | --- |
| 2 weeks (minimal endpoint in included studies) to 11 weeks  12 weeks (primary endpoint)  13 to 36 weeks (3 months to 6 months)  37 to 52 weeks (7 months to 1 year) | 2 weeks (minimal endpoint in included studies) to 11 weeks  12 weeks  13 to 36 weeks (3 months to 6 months)  37 to 52 weeks (7 months to 1 year) |

If several outcome measures or subscales were reported that could be eligible, we extracted information on all of them. Minimal important differences (MID) for each outcome measures were systematically identified via PubMed. If possible, the most robust anchor-based and condition-specific MID was used (see S5 Appendix). The MID is defined as the smallest difference that patients consider important.^1^

For each study, endpoint and outcome measure, unadjusted mean changes and standard deviation of mean changes were extracted. Missing mean change or SD_change_ were calculated from reported means and standard deviations. Missing information on means were imputed from medians via the conversion formulae reported in Wan *et al*^2^ and the associated Excel spreadsheet. Missing information on the standard deviation (SD) of each group and the SD_change_ was imputed from standard errors, 95% confidence intervals (CI), interquartile ranges, and *p* values using the conversion formulae reported in the Cochrane handbook^3^ and by Cooper *et al*^4^ If high scores on an outcome measure meant a worse outcome, we inverted the mean change (from a positive to a negative sign or vice versa).

Johnston *et al*^5^ detail to substitute the MID for the usual denominator of the standardized mean difference (SMD), the SD. The authors describe in their article an example based on a Cochrane review of pulmonary rehabilitation for chronic obstructive pulmonary disease^6^ in which the differences in mean changes per intervention and control group is divided by the MID for the respective measurement instrument. Thus, the SMD in MID units is calculated via

$$SMD= \frac{MD}{MID} with MD: \frac{{mean change intervention group}_{baseline, post}-{mean change control group}_{baseline,post}}{MID}$$

In the appendix to the publication,^54^ the following formula is given for calculating the standard error (SE) of this SMD (Var = variance, MD = mean difference, MID = minimal important difference). Both the SMD and the SE(SMD) are calculated for each trial. The MID SMDs are pooled using inverse variance methods.

$$SE\left( \frac{MD}{{MID}_{x}^{2}} \right)=\sqrt{\frac{Var\left( MD \right)}{{MID}_{x}^{2}}}=\frac{SE\left( MD \right)}{{MID}_{x}}$$

Johnston *et al*^5^ give the following guidance as to the interpretation of the resulting SMD in MID units:

- if the pooled SMD in MID units is greater than 1 MID with an accurate estimate of effect, many patients may gain important benefits of treatment;
- if the pooled SMD in MID units is between 0.5 and 1.0, the treatment may benefit an appreciable number of patients
- if the pooled SMD in MID units falls below 0.5, it becomes progressively less likely that an appreciable number of patients benefit from treatment
- point estimates or pooled SMD in MID units approaching 2 MIDs with the lower 95% confidence interval bound being lower than 1 suggests a large effect
- a pooled SMD in MID units between 0 and 1 maay have an important impact on many patients.^7^

Kristian Thorlund and co-authors^8^ present conversion formulae to arrive from a summary estimation based on minimally important differences to relative risks using minimally important differences and the derivation of a number needed to treat (NNT). The first step is the conversion of the summary statistics to probabilities and risk differences or relative risks and, ultimately, the NNT.

First, the probability for a benefit in the control group is calculated via the formula

$$p_{control}=1-\Phi\left( \frac{MID-M_{control}}{{SD}_{control}} \right)$$

with $p_{control}$ being the probability of a response in the control group, $\Phi$ being the standard normal distribution, $MID$ being the minimal important difference, $M_{control}$ being the mean change in the control group between baseline and the post-intervention time point, and ${SD}_{control}$ being the standard deviation of change in the control group between baseline and the post-intervention time point. By the same logic, the probability of a response can be calculated for the intervention group, a risk difference can be derived and the corresponding standard error for the risk difference can be calculated according to formula 8 on page 196 in Thorlund *et al*^8^ The relative risk is determined via

$$RR= \frac{p_{intervention}}{p_{control}}$$

with standard error

$$SE\left( \ln\left( RR \right) \right)= \sqrt{\frac{1}{p_{control}\cdot n_{control}}+\frac{1}{p_{intervention}\cdot n_{intervention}}-\frac{1}{n_{control}}-\frac{1}{n_{intervention}}}$$

The NNT is then derived as

$$NNT = \frac{1}{p_{control}\cdot(1-RR)}$$

with *RR* being the relative risk.

Outcomes were explored further using either univariate linear meta-regression (continuous variables with the SMD in MID units) or subgroup analysis with Mixed-Effects Model (categorical variables with with the SMD in MID units) when there were a minimum of 10 trials for the outcome of interest. We used meta-regression with fewer trials, but considered the effects on statistical power in the interpretation.

The individual trial SMD in MID units were pooled using the Hartung-Knapp random-effects meta-analyses approach via the meta and metabin commands in the meta package^9^ in R v4.0.1.^10^ Other packages used were the metafor,^11^ janitor,^12^ car,^13^ and the tidyverse package.^14^ All R scripts can be accessed via the Open Science Framework repository for this project: <https://osf.io/h8pmz/>.

**References to S4 Appendix**

1. Guyatt GH, Osoba D, Wu AW, Wyrwich KW, Norman GR. Methods to explain the clinical significance of health status measures. Mayo Clinic Proceedings. 2002;77(4):371–383.

2. Wan X, Wang W, Liu J, Tong T. Estimating the sample mean and standard deviation from the sample size, median, range and/or interquartile range. BMC Med Res Methodol. 2014;14:135.

3. Higgins JPT, Thomas J, Chandler J, et al. Cochrane Handbook for Systematic Reviews of Interventions version 6.3 (updated February 2022). Cochrane, 2022. www.training.cochrane.org/handbook. Accessed April 23, 2023.

4. Cooper H, Hedges LV, Valentine JC. The handbook of research synthesis and meta-analysis. Russell Sage Foundation. 2009.

5. Johnston BC, Thorlund K, Schünemann HJ, et al. Improving the interpretation of quality of life evidence in meta-analyses: the application of minimal important difference units. Health Qual Life Outcomes. 2010;8:116.

6. Lacasse Y, Goldstein RS, Lasserson TJ, Martin S. Pulmonary rehabilitation for chronic obstructive pulmonary disease. Cochrane Database Syst Rev. 2006;18:CD003793.

7. Guyatt GH, Juniper EF, Walter SD, Griffith LE, Goldstein RS. Interpreting treatment effects in randomized trials. BMJ. 1998;316:690–693.

8. Thorlund K, Walter SD, Johnston BC, Furukawa TA, Guyatt GH. Pooling health-related quality of life outcomes in meta-analysis – a tutorial and review of methods for enhancing interpretability. Res Synth Methods. 2011;2:188-203.

9. Balduzzi S, Rücker G, Schwarzer G. How to perform meta-analysis with R: a practical tutorial. Evid Based Ment Health. 2019;22:153–160.

10. R Core Team. R: A language and environment for statistical computing. R Foundation for Statistical Computing. Vienna, Austria; 2022. Available at: https://www.R-project.org/. Accessed August 3, 2023.

11. Viechtbauer W. Conducting meta-analyses in R with the metafor package. J Stat Softw. 2010;36:1–48.

12. Firke S. janitor: simple tools for examining and cleaning dirty data. R package version 2.1.0. https://CRAN.R-project.org/package=janitor. Accessed August 3, 2023.

13. Fox J, Weisberg S. An R companion to applied regression, 3rd edition. Thousand Oaks: Sage; 2019.

14. Wickham H, et al. Welcome to the tidyverse. J Open Source Softw. 2019;4:1686.
