# Supplementary material for: Benefits of specialist palliative care by identifying active ingredients of service composition, structure, and delivery model: A systematic review with meta-analysis and meta-regression
Source: PLoS Med. 2024 Aug 2;21(8):e1004436. doi: 10.1371/journal.pmed.1004436 (PMC11329153; doi:10.1371/journal.pmed.1004436)
Supplement: S5 Appendix — (DOCX) [file pmed.1004436.s005.docx]

**Benefits of specialist palliative care by identifying active ingredients of service composition, structure, and delivery model: A systematic review with meta-analysis and meta-regression**

**S5 Appendix**

Miriam J. Johnson, Leah Rutherford, Anisha Sunny, Sophie Pask, Susanne de Wolf-Linder, Fliss E. M. Murtagh, Christina Ramsenthaler

[hycr22@hyms.ac.uk](mailto:hycr22@hyms.ac.uk)

**Tables with MIDs per outcome measure**

#

**Table A. MIDs per outcome measure (a) Primary outcome: Quality of life**

| **Instrument** | **Trial*** | **Condition** | **Reference MID** | **MID** | **Notes** | **Type of MID** |
| --- | --- | --- | --- | --- | --- | --- |
| **Chronic Heart Failure Questionnaire**  **CHF** | Wong 2016^1^ | CHF | O'Keeffe ST, Lye M, Donnellan C, Carmichael DN. Reproducibility and responsiveness of quality of life assessment and six minute walk test in elderly heart failure patients. *Heart*. 1998;80(4):377-382. doi:10.1136/hrt.80.4.377 | 1.4 | 1.4 is the score of being „a bit better“ in responsiveness analyses determined to global rating of change; there is no anchor-based approach | Distribution-based approach |
| **EORTC QLQ-C30 GHS (global health / quality of life subscale)** | Brims 2019^2^ | Mesothe-lioma | Maringwa JT, Quinten C, King M, et al. Minimal important differences for interpreting health-related quality of life scores from the EORTC QLQ-C30 in lung cancer patients participating in randomized controlled trials. *Support Care Cancer*. 2011;19(11):1753-1760. doi:10.1007/s00520-010-1016-5 | 4.4 | Anchor: change in performance status, weight gain | Anchor-based |
|  | Groenvold 2017^3^ | Cancer |  | 4.4 |  |  |
|  | Liu 2022^4^ | Cancer |  | 4.4 |  |  |
|  | Nottelmann 2021^5^ | Cancer |  | 4.4 |  |  |
|  | Slama 2020^6^ | Cancer |  | 4.4 |  |  |
|  | Vanbutsele 2020^7^ | Cancer |  | 4.4 |  |  |
|  | Woo 2019^8^ | Cancer |  | 4.4 |  |  |
| **EORTC QLQ-C15PAL total score/global** | do Carmo 2017^9^ | Cancer | At the time of the meta-analysis, there was no MCID study for the C15PAL that evaluated the global health/QOL domain. In studies by Bedard and Pilz *et al*, this domain had been used for determining the clinically important changes as a reference category.  We therefore used the same MCID since this subscale is similar to the QLQ-C30. | 4.4 | Anchor: change in performance status, weight gain | Anchor-based |
| **EQ5D Index and VAS** | Brännström 2014^10^ | CHF | Pickard AS, Neary MP, Cella D. Estimation of minimally important differences in EQ-5D utility and VAS scores in cancer [published correction appears in Health Qual Life Outcomes. 2010;8:4]. *Health Qual Life Outcomes*. 2007;5:70. Published 2007 Dec 21. doi:10.1186/1477-7525-5-70  Tsiplova K, Pullenayegum E, Cooke T, Xie F. EQ-5D-derived health utilities and minimally important differences for chronic health conditions: 2011 Commonwealth Fund Survey of Sicker Adults in Canada. *Qual Life Res*. 2016;25(12):3009-3016. doi:10.1007/s11136-016-1336-0 | Index:  0.017,  VAS:  9 | Performance status as anchor  Performance status (Eastern Cooperative Oncology Group, ECOG) (for VAS)  Global health question, FACT-G total score-based quintiles (for heart disease EQ5D index score) | Anchor-based |
| **Edmonton Symptom Assessment System (ESAS) total score** | Bakitas 2009^11^ | Cancer | Hui D, Shamieh O, Paiva CE, et al. Minimal Clinically Important Difference in the Physical, Emotional, and Total Symptom Distress Scores of the Edmonton Symptom Assessment System*. J Pain Symptom Manage*. 2016;51(2):262-269. doi:10.1016/j.jpainsymman.2015.10.004 | 5.7 | Anchor-based method according to patient’s global rating of change (better, about the same, worse), MCID based on patient-change approach.  MID only available for cancer patients, no MID work for chronic heart failure | Anchor-based |
|  | Benthien 2020^12^ | Cancer |  | 5.7 |  |  |
|  | El-Jahwari 2016^13^ | HSCT |  | 5.7 |  |  |
|  | El-Jahwari 2021^14^ | AML |  | 5.7 |  |  |
|  | Hoek 2017^15^ | Cancer |  | 5.7 |  |  |
|  | Sidebottom 2015^16^ | CHF |  | 5.7 |  |  |
|  | Wong 2016^1^ | CHF |  | 5.7 |  |  |
|  | Zimmermann 2014^17^ | Cancer |  | 5.7 |  |  |
| **Edmonton Symptom Assessment Scale-Revised Parkinson’s Disease (ESAS PD)** | Kluger 2020^18^ | Parkinson’s Disease | Hui D, Shamieh O, Paiva CE, et al. Minimal Clinically Important Difference in the Physical, Emotional, and Total Symptom Distress Scores of the Edmonton Symptom Assessment System. *J Pain Symptom Manage*. 2016;51(2):262-269. doi:10.1016/j.jpainsymman.2015.10.004 | 5.7 | Although the ESAS PD total score is formed from 14 items and not 9 items as in the ESAS, no MID work has been done. Since there was a lack of responsiveness studies, the MID from the cancer population was used for this measure. | Anchor-based |
| **FACIT-PAL TOI** | Bakitas 2015^19^ | Cancer | No MID for the FACIT-PAL exists. We used the sample size calculation in this trial to determine the SD as 17. Using Norman’s rule of half a standard deviation, we determined the MID as the rounded value.  Norman GR, Sloan JA, Wyrwich KW. Interpretation of changes in health-related quality of life: the remarkable universality of half a standard deviation. *Med Care*. 2003;41(5):582-592. doi:10.1097/01.MLR.0000062554.74615.4C  Also, change scores according to a global rating of change are reported in King MT, Agar M, Currow DC, Hardy J, Fazekas B, McCaffrey N. Assessing quality of life in palliative care settings: head-to-head comparison of four patient-reported outcome measures (EORTC QLQ-C15-PAL, FACT-Pal, FACT-Pal-14, FACT-G7). *Support Care Cancer*. 2020;28(1):141-153. doi:10.1007/s00520-019-04754-9 | 9 | Distribution-based MID based on sample size calculation in Bakitas 2015 and in King et al (2020) (global patient-rated change | Distribution-based |
| **FACIT-PAL46 / FACIT-PAL14** | Bakitas 2009^11^ | Cancer | King MT, Agar M, Currow DC, Hardy J, Fazekas B, McCaffrey N. Assessing quality of life in palliative care settings: head-to-head comparison of four patient-reported outcome measures (EORTC QLQ-C15-PAL, FACT-Pal, FACT-Pal-14, FACT-G7). *Support Care Cancer*. 2020;28(1):141-153. doi:10.1007/s00520-019-04754-9 | 5 | Distribution based, global rating of change, extrapolated from MID ~ 5 for FACIT-PAL for improved on the global rating of change | Distribution-based |
|  | Bakitas 2015^19^ | Cancer |  | 5 |  |  |
| **FACIT-Sp** | Zimmermann 2014^17^ | Cancer | King MT, Agar M, Currow DC, Hardy J, Fazekas B, McCaffrey N. Assessing quality of life in palliative care settings: head-to-head comparison of four patient-reported outcome measures (EORTC QLQ-C15-PAL, FACT-Pal, FACT-Pal-14, FACT-G7). *Support Care Cancer*. 2020;28(1):141-153. doi:10.1007/s00520-019-04754-9 | 9 | Distribution-based, global rating of change | Distribution-based |
| **FACT H&N total** | Patil 2021^20^ | Cancer | Webster K, Cella D, Yost K. The Functional Assessment of Chronic Illness Therapy (FACIT) Measurement System: properties, applications, and interpretation. *Health Qual Life Outcomes*. 2003;1:79. Published 2003 Dec 16. doi:10.1186/1477-7525-1-79 | 6 | MID 6-12 points, lower threshold taken | Anchor-based |
| **FACT-B** | Greer 2022^21^ | Metastatic breast cancer | Eton DT, Cella D, Yost KJ, et al. A combination of distribution- and anchor-based approaches determined minimally important differences (MIDs) for four endpoints in a breast cancer scale. *J Clin Epidemiol*. 2004;57(9):898-910. doi:10.1016/j.jclinepi.2004.01.012 | 7 | Anchor-based, anchor: Performance status, physician  assessment of current pain, and  response to treatment | Anchor-based |
| **FACT-BMT** | El-Jahwari 2016^13^ | HSCT | McQuellon RP, Russell GB, Cella DF, et al. Quality of life measurement in bone marrow transplantation: development of the Functional Assessment of Cancer Therapy-Bone Marrow Transplant (FACT-BMT) scale. *Bone Marrow Transplant*. 1997;19(4):357-368. doi:10.1038/sj.bmt.1700672 Also Eton *et al* (2004) see above | 3 | Anchor-based, anchor: Performance status, physician  assessment of current pain, and  response to treatment | Anchor-based |
| **FACT-G** | Eychmüller 2021^22^ | Cancer | Cella D, Hahn EA, Dineen K. Meaningful change in cancer-specific quality of life scores: differences between improvement and worsening. *Qual Life Res*. 2002;11(3):207-221. doi:10.1023/a:1015276414526  Brucker PS, Yost K, Cashy J, Webster K, Cella D. General population and cancer patient norms for the Functional Assessment of Cancer Therapy-General (FACT-G) [published correction appears in Eval Health Prof. 2005 Sep;28(3):370]. *Eval Health Prof*. 2005;28(2):192-211. doi:10.1177/0163278705275341 | 5 | Anchor: performance status | Anchor-based |
|  | Franciosi 2019^23^ | Cancer |  | 5 |  |  |
|  | Scarpi 2019^24^ | Gastric cancer |  | 5 |  |  |
|  | Temel 2020^25^ | Cancer |  | 5 |  |  |
|  | Patil 2021^20^ | Cancer |  | 5 |  |  |
|  | Bekelman 2022^26^ | CHF, COPD, ILD |  | 5 |  |  |
| **FACT-GA** | Scarpi 2019^24^ | Gastric cancer | Garland SN, Pelletier G, Lawe A, et al. Prospective evaluation of the reliability, validity, and minimally important difference of the functional assessment of cancer therapy-gastric (FACT-Ga) quality-of-life instrument. *Cancer*. 2011;117(6):1302-1312. doi:10.1002/cncr.25556 | 6 | Anchors: clinician-rated Karnofsky performance status | Anchor-based |
| **FACT-HEP** | Maltoni 2016^27^ | Gastric cancer | Steel JL, Eton DT, Cella D, Olek MC, Carr BI. Clinically meaningful changes in health-related quality of life in patients diagnosed with hepatobiliary carcinoma. *Ann Oncol*. 2006;17(2):304-312. doi:10.1093/annonc/mdj072 | 8 | Alpha-fetoprotein, alkaline  phosphate and hemoglobin  levels, survival | Anchor-based |
| **FACT-L** | Temel 2010^28^ | Cancer | Cella D, Eton DT, Fairclough DL, et al. What is a clinically meaningful change on the Functional Assessment of Cancer Therapy-Lung (FACT-L) Questionnaire? Results from Eastern Cooperative Oncology Group (ECOG) Study 5592. *J Clin Epidemiol*. 2002;55(3):285-295. doi:10.1016/s0895-4356(01)00477-2 | 2.4 | Anchor: best overall response to treatment, time to disease progression | Anchor-based |
| **FACT-Leu** | El-Jahwari 2021^14^ | AML | Peipert JD, Yount SE, Efficace F, et al. Validation of the Functional Assessment of Cancer Therapy-Leukemia instrument in patients with acute myeloid leukemia who are not candidates for intensive therapy. *Cancer*. 2020;126(15):3542-3551. doi:10.1002/cncr.32977 | 2.5 | Anchor-based: anchoring change in the FACT-Leu scales to a 0.10 change in the EQ-5D Health Utility Index, midpoint of MID | Anchor-based |
| **IPOS Neuro**  **S-8** | Gao 2020^29^ | Neuro | MID estimated from control group confidence interval of change in etable 3 in publication and using Norman’s rule of half a standard deviation | 1.1 | Distribution-based | Distribution-based |
| **IPOS-5** | Evans 2021^30^ | Older noncancer | The MID has not been determined. We used the estimate of a minimum detectable change of 2 points from the article’s sample size calculation. | 2 | Distribution-based | Distribution-based |
| **KCCQ** | Bakitas 2020^31^ | CHF | Spertus J, Peterson E, Conard MW, et al. Monitoring clinical changes in patients with heart failure: a comparison of methods. *Am Heart J*. 2005;150(4):707-715. doi:10.1016/j.ahj.2004.12.010 | 5 | Anchor-based MID: physician-assessed functional class (New York Heart Association [NYHA]), an exercise test (6-minute walk), patient weight, and a biomarker (B-type natriuretic peptide) | Anchor-based |
|  | Bekelman 2018^32^ | CHF |  | 5 |  |  |
|  | Rogers 2017^33^ | CHF |  | 5 |  |  |
| **Maugeri Respiratory QoL revised** | Bassi 2021^34^ | ILD | Coquart JB, Heutte N, Terce G, Grosbois JM. Convergent Validity and Minimal Clinically Important Difference of the Maugeri Foundation Respiratory Failure Questionnaire (MRF-28) and the Chronic Obstructive Pulmonary Disease-Specific Health-Related Quality of Life questionnaire (VQ11). *Int J Chron Obstruct Pulmon Dis*. 2019;14:2895-2903. Published 2019 Dec 13. doi:10.2147/COPD.S222165 | 3 | Anchor-based, patient-rated change | Anchor-based |
| **McGill QoL** | Tattersall 2014^35^ | Cancer | No responsiveness study could be found. We therefore used the reported data in Wong 2016 to estimate an SD_change_ from which the MID was calculated according to Norman’s rule. | 3 | Distribution-based from reported study data. | Distribution-based |
|  | Wong 2016^1^ | CHF |  | 3 |  |  |
|  | Goldstein 2022^36^ | Organ failure |  | 3 |  |  |
| **Minnesota Living with HF total** | Sidebottom 2015^16^ | CHF | Gonzalez-Saenz de Tejada M, Bilbao A, Ansola L, et al. Responsiveness and minimal clinically important difference of the Minnesota living with heart failure questionnaire. *Health Qual Life Outcomes*. 2019;17(1):36. Published 2019 Feb 14. doi:10.1186/s12955-019-1104-2 | 3.59 | Anchor-based MID, anchor was global rating of change (3-point); Lower point taken from: MCID – 3.59-19.14 points | Anchor-based |
| **MSIS Physical subscale** | Edmonds 2010^37^ | MS | Costelloe L, O'Rourke K, Kearney H, et al. The patient knows best: significant change in the physical component of the Multiple Sclerosis Impact Scale (MSIS-29 physical). *J Neurol Neurosurg Psychiatry*. 2007;78(8):841-844. doi:10.1136/jnnp.2006.105759 | 8 | Anchor-based MID, anchor: change on the Expanded Disability Status Scale | Anchor-based |
| **MS-POS** | Edmonds 2010^37^ | MS | No MID has been determined. The MID was taken from the SD_change_ of 7.29 for the control group reported in the article and using Norman’s rule of half an SD. | 4 | Distribution-based | Distribution-based |
| **QOL-AD** | Kluger 2020^18^ | Parkinson | Holden SK, Koljack CE, Prizer LP, Sillau SH, Miyasaki JM, Kluger BM. Measuring quality of life in palliative care for Parkinson's disease: A clinimetric comparison. *Parkinsonism Relat Disord*. 2019;65:172-177. doi:10.1016/j.parkreldis.2019.06.018 | 3.9 | Anchor-based MID, anchor global rating of change (3-point) | Anchor-based |
| **SF-36 General health** | Aiken 2006^38^ | CHF and COPD | Jayadevappa R, Malkowicz SB, Wittink M, Wein AJ, Chhatre S. Comparison of distribution- and anchor-based approaches to infer changes in health-related quality of life of prostate cancer survivors. *Health Serv Res*. 2012;47(5):1902-1925. doi:10.1111/j.1475-6773.2012.01395.x | 8 | Anchor: A patient-reported physical signs/symptoms (more tired or worn out than usual) | Anchor-based |
| **SF-36 physical role** | Given 2002^39^ | Cancer | Jayadevappa R, Malkowicz SB, Wittink M, Wein AJ, Chhatre S. Comparison of distribution- and anchor-based approaches to infer changes in health-related quality of life of prostate cancer survivors. *Health Serv Res*. 2012;47(5):1902-1925. doi:10.1111/j.1475-6773.2012.01395.x | 14 | Anchor: A patient-reported physical signs/symptoms (more tired or worn out than usual) | Anchor-based |
|  | Aiken 2006^38^ | CHF,COPD |  | 14 |  |  |

*Reference numbers for trials are different to those of the main publication, see end of S5 for the reference list.

**Table B. MIDs per outcome measure (b) Secondary outcome: Emotional well-being**

| **Instrument** | **Trial*** | **Condition** | **Reference MID** | **MID** | **Notes** | **Type of MID** |
| --- | --- | --- | --- | --- | --- | --- |
| **Center of Epidemiologic Studies-Depression Scale**  **CES-D**  **(20 item version)** | Bakitas 2009^11^ | Cancer | Haase I, Winkeler M, Imgart H. Ankerbasierte Ermittlung klinisch relevanter Veränderung depressiver Symptomatik am Beispiel der Kurzform der CES-D [Anchor-based ascertaining of meaningful changes in depressive symptoms using the example of the German short form of the CES-D]. *Neuropsychiatr*. 2016;30(2):82-91. doi:10.1007/s40211-016-0184-z | 4.82 | Anchor-based MCID to global impression of change question only for psychosomatic sample, not cancer.  Small change MCID was 3, smallest detectable change was 4.82 | Anchor-based |
| **EORTC C30 Emotional function** | Groenvold 2017^3^ | Cancer | Hong F, Bosco JL, Bush N, Berry DL. Patient self-appraisal of change and minimal clinically important difference on the European organization for the research and treatment of cancer quality of life questionnaire core 30 before and during cancer therapy. *BMC Cancer*. 2013;13:165. Published 2013 Mar 28. doi:10.1186/1471-2407-13-165  Bedard G, Zeng L, Zhang L, et al. Minimal important differences in the EORTC QLQ-C30 in patients with advanced cancer. *Asia Pac J Clin Oncol*. 2014;10(2):109-117. doi:10.1111/ajco.12070 | 4 | Anchor: global change rating of change, minimum -maximum for all cancer MIDs was 2.9 to 6.9, mid-point taken | Anchor-based |
|  | Woo 2019^8^ | Cancer |  | 4 |  |  |
|  | Slama 2020^6^ | Cancer |  | 4 |  |  |
|  | Liu 2022^4^ | Cancer |  | 4 |  |  |
| **HADS total** | Slama 2020^6^ | Cancer | Puhan MA, Frey M, Büchi S, Schünemann HJ. The minimal important difference of the hospital anxiety and depression scale in patients with chronic obstructive pulmonary disease. Health Qual Life Outcomes. 2008;6:46. Published 2008 Jul 2. doi:10.1186/1477-7525-6-46 | 2 | Anchors: response to treatment, global rating of change, having an MID change on the Chronic Respiratory Questionnaire and the Feeling Thermometer | Anchor-based |
| **HADS-A** | Bakitas 2020^31^ | CHF | Puhan MA, Frey M, Büchi S, Schünemann HJ. The minimal important difference of the hospital anxiety and depression scale in patients with chronic obstructive pulmonary disease. *Health Qual Life Outcomes*. 2008;6:46. Published 2008 Jul 2. doi:10.1186/1477-7525-6-46  Lemay KR, Tulloch HE, Pipe AL, Reed JL. Establishing the Minimal Clinically Important Difference for the Hospital Anxiety and Depression Scale in Patients With Cardiovascular Disease. *J Cardiopulm Rehabil Prev*. 2019;39(6):E6-E11. doi:10.1097/HCR.0000000000000379  Wynne SC, Patel S, Barker RE, et al. Anxiety and depression in bronchiectasis: Response to pulmonary rehabilitation and minimal clinically important difference of the Hospital Anxiety and Depression Scale. *Chron Respir Dis*. 2020;17:1479973120933292. doi:10.1177/1479973120933292  Hansen H, Beyer N, Frølich A, Godtfredsen N, Bieler T. Inter-Day Test-Retest Reproducibility of the CAT, CCQ, HADS and EQ-5D-3L in Patients with Severe and Very Severe COPD. *Patient Relat Outcome Meas*. 2021;12:117-128. Published 2021 Jun 1. doi:10.2147/PROM.S306352  Edwards GD, Polgar O, Patel S, et al. Mood disorder in idiopathic pulmonary fibrosis: response to pulmonary rehabilitation. *ERJ Open Res*. 2023;9(3):00585-2022. Published 2023 May 22. doi:10.1183/23120541.00585-2022 | 1.32 | Variety of anchor-based and distribution-based approaches.  Anchors: response to treatment, global rating of change, having an MID change on the Chronic Respiratory Questionnaire and the Feeling Thermometer | Anchor-based |
|  | do Carmo 2017^9^ | Cancer |  | 1.32 |  |  |
|  | El-Jahwari 2016^13^ | HSCT |  | 1.32 |  |  |
|  | El-Jahwari 2021^14^ | AML |  | 1.32 |  |  |
|  | Gao 2020^29^ | Neuro |  | 1.32 |  |  |
|  | Groenvold 2017^3^ | Cancer |  | 1.32 |  |  |
|  | Hoek 2017^15^ | Cancer |  | 1.32 |  |  |
|  | Kluger 2020^18^ | Parkinson |  | 1.32 |  |  |
|  | Rogers 2017^33^ | CHF |  | 1.32 |  |  |
|  | Temel 2020^25^ | Cancer |  | 1.32 |  |  |
|  | Slama 2020^6^ | Cancer |  | 1.32 |  |  |
|  | Greer 2022^21^ | Metastatic breast cancer |  | 1.32 |  |  |
| **HADS-D** | Bakitas 2020^31^ | CHF | Puhan MA, Frey M, Büchi S, Schünemann HJ. The minimal important difference of the hospital anxiety and depression scale in patients with chronic obstructive pulmonary disease. *Health Qual Life Outcomes*. 2008;6:46. Published 2008 Jul 2. doi:10.1186/1477-7525-6-46  Lemay KR, Tulloch HE, Pipe AL, Reed JL. Establishing the Minimal Clinically Important Difference for the Hospital Anxiety and Depression Scale in Patients With Cardiovascular Disease. *J Cardiopulm Rehabil Prev*. 2019;39(6):E6-E11. doi:10.1097/HCR.0000000000000379  Wynne SC, Patel S, Barker RE, et al. Anxiety and depression in bronchiectasis: Response to pulmonary rehabilitation and minimal clinically important difference of the Hospital Anxiety and Depression Scale. *Chron Respir Dis*. 2020;17:1479973120933292. doi:10.1177/1479973120933292  Hansen H, Beyer N, Frølich A, Godtfredsen N, Bieler T. Inter-Day Test-Retest Reproducibility of the CAT, CCQ, HADS and EQ-5D-3L in Patients with Severe and Very Severe COPD. *Patient Relat Outcome Meas*. 2021;12:117-128. Published 2021 Jun 1. doi:10.2147/PROM.S306352  Edwards GD, Polgar O, Patel S, et al. Mood disorder in idiopathic pulmonary fibrosis: response to pulmonary rehabilitation. *ERJ Open Res*. 2023;9(3):00585-2022. Published 2023 May 22. doi:10.1183/23120541.00585-2022 | 1.4 | Variety of anchor-based and distribution-based approaches.  Anchors: response to treatment, global rating of change, having an MID change on the Chronic Respiratory Questionnaire and the Feeling Thermometer | Anchor-based |
|  | do Carmo 2017^9^ | Cancer |  | 1.4 |  |  |
|  | El-Jahwari 2016^13^ | HSCT |  | 1.4 |  |  |
|  | El-Jahwari 2021^14^ | AML |  | 1.4 |  |  |
|  | Gao 2020^29^ | Neuro |  | 1.4 |  |  |
|  | Groenvold 2017^3^ | Cancer |  | 1.4 |  |  |
|  | Hoek 2017^15^ | Cancer |  | 1.4 |  |  |
|  | Kluger 2020^18^ | Parkinson |  | 1.4 |  |  |
|  | Rogers 2017^33^ | CHF |  | 1.4 |  |  |
|  | Temel 2020^25^ | Cancer |  | 1.4 |  |  |
|  | Slama 2020^6^ | Cancer |  | 1.4 |  |  |
|  | Greer 2022^21^ | Metastatic breast cancer |  | 1.4 |  |  |
| **IPOS psychosocial** | Evans 2021^30^ | Older noncancer | The MID has not been determined. We used the trial data to determine the SD_change_ and the rule of ½ an SD. | 4 | Distribution-based | Distribution-based |
| **MSIS Psychological subscale** | Edmonds 2010^37^ | MS | No MID study for the psychological subscale, inferred from Costelloe L, O'Rourke K, Kearney H, et al. The patient knows best: significant change in the physical component of the Multiple Sclerosis Impact Scale (MSIS-29 physical). *J Neurol Neurosurg Psychiatry*. 2007;78(8):841-844. doi:10.1136/jnnp.2006.105759 | 8 | (Anchor-based) | (Anchor-based) |
| **NCCN Distress Thermometer** | Eychmüller 2021^22^ | Cancer | Head BA, Schapmire TJ, Keeney CE, et al. Use of the Distress Thermometer to discern clinically relevant quality of life differences in women with breast cancer. *Qual Life Res*. 2012;21(2):215-223. doi:10.1007/s11136-011-9934-3  Cutillo A, O'Hea E, Person S, Lessard D, Harralson T, Boudreaux E. The Distress Thermometer: Cutoff Points and Clinical Use. *Oncol Nurs Forum*. 2017;44(3):329-336. doi:10.1188/17.ONF.329-336 | 3 | Both studies used distribution-based approaches to other measures, mainly quality of life. | Distribution-based |
| **PHQ-9** | Bekelman 2018^32^ | CHF | Löwe B, Unützer J, Callahan CM, Perkins AJ, Kroenke K. Monitoring depression treatment outcomes with the patient health questionnaire-9. *Med Care*. 2004;42(12):1194-1201. doi:10.1097/00005650-200412000-00006 | 5 | Distribution-based from change with 2 x standard error of measurement | Distribution-based |
|  | do Carmo 2017^9^ | Cancer |  | 5 |  |  |
|  | El-Jahwari 2016^13^ | HSCT |  | 5 |  |  |
|  | El-Jahwari 2021^14^ | AML |  | 5 |  |  |
|  | Sidebottom 2015^16^ | CHF |  | 5 |  |  |
| **RSCL psychological** | Tattersall 2014^35^ | Cancer | No MID available, determined from the normative SD described in the manual and used Norman’s rule of ½ SD | 10.5 | Distribution-based | Distribution-based |
| **SF-36 mental** | Brims 2019^2^ | Mesothelioma | Ogura K, Yakoub MA, Christ AB, et al. What Are the Minimum Clinically Important Differences in SF-36 Scores in Patients with Orthopaedic Oncologic Conditions?. *Clin Orthop Relat Res*. 2020;478(9):2148-2158. doi:10.1097/CORR.0000000000001341 | 5 | Anchor-based MID: Midpoint between improvement and deterioration (4 to 6). Anchor: patient rating of change (5-point scale) | Anchor-based |

*Reference numbers for trials are different to those of the main publication, see end of S5 for the reference list.

**References for S5 Appendix**

1. Wong FK, Ng AY, Lee PH, et al. Effects of a transitional palliative care model on patients with end-stage heart failure: a randomised controlled trial. Heart. 2016;102(14):1100-1108. doi:10.1136/heartjnl-2015-308638

2. Brims F, Gunatilake S, Lawrie I, et al. Early specialist palliative care on quality of life for malignant pleural mesothelioma: a randomised controlled trial. Thorax. 2019;74(4):354-361. doi:10.1136/thoraxjnl-2018-212380

3. Groenvold M, Petersen MA, Damkier A, et al. Randomised clinical trial of early specialist palliative care plus standard care versus standard care alone in patients with advanced cancer: The Danish Palliative Care Trial. Palliat Med. 2017;31(9):814-824. doi:10.1177/0269216317705100

4. Liu Y, Shen Y, Pan Q, et al. Application of interdisciplinary collaborative hospice care for terminal geriatric cancer patients: a prospective randomized controlled study. Support Care Cancer. 2022;30(4):3553-3561. doi:10.1007/s00520-022-06816-x

5. Nottelmann L, Groenvold M, Vejlgaard TB, Petersen MA, Jensen LH. Early, integrated palliative rehabilitation improves quality of life of patients with newly diagnosed advanced cancer: The Pal-Rehab randomized controlled trial. Palliat Med. 2021;35(7):1344-1355. doi:10.1177/02692163211015574

6. Slama O, Pochop L, Sedo J, et al. Effects of Early and Systematic Integration of Specialist Palliative Care in Patients with Advanced Cancer: Randomized Controlled Trial PALINT. J Palliat Med. 2020;23(12):1586-1593. doi:10.1089/jpm.2019.0697

7. Vanbutsele G, Van Belle S, Surmont V, et al. The effect of early and systematic integration of palliative care in oncology on quality of life and health care use near the end of life: A randomised controlled trial. Eur J Cancer. 2020;124:186-193. doi:10.1016/j.ejca.2019.11.009

8. Woo SM, Song MK, Lee M, et al. Effect of Early Management on Pain and Depression in Patients with Pancreatobiliary Cancer: A Randomized Clinical Trial. Cancers (Basel). 2019;11(1):79. Published 2019 Jan 11. doi:10.3390/cancers11010079

9. do Carmo TM, Paiva BSR, de Oliveira CZ, Nascimento MSA, Paiva CE. The feasibility and benefit of a brief psychosocial intervention in addition to early palliative care in patients with advanced cancer to reduce depressive symptoms: a pilot randomized controlled clinical trial. BMC Cancer. 2017;17(1):564. Published 2017 Aug 23. doi:10.1186/s12885-017-3560-6

10. Brännström M, Boman K. Effects of person-centred and integrated chronic heart failure and palliative home care. PREFER: a randomized controlled study. *Eur J Heart Fail*. 2014;16(10):1142-1151. doi:10.1002/ejhf.151

11. Bakitas M, Lyons KD, Hegel MT, et al. Effects of a palliative care intervention on clinical outcomes in patients with advanced cancer: the Project ENABLE II randomized controlled trial. JAMA. 2009;302(7):741-749. doi:10.1001/jama.2009.1198

12. Benthien K, Diasso P, von Heymann A, et al. Oncology to specialised palliative home care systematic transition: the Domus randomised trial. BMJ Support Palliat Care. 2020;10(3):350-357. doi:10.1136/bmjspcare-2020-002325

13. El-Jawahri A, LeBlanc T, VanDusen H, et al. Effect of Inpatient Palliative Care on Quality of Life 2 Weeks After Hematopoietic Stem Cell Transplantation: A Randomized Clinical Trial. JAMA. 2016;316(20):2094-2103. doi:10.1001/jama.2016.16786

14. El-Jawahri A, LeBlanc TW, Kavanaugh A, et al. Effectiveness of Integrated Palliative and Oncology Care for Patients With Acute Myeloid Leukemia: A Randomized Clinical Trial. JAMA Oncol. 2021;7(2):238-245. doi:10.1001/jamaoncol.2020.6343

15. Hoek PD, Schers HJ, Bronkhorst EM, Vissers KCP, Hasselaar JGJ. The effect of weekly specialist palliative care teleconsultations in patients with advanced cancer -a randomized clinical trial. BMC Med. 2017;15(1):119. Published 2017 Jun 19. doi:10.1186/s12916-017-0866-9

16. Sidebottom AC, Jorgenson A, Richards H, Kirven J, Sillah A. Inpatient palliative care for patients with acute heart failure: outcomes from a randomized trial. J Palliat Med. 2015;18(2):134-142. doi:10.1089/jpm.2014.0192

17. Zimmermann C, Swami N, Krzyzanowska M, et al. Early palliative care for patients with advanced cancer: a cluster-randomised controlled trial. Lancet. 2014;383(9930):1721-1730. doi:10.1016/S0140-6736(13)62416-2

18. Kluger BM, Miyasaki J, Katz M, et al. Comparison of Integrated Outpatient Palliative Care With Standard Care in Patients With Parkinson Disease and Related Disorders: A Randomized Clinical Trial. JAMA Neurol. 2020;77(5):551-560. doi:10.1001/jamaneurol.2019.4992

19. Bakitas MA, Tosteson TD, Li Z, et al. Early Versus Delayed Initiation of Concurrent Palliative Oncology Care: Patient Outcomes in the ENABLE III Randomized Controlled Trial. J Clin Oncol. 2015;33(13):1438-1445. doi:10.1200/JCO.2014.58.6362

20. Patil VM, Singhai P, Noronha V, et al. Effect of Early Palliative Care on Quality of Life of Advanced Head and Neck Cancer Patients: A Phase III Trial. J Natl Cancer Inst. 2021;113(9):1228-1237. doi:10.1093/jnci/djab020

21. Greer JA, Moy B, El-Jawahri A, et al. Randomized Trial of a Palliative Care Intervention to Improve End-of-Life Care Discussions in Patients With Metastatic Breast Cancer. J Natl Compr Canc Netw. 2022;20(2):136-143. doi:10.6004/jnccn.2021.7040

22. Eychmüller S, Zwahlen S, Fliedner MC, et al. Single early palliative care intervention added to usual oncology care for patients with advanced cancer: A randomized controlled trial (SENS Trial). Palliat Med. 2021;35(6):1108-1117. doi:10.1177/02692163211005340

23. Franciosi V, Maglietta G, Degli Esposti C, et al. Early palliative care and quality of life of advanced cancer patients-a multicenter randomized clinical trial. Ann Palliat Med. 2019;8(4):381-389. doi:10.21037/apm.2019.02.07

24. Scarpi E, Dall'Agata M, Zagonel V, et al. Systematic vs. on-demand early palliative care in gastric cancer patients: a randomized clinical trial assessing patient and healthcare service outcomes. Support Care Cancer. 2019;27(7):2425-2434. doi:10.1007/s00520-018-4517-2

25. Temel JS, Sloan J, Zemla T, et al. Multisite, Randomized Trial of Early Integrated Palliative and Oncology Care in Patients with Advanced Lung and Gastrointestinal Cancer: Alliance A221303. J Palliat Med. 2020;23(7):922-929. doi:10.1089/jpm.2019.0377

26. Bekelman D, Baron A, Paden G, et al. Effect of a collaborative palliative care intervention vs usual care on quality of life of patients with symptomatic heart and lung diseases: a randomized clinical trial (CO202A). J Pain Symptom Manage. 2022;63(Suppl):1063.

27. Maltoni M, Scarpi E, Dall'Agata M, et al. Systematic versus on-demand early palliative care: results from a multicentre, randomised clinical trial. Eur J Cancer. 2016;65:61-68. doi:10.1016/j.ejca.2016.06.007

28. Temel JS, Greer JA, Muzikansky A, et al. Early palliative care for patients with metastatic non-small-cell lung cancer. N Engl J Med. 2010;363(8):733-742. doi:10.1056/NEJMoa1000678

29. Gao W, Wilson R, Hepgul N, et al. Effect of Short-term Integrated Palliative Care on Patient-Reported Outcomes Among Patients Severely Affected With Long-term Neurological Conditions: A Randomized Clinical Trial. JAMA Netw Open. 2020;3(8):e2015061. Published 2020 Aug 3. doi:10.1001/jamanetworkopen.2020.15061

30. Evans CJ, Bone AE, Yi D, et al. Community-based short-term integrated palliative and supportive care reduces symptom distress for older people with chronic noncancer conditions compared with usual care: A randomised controlled single-blind mixed method trial. Int J Nurs Stud. 2021;120:103978. doi:10.1016/j.ijnurstu.2021.103978

31. Bakitas MA, Dionne-Odom JN, Ejem DB, et al. Effect of an Early Palliative Care Telehealth Intervention vs Usual Care on Patients With Heart Failure: The ENABLE CHF-PC Randomized Clinical Trial. JAMA Intern Med. 2020;180(9):1203-1213. doi:10.1001/jamainternmed.2020.2861

32. Bekelman DB, Allen LA, McBryde CF, et al. Effect of a Collaborative Care Intervention vs Usual Care on Health Status of Patients With Chronic Heart Failure: The CASA Randomized Clinical Trial. JAMA Intern Med. 2018;178(4):511-519. doi:10.1001/jamainternmed.2017.8667

33. Rogers JG, Patel CB, Mentz RJ, et al. Palliative Care in Heart Failure: The PAL-HF Randomized, Controlled Clinical Trial. J Am Coll Cardiol. 2017;70(3):331-341. doi:10.1016/j.jacc.2017.05.030

34. Bassi I, Guerrieri A, Carpano M, et al. Feasibility and efficacy of a multidisciplinary palliative approach in patients with advanced interstitial lung disease. A pilot randomised controlled trial. Pulmonology. 2023;29(Suppl 4):S54-S62. doi:10.1016/j.pulmoe.2021.11.004

35. Tattersall MH, Martin A, Devine R, et al. Early contact with palliative care services: a randomized trial in patients with newly detected incurable metastatic cancer. *J Palliat Care Med.* 2014;4(1):1. doi:10.4172/2165-7386.1000170

36. Goldstein N, Mather H, DeCherrie L, et al. A randomized controlled trial of a novel home-based palliative care team centered around community health workers improved patient outcomes (RP524). J Pain Symptom Manage. 2022;63(Suppl):1110–1111.

37. Edmonds P, Hart S, Wei Gao, et al. Palliative care for people severely affected by multiple sclerosis: evaluation of a novel palliative care service. Mult Scler. 2010;16(5):627-636. doi:10.1177/1352458510364632

38. Aiken LS, Butner J, Lockhart CA, Volk-Craft BE, Hamilton G, Williams FG. Outcome evaluation of a randomized trial of the PhoenixCare intervention: program of case management and coordinated care for the seriously chronically ill. J Palliat Med. 2006;9(1):111-126. doi:10.1089/jpm.2006.9.111

39. Given B, Given CW, McCorkle R, et al. Pain and fatigue management: results of a nursing randomized clinical trial. Oncol Nurs Forum. 2002;29(6):949-956. doi:10.1188/02.ONF.949-956
