# Supplementary material for: Benefits of specialist palliative care by identifying active ingredients of service composition, structure, and delivery model: A systematic review with meta-analysis and meta-regression
Source: PLoS Med. 2024 Aug 2;21(8):e1004436. doi: 10.1371/journal.pmed.1004436 (PMC11329153; doi:10.1371/journal.pmed.1004436)
Supplement: S6 Appendix — (DOCX) [file pmed.1004436.s006.docx]

**Benefits of specialist palliative care by identifying active ingredients of service composition, structure, and delivery model: A systematic review with meta-analysis and meta-regression**

**S6 Appendix**

Miriam J. Johnson, Leah Rutherford, Anisha Sunny, Sophie Pask, Susanne de Wolf-Linder, Fliss E. M. Murtagh, Christina Ramsenthaler

[hycr22@hyms.ac.uk](mailto:hycr22@hyms.ac.uk)

# Excluded full-texts with reasons for exclusion

The main reasons for excluding full-texts were not fulfilling the study design of a phase III trial that also presented an adequate a priori sample size calculation or a priori demonstration of statistical power. Many full-texts had to be excluded due to the study not reporting the patient-centered outcome measures of interest to this review (patient health-related quality of life/symptom burden or emotional well-being). One study was excluded due to it presenting specialist palliative care in a study population of HIV/AIDS. Often, we excluded full-texts due to them not reporting a complex specialist palliative care intervention. Often, these were single-component intervention like advance care planning or psychosocial support only, lacking a multi-component, complex and multi-disciplinary approach.

Rather than listing all excluded studies, we detail below why studies previously identified as eligible in systematic reviews and meta-analyses (see S2 Appendix) that were not included in this study. The full list of excluded references can be obtained from the first author of this work.

## Pilot or feasibility study and/or no sample size calculation

1. Bajwah S, Ross JR, Wells AU, *et al*. Palliative care for patients with advanced fibrotic lung disease: a randomised controlled phase II and feasibility trial of a community case conference intervention. *Thorax.* 2015;70(9):830–839.
2. Bonsignore L, Bloom N, Steinhauser K, et al. Evaluating the feasibility and acceptability of a telehealth program in a rural palliative care population: TapCloud for palliative care. *J Pain Symptom Manage.* 2018;56(1):7–14.
3. Cheung W, Aggarwal G, Fugaccia E, *et al*. Palliative care teams in the intensive care unit: a randomised, controlled, feasibility study. *Critical Care Resusc.* 2010;12(1):28–35.
4. Dyar S, Lesperance M, Shannon R, Sloan J, Colon-Otero G. A nurse practitioner directed intervention improves the quality of life of patients with metastatic cancer: results of a randomized pilot study. *J Palliat Med.* 2012;15(8):890–895.
5. Fischer S, Golub M, Plata A, *et al*. Integrating palliative care social workers into sub-acute settings: feasibility of the ALIGN Intervention Trial (S825). *J Pain Symptom Manage.* 2019;57(2):495.
6. Grudzen CR, Richardson LD, Johnson PN, *et al*. Emergency department-initiated palliative care in advanced cancer: a randomised clinical trial. *JAMA Oncol.* 2016;2(5):591–598.
7. Hanson LC, Kistler CE, Lavin K, *et al*. Triggered palliative care for late-stage dementia: a pilot randomized trial. *J Pain Symptom Manage.* 2019;57:10–19.
8. Higginson IJ, McCrone P, Hart SR, Burman R, Silber E, Edmonds PM. Is short-term palliative care cost-effective in multiple sclerosis? A randomized phase II trial. *J Pain Symptom Manage.* 2009;38(6):816–826.
9. Janssens JP, Weber C, Herrmann FR, *et al*. Can Early Introduction of Palliative Care Limit Intensive Care, Emergency and Hospital Admissions in Patients with Severe Chronic Obstructive Pulmonary Disease? A Pilot Randomized Study. *Respiration.* 2019;97(5):406–415.
10. Jingfen RA, Tong ZH, Yanling RE, Yanli LI, Cuimin ZH. Influence of palliative care based on knowledge-belief-action model on the cancer-related fatigue and quality of life for patients with advanced lung cancer. *Anti-Tumor Pharm.* 2017;7(1):124–128.
11. Kimbell B, Murray SA, Byrne H, *et al*. Palliative care for people with advanced liver disease: A feasibility trial of a supportive care liver nurse specialist. *Palliat Med.* 2018;32(5):919–929.
12. Matsumoto Y. Early specialised palliative care in Japan: a feasibility study. *Ann Oncol.* 2016;27(Suppl 7):mdw466.
13. O’Donnell AE, Schaefer KG, Stevenson LW, *et al*. Social worker-aided palliative care intervention in high-risk patients with heart failure (SWAP-HF): a pilot randomized clinical trial. *JAMA Cardiol.* 2018;3(6):516–519.
14. O'Riordan D, Rathfon M, Joseph D, *et al*. Feasibility of implementing a palliative care intervention for people with heart failure: learnings from a pilot randomised clinical trial. *J Palliat Med.* 2019;22(12):1583–1588.
15. Pantilat SZ, O'Riordan DL, Dibble SL, Landefeld CS. Hospital-based palliative medicine consultation: a randomized controlled trial. *Arch Intern Med.* 2010;170(22):2038–2040.
16. Rodin G, Malfitano C, Rydall A, *et al*. Emotion And Symptom-focused Engagement (EASE): a randomised phase II trial of an integrated psychological and palliative care intervention for patients with acute leukemia. *Support Care Cancer.* 2019;28:163–176.
17. Sampson EL, Jones L, Thune-Boyle IC, *et al*. Palliative assessment and advance care planning in severe dementia: An exploratory randomized controlled trial of a complex intervention. *Palliat Med.* 2011;25:197–209.
18. Schenker Y, Bahary N, Claxton R, *et al*. A Pilot Trial of Early Specialty Palliative Care for Patients with Advanced Pancreatic Cancer: Challenges Encountered and Lessons Learned. *J Palliat Med.* 2018;21(1):28–36.
19. Shinall MC Jr, Karlekar M, Martin S, *et al*. COMPASS: A Pilot Trial of an Early Palliative Care Intervention for Patients With End-Stage Liver Disease. *J Pain Symptom Manage.* 2019;58(4):614–622.e3.
20. Solari A, Giordano A, Patti F, *et al*. Randomized controlled trial of a home-based palliative approach for people with severe multiple sclerosis. *Mult Scler.* 2018;24(5):663–674.
21. Spatuzzi R, Giulietti MV, Ricciuti M, *et al*. Quality of life and burden in family caregivers of patients with advanced cancer in active treatment settings and hospice care: A comparative study. *Death Stud.* 2017;41(5):276–283.
22. Treasure M, Daly B, Cao S, *et al*. A randomized controlled trial of structured palliative care versus standard supportive care for patients enrolled in phase 1 clinical trials. *Cancer Med.* 2021;10(13):4312–4321.
23. Véron C, Pautex S, Weber C, Janssens JP, Cedraschi C. Recollection of participating in a trial: A qualitative study of patients with severe and very severe chronic obstructive pulmonary disease. *PLoS One.* 2018;13(9):e0204701.
24. Veronese S, Gallo G, Valle A, *et al*. Specialist palliative care improves the quality of life in advanced neurodegenerative disorders: NE-PAL, a pilot randomised controlled study. *BMJ Support Palliat Care.* 2017;7(2):164–172.
25. Wallen GR, Baker K, Stolar M, *et al*. Palliative care outcomes in surgical oncology patients with advanced malignancies: a mixed methods approach. *Qual Life Res.* 2012;21(3):405–415.
26. Yang GM, Teo I, Neo SH, Tan D, Cheung YB. Pilot Randomized Phase II Trial of the Enhancing Quality of Life in Patients (EQUIP) Intervention for Patients With Advanced Lung Cancer. *Am J Hosp Palliat Care.* 2018;35(8):1050–1056.

## No patient-centered outcome measures (quality of life, emotional wellbeing) reported

1. Ahronheim JC, Morrison RS, Morris J, Baskin S, Meier DE. Palliative care in advanced dementia. *J Palliat Med.* 2000;3(3):265–273.
2. Brumley R, Enguidanos S, Jamison P, *et al*. Increased satisfaction with care and lower costs: results of a randomized trial of in-home palliative care. *J Am Geriatr Soc.* 2007;55(7):993–1000.
3. Casarett D, Karlawish J, Morales K, Crowley R, Mirsch T, Asch DA. Improving the use of hospice services in nursing homes. *JAMA.* 2005;294(2):211–217.
4. Chapman DG, Toseland RW. Effectiveness of advanced illness care teams for nursing home residents with dementia. *Soc Work.* 2007;52(4):321–329.
5. Farquhar MC, Prevost AT, McCrone P, *et al*. Is a specialist breathlessness service more effective and cost-effective for patients with advanced cancer and their carers than standard care? Findings of a mixed-method randomised controlled trial. *BMC Med.* 2014;12:194.
6. Farquhar MC, Prevost AT, McCrone P, *et al*. The clinical and cost effectiveness of a breathlessness intervention service for patients with advanced non-malignant disease and their informal carers: mixed findings of a mixed method randomised controlled trial. *Trials.* 2016;17(1):185.
7. Gade G, Venohr I, Conner D, *et al*. Impact of an inpatient palliative care team: a randomized control trial. *J Palliat Med.* 2008;11(2):180–190.
8. Grande GE, Todd CJ, Barclay SI, Farquhar MC. A randomized controlled trial of a hospital at home service for the terminally ill. *Palliat Med.* 2000;14(5):375–385.
9. Hopp FP, Zalenski RJ, Waselewsky D, *et al*. Results of a hospital-based palliative care intervention for patients with an acute exacerbation of chronic heart failure. *J Card Fail.* 2016;22:1033–1036.
10. Husebo BS, Ballard C, Aarsland D, *et al*. The effect of a multicomponent intervention on quality of life in residents of nursing homes: a randomized controlled trial (COSMOS). *J Am Med Dir Assoc.* 2019;20:330–339.
11. Jordhøy MS, Fayers P, Saltnes T, Ahlner-Elmqvist M, Jannert M, Kaasa S. A palliative-care intervention and death at home: a cluster randomised trial. *Lancet.* 2000;356(9233):888–893.
12. Ma J, Chi S, Buettner B, *et al*. Early Palliative Care Consultation in the Medical ICU: A Cluster Randomized Crossover Trial. *Crit Care Med.* 2019;47(12):1707–1715.
13. McCaffrey N, Agar M, Harlum J, Karnon J, Currow D, Eckermann S. Is home-based palliative care cost-effective? An economic evaluation of the Palliative Care Extended Packages at Home (PEACH) pilot. *BMJ Support Palliat Care*. 2013;3(4):431–435.
14. O’Hara RE, Hull JG, Lyons KD, *et al*. Impact on caregiver burden of a patient-focused palliative care intervention for patients with advanced cancer. *Palliat Support Care.* 2010;8(4):395–404.
15. Sahlen K-G, Boman K, Brännström M. A cost-effectiveness study of person-centered integrated heart failure and palliative home care: based on a randomized controlled trial. *Palliat Med.* 2016;30:296–302.
16. Van Den Block L, Honinx E, Pivodic L, *et al*. Evaluation of a palliative care program for nursing homes in 7 countries: The PACE cluster-randomized clinical trial. *JAMA Intern Med.* 2020;180:233–242.
17. Walsh K, Jones L, Tookman A, *et al*. Reducing emotional distress in people caring for patients receiving specialist palliative care. Randomised trial. *Br J Psychiatr.* 2007;190:142–147.

## Study in HIV/AIDS (excluded patient group)

1. Lowther K, Simms V, Selman L, *et al*. Treatment outcomes in palliative care: the TOPCare study. A mixed methods phase III randomised controlled trial to assess the effectiveness of a nurse-led palliative care intervention for HIV positive patients on antiretroviral therapy. *BMC Infect Dis.* 2012;12:288.

## Study not a phase III RCT controlled trial

1. Ahlner-Elmqvist M, Jordhøy MS, Bjordal K, Jannert M, Kaasa S. Characteristics and quality of life of patients who choose home care at the end of life. *J Pain Symptom Manage.* 2008;36(3):217–227.
2. Bužgová R, Kozáková R, Bar M. Satisfaction of Patients With Severe Multiple Sclerosis and Their Family Members With Palliative Care: Interventional Study. *Am J Hosp Palliat Care.* 2021;38(11):1348–1355.
3. Bužgová R, Kozáková R, Bar M. The effect of neuropalliative care on quality of life and satisfaction with quality of care in patients with progressive neurological disease and their family caregivers: an interventional control study. *BMC Palliat Care.* 2020;19:143.
4. Daly BJ, Douglas SL, Gunzler D, Lipson AR. Clinical trial of a supportive care team for patients with advanced cancer. *J Pain Symptom Manage.* 2013;46(6):775–784.
5. de Lusignan S, Wells S, Johnson P, *et al*. Compliance and effectiveness of 1 year’s home telemonitoring. The report of a pilot study of patients with chronic heart failure. *Eur J Heart Fail.* 2001;3:723–730.
6. Evangelista LS, Lombardo D, Malik S, *et al*. Examining the effects of an outpatient palliative care consultation on symptom burden, depression, and quality of life in patients with symptomatic heart failure. *J Card Fail.* 2012;18:894–899.
7. Gómez-Batiste X, Porta-Sales J, Espinosa-Rojas J, Pascual- Lopez A, Tuca A, Rodriguez J. Effectiveness of palliative care services in symptom control of patients with advanced terminal cancer: a Spanish, multicenter, prospective, quasiexperimental, prepost study. *J Pain Symptom Manage.* 2010;40(5):652–660.
8. Hanson LC, Reynolds KS, Henderson M, Pickard CG. A quality improvement intervention to increase palliative care in nursing homes. *J Palliat Med.* 2005;8(3):576–584.
9. O'Mahony S, Johnson TJ, Amer S, *et al*. Integration of Palliative Care Advanced Practice Nurses Into Intensive Care Unit Teams. *Am J Hosp Palliat Care.* 2017;34(4):330–334.
10. Ozcelik H, Fadiloglu C, Karabulut B, Uyar M. Examining the effect of the case management model on patient results in the palliative care of patients with cancer. *Am J Hosp Palliat Care.* 2014;31(6):655–664.
11. Ringdal GI, Ringdal K, Jordhøy MS, Ahlner-Elmqvist M, Jannert M, Kaasa S. Health-related quality of life (HRQOL) in family members of cancer victims: results from a longitudinal intervention study in Norway and Sweden. *Palliat Med.* 2004; **18**(2): 108–20.
12. Schwartz CE, Wheeler HB, Hammes B, *et al*. Early intervention in planning end-of-life care with ambulatory geriatric patients: results of a pilot trial. *Arch Intern Med.* 2002; **162**(14): 1611–8.
13. Ullrich A, Ascherfeld L, Marx G, Bokemeyer C, Bergelt C, Oechsle K. Quality of life, psychological burden, needs, and satisfaction during specialized inpatient palliative care in family caregivers of advanced cancer patients. *BMC Palliat Care.* 2017; **16**(1): 31.

## Intervention is not specialist palliative care intervention (or not multi-component)

1. Agar M, Luckett T, Luscombe G, *et al*. Effects of facilitated family case conferencing for advanced dementia: A cluster randomised clinical trial. *PLoS One.* 2017;12:e0181020.
2. Austin J, Williams R, Ross L, *et al*. Randomised controlled trial of cardiac rehabilitation in elderly patients with heart failure. *Eur J Heart Fail.* 2005;7:411–417.
3. Berglund K, Chai E, Moreno J, Reyna MA, Gelfman L. Development of a social work-led primary palliative care model in hospital medicine (FR481C). *J Pain Symptom Manage.* 2019;57(2):436.
4. Carson SS, Cox CE, Wallenstein S, *et al*. Effect of palliative care-led meetings for families of patients with chronic critical illness: a randomised controlled trial. *JAMA.* 2016;316(1):51–62.
5. Chang B-H, Hendricks A, Zhao Y, *et al*. A relaxation response randomized trial on patients with chronic heart failure. *J Cardiopulm Rehabil Prevent.* 2005;25:149–157.
6. Chochinov HM, Kristjanson LJ, Breitbart W, *et al*. Effect of dignity therapy on distress and end-of life experience in terminally ill patients: A randomised controlled trial. *Lancet Oncol.* 2011;12:753–762.
7. Clark MM, Rummans TA, Atherton PJ, *et al*. Randomized controlled trial of maintaining quality of life during radiation therapy for advanced cancer. *Cancer.* 2015;119(4):880–887.
8. Cornbleet MA, Campbell P, Murray S, Stevenson M, Bond S; Joint Working Party of the Scottish Partnership Agency for Palliative and Cancer Care and National Council for Hospice and Specialist Palliative Care Services. Patient-held records in cancer and palliative care: a randomized, prospective trial. *Palliat Med.* 2002;16(3):205–212.
9. Detmar SB, Muller MJ, Schornagel JH, Wever LD, Aaronson NK. Health-related quality-of-life assessments and patient-physician communication: a randomized controlled trial. *JAMA.* 2002;288(23):3027–3034.
10. Ditto PH, Danks JH, Smucker WD, *et al*. Advance directives as acts of communication: a randomized controlled trial. *Arch Intern Med.* 2001;161(3):421–430.
11. Doughty R, Wright S, Pearl A, *et al*. Randomized, controlled trial of integrated heart failure management. The Auckland Heart Failure Management Study. *Eur Heart J.* 2002;23:139–146.
12. Dunbar SB, Reilly CM, Gary R, *et al*. Randomized clinical trial of an integrated self-care intervention for persons with heart failure and diabetes: quality of life and physical functioning outcomes. *J Card Fail.* 2015;21:719–729.
13. Engelhardt JB, McClive-Reed KP, Toseland RW, *et al*. Effects of a program for coordinated care of advanced illness on patients, surrogates, and healthcare costs: a randomized trial. *Am J Manag Care.* 2006;12:93–100.
14. Ferrell B, Chung V, Hughes MT, *et al*. A Palliative Care Intervention for Patients on Phase 1 Studies. *J Palliat Med.* 2021;24(6):846–856.
15. Gary RA, Dunbar SB, Higgins MK, *et al*. Combined exercise and cognitive behavioral therapy improves outcomes in patients with heart failure*. J Psychosom Res.* 2010;69:119–131.
16. Goldberg LR, Piette JD, Walsh MN, *et al*. Randomized trial of a daily electronic home monitoring system in patients with advanced heart failure: the Weight Monitoring in Heart Failure (WHARF) trial. *Am Heart J.* 2003;146:705–712.
17. Harding R, Higginson IJ, Leam C, *et al*. Evaluation of a short-term group intervention for informal carers of patients attending a home palliative care service. *J Pain Symptom Manage.* 2004;27(5):396–408.
18. Harrison MB, Browne GB, Roberts J, *et al*. Quality of life of individuals with heart failure: a randomized trial of the effectiveness of two models of hospital-to-home transition. *Med Care.* 2002;40(2):271–282.
19. Higginson IJ, Bausewein C, Reilly CC, *et al*. An integrated palliative and respiratory care service for patients with advanced disease and refractory breathlessness: A randomised controlled trial. *Lancet Respir Med.* 2014;2:979–987.
20. Hudson PL, Aranda S, Hayman-White K. A psychoeducational intervention for family caregivers of patients receiving palliative care: a randomized controlled trial. *J Pain Symptom Manage.* 2005;30(4):329–341.
21. Hughes SL, Weaver FM, Giobbie-Hurder A, *et al*. Effectiveness of team-managed home-based primary care: a randomized multicenter trial. *JAMA*. 2000;284:2877–2885.
22. Jaarsma T, Halfens R, Tan F, *et al*. Self-care and quality of life in patients with advanced heart failure: the effect of a supportive educational intervention. *Heart Lung.* 2000;29:319–330.
23. Kasper EK, Gerstenblith G, Hefter G, *et al*. A randomized trial of the efficacy of multidisciplinary care in heart failure outpatients at high risk of hospital readmission. *J Am Coll Cardiol.* 2002;39:471–480.
24. Kissane DW, Grabsch B, Clarke DM, *et al*. Supportive-expressive group therapy: the transformation of existential ambivalence into creative living while enhancing adherence to anti-cancer therapies. *Psychooncology.* 2004;13(11):755–768.
25. Lang CC, Smith K, Wingham J, et al. A randomised controlled trial of a facilitated home-based rehabilitation intervention in patients with heart failure with preserved ejection fraction and their caregivers: the REACH-HFpEF Pilot Study. *BMJ Open.* 2018;8(4):e019649.
26. Lu Z, Fang Y, Liu C, *et al*. Early Interdisciplinary Supportive Care in Patients With Previously Untreated Metastatic Esophagogastric Cancer: A Phase III Randomized Controlled Trial. *J Clin Oncol.* 2021;39(7):748–756.
27. Luskin F, Reitz M, Newell K, *et al*. A controlled pilot study of stress management training of elderly patients with congestive heart failure. *Prevent Cardiol.* 2002;5:168–174.
28. McCorkle R, Jeon S, Ercolano E, *et al*. An advanced practice nurse coordinated multidisciplinary intervention for patients with late-stage cancer: a cluster randomized trial. *J Palliat Med.* 2015;18(11):962–969.
29. McMillan SC, Small BJ, Weitzner M, *et al*. Impact of coping skills intervention with family caregivers of hospice patients with cancer: a randomized clinical trial. *Cancer.* 2006;106:214–222.
30. McMillan SC, Small BJ. Using the COPE intervention for family caregivers to improve symptoms of hospice homecare patients: a clinical trial. *Oncol Nurs Forum.* 2007;34:313–321.
31. Meyers FJ, Carducci M, Loscalzo MJ, *et al*. Effects of a problem-solving intervention (COPE) on quality of life for patients with advanced cancer on clinical trials and their caregivers: simultaneous care educational intervention (SCEI): linking palliation and clinical trials. *J Palliat Med.* 2011;14:465–473.
32. Miller DK, Chibnall JT, Videen SD, *et al*. Supportive-affective group experience for persons with life-threatening illness: reducing spiritual, psychological, and death-related distress in dying patients. *J Palliat Med.* 2005;8:333–343.
33. Mitchell GK, Del Mar CB, O'Rourke PK, Clavarino AM. Do case conferences between general practitioners and specialist palliative care services improve quality of life? A randomised controlled trial. *Palliat Med.* 2008;22(8):904–912.
34. Moore S, Corner J, Haviland J, *et al*. Nurse-led follow-up and conventional medical follow up in management of patients with lung cancer. *BMJ.* 2002; 325(7373):1145.
35. Naylor MD, Brooten DA, Campbell RL, *et al*. Transitional care of older adults hospitalized with heart failure: a randomized, controlled trial. *J Am Geriatr Soc.* 2004;52:675–684.
36. Nipp RD, Horick NK, Qian CL, *et al*. Effect of a Symptom Monitoring Intervention for Patients Hospitalized With Advanced Cancer: A Randomized Clinical Trial. *JAMA Oncol.* 2022;8(4):571–578.
37. Northouse L, Kershaw T, Mood D, Schafenacker A. Effects of a family intervention on the quality of life of women with recurrent breast cancer and their family caregivers. *Psychooncology.* 2005;14(6):478–491.
38. Northouse LL, Mood DW, Schafenacker A, *et al*. Randomized clinical trial of a family intervention for prostate cancer patients and their spouses. *Cancer.* 2007;110(12):2809–2818.
39. Northouse LL, Mood DW, Schafenacker A, *et al*. Randomized clinical trial of a brief and extensive dyadic intervention for advanced cancer patients and their family caregivers. *Psychooncology.* 2013;22(3):555–563.
40. Possin KL, Merrilees JJ, Dulaney S, *et al*. Effect of collaborative dementia care via telephone and internet on quality of life, caregiver well-being, and health care use: the care ecosystem randomized clinical trial. *JAMA Intern Med.* 2019; 179:1658–1667.
41. Rabow MW, Dibble SL, Pantilat SZ, McPhee SJ. The comprehensive care team: a controlled trial of outpatient palliative medicine consultation. *Arch Intern Med.* 2004;164(1):83–91.
42. Radwany SM, Hazelett SE, Allen KR, *et al*. Results of the Promoting Effective Advance Care Planning for Elders (PEACE) randomized pilot study. *Popul Health Manag.* 2014;17(2):106–111.
43. Raftery JP, Addington-Hall JM, MacDonald LD, *et al*. A randomized controlled trial of the cost-effectiveness of a district co-ordinating service for terminally ill cancer patients. *Palliat Med.* 1996;10:151–161.
44. Riegel B, Carlson B, Glaser D, *et al*. Randomized controlled trial of telephone case management in Hispanics of Mexican origin with heart failure. *J Cardiac Fail.* 2006;12:211–219.
45. Rummans TA, Clark MM, Sloan JA, *et al*. Impacting quality of life for patients with advanced cancer with a structured multidisciplinary intervention: a randomized controlled trial. *J Clin Oncol.* 2006;24(4):635–642.
46. Schenker Y, Althouse AD, Rosenzweig M, *et al*. Effect of an Oncology Nurse-Led Primary Palliative Care Intervention on Patients With Advanced Cancer: The CONNECT Cluster Randomized Clinical Trial. *JAMA Intern Med.* 2021;181(11):1451–1460.
47. Schunk M, Le L, Syunyaeva Z, *et al*. Effectiveness of a specialised breathlessness service for patients with advanced disease in Germany: a pragmatic fast-track randomised controlled trial (BreathEase). *Eur Respir J.* 2021;58(2):2002139.
48. Sherwood A, Blumenthal JA, Koch GG, *et al*. Effects of coping skills training on quality of life, disease biomarkers, and clinical outcomes in patients with heart failure: a randomized clinical trial. *Circ Heart Fail.* 2017;10:1.
49. Steel JL, Geller DA, Kim KH, *et al*. Web-based collaborative care intervention to manage cancer-related symptoms in the palliative care setting. *Cancer.* 2016;122(8):1270–1282.
50. Steinhauser KE, Alexander S, Olsen MK, *et al*. Addressing Patient Emotional and Existential Needs During Serious Illness: Results of the Outlook Randomized Controlled Trial. *J Pain Symptom Manage.* 2017;54(6):898–908.
51. Sussman J, Bainbridge D, Whelan TJ, *et al*. Evaluation of a specialised oncology nursing supportive care intervention in newly diagnosed breast and colorectal cancer patients following surgery: a cluster randomised trial. *Support Care Cancer.* 2018;26(5):1533–1541.
52. Tsianakas V, Harris J, Ream E, *et al*. CanWalk: a feasibility study with embedded randomised controlled trial pilot of a walking intervention for people with recurrent or metastatic cancer. *BMJ Open.* 2017;7(2):e013719.
53. Uitdehaag MJ, van Putten PG, van Eijck CH, *et al*. Nurse-led follow-up at home vs. conventional medical outpatient clinic follow-up in patients with incurable upper gastrointestinal cancer: A randomized study. *J Pain Symptom Manage.* 2014;47:518–530.
54. van Boxell P, Anderson K, Regnard C. The effectiveness of palliative care education delivered by videoconferencing compared with face-to-face delivery. *Palliat Med.* 2003;17(4):344–358.
55. Van Spall HGC, Lee SF, Xie F, *et al*. Effect of Patient-Centered Transitional Care Services on Clinical Outcomes in Patients Hospitalized for Heart Failure: The PACT-HF Randomized Clinical Trial. *JAMA.* 2019;321(8):753–761.
56. Wang TC, Huang JL, Ho WC, *et al*. Effects of a supportive educational nursing care programme on fatigue and quality of life in patients with heart failure: a randomised controlled trial. *Eur J Cardiovasc Nurs.* 2016;15:157–167.
57. Young JM, Butow PN, Walsh J, *et al*. Multicenter randomized trial of centralized nurse-led telephone-based care coordination to improve outcomes after surgical resection for colorectal cancer: the CONNECT intervention. *J Clin Oncol.* 2013;31(28):3585–3591.
58. Yu DS, Lee DT, Woo J. Improving health-related quality of life of patients with chronic heart failure: effects of relaxation therapy. *J Adv Nurs.* 2010;66:392–403.
