# Supplementary material for: Benefits of specialist palliative care by identifying active ingredients of service composition, structure, and delivery model: A systematic review with meta-analysis and meta-regression
Source: PLoS Med. 2024 Aug 2;21(8):e1004436. doi: 10.1371/journal.pmed.1004436 (PMC11329153; doi:10.1371/journal.pmed.1004436)
Supplement: S7 Appendix — (DOCX) [file pmed.1004436.s007.docx]

**Benefits of specialist palliative care by identifying active ingredients of service composition, structure, and delivery model: A systematic review with meta-analysis and meta-regression**

**S7 Appendix**

Miriam J. Johnson, Leah Rutherford, Anisha Sunny, Sophie Pask, Susanne de Wolf-Linder, Fliss E. M. Murtagh, Christina Ramsenthaler

[hycr22@hyms.ac.uk](mailto:hycr22@hyms.ac.uk)

**Description of included studies**

## Table A. Description of study & population characteristics of included RCTs (k = 39)

| **Study and country*** | **Unit of allocation** | **Aim** | **Setting** | **# centres** | **Recruitment** | **Inclusion criteria** | **Exclusion criteria** | **Disease group** |
| --- | --- | --- | --- | --- | --- | --- | --- | --- |
| Aiken *et al* 2006^1^  United States of America | Patient | To test the hypotheses that the PhoenixCare participants, compared to control group would: achieve better self-management of illness, improve better preparedness for end of life, and enhanced physical and mental functioning , with lower utilization of costly emergency and inpatient services, specifically fewer emergency department visits and reduced length of stay in hospitals. | multiple settings | Mono | Patients could be referred for evaluation to the study by community agencies, hospitals, the MCOs, physicians, family/friends, or by self-referral to the MCO. Referrals were screened for eligility. | Advanced stage COPD, limited physical functioning, symptom exacerbation within 3 months prior to enrollment. Telephone at home available. | No telephone available at home, no understanding of English language or no translator available. | noncancer |
| Bakitas *et al* 2009^2^  United States of America | Patient | a) To test the hypotheses that soon after a new diagnosis of an advanced cancer would become informed, active participants in their care and would experience improved quality of life and mood, symptom relief, and lower resource use over the course of the illness, including at the very end of life compared with patients who received usual care. b) caregiver outcomes (eg, caregiver burden, perceptions of end-of-life care, and grief | multiple settings | Multi | Identification at the Cancer centre Tumor Board | New diagnosed advanced solid cancer, survival prognosis 1 year. | Impaired cognition, psychiatric disorder, active substance use | cancer |
| Bakitas *et al* 2015^3^  United States of America | Patient | To compare the effect of early (at diagnosis or recurrent terminal cancer) versus delayed intervention timing (12weeks) on patient-reported outcomes, 1-year survival, and resource use. | multiple settings | Multi | Research coordinators reviewed all outpatient clinicians’ schedules, tumor board lists, oncology clinic appointments, and inpatient admissions using eligibility criteria | New, progressive, or reoccuring advanced cancer, survival prognosis 1 year. | Impaired cognition, psychiatric disorder, substance disorder, hearing disorder, unreliable telephone service, prior involvement with palliative care service within the last year. | cancer |
| Bakitas *et al* 2020^4^  United States of America | Patient | To determine whether ENABLE CHF-PC leads to higher advanced heart failure patient-reported quality of life (QOL) and mood (depression/anxiety); and lower symptom burden and resource use (e.g. hospital admissions and days, emergency visits) through 16 weeks post baseline. | multiple settings | Multi | Recruited from cardiology, advanced HF, geriatric, and primary care clinics | Advanced stage heart failure, ≥65 years | Dementia or significant confusion, psychiatric disorder, uncorrectable hearing disorder. | noncancer |
| Bassi *et al* 2021^5^  Italy | Patient | To determine the feasibility and efficacy of a multidisciplinary approach to relieve patients’ symptoms compared to usual care. | hospital outpatient model | Mono | Fifty consecutive outpatients were enrolled at the ILD center. | Age over 18 years, evidence on a High-Resolution Chest CT of fibrosing interstitial disease with at least one of the following: traction bronchiectasis and honeycombing by CT scan, evidence of advanced disease, defined as the partial pressure of oxygen in arterial blood (PaO2) < 60 mmHg at room air, a decline in forced vital capacity (FVC) > 10% in the previous 6 months, or a GAP index of at least 3 | Diagnosis of active cancer, treatment with anti-fibrotic therapy, and ongoing palliative pharmacological treatments | noncancer |
| Bekelman *et al* 2018^6^  United States of America | Patient | To evaluate the effect of the CASA intervention on heart failure specific health status in a population of patients with poor health status. Secondary outcomes included common symptoms (fatigue, pain, shortness of breath, anxiety, and depression), hospitalizations, and mortality. | multiple settings | Multi | Patients with heart failure were identified through the study sites’ electronic health records, During the study screening process, patients who reported reduced heart failure–specific health status (a KCCQ score of ≤ 70) or reported at least 1 of the study’s target symptoms (fatigue, shortness of breath, pain, and/or depression) were targeted for enrolment. | Advanced stage heart failure, access to a telephone, primary care or other provider, low health status Presence of either pain, depression, fatigue, or breathlessness. | Dementia, substance abuse, metastatic cancer, nursing home resident, heart Transplant recipient, LVAD recipient | noncancer |
| Bekelman *et al* 2022^7^  United States of America | Patient | (a) To propose a primary palliative care approach to improve QOL in COPD and CHF, (b) to compare QOL outcomes from a randomized clinical trial of a primary palliative care intervention (ADAPT intervention) | hospital outpatient model | Multi | Population-based sampling methods will be used to enroll 300 Veterans with CHF, COPD, or interstitial lung disease who have poor quality of life and are at high risk for hospitalization or death. | Veterans enrolled in VA Eastern Colorado Health Care System or VA Puget - Sound Health Care System Diagnosis of CHF, pulmonary fibrosis, or COPD in 2 years prior to enrollment; High risk for hospitalization and death, Poor quality of life, Symptomatic, Primary care or other provider who is willing to facilitate intervention medical recommendations, Able to read and understand English, Consistent access to and able to use a standard telephone | Previous diagnosis of dementia, Active substance abuse, Comorbid metastatic cancer, Nursing home resident, Heart or lung transplant or left ventricular assist device (LVAD), Currently receiving hospice, palliative or home-based primary care, Currently pregnant, Currently a prisoner | noncancer |
| Benthien *et al* 2020^8^  Denmark | Patient | To assess the effect of the Domus trial intervention on symptom burden, to explore intervention mechanisms through patient and intervention provider characteristics and to assess long-term survival and place of death 30 months after study termination. | multiple settings | Mono | Bed wards were screened daily and outpatient wards were screened sequentially. | Incurable cancer with limited or no antineoplastic treatment options, want to spend as much time as possible at home supported by an SPC home care team, Living in the Captial Region | Already been referred to an SPC home care team; Inpatients with no planned discharge to home date. | cancer |
| Brännström *et al* 2014^9^  Sweden | Patient | To evaluate the effects of the PREFER intervention on symptom burden, quality of life (QOL), and functional classes compared with usual care. | hospital outreach model | Mono | Patients fullfilling eligibility criteria were asked to participate. | Advanced heart failure, need for frequent or continual i.v. support, chronically poor QOL, signs of cardiac cachexia, life expectancy of <1 year. | Severe communication problems; dementia or other serious diseases, treating Health Care Centre > 30 km from the hospital. | noncancer |
| Brims *et al* 2019^10^  Australia | Patient | To examine the effect of regular early SPC in combination with current standard care on HRQoL in patients with malignant pleural mesothelioma (MPM) and their carers compared with standard care alone. | hospital outreach model | Multi | Screening specialist thoracic cancer MDT meetings. Approached by Lung Cancer CNS and study team. | Histological or cytological confirmation of malignant pleural mesothelioma (MPM),  ECOG Performance Score of 0–1 (0 indicating the patient is asymptomatic, 1 indicating some symptoms but ambulatory), Diagnosis of MPM received within the last 6 weeks, Able to provide written informed consent in English, comply with trial procedures. | Another known malignancy within 5 years (excluding localised squamous cell carcinoma of the skin, cervical intraepithelial neoplasia, grade III and low-grade prostate cancer (Gleason score<5, with no metastases)), Significant morbidity; A symptom burden sufficient to require referral to SPC at the time of diagnosis, Concurrent or less than 3 months since participation in another clinical trial that may affect HRQoL; Surgery for MPM; Chemotherapy for MPM initiated prior to consent, significant history of psychiatric illness requiring specialist hospital care within the last 12 months. | cancer |
| Do Carmo *et al* 2017^11^  Brazil | Patient | To assess the feasibility and potential benefit of a brief psychosocial intervention based on cognitive-behavioral therapy performed in addition to early palliative care (PC) in the reduction of depressive symptoms among patients with advanced cancer. | inpatient consulting model | Mono | Screening in oncology outpatient clinic | ≥ 18 years and <75 years, knowledge about the cancer diagnosis, ECOG ≤ 2; Life expectancy> 6 months and <24 months, advanced solid cancer | Undergoing any psychological treatment, using antidepressants, cognitive deficit or attention problem, psychological conditions, mood disorders, Patients with single resected metastasis, any co-morbid condition, unable to go to the hospital. | cancer |
| Edmonds *et al* 2010^12^  United Kingdom | Patient | To explore whether a new palliative care service improved outcomes for people severely affected by multiple sclerosis | multiple settings | Mono | Identification during weekly multiprofessional MS clinic. Referral criteria: palliative care (pain and symptom control, complex psychosocial issues, EOL care planning) in addition to some specific triggers identified by the clinical team (hydration and nutrition, advance directives, competency and consent) | Patients would be able to benefit from SPC-Assessment, greater than 8 on the EDSS, referral on the basis of need. | No specific exclusion criteria | noncancer |
| El-Jahwari *et al* 2016^13^  United States of America | Patient | To assess the effect of inpatient palliative care integrated with standard transplant care on patient-reported QOL, mood, and symptom burden during hospitalization for HSCT and at 3 months after HSCT. | inpatient consulting model | Mono | Identification during the weekly transplant team meetings. Patients were approached within 72 hours of their transplant admission. | Hematologic malignancy admitted to Hospital for (HSCT). Adult caregivers of patients undergoing HSCT who agreed to participate in study. A relative or a friend, who either lives with the patient or has in-person contact with him or her at least twice per week. | Prior history of HSCT. Patients undergoing HSCT for a benign hematologic condition, Psychiatric disorder, co-morbid disease, enrolled in other supportive care intervention trials. | cancer |
| El-Jahwari *et al* 2021^14^  United States of America | Patient | To assess the effect of integrated palliative and oncology care (IPC) on patient-reported and EOL outcomes in patients with Acute Myeloid Leukemia (AML). | inpatient consulting model | Multi | We identified consecutive eligible hospitalized patients with AML by screening the hospital admission census at participating institutions. Patients were approached within 72 hours of initiating chemotherapy. | High-risk AML receiving intensive chemotherapy. | Acute promyelocytic leukemia, receiving nonintensive chemotherapy, already receiving palliative care, major psychiatric conditions, comorbid conditions. | cancer |
| Evans *et al* 2021^15^  United Kingdom | Patient | To evaluate the impact of the short-term integrated palliative and supportive care intervention for older people living with chronic noncancer conditions and frailty on clinical and economic outcomes and perceptions of care. | multiple settings | Multi | General practitioner identified people fullfilling eligibility criteria and referred them to the palliative care team. | Registerd with a GP, aged ≥75, with a Clinical Frailty Scale sore of ≥4, severely affected by non-malignant chronic conditions, with or without mental capacity. | Patients receiving specialist palliative care. | noncancer |
| Eychmüller *et al* 2021^16^  Switzerland | Patient | To compare a single structured early palliative care intervention added to a usual oncology care in terms of distress and health-related quality of life | hospital outpatient model | Multi | Screening of clinical admissions and tumor board meetings. Offering SPC for patients being discussed in tumor board meetings. Handing out leaflets to potential eligible patients. | New diagnosis of histologically confirmed advanced solid cancer, which is not amenable or not responsive to curative treatment. ECOG 0-2, | Cognitive impairment, previous contact with the specialized palliative care team. | cancer |
| Franciosi *et al* 2019^17^  Italy | Patient | To compare quality of life (QoL) of patients receiving early palliative care (EPC) vs. Standard oncologic care (SOC) | multiple settings | Multi | Identification by research personnel. (Not screening for palliative care needs.) | Newly diagnose, pathologically confirmed solid cancer, ECOG 1-2, metastatic or locally advanced disease, life expectancy > three months, eligibile for first-line chemotherapy and/or biological therapy. | Patients already receiving SPC or previously treated with chemotherapy and/or biological therapy, patients with NSCLC with EGFR mutation | cancer |
| Gao *et al* 2020^18^  United Kingdom | Patient | To determine the effectiveness of a SIPC intervention for people with long-term neurological conditions | multiple settings | Multi | Identified by a neurologist or a clinical nurse specialist and referred to the trial. | Severely affected by advanced or progressive stages of neurological illnesses, unresolved symptoms. | Already receiving specialist palliative care, lacking mental capacity, no caregiver | noncancer |
| Given *et al* 2002^19^  United States of America | Patient with carer | To test the effectiveness of a supportive nursing intervention on patients’ symptom management, the physical role impact, and social functioning. | multiple settings | Multi | Nurse recruiters specially trained for this study at each site identified patients (and their caregivers) who were eligible for the study, explained the study, and obtained signed informed consents for those willing to participate. | Initiating first cycle of chemotherapy following a new cancer diagnosis (solid tumors; non-Hodgkin lymphoma), ≥40 years; Report of pain and fatigue at baseline, caregivers willing to participate. | Cognitive impairment; Expected survival <32 weeks. | cancer |
| Goldstein *et al* 2022^20^  United States of America | Patient | To study the impact of a new home based palliative care program on patients' symptoms, quality of life, satisfaction with care, completion of advance care planning documentation and receipt of care consistent with preferences. In addition, the study will examine the impact of this model of care on patient healthcare utilization, including hospitalization, emergency department utilization, and hospice use prior to death. The trial will also include patients' caregivers, in order to examine the impact of the intervention on caregiver burden and prevalence of depression | home setting | Mono | Potential subjects will be identified from Mount Sinai records or referred by a Mount Sinai healthcare provider. Patients will only be approached after authorization by their Mount Sinai physician. Participants who consent to enrollment will be randomized to receive the intervention (home-based palliative care program) or usual care (with their nominated Mount Sinai physician). Patients will be enrolled in the study for a minimum of 6 months. | Presence of a "serious medical illness" - according to pre-specified diagnostic code-based criteria; Subject has a Mount Sinai physician who authorizes their participation in the study and receipt of the program; Subject is resident in Manhattan outside of a long term care facility and is not receiving hospice 5, Subject has evidence of capacity to benefit from enrollment in palliative care program, Subject is conversant in English or Spanish, Subject has capacity to consent | Subject has no usual physician within Mount Sinai, Subject's usual physician doesn't provide authorization to patient participation, Subject resident outside of Manhattan or in long term care facility or receiving hospice, Subject is not conversant in English or Spanish, Subject cannot provide informed consent to participation, Dementia (where the subject does not have the capacity to consent) | noncancer |
| Greer *et al* 2022^21^  United States of America | Patient | Primary aim: To determine the effect of a structured PC intervention on documentation of EOL care discussions in the electronic health record (EHR). Secondary aim: To assess the effect of the intervention on patient-reported discussions about EOL care preferences, QOL, and symptoms of anxiety and depression, as well as hospice utilization | inpatient consulting model | Mono | Screening at the breast oncolcogy clinic via research assistant. Research assistant sought permission to approach patients via treating oncologist. | Metastatic breast cancer with poor prognosis, ECOG ≤2. | Already receiving PC or needing immediate palliative or hospice care; mental illness/cognitive impairment. | cancer |
| Groenvold *et al* 2017^22^  Denmark | Patient | To investigate the effect of early SPC among advanced cancer patients | multiple settings | Multi | Screening of patients in oncological treatment or follow-up by research nurse. | Cancer stage IV or cancer in the central nervous system grade III/IV, lived in the area of one of the participating SPC centres, no contact with an SPC during the previous year; Presence of symptoms or declined functional and at least four additional symptoms measured with the EORTC-QLQ-C30. | Not understaning Danish incapable of complying with the trial protocol. | cancer |
| Hoek *et al* 2017^23^  The Netherlands | Patient | To evaluate whether weekly SPC teleconsultations improves patient-experienced symptom burden when compared to “care as usual”. | multiple settings | Mono | Recruitment from outpatient clinic | Advanced cancer, reside at home, have a GP who agrees to participate, Karnofsky performance status of 60 or below, life expectancy of 3 months or less, not receiving any disease-modifying treatment | Active psychotic disorder or a serious cognitive disorder. | cancer |
| Kluger *et al* 2020^24^  United States of America | Patient | To compare outpatient integrated PC with standard care alone to evaluate its effectiveness on patient QOL, caregiver burden, and other patient-centered outcomes to understand long-term outcomes. | hospital outpatient model | Multi | Participants were referred from academic medical centers, community neurologists, regional PD support organizations, and clinical trial websites. | Probable PD; another Parkinson’s Spectrum Disorder, has moderate to high PC needs based on the Palliative Care Needs Assessment Tool modified for PD. | Urgent PC needs, unable to commit to study procedures, other illnesses that could require PC, or were already receiving PC. | noncancer |
| Liu *et al* 2022^25^  China | Patient | To gain insight into interdisciplinary collaborative hospice care (ICHC) provided to terminal geriatric cancer patients by an ICHC team and identify factors to ameliorate multidimensional hospice care | inpatient model | Mono | Screening | Pathologically confirmed stage III–IV solid malignant tumors, terminal stage and knew their condition and physical status; ECOG-Performance Status scores 3 points; palliative prognosis index ≥ 6 points; they chose death in hospital, cause of death was related to a malignant tumor. | Hospitalized time less than 72h, had communication difficulties, other serious diseases, died due to unexpected complications, participating in other clinical trials within 3 months. | cancer |
| Maltoni *et al* 2016^26^  Italy | Patient | To compare the impact of ‘standard cancer care + systematic EPC’ with that of ‘standard cancer care + on-demand EPC’ on patient-reported outcomes, use of health services and quality of end-of-life care in patients with advanced pancreatic cancer who were candidates for antitumour treatment. | hospital outpatient model | Multi | Screening of all newly referred patients | New diagnosed, advanced and/or metastatic pancreatic cancer, ECOG 0-2; life expectancy >2months; fit for antitumour treatment (chemotherapy or target therapy). | Patients already receiving PC, who had received prior chemotherapy for metastatic or advanced disease, or who had participated in a clinical trial | cancer |
| Nottelmann *et al* 2021^27^  Denmark | Patient | To investigate whether quality of life is improved by systematic use of early palliative care in the form of palliative rehabilitation. | hospital outpatient model | Mono | All staff involved in recruitment were given pocket cards with bullet points about the trial and suggested phrases for information of potential study participants. The recruitment rate was presented graphically at brief monthly staff meetings, thus creating an ongoing focus and a milieu where the personnel felt safe to engage with the research group about their experiences when recruiting. | Receiving systemic medical treatment for newly diagnosed metastatic or otherwise unresectable solid tumor, be fit to receive standard oncology treatment. | Patients who received specialised palliative care within a year prior involvement. | cancer |
| Patil *et al* 2021^28^  India | Patient | To determine whether the addition of EPC to standard therapy leads to an improvement in the quality of life (QOL), decrease in symptom burden, and improvement in overall survival in patients with head and neck cancer. | multiple settings | Mono | All patients were recruited after obtaining written informed consent. | Squamous cell carcinoma of the head and neck region with stage IV, ECOG 0-2; planned for palliative intent first line chemotherapy in a multidisciplinary joint clinic. | Patients with surgically resectable tumors, planned for definitive radiotherapy; Uncontrolled co morbidities, already receiving care from palliative care services. | cancer |
| Rogers *et al* 2017^29^  United States of America | Patient | To assess the impact of an interdisciplinary palliative care intervention combined with usual CHF management on CHF-related and overall quality of life in patients with advanced CHF (PAL-HF trial) | multiple settings | Mono | PAL-HF screened and enrolled both hospitalized patients (n =148) and recently discharged patients (n =2) who were at high risk of rehospitalization and mortality based on their ESCAPE (Evaluation Study of Congestive Heart Failure and Pulmonary Artery Catheterization Effectiveness) risk score | Hospitalization for acute CHF or within 2 weeks of discharge of a hospitalization for acute HF; Dyspnea at rest, ESCAPE risk score ≥4, or acute heart failure with signs/symptoms of volume overload. | Acute coronary syndrome within 30 days; Cardiac resynchronization therapy within the past 3 months or current plan to implant; Active myocarditis, constrictive pericarditis; Severe stenotic valvular disease amendable to surgical intervention; Anticipated heart transplant or ventricular assist device within 6 months; Renal replacement therapy. Patients with surgically resectable tumors; Planned for definitive radiotherapy; uncontrolled co morbidities, already receiving care from palliative care services, Planned for definitive radiotherapy. | noncancer |
| Scarpi *et al* 2019^30^  Italy | Patient | To evaluate early impact on quality of life (QOL), quality of care, and healthcare costs between palliative care (EPC) plus standard cancer care and standard cancer care plus on-demand EPC. | hospital outpatient model | Multi | All newly referred patients were considered for enrollment in the study | New inoperable locally advanced and/or metastatic gastric cancers, eligible for chemotherapy and/or new target drugs treatments, ECOG: 0-2; Life expectancy >2 months. | Patients already receiving care from the palliative care service; Already receiverd prior chemotherapy and/or new target drugs treatments. | cancer |
| Sidebottom *et al* 2015^31^  United States of America | Patient | To assess if inpatient PC for HF patients is associated with improvements in symptom burden, depressive symptoms, QOL, or differential use of services. | inpatient consulting model | Mono | Potentially eligible patients were identified using reports from the electronic health record (EHR). Eligibility was verified by reviewing patient records and talking with a floor nurse if needed. Patients determined to be eligible were visited by the research nurse who explained the study and enrolled patients who were interested in participating. | Adult inpatients with a diagnosis of acute HF | Patients in ICU, actively dying, cognitive impairments, already had a PC order request by their attending physician. | noncancer |
| Slama *et al* 2020^32^  Czech Republic | Patient | To evaluate the benefit of an early and systematic palliative intervention alongside standard oncology care compared with standard oncology care alone in patients with advanced solid tumors. | hospital outpatient model | Mono | Primary oncologists were asked to inform their patients about the trial and to refer patients interested in participation to a study coordinator nurse. | Newly diagnosed advanced solid cancer, who started the first line of palliative anticancer treatment, ECOG=0-2. | Not stated | cancer |
| Tattersall *et al* 2014^33^  Australia | Patient | To improve patients’ EOL experiences through better symptom control and quality of life; addressing patients’ supportive care needs; reducing the lines of chemotherapy delivered; and reducing the likelihood of dying in the acute hospital setting through early contact with palliative care services. | hospital outpatient model | Mono | Ambulatory patients with newly detected incurable metastatic cancer attending a medical oncolgoy clinic with a life expecancy of less than 12 months were invited to take part in this study. | Life expectancy <1 2months; newly detected incurable metastatic cancer. | Not stated | cancer |
| Temel *et al* 2010^34^  United States of America | Patient | To examine the effect of introducing palliative care early after diagnosis on patient-reported outcomes and end-of-life care among ambulatory patients with newly diagnosed disease. | hospital outpatient model | Mono | From outpatient thoracic oncology: All the medical oncologists in the clinic agreed to approach, recruit, and obtain consent from their patients. | Pathologically confirmed metastatic NSCLC diagnosed within the previous 8 weeks; ECOG 0-2. | Already receiving care from the palliative care service. | cancer |
| Temel *et al* 2020^35^  United States of America | Patient | The primary aim of this study was to confirm the improvements in QOL with early PC for patients with advanced cancer. | multiple settings | Multi | Oncology clinicians identified eligible patients who were not receiving treatment with curative intent and invited them to participate. | Newly diagnosis of incurable solid cancer lung, ECOG=0-2, must be under the care of an oncologist. Participating sites: were required to have PC clinics with at least six months of experience providing care in the outpatient oncology setting, be led by a board-certified PC physician or advanced practice nurse (APN) | Non stated. | cancer |
| Vanbutsele *et al* 2020^36^  Belgium | Patient | To evaluate the effect of early integrated palliative care in oncology on QOL near the end of life and use of health care resources near the end of life. | multiple settings | Mono | In and outpatients were considered for inclusion. | Advanced cancer diagnosis, ECOG: 0-2; estimated life expectancy of 12 months, within the first 12 weeks of a diagnosis | Impaired cognition, more than one palliative care consultation since the onset of the disease, one palliative care consultation in the six months before inclusion. | cancer |
| Wong *et al* 2016^37^  China | Patient | To examine the effects of home-based transitional palliative care for patients with end-stage heart failure after hospital discharge. | home setting | Multi | An advanced practice nurse (APN) helped to screen cases for eligibility and confirmed the recruitment with the physician. | End stage heart failure, repeated hospital admissions (three within 1 year) with symptoms of HF and existence of physical/ psychological symptoms, contactable by phone and referral accepted by PC team. | Discharged to institutions, severe psychiatric disorders, recruited to other programmes. | noncancer |
| Woo *et al* 2019^38^  South Korea | Patient | To assess whether early palliative care (EPC) targeting pain and depression and automated symptom monitoring could improve symptoms in patients with advanced pancreatobiliary cancer. | hospital outpatient model | Mono | Patients who presented to the outpatient cancer clinic were invited by their physicians to enroll in the study; all the physicians in the clinic agreed to approach, recruit, and obtain consent from their patients. | Newly, pathologically confirmed locally advanced or metastatic Pancreatic cancer or Biliary Tract Cancer KPS ≥50% and cancer-related pain (BPI worst pain score >3) and/or depression (CES-D >16) | Not stated | cancer |
| Zimmermann *et al* 2014^39^  Canada | Medical oncology clinics | To postulated that, compared with standard cancer care, early intervention (clinical prognosis of 6–24 months) by a palliative care team would be associated with improved patient quality of life, symptom control, and satisfaction with care, and less difficulty with clinician–patient interactions. | hospital outpatient model | Multi | Daily screening of participating oncology clinics by research personnel to establish eligibility | Stage IV cancer, estimated survival of 6–24 months, ECOG: 0-2 | Cognitive impairment | cancer |

*Abbreviations:* AML = Acute Myeloid Leukemia, APN = Advanced practice nurse, BPI = Brief Pain Inventory, CES-D = Center for Epidemiologic Studies Depression Scale, CHF = chronic heart failure, CNS = clinical nurse specialist, COPD = chronic obstructive pulmonary disease, ECOG = Eastern Cooperative Oncology Group, EDSS = Expanded Disability Status Scale, EGFR = Epidermal growth factor receptor, EHR = electronic health record, EOL = end of life, EORTC-QLQ-C30 = European Organization of the Research and Treatment of Cancer Quality of Life Questionnaire - C30, EPC = early palliative care, GAP index = multidimensional index consisting of Gender [G], Age [A], and two lung physiology variables [P], forced vital capacity [FVC] and diffusion lung carbon monoxide [DLCO], GP = general practitioner, HF = heart failure, HSCT = hematopoietic stem cell transplantation, ICHC = interdisciplinary collaborative hospice care, ICU = intensive care unit, ILD = interstitial lung disease, IPC = integrated palliative and oncology care, KCCQ = Kansas City Cardiomyopathy Questionnaire Short Version, KPS = Karnofsky Performance Status, LVAD = Left-ventricular assistive device, MCO = managed care organizations, MDT = multidisciplinary team, MPM = malignant pleural mesothelioma, MS = multiple sclerosis, NSCLC = non-small cell lung cancer, PC = palliative care, PD = Parkinson's Disease, QOL = quality of life, SOC = standard oncologic care, SPC = specialist palliative care

*Reference numbers to included studies are different from the main publication.

## Table B. Description of intervention characteristics of included studies (k = 39)

| **Study** | **Label intervention** | **Comparison** | **Intervention description** | **Intervention duration** | **Control description** | **Contamination likely?** |
| --- | --- | --- | --- | --- | --- | --- |
| Aiken *et al* 2006^1^  United States of America | PhoenixCare Intervention + PhoenixCare Palliative intervention services | Usual care (case management) | **Nurse-led PC:** PhoenixCare Intervention + PhoenixCare palliative intervention services = Interdisciplinary team approach (RNs=case management; medical director, social worker and pastoral counselor were consultants to the case manager). Three distinct care protocols addressed phases of service delivery: (1) admission and initial case management of medically unstable patients, (2) management of stable patients following stabilization, and (3) support of unstable patients experiencing an exacerbation episode. All three protocols provided disease and symptom management (at admission medical management and emergency response plan), educational services (Advances care planning, disease and health promotion education, self-monitoring of disease), and support services (Psychological, spiritual and emotional support and counseling, access to community resources, transportation and help with finances). When patient was stable: all three categories/classes continued as needed (evaluation, assessment, educational activities, ongoing monitoring for support services) When illness- and symptom exacerbation: RN case managerr actively got involved in symptom control with patients and relationves and involving interdisziplinary team). | 15months | Usual case management - (case management was medical and disease orientated). Service delivery via telephone and home visits. Referral to support services. Telephone interviews every three months were study specific. | No |
| Bakitas *et al* 2009^2^  United States of America | ENABLE II | Usual care | **Nurse-led PC:** The intervention, based on the chronic care model, used a case management, educational approach to encourage patient activation, selfmanagement, and empowerment. The strategies used in the author’s prior studies were refined and converted to a manualised, telephone-based format to improve access to palliative care in a rural population. A nurse with specialist training in palliative care carried out four initial structured educational and problem-solving sessions and at least monthly telephone follow-up sessions until the participant died or the study ended. A bereavement follow-up call was made to the caregiver. | From enrolment until death or study completion (42months) | Usual care: Patients were allowed to use all oncology and supportive services without restrictions including referral to the institutions’ interdisciplinary palliative care service. Advanced illness care programm available for oncology staff. | Yes |
| Bakitas *et al* 2015^3^  United States of America | ENABLE III | Usual care | **Nurse-led PC:** The ENABLE study comprised an initial in-person, standardised outpatient palliative care consultation by a board-certified palliative care clinician and six structured weekly telephone coaching sessions by an advanced practice nurse using a manualised curriculum. Sessions covered problem-solving, symptom management, self-care, identification and co-ordination of local resources, communication, decision-making and advance care planning as well as a life-review approach that supported participants to redefine advanced illness. After the sessions, the nurse followed up patients via the telephone to provide further support. Nurse coach training included self-study, review of treatment manuals and scripts, and role-playing with feedback. The study principal investigator met with the nurse coaches weekly to review and provide feedback on difficult cases. | Enrolment until death or study completion (17months) | Medical oncologist-directed anticancer and symptom control treatments and consultations with oncology and supportive care specialists, including palliative care team, Palliative care provided when requested | Yes |
| Bakitas *et al* 2020^4^  United States of America | ENABLE CHF-PC | Usual HF Care | **Nurse-led PC:** In-person comprehensive Palliative Care Team Consultation- as soon as feasible after enrollment. PNC embedded within HF teams, instituting a phone-based 6-session patient and a 4-session caregiver curriculum followed by monthly phone-based supportive care for 48 weeks or patient death. The PNC uses the manualized curriculum: Charting Your Course: An Intervention for Patients with Heart Failure and their Families". | 16 weeks | Usual heart failure care includes any available supportive care resources and heart failure patient medical management based on national HF guidelines. Additional consultative services, such as palliative care, were requested based on clinical evaluation. | Yes |
| Bassi *et al* 2021^5^  Italy | Palliative care for ILD | Usual care | **Integrated collaborative care:** Patients in the intervention group were scheduled to meet, at least every 6 weeks, for the whole duration of the study, a physiotherapist, a psychologist, a dedicated nurse, a palliative care doctor, and a pulmonologist experienced in ILD care. In the intervention group, the visit addressed several topics which included evaluation of patient’s understanding of their illness and prognosis and establishing goals of care. Medical therapy (anxiolytic and anti-depressive medications, low-dose opioids, cough depressant, oxygen titration, etc.) was also initiated or changed to ameliorate the symptoms. Patients allocated to the intervention group also participated in a rehabilitation program consisting of breathing and motor exercises. Pulmonary rehabilitation programs provided to patients with interstitial lung diseases typically include a period of 3-4 weeks of daily incremental exercise training sessions | 12 months | Patients in the usual care group followed the regular center schedule for medical visits (at least 3 times in a year) with a pulmonologist experienced in ILD care; medical therapy was also initiated or changed to ameliorate the symptoms. | No |
| Bekelman *et al* 2018^6^  United States of America | CASA intervention | Usual care | **Integrated collaborative care:** Three components: A registered nurse (symptom care), social worker (structured psychosocial care), and a team (including the nurse and social worker, a primary care clinician, palliative care physician and cardiologist) reviewed the care provided to the patient and, when needed, ordered tests and medications. The patient and the nurses decided on one symptom that needed to be addressed (either pain, breathlessness, fatigue, or depression). The nurse using a structured guideline for behavioural and paliative approaches for the chosen symptom. Telephone based, routine assessment and structured intervention, support to caregivers as needed by social worker. Case review by PC Team. Case review by cardiologist. Six twice monthly visits by the nurse, which were mostly conducted by telephone using structured symtpom rating scale. Training in communication, motivational interviewing and the symptom guidelines was received by the nurse. Nurse and social worker were not trained in specialist palliative care. | 6 months | Primary care physician or Nurse Practitioner. Access to social worker was granted for almost all participants. Visits as needed (in general every 3-6months). Unstructured symptom assessment and management as needed. Psychosocial assessment and management on referral to social work or mental health (Patients, who flagged with significant depressive symptoms were notified as well as their clinicians. Care was then at discretion of clinician with no constraints on treatment or referrals). In-person visit by SPC when consulted. Many patients were already in the care of a cardiologist. Ad hoc telephone and between-visit care. | Yes |
| Bekelman *et al* 2022^7^  United States of America | ADAPT | Usual care | **Integrated collaborative care:** The intervention includes 3 components: 1) nurse (RN) follows structured algorithms to help patients with symptoms, specifically breathlessness, fatigue, and pain. 2) social worker provides structured counseling targeting adjustment to illness and depression and advance care planning. 3) collaborative care model of care delivery, in which the nurse and social worker meet weekly with a primary care provider and palliative care specialist. This team makes medical recommendations to the intervention subjects' providers and supervises the nurse and social worker. The team has as-needed consultation with a cardiologist or pulmonologist. The nurse and social worker visits are in-person or by phone | Not stated | Patients in the control group will continue to receive care at the discretion of their providers, which may include referrals to and ongoing care from cardiology, pulmonary, palliative care, or mental health. They will also have the same amount of interaction with research assistants as the intervention patients, completing questionnaires and participating in study visits at the same frequency. Patients' providers will be given the results of baseline depression surveys if they screen positive for depression, and patients will be given an information sheet that outlines self-care for CHF or COPD. | Yes |
| Benthien *et al* 2020^8^  Denmark | DOMUS | Usual care | **Specialised palliative home care:** Accelerated transition program from oncological treatment to continuous specialized palliative care and psychological intervention at home: Discussion of patients and carers wishes for SPC in their own home. Perception of challenges and concerns from patients and caregivers are explored and addressed. Conditions at home are optimized if needed. Home conference with SPT (Nurse, GP, study psychologist) after 4-5 days of randomisation. SPT, GP, and DNs are now responsible for delivery of tasks related to treatment and care. Patient and caregiver are offered psychological intervention according to the study intervention manual. After patients death, caregivers will be offered 1-2 additional sessions. | Six months. If patients in the intervention arm was still alive after six months, they were offered to remain in the study. | In the control group, the patients continue to be attached to the health care system in line with current practice. This means that the patient typically remains in hospital or ambulatory and may have contact with one or more hospitals, GP and possibly homecare later in the process. Caregivers in the control group may receive psychological counseling through referral from a GP. | No |
| Brännström *et al* 2014^9^  Sweden | PREFER Intervention | Usual HF care | **Specialised palliative home care:** Hospital outreach model: patients in the intervention group were offered a multidisciplinary approach involving collaboration between specialists in palliative and HF care. The intervention (structured, person-centred care, PCC) was delivered at home. PCC involves joint working between patients/carers and professional caregivers, including documenting the partnership. The nurses used a model of PCC that incorporated the six Ss, namely self-image, self-determination, social relationships, symptom control, synthesis and surrender. The clinical team was responsible for managing co-morbidities. Symptom assessment, QoL, and risks of decubitus, falling, and malnutrition were done using validated questionnaires. | 6 months - after this patients were transferred to the original care provider following the establishment of an individual care plan. | Mainly provided by GP or doctors and/or nurse-led CHF clinic at geriatrics department. | No |
| Brims *et al* 2019^10^  Australia | Early specialist palliative care | Standard care | **Early palliative care:** Specialist palliative Care (SPC) team consultation within three weeks of allocation. Carers encouraged to attend. Regular consultation every four weeks until end of study. Standardised SPC Assessment with Sheffield Profile for Assessment and Referral to Care (SPARC) tool and ESAS-r at initial consultations. Consultations include assessment of physical, psychological, social and spiritual needs of the participant, with appropriate provision of additional medicine (for example, analgesia) and referral to additional support services as required. | Every four weeks for 12 weeks (primary endpoint) and at least 24 weeks, until death or end of trial. | Appropriate and routine treatment (no treatment withheld) for MPM in UK and AUS; Patients discussed at specialist MDT incl. SPC representation with consensus for a treatment plan. Frequent contact with patients/carers (symptom management, advice on legal compensatin, and prep for chemotherapie) at the beginning. CNS (thoracic cancer or mesothelioma) available to all patients. SPC referral based on clinical need. Standardised SPC Assessment with Sheffield Profile for Assessment and Referral to Care (SPARC) tool and ESAS-r if and when referred. | Yes |
| Do Carmo *et al* 2017^11^  Brazil | Brief psychosocial intervention based on CBT + Early PC (Arm A: CBT-based psychosocial intervention + early PC combined with standard cancer treatment); Arm B: early PC combined with standard cancer treatment; Arm C: standard cancer treatment.) | Control group | **Early palliative care:** Arm A: Two psychologists conducted study procedures: five weekly (every week for five weeks) individual sessions performed in a room fit to receive the participants. Provision of psychoeducation on the patients’ current clinical condition and the aims of PC, the functioning of anxiety, and techniques to manage symptoms, discuss depressive symptoms, and techniques for the detection and questioning of automatic thoughts as well as their influence and essential role in the triggering of emotions and behaviors. The first PC appointment were planned to occur after the first two sessions of the psychosocial and educational intervention for participants who are allocated to Arm A.  ArmB: Palliative Care visits every 3 (±1) weeks. First visit was two to three weeks after randomisation. Study trained PC doctors following a standardised protocol. | 5 weeks | Patients with advanced cancers are referred to PC as per attending oncologists’ decisions; there is no standard protocol guiding referrals. Availability in hospital of outpatients clinic and inpatient ward including medical team and comprehensive MDT. | Yes |
| Edmonds *et al* 2010^12^  United Kingdom | Multiprofessional palliative care team assessment and follow-up | Standard care for 12 weeks, then received intervention (delayed intervention trial) | **Specialist palliative care:** Following the initial assessment of patient symptoms and psychosocial and advanced care planning needs, as well as carer needs, an action plan was developed and shared with the primary health-care team and other involved professionals as appropriate. Follow-up telephone calls or visits were arranged depending on clinical need. The clinical team had weekly meetings during which the palliative care consultant made recommendations about patient management. Based on the information collected during patient assessments and response to measures in the action plan, the consultant assessed if patients had ongoing specialist palliative care needs. Those who did were referred on to existing specialist community palliative care teams. Patients also received standard care. | 12 weeks | Usual care: - nurses (specialised in MS), physiotherapy, nerology, rehabilitation services - district nurses, social services, GP community support - inpatient care as needed - continence advice, psychiatry/psychology - MS Society support | No |
| El-Jahwari *et al* 2016^13^  United States of America | Palliative care intervention, inpatient consulting model | Standard care | **Specialist palliative care:** Patients met with the inpatient palliativecare physician or advanced practice nurse within 3 days of randomisation. At least twice per week, the palliative care clinician followed up patients during hospitalisation to address symptom management. Additional visits could be carried out as needed. There was no outpatient palliative care follow-up after discharge. After each visit, the palliative care clinicians communicated their recommendations to the transplant team and documented their recommendations in the medical record. | Period of hospitalisation | Standard transplant care: Supportive care measures instituted by transplant team. Patients, caregivers, and transplant clinicians permitted to request consultations with the palliative care clinicians. Thereafter, individual palliative care clinicians may follow standard transplant care participants per their clinical judgment, rather than according to the required twice weekly visits. (In the transplant unit at MGH, palliative care is consulted in less than 5% of patients admitted for HSCT, and therefore we anticipate minimal cross contamination in this study) | Yes |
| El-Jahwari *et al* 2021^14^  United States of America | Integrated Palliative and Oncology Care (IPC) | Usual care | **Integrated collaborative care:** Palliative care clinicians initially focused on establishing rapport, assessing palliative care needs,and developing a relationship with the patient. Throughout hospitalization (two visits per week after randomisation during all hospitalisations up to one year after randomisation), clinicians addressed patients’symptoms, assessed their illness understanding, ascertained their goals and expectations, and assisted with their treatment decision-making. Palliative care clinicians documented the elements of care that they addressed after each visit by using a structured questionnaire in the Research Electronic Data Capture (REDCap) system. | 2, 4, 12, and 24weeks | Patients assigned to usual care received supportive care measures as per their oncology team. They were permitted to receive palliative care at their request or at the request of their oncologist. | Yes |
| Evans *et al* 2021^15^  United Kingdom | SIPScare – Short-term integrated palliative and supportive care and usual primary and community healthcare | Usual primary and community health care  (SIPS after 12 weeks, delayed intervention trial) | **Integrated collaborative care:** Multidimensional person-centred palliative care assessment, then multidisciplinary review and management, with coordination of care usually by a nurse specialist in palliative care. Delivered over-12 weeks with up to three-visits/contacts. Integrated care between the palliative care team, GP and community nursing services though primary care multidisciplinary team review, information sharing on assessments and care and treatment plans. Face-to-face assessments with phone or face-to-face follow-ups for regular review. | 12 weeks | Usual care from primary and community healthcare, and social care providers informed by individual assessment, priorities and preferences and agreed goals of care. Face-to-face or phone contacts, as per usual clinical practice. Patients in the control arm were offered SIPScare after 12-weeks. | No |
| Eychmüller *et al* 2021^16^  Switzerland | Early palliative care intervention + usual oncology care | Usual oncology care | **Early palliative care:** Single intervention within 16 weeks of enrollment by a SPC physician and a SPC nurse. Patients were encouraged to bring a family member. Structured face-to-face conversation of about 50 min according to our SENS structure (Symptoms and their management; End-of-life desicions; Network; Support of the carers). Specific questions following the SENS-structured intervention to enhance further discussions with professionals were handed out to the patients. Follow-up visits were allowed on request by patients PLUS usual oncology care. | 50 minutes | Participants were treated primarily by oncologists who were trained in both communication and tumor-centered care but not in specialized, palliative medicine. Outpatient consultations involve a systematic, structured oncology survey sheet providing information on drug therapy, severity of major symptoms, physical examination, imaging, and laboratory diagnostics. On request, specialist palliative care consultation was performed by a SPC physician and a SPC nurse. In addition, psycho-oncologists, social workers, pastors, and nutritionists, were available on demand, rather than offered to participants. | Yes |
| Franciosi *et al* 2019^17^  Italy | EPC (Early palliative care) | SOC (Standard oncolgoy care) | **Early palliative care:** Patients had a meeting with the palliative care team within 2 weeks of enrolment, and at least every 2–3 weeks thereafter for 24 weeks. Additional visits with the palliative care team were available based on request from the patient, oncologist or palliative care provider. General guidelines for the palliative care visits were adapted from the protocol of the Temel 2010 study. Care provided was documented in a patient’s medical record by the palliative care team. Physical and psychosocial symptoms were assessed using validated instruments, and services were provided based on patients’ needs. | Enrolment to 24 weeks or death | Standard care: Anticancer and symptom control treatments provided by oncologists and nurses without formal PC training, PC referral available upon request - those who did did not cross-over or received the specified PC protocol | Yes |
| Gao *et al* 2020^18^  United Kingdom | Short-term integrated palliative care (SIPC) | Standard care (control), SIPS after 12 weeks (delayed intervention trial) | **Integrated collaborative care** Comprehensive assessment, personalized care planning, case management and care coordination, and advising existing care providers. Delivered by: SPC teams linked with local neurological services. Content of intervention: visit within 5 working days after referral. Visit 1: Comprehensive assessment with patient and caregiver; generating problem list; development of mutually agreed care plan. Visit 2 (person or telephone and within 2 weeks of visit 1): r/v progreass with the care plan. Visit 3 (final contact) r/v of outcomes from actions already taken before d/c to local services as appropriate. | 6-8 weeks from referral | Within study sites (description of study sites in general n=7), a broad range of services were offered, including voluntary and National Health Services hospices, hospital and community based multidisciplinary palliative care, as well as tertiary an secondary neurological services. This group was offered SIPC intervention after 12 weeks. | No |
| Given *et al* 2002^19^  United States of America | Nurse-led intervention + Conventional care as prescribed by oncology care team and telephone interviews. | Conventional care as prescribed by oncology care team and telephone interview. | **Nurse-led PC**: guided by a cognitive behavioural framework directed towards pain, fatigue, and 12 other common symptoms. Content of cognitive behavioural framework: utilising problem-solving approaches to symptom management+improving physical functioning and emotional health = nurses were assisting patients in acquiring knowledge, skills, behavioural reframing, support necessare to manage problems. Frequency of intervention: 10 visits/contacts (six in person and four via telephone) every two weeks. Qualitative Interviews with all patients at baseline (within eight weeks of commencing chemotherapy), week ten, and week 20 via telephone. | 20 weeks | Qualitative Interviews with all patients at baseline (within eight weeks of commencing chemotherapy), week ten, and week 20 via telephone. | No |
| Goldstein *et al* 2022^20^  United States of America | Mount Sinai Palliative care at home intervention | Usual care from primary care physician | **Specialist palliative home care**: Patients/caregivers will be cared for by an interdisciplinary team that includes a social worker, nurse, community health worker, nurse practitioner, and physician.  Patients randomized to the intervention will be scheduled for an intake visit. This visit will be undertaken by the team's registered nurse and/or social worker, together with a community health worker, and other team members (advanced practice nurse, MD), depending on patients' needs. Following this visit, and in conjunction with the nurse practitioner and/or MD, a care plan will be developed to address areas of clinical need highlighted during the intake visit.Patients in the intervention arm will receive ongoing monitoring and input (telephone-based and in-person) from members of the clinical team, dependent on their identified needs. Patients' cases will be discussed at the weekly IDT meeting, as appropriate to the level of clinical need. Patients and caregivers will be provided with access to a 24 hour telephone line, staffed by a Mount Sinai based physician, which acts as an advice line out of hours. These physicians will be able to provide advice to patients and caregivers. | Not stated | Usual Care - Patients will be cared for by the physician who treats their primary illness(es). | No |
| Greer *et al* 2022^21^  United States of America | Palliative care intervention | Usual care | **Integrated collaborative care**: Five four weekly structured visits (first visit withing four weeks of enrollment) addressing rapport building, symptom management, illness understanding, coping, treatment decision-making, and EOL/advance care planning. PC visits at the same day as oncology visits. The PC clinicians received an email prior to each structured visit reminding them of the intervention content. *No clinicians received training in documenting patient EOL care preferences.* PC clincians completed a survey to capture content adressed during visit. Oncology treatment by treating oncologist who was already involved before enrollement. | 20 weeks | Meeting with a PC clinician only upon request of the patient/caregiver or oncologist. When these patients received PC, they did not follow the intervention protocol.Oncology treatment by treating oncologist who was already involved before enrollement. | Yes |
| Groenvold *et al* 2017^22^  Denmark | DanPaCT (early SPC ) AND Standard care | Standard care | **Early palliative care:**: DanPaCT intervention (early SPC = usual SPC initiated at an earlier time): Number, frequency, and location of visits determined by the patient's needs following the WHO and the EAPC guidelines for palliative care. Procedures, activities and processes were those normally used by the corresponding teams (Oncology and PC) and the interventions were given by the staff normally providing the interventions. After completion of 8week trial period, patients remained in contact with SPC team if clinically relevant. | 8 weeks | Patients continue with their standard treatment. Standard care (Procedures, activities and processes were those normally used by the oncology team and the interventions were given by the staff normally providing the interventions. Potentially included palliative care provided by the departments of oncology, general practitioners (GPs) or home care services. | Yes |
| Hoek *et al* 2017^23^  The Netherlands | Teleconsultations AND care as usual | Care as usual | **Specialist palliative care**: Weekly teleconsultations (NP or physician) via teleconsultation device (iPad) installed in patients home. Evaluation of patient if not seen before by SPCT. Patient could not contact SPCT via teleconsultations but by phone in between visits if needed. When in need of medical advice, patients were encouraged to contact GP. Strong involvement of GP during teleconsultations to discuss patients needs and treatment. GP was left with a discharge letter from the SPCT outlining the patient’s current problems and needs and advised treatment policies . | 13 weeks | Patients could be referred to the SPCT by their GP or by the attending hospital specialist or were not referred at all. If applicable, follow-up by the SPCT occurred by phone or by patients visiting the outpatient clinic, depending on the patient’s preference, the complexity of their problems, and/or the stage of their disease. | Yes |
| Kluger *et al* 2020^24^  United States of America | Outpatient integrated PC AND standard care | Standard care | **Integrated collaborative care**: Oupatient PC + Standard care: Participants could elect to transfer their neurology care to the PC team to consolidate care. Received outpatient PC visits (in person or telemedicine) every 3 months for 1 year were supplemented with phone calls at the discretion of the PC team, and participants could contact the PC team as needed. After-visit summaries were provided to the patient, and standard clinic notes were provided to the primary care physician and neurologist. Suggestions for care outside of PC issues were provided to the patient’s standard care team. The interdisciplinary team consisted of a palliative neurologist with informal training in PC (eg, education through a palliative and end-of-life care workshop); a nurse, social worker, and chaplain with PD experience; and a boardcertified palliative medicine physician. PC models varied in clinic flow between centres. | 52weeks | Provided by a neurologist and a primary care practitioner. Practitioners can refer patients to other services at their discretion. Patients who were not established with a neurologist at enrollment were scheduled for an appointment with a neurologist to establish care. | No |
| Liu *et al* 2022^25^  China | Interdisciplinary collaborative hospice care (ICHC) | Life-sustaining treatments (LSTs) | **Specialist palliative care**: No intention to treat: Holistic hospice care and optimal collaboration based on four core competency domains of values and ethics, roles and responsibilities, communication, and teamwork. Our ICHC team provided a curriculum focused on team building, knowledge, and skills of TGCPs and HC, quality improvement assessment methods, also access to expert consultation and community resources using a hybrid learning model. | From admission until death. | Active treatment, necessary symptoms management and communication, etc. by physicians. When necessary, pharmacists provided medication consultation and monitoring. Nurses performed nursing evaluation and care. The patient families provided daily care and emotional communication, and patients participated in the whole process. Some patients might have been crossed over and underwent ICHC. | Yes |
| Maltoni *et al* 2016^26^  Italy | Standard cancer care plus systematic Early Palliative Care (EPC) | Standard cancer care plus on-demand EPC  (delayed intervention trial) | **Early palliative care:**: Visit with PC specialist, who had predefined checklist (same as Temel study) of issues addressed during consultation. Consultation every 2-4weeks until death. Availability between appointments not scheduled in the protocol, but according to the clinical and organisational solutions, was present in every centre. Moreover, every researcher could have adjunctive routine tools of assessment, not considered in the present study. Interventions followed general PC guidelines. | 12(±3) weeks | Not scheduled to meet the PC team unless they, their families, or the attending oncologist requested an appointment. Availability between PC appointments not scheduled in the protocol, but according to the clinical and organisational solutions, was present in every centre. Moreover, every researcher could have adjunctive routine tools of assessment, not considered in the present study. After max 15weeks, patients were followed by the PC team as needed. | Yes |
| Nottelmann *et al* 2021^27^  Denmark | Early palliative care in the form of palliative rehabilitation AND Standard care | Standard care | **Early palliative care:**: The intervention consisted of a ‘basic offer’ and tailored elements. The basic offer was two mandatory consultations and the option of contacting a palliative rehabilitation team directly during the 12-week participation period, if needed. Furthermore, patients and family caregivers could be offered participation in a 12-week patient/ caregiver school, combined with individually tailored physical exercise in groups, individual consultations with members of the palliative rehabilitation team, or both. At the end of the first consultation, the patient and family caregivers were given the team’s contact information. All specialist palliative care team members except the chaplain offered individual consultations to patients and family caregivers in the palliative rehabilitation clinic or over the telephone. The specialist palliative care team had weekly multidisciplinary conferences during which they discussed patients. | 12 weeks | Standard care was provided at the discretion of the medical oncologist. Upon indication, referral could be made to specialized palliative care, dieticians, physiotherapists, and psychosocial support from chaplains, psychologists, and social workers. Thus, patients in the standard arm were not refused help with palliative or rehabilitation needs emerging during their treatment. | Yes |
| Patil *et al* 2021^28^  India | Early palliative care AND standard care | Standard oncology care | **Early palliative care:**: Patients received standard oncologic care as outlined in control condition and were referred to the Department of Palliative medicine immediately after random assignment. Palliative medicine experts conducted nursing, medical, psychological, symptom, and social assessment followed by interventions in the form of counseling, symptom management, home-based palliative care support, liaison with family physicians, hospice referral, and liaison with psycho-oncologist/psychiatrist if there were complex psychological issues. Minimum of monthly consultations for three months. Both arms: continued chemotherapy and regular radiology assessment every 2months. | 12 weeks | Patients received the standard oncologic care, which consisted of chemotherapy, supportive care, symptom management, counseling, nutritional support, social support, and optimization of appointments with other specialties. Both arms: continued chemotherapy and regular radiology assessment every 2months. | No |
| Rogers *et al* 2017^29^  United States of America | Interdisciplinary palliative care intervention AND Evidence-based HF care | Evidence-based HF care | **Integrated collaborative care**: The study team assessed and managed the different domains of QoL for patients with advanced HF. A certified palliative care nurse practitioner co-ordinated patient care in collaboration with a hospice and palliative medicine board-certified physician. The intervention was performed in collaboration with each patient’s clinical cardiology team and focused on shared goal-setting to combine HF symptom amelioration with palliativecare goals. After hospital discharge, the PAL-HF nurse practitioner actively participated in the ongoing management of the patients in the outpatient environment. After the 6-month intervention period was completed, the nurse practitioner continued to contact the patients in the intervention arm every 3 months to provide ongoing support and clinical care. | 6 months | Patients under usual care were managed by a cardiologist-directed team with HF expertise. Inpatient care focused on symptom relief and use of evidence-based therapies as detailed in current guidelines. Inpatient palliative care consultation was available on request. After discharge, patients received outpatient follow-up with their GPs, as well as with a HF cardiologist or nurse practitioner. | No |
| Scarpi *et al* 2019^30^  Italy | Early Palliative care AND standard cancer care | On-demand EPC AND standard cancer care | **Early palliative care:**: Pateints had an appointment scheduled with a PC specialist who had a predefined checklist of issues to be addressed during the consultation. The checklist of topics to be discussed during the visit of PC was the same as that used by Temel. Patients met a PC physician within 2 weeks of enrollment and were seen every 2 to 4 weeks thereafter until death. In both arms, non protocol-scheduled meetings with a PC specialist were possible when needed. Moreover, each researcher had the option of using adjunctive routine tools of assessment that were not considered in the present study. PC appointments and interventions were scheduled on the basis of general PC guidelines. | 2 to 4weeks after enrolement and thereafter until death. | In both arms, non protocol-scheduled meetings with a PC specialist were possible when needed. Moreover, each researcher had the option of using adjunctive routine tools of assessment that were not considered in the present study. PC appointments and interventions were scheduled on the basis of general PC guidelines. | Yes |
| Sidebottom *et al* 2015^31^  United States of America | Inpatient Palliative care | Control group | **Specialist palliative care**: Following randomisation, the intervention group received a palliative care consult from the hospital palliative care team. The intervention was not the same as the standard palliative care process as baseline assessments of depression, QoL and symptoms could be reviewed before patients were seen by the team, as well as changes to payment for the hospital palliative care service. Areas covered by the hospital palliative care team during patient visits included symptom assessment; psychosocial, emotional and spiritual care; care co-ordination; treatment recommendation referrals; and future care-planning assessment and discussions. | Period of hospitalisation | Not described | Unclear |
| Slama *et al* 2020^32^  Czech Republic | Palliative systemic therapy AND standard oncology care | Standard oncology care | **Early palliative care:**: The palliative intervention consisted of consultation with palliative care physician every six to eight weeks. Consultations included pain and other symptom assessment made by the Edmonton Symptom Assessment Scale (ESAS), assessment of patient’s coping strategies and the need for psychosocial support. The average duration of the first visit with a palliative care physician was 45 minutes, follow-up visits took*20 minutes. Besides pharmacological symptom management recommendations, a palliative care specialist also explored patient’s individual psychological and spiritual needs and referred them to other members of the team (social worker, psychologist, or chaplain) when an unmet need was discovered. | Every six to eight weeks for 6months. | An on-demand consultation with palliative care physician as well as other supportive/ palliative services requested by the treating oncologist were available. The study appointments with PC physician had usually the character of a consultation (recommendation about symptom management had been given to primary oncologist). In some patients co-management model had been practiced (PC physician directly prescribed medication for pain/non-pain symptoms). | Yes |
| Tattersall *et al* 2014^33^  Australia | Early Palliative Care | Standard oncologic care | **Early palliative care:**: The patient met with a palliative care nurse consultant (PC nurse) member of the hospital palliative care team. She outlined available palliative care services including advice about symptom control, and she offered to arrange review by a palliative care physician, and provided contact details for the palliative care service. The PC nurse offered to telephone the patient monthly to check on their well-being, or, if the patient preferred, provided her contact details. | During the lifespan of the patient | Standard oncologic care was given in line with the oncologist’s recommendation. Control patients were referred to the palliative care service when recommended by the oncologist. | Yes |
| Temel *et al* 2010^34^  United States of America | Early palliative care integrated with standard oncologic care | Standard oncologic carestandard oncologic care | **Early palliative care:**: Patients assigned to early palliative care met with a member of the palliative care team, comprising boardcertified palliative care physicians and advanced practice nurses, within 3 weeks of enrolment and at least monthly thereafter in the outpatient setting until death. Additional visits with the palliative care service could be requested by the patient, oncologist or palliative care provider. Guidelines for the palliative care visits were adapted from the National Consensus Project for Quality Palliative Care. Palliative care clinicians documented the care they provided according to these guidelines. All the participants continued to receive routine oncologic care throughout the study period. | Montly after enrolement and thereafter until death. | Patients in the standard care group did not meet with the palliative care service unless a meeting was requested by the patient, the family or the oncologist. Those who were referred to the service did not cross over to the palliative care group or follow the specified palliative care protocol. | No |
| Temel *et al* 2020^35^  United States of America | Early PC integrated with oncology care | Usual oncology care | **Early palliative care:**: According to the study protocol, patients assigned to early PC should meet with a PC physician or APN in the cancer center within four weeks of enrollment and at least monthly until death, in conjunction with patients’ scheduled oncology visits. If in-person visit not possible, the PC clinician contacted patient via telephone. Evidence-based PC practice model: developing and maintaining the therapeutic relationship with patients and caregivers; assessing and treating patients’ symptoms; providing support and reinforcement of coping with advanced cancer in patients and caregivers; assessing and enhancing prognostic awareness and illness understanding in patients and caregivers; assisting with treatment decision making; and planning for EOL care. | Montly after enrolement and thereafter until death. | Patients assigned to usual oncology care were able to meet with a PC clinician on request by the oncologist, patient, or caregiver. | Yes |
| Vanbutsele *et al* 2020^36^  Belgium | Early and systematic PC integrated in usual care | Usual care | **Early palliative care:**: Patients consulted with nurses from the PC team within 3 weeks of enrolment and monthly thereafter. Additional PC consultations could be scheduled at the discretion of the patients. The nurses focused on illness understanding, symptom burden, and provided support in decisionmaking, and support for emotional, social, and/or spiritual needs. | 12 weeks | Patients assigned to usual care only met with the PC team on demand of the patient or treating physician, these patients did not cross over to the early and systematic PC arm. | Yes |
| Wong *et al* 2016^37^  China | Home visit/telephone calls | Usual care | **Specialist palliative care**: Transitional Care Palliative-ESHF: The 4Cs are comprehensiveness, continuity, coordination and collaboration in alignment with the PC principles from Europe and Canada in providing continuous and coordinated care with multidisciplinary support. Intervention delivered by Nurse case manager (qualified PC nurses with experience in caring for pateints with HF). Home visits/telephone calls every week for the first month and less frequently during the subsequent months. First visit was an in person visit. | 12months | Both groups received usual care, which consisted of PC medical clinic consultation, discharge advice on symptom management and medication and referrals if appropriate (eg, home visits). Placebo calls: The control group received two attention control social calls from an assistant, consisting of light conversation topics unrelated to clinical issues | Yes |
| Woo *et al* 2019^38^  South Korea | Early palliative care AND routine oncologic care | Oncologic care | **Early palliative care:**: The early palliative care intervention included the following: (1) nursing assessment of pain and depression, (2) pain control based on NCCN guidelines, (3) depression control by psychoeducation and/or consultation with a psychiatric specialist and (4) patient education. Patients were managed by research nurses trained in symptom assessment and medication adherence; pain and depression education; and in making treatment adjustments according to NCCN guidelines. Patients with CES-D scores of > 25 were referred to psychiatric specialists. The interventions were delivered by telephone or during regularly scheduled outpatient care. Follow-up intervention visits or telephone coaching were scheduled daily until BPI worst pain score was ≤ 3. Telephone calls were triggered when patients reported inadequate symptom improvement, non-adherence to medication, adverse effects or suicidal ideation, or when patients requested to be contacted | 12 months | The control group received no formal intervention, but were informed of their depressive and pain symptoms. Their screening results were provided to their physician. Usual oncology care was directed by an attending physician and consisted of anticancer and symptom control treatments and consultation with psychiatric and pain care specialists. Pain care specialists were provided whenever requested, regardless of group assignment | No |
| Zimmermann *et al* 2014^39^  Canada | Early palliative care | Standard cancer care | **Early palliative care:**: The core intervention was consultation and follow-up in the oncology palliative care clinic by a palliative care physician and nurse, consisting of: (1) comprehensive, multidisciplinary assessment of symptoms, psychological distress, social support, and home services, within 1 month of recruitment (60–90 min duration); (2) routine telephone contact from a palliative care nurse 1 week after the first consultation, and thereafter as needed; (3) monthly outpatient palliative care follow-up (20–50 min); and (4) a 24-h on-call service for telephone management of urgent issues. Ancillary Interventions depending on the status of the patient, included: arrangement of home nursing care services; transfer of care to a home palliative care physician (when the patient’s ECOG performance status was 3 or worse, or when requested); and admission to the Princess Margaret Cancer Centre palliative care unit for urgent symptom control or terminal care. | 4 months | The control group received no formal intervention, but palliative care referral was not denied, if requested. Participants in the control group referred to the palliative care service received the same care as patients in the intervention group, but did not have the same standardised monthly follow-up. | Yes |

## Table C. Description of characteristics of outcome measures in included studies (k = 39)

| **Study*** | **Primary outcome** | **All QOL or emotional wellbeing outcomes** | **Primary endpoint in weeks** | **All endpoints in weeks** | **Outcome scored by** | **Primary outcome as stated in paper** | **Secondary outcome as stated in paper** |
| --- | --- | --- | --- | --- | --- | --- | --- |
| Aiken *et al* 2006^1^  United States of America | SF-36 | SF-36 physical role functioning  SF-36 General health | 36 | 36 | patient | Physical and mental functioning  Memorial Symptom Assessment Scale  SF-36  Number of A&E visits in the past six months & number of A&E visits during the study | - |
| Bakitas *et al* 2009^2^  United States of America | FACIT-PAL46 | FACIT-PAL46  ESAS  CES-D | 52 | 4  16  28  40  52 | patient | Patient-reported QOL: FACIT-PAL46 Symptom intensity: ESAS-r Resource use: number of days in hospital, number of days in the ICU, number of emergency department visits | Emotional wellbeing: CES-D  Caregiver burden: Zarit Burden Inventroy  Perceptions of end-of-life care  Survival |
| Bakitas *et al* 2015^3^  United States of America | FACIT-PAL46 | FACIT-PAL46  FACIT TOI  QUAL-E symptom impact  CES-D | 13 | 13  28  52 | patient | Patient-reported QOL: FACIT-PAL46 (including TOI), CES-D, QUAL-E  Patient reported quality of dying: QUAL-E  Emotional wellbeing: CES-D  Survival: Vital status, time from enrolment to death  Resource use: ICU days, A&E visits, Chemotherapy use within 14 days of death, location of death | - |
| Bakitas *et al* 2020^4^  United States of America | KCCQ | KCCQ  FACIT-PAL14  HADS-A  HADS-D | 16 | 8  16 | patient, caregiver | Patient-reported QOL: KCCQ, FACIT-PAL-14  Emotional wellbeing: HADS | General Domains of health: Global Health-10  3-item Pain Intensity  2-item Pain Inference Scale |
| Bassi *et al* 2021^5^  Italy | Borg Dyspnoea | Maugeri Respiratory Questionnaire  CES-D | 52 | 26  52 | patient | Patient-rated dyspnea: Borg scale (0-10 NRS)  Cough: VAS  Emotional wellbeing: CES-D  Patient-reported QOL: Maugeri Respiratory Questionnaire reduced form | Survival  Changes in physical function: Spirometry, lower body flexibility (chair sit-and-reach test) and strength (30s Chair-Stand test) |
| Bekelman *et al* 2018^6^  United States of America | KCCQ | KCCQ  PHQ-9  GAD-7 | 26 | 13  26 | patient | Patient-reported disease-specific QOL:  KCCQ | Emotional wellbeing: Depression with PHQ-9, Anxiety with GAD7  Overall symptom distress: General Symptom Distress Scale Pain and shortness of breath: three items derived from the BPI Fatigue: Patient-Reported Outcomes Measurement Information System - Short Form  Mortality: Medical records Resource use: Number of hospitalisations |
| Bekelman *et al* 2022^7^  United States of America | FACT-G | FACT-G | 26 | 26 | patient | Patient-reported QOL: FACT-G | Symptom burden: General Symptom Distress Scale, PEG (pain intensity and inference), Insomnia Severity Index, PROMIS Fatigue  Emotional wellbeing: PHQ-9, GAD-7  Patient-reported QOL: KCCQ-Short form, Clinical COPD Questionnaire, QUAL-E, K-BILD  Advance care planning communication  Hospitalizations  Survival |
| Benthien *et al* 2020^8^  Denmark | ESAS | ESAS  ESAS wellbeing item | 26 | 8  26 | patient | Place of care and death | Patient-reported symptom burden: ESAS-r  Survival time  Health care utilisation: Use of medicine, Healthcare services and informal care; Cooperation and quality of the process; Caregiver wellbeing: anxiety and depression, prolonged grief, caregiver QOL, caregiver use of services  Dyadic coping  Preference for place of treatment and care |
| Brännström *et al* 2014^9^  Sweden | EQ5D | EQ-5D VAS | 26 | 26 | patient | Symptom burden: ESAS Patient health status: EQ-5D Patient-reported QOL: KCCQ NYHA Functional classes | Resource use: Number of hospitalisations Number of days spent in hospital |
| Brims *et al* 2019^10^  Australia | EORTC QLQ C30 Global health | EORTC QLQ C30 Global health  SF-36 physical  SF-36 mental  GHQ-12 | 13 | 13  26 | patient | Patient-reported QOL: EORTC QLQ-C30 | Emotional wellbeing: GHQ-12  Survival  Primary caregiver QOL: SF-36, GHQ-12  Satisfaction with care: FAMCARE-2  Cost-effectiveness: EQ-5D |
| Do Carmo *et al* 2017^11^  Brazil | PHQ-9 | EORTC QLQ-C15 PAL global health  HADS-A  HADS-D  PHQ-9  EORTC QLQ-C15PAL emotional function | 13 | 6  13  16  26 | patient | Emotional wellbeing: HADS-D, PHQ-9  Satisfaction with care: FAMCARE-patient scale | Emotional wellbeing: HADS-A  Cancer understanding with adapted instrument  Symptom burden: ESAS  Patient-reported QOL: EORTC QLQ-C15PAL |
| Edmonds *et al* 2010^12^  United Kingdom | MSIS | POS-8  MS-POS 5 symptoms  MS-POS  MSIS Physical subscale  MSIS Psychological subscale | 13 | 6  13 | patient | Patient symptoms and concerns: POS and MS-POS  Patient-reported QOL: MSIS Caregiver burden: 12-item ZBI Caregiver mastery: Lawton positivity questionnaire | - |
| El-Jahwari *et al* 2016^13^  United States of America | FACT-BMT | FACT-BMT  ESAS  HADS-A  HADS-D  PHQ-9 | 2 | 2  13 | patient | Patient-reported QOL: FACT-BMT  Perception of PC intervention | Patient-reported QOL: FACT-BMT  Emotional wellbeing: HADS, PHQ-9, PTSD – Civilian version, NCCN Distress Thermometer  Fatigue: FACT fatigue subscale  Symptom burden: ESAS-r  Incidence of acute or chronic graft vs host disease Non-relapse mortality (Exploratory) Overall survival (Exploratory) Caregiver QoL: CareGiver Oncology Quality of Life questionnaire Caregiver emotional wellbeing: HADS, PHQ-9 Resource use: Number of palliative care visits |
| El-Jahwari *et al* 2021^14^  United States of America | FACT-Leu | FACT-Leu  ESAS  HADS-A  HADS-D  PHQ-9 | 2 | 2  4  13  26 | patient | Patient-reported QOL: FACT-Leu | Emotional wellbeing: HADS, PHQ-9, PTSD-Civilian version  Symptom burden: ESAS-r |
| Evans *et al* 2021^15^  United Kingdom | IPOS-5 | IPOS-5  IPOS physical  EQ5D-5L | 13 | 6  13 | patient | 5 key symptoms (pain, breathlessness, patient anxiety, drowsiness and constipation) from the IPOS | Patient symptom and concerns: IPOS  Patient functioning: Barthel index  Caregiver burden: Carer Zarit Burden  Economic evaluation: EQ-5D Crosswalk Index, IPOS |
| Eychmüller *et al* 2021^16^  Switzerland | NCCN distress thermometer | NCCN distress thermometer  FACT-G  POS score  FACT-G | 26 | 8  16  26 | patient | Emotional wellbeing: NCCN Distress thermometer. | Patient-reported QOL: FACT-G  Symptom burden: POS  Overall survival  Location of death  Health care utilization: Stanford Patient Education Research Centre Questionnaire |
| Franciosi *et al* 2019^17^  Italy | FACT-G | FACT-G | 13 | 13 | patient | Patient-reported QOL: FACT-G | Survival Use of end-of-life care (percentage of deceased patients who used the following in the 30 days prior to death: chemotherapy, hospital admission, ED visits) |
| Gao *et al* 2020^18^  United Kingdom | IPOS Neuro-S8 | IPOS Neuro-S8  IPOS Neuro-S24  IPOS Neuro  HADS-A  HADS-D  EQ5D VAS | 13 | 13 | patient | 8 key palliative care symptoms (pain, shortness of breath, nausea, vomiting, constipation, spasms, difficulty sleeping, and mouth problems) from the IPOS-Neuro | Patient sympom and concerns: IPOS Neuro  Caregiver burden: Zarit Burden Inventory  Healthcare costs: Client Services Receipt Inventory |
| Given *et al* 2002^19^  United States of America | Symptom Experience Scale | Symptom Experience Scale  SF-36 physical role functioning  SF-36 social functioning | 20 | 10  20 | patient | Symptom burden: Symptom Experience scale  Patient-reported QOL: SF-36 physical role functioning, SF-36 social functioning | - |
| Goldstein *et al* 2022^20^  United States of America | McGill QoL | McGill QoL | 26 | 26 | patient | Patient symptom and concerns: IPOS | Patient-reported QOL: McGill QOL  Family satisfaction with EOL care: FAMCARE-10  Caregiver burden: Zarit burden inventory  Process measures: number of completed advanced directives, number of preference of consistency in care  Caregiver emotional wellbeing: PHQ-9  Health care utilization: Number of inpatient admissions, ED visits, outpatient appointments |
| Greer *et al* 2022^21^  United States of America | EoL preferences | FACT-B  HADS-A  HADS-D | 26 | 6  13  18  26 | patient | Documentation of EOL Care preferences: Natural Language Processing (Clinical Regex) methods (EHR was queried with validated algorithm identifying goals of care and EOL discussions) | Patient-reported QOL: FACT-B  Emotional wellbeing: HADS  EOL care discussions and prognostic awareness (2 dichotomous questions)  Health record review (tumor biomarkers, presence of brain metastasis, smoking status, ECOG, cancer treatment, hospice referral) |
| Groenvold *et al* 2017^22^  Denmark | EORTC most severe symptom | EORTC QLQ-C30 physical functioning  EORTC QLQ-C30 role functioning  EORTC QLQ-C30 emotional functioning  EORTC QLQ-C30 Global health  EORTC QLQ-C30 Sum score  HADS-A  HADS-D | 3 | 3  8 | patient | Change in patient's primary need (the most severe of the 7 EORTC QLQ-C30 scales) | Patient-reported QOL: EORTC QLQ-C30  Survival  Resource use |
| Hoek *et al* 2017^23^  The Netherlands | ESAS | ESAS  HADS-A  HADS-D | 13 | 4  8  13 | patient | Symptom burden: ESAS  Emotional wellbeing: HADS | Number of hospital admissions  Experienced problems and needs for palliative care  Patient and caregiver satisfaction with teleconsultation Experienced continuity of medical care in the last phase of life  Caregiver burden |
| Kluger *et al* 2020^24^  United States of America | QOL-AD | QOL-AD  ESAS-PD  PDQ-39  HADS-A  HADS-D | 26 | 26  52 | patient | Patient-reported QOL: QOL-AD  Caregiver burden: Zarit Burden Inventory-12 | Symptom burden: ESAS-PD, PDQ-39  Emotional wellbeing: HADS, FACIT-Sp  Caregiver emotional wellbeing: HADS, Prolonged Grief Disorder questionnaire 12-items, FACIT-Sp  Patient and caregiver clinical global impression (0-7 point scale) |
| Liu *et al* 2022^25^  China | Survival time | EORTC QLQ-C30 Physical function  EORTC QLQ-C30 Emotional function  EORTC QLQ-C30 Global health  Hamilton Anxiety Scale | 13 | 2  13 | proxy | Median survival  Patient-centered QOL: EORTC QLQ-C39  Satisfaction with care: FAMCARE-P16 | Healthcare use: Median average daily cost of drugs Symptom burden  Emotional wellbeing: Hamilton Anxiety Scale |
| Maltoni *et al* 2016^26^  Italy | FACT-HEP | FACT-HEP  FACT-HEP HCS  FACT-HEP TOI | 13 | 13 | patient | Patient-reported QOL: FACT-HEP | Emotional wellbeing: HADS  Caregiver satisfaction with care: FAMCARE  Overall survival |
| Nottelmann *et al* 2021^27^  Denmark | EORTC QLQ-C30 | EORTC QLQ-C30 Global health | 13 | 13 | patient | Area under the curve for the symptom/problem prioritised by the patient on the adapted form of the EORTC QLQ-C30 | Patient-reported QOL: EORTC QLQ-C30  Survival  Healthcare costs: ED consultations; hospitalisation days  Health service use (number and duration of hospital admissions and treatments, and visits to outpatient clinics, to the ED or GP). |
| Patil *et al* 2021^28^  India | FACT H&N total | FACT-G  FACT-H&N total  FACT H&N TOI | 13 | 4  8  13 | patient | Patient-reported QOL: FACT-H&N | Patient-reported QOL: FACT H&N  Symptom burden: ESAS-r  Survival |
| Rogers *et al* 2017^29^  United States of America | KCCQ | KCCQ  FACIT-PAL14  HADS-A  HADS-D | 26 | 2  13  26 | patient | Patient-reported QOL: KCCQ, FACIT-PAL14 | Emotional wellbeing: HADS, FACIT-Sp  Healthcare use: Number of hospitalizations  Survival |
| Scarpi *et al* 2019^30^  Italy | FACT-Ga TOI | FACT-Ga score  FACT-Ga GaCS  FACT-Ga TOI | 13 | 13 | patient | Patient-reported QOL: FACT-Ga | Family satisfaction with care: FAMCARE  Survival |
| Sidebottom *et al* 2015^31^  United States of America | MLHFQ | ESAS  PHQ-9  MLHFQ | 13 | 4  13 | patient | Symptom burden: ESAS  Emotional wellbeing: PHQ-9  Patient-reported QOL: MLHFQ | Advance care planning  Resource use: inpatient 30-day readmission, hospice use  Survival |
| Slama *et al* 2020^32^  Czech Republic | EORTC QLQ-C30 | EORTC QLQ-C30 Global health  EORTC QLQ-C30 Physical function  EORTC QLQ-C30 Emotional function  HADS total  HADS-A  HADS-D | 13 | 13  26 | patient | Patient-reported QOL: EORTC QLQ-C30  Emotional wellbeing: HADS | Overall survival  EOL outcomes: Use of anticancer treatment in the last 30 days of life; enrolment in hospice services; place of death |
| Tattersall *et al* 2014^33^  Australia | McGill QOL | McGill QOL  RSCL physical  RSCL psychological | 52 | 4  13  26  36  52 | patient | Patient-reported QOL: McGill QOL  Symptom burden: Rotterdam Symptom Checklist  Emotional wellbeing: Rotterdam Symptom Checklist  Healthcare Needs: Supportive Care Needs-Short Form | EOL outcomes: Number of lines of chemotherapy, place of death. |
| Temel *et al* 2010^34^  United States of America | FACT-L | FACT-L  FACT-L LCS score  FACT-L TOI | 13 | 13 | patient | Patient-reported QOL: FACT-L | Emotional wellbeing: HADS, PHQ-9  Survival  Place of death  Healthcare costs and resource use: Number of palliative care visits, use of health services and EOL care including anticancer therapy, medication prescriptions, referral to hospice, hospital admissions, ED visits |
| Temel *et al* 2020^35^  United States of America | FACT-G | FACT-G  HADS-A  HADS-D | 13 | 13  26 | patient | Patient-reported QOL: FACT-G | Emotional wellbeing: HADS  Communication about prognosis and EOL care: Prognosis and Treatment Perceptions Questionnaire |
| Vanbutsele *et al* 2020^36^  Belgium | EORTC QLQ-C30 Global health | EORTC QLQ-C30 Global health  McGill single QOL item | 13 | 13  26  48 | patient | Patient-reported QOL: EORTC QLQ-C30, McGill QOL single item | Illness understanding  Influence of palliative care on the decision of physicians with regards to EOL care |
| Wong *et al* 2016^37^  China | readmission | ESAS  CHFQ total  McGill QOL | 4 | 4 | patient | Readmission in days (over 4 weeks) | Readmission in days (over 12 weeks)  Symptom burden: ESAS  Functional Status: Palliative Performance Scale  Patient-reported QOL: CHFQ, McGill QOL  Satisfaction with care |
| Woo *et al* 2019^38^  South Korea | EORTC QLQ-C30 Global health | EORTC QLQ-C30 Global health  EORTC QLQ-C30 Emotional function  Satisfaction with pain management  CES-D | 4 | 4  13  26  52 | patient | Symptom burden: Pain with Brief Pain Inventory  Emotional wellbeing: CES-D | Patient-reported QOL: EORTC QLQ-C30  Sleep disturbance: Insomnia Severity Index  Satisfaction with pain control  Patient and caregiver global impression. Clinical global Impression Improvement Scale  Survival |
| Zimmermann *et al* 2014^39^  Canada | FACIT-Sp | FACIT-Sp  QUAL-E  ESAS | 4 | 4  8  13  16 | patient | Patient-reported QOL: FACIT-Sp | Patient-reported QOL. FACIT-Sp, QUAL-E  Symptom burden: ESAS-r  Satisfaction with care: FAMCARE-P16, The Cancer Rehabilitation Evaluation System Medical Interaction Subscale (CARES-MIS) |

*Abbreviations*: A&E = Accident and emergency, BPI = Brief Pain Inventory, CES-D = Center for Epidemiologic Studies Depression Scale, CHFQ = Chronic Heart Failure Questionnaire, ECOG = Eastern Cooperative Oncology Group, ED = emergency department, EHR = electronic health record, EOL = end of life, EORTC QLQ-C15PAL = European Organization for the Research and Treatment of Cancer Quality of Life Questionnaire - C15 Palliative care, EORTC QLQ-C30 = European Organization for the Research and Treatment of Cancer Quality of Life Questionnaire - C30, EQ5D = European Quality of Life with 5 Dimensions, ESAS-PD = Edmonton Symptom Assessment Scale-Parkinson's Disease, ESAS-r = Edmonton Symptom Assessment Scale-revised, FACIT-PAL46 = Functional Assessment of Cancer Therapy-Palliative care 46-item questionnaire, FACIT-Sp = Functional Assessment of Cancer Therapy-Spiritual wellbeing, FACT-B = Functional Assessment of Cancer Therapy-Breast, FACT-BMT = Functional Assessment of Cancer Therapy-Bone marrow transplant, FACT-G = Functional Assessment of Cancer Therapy-General, FACT-Ga = Functional Assessment of Cancer Therapy-Gastric cancer, FACT-Ga GaCS = Functional Assessment of Cancer Therapy-Gastric Cancer Scale, FACT-HEP = Functional Assessment of Cancer Therapy-Hepatobiliary, FACT-HEP HCS = Functional Assessment of Cancer Therapy-Hepatobiliary Cancer Scale, FACT-H&N = Functional Assessment of Cancer Therapy-Head & Neck Cancer, FACT-L = Functional Assessment of Cancer Therapy-Lung, FACT-L LCS = Functional Assessment of Cancer Therapy-Lung cancer scale, FACT-Leu = Functional Assessment of Cancer Therapy-Leukemia, FAMCARE = Family Satisfaction with Advanced Cancer, GAD-7 = Generalised Anxiety Disorder Assessment - 7 item version, GHQ-12 = General Health Questionnaire-12 item version, GP = general practitioner, HADS-A = Hospital Anxiety and Depression Scale-Anxiety subscale, HADS-D = Hospital Anxiety and Depression Scale - Depression subscale, IPOS = Integrated Palliative Care Outcome Scale, k = number of studies, KCCQ = Kansas City Cardiomyopathy Questionnaire, ICU = Intensive care unit, McGill QOL = McGill Quality of Life Questionnaire, MHLHFQ = Minnesota Living with Heart Failure Questionnaire, MS-POS = Palliative care Outcome Scale - Multiple Sclerosis, MSIS-29 = Multiple Sclerosis Impact Scale-29 item version, NCCN = National Comprehensive Cancer Network, NRS = numerical rating scale, NYHA = New York Heart Association, PDQ-39 = Parkinson Disease Questionnaire-39 item version, PHQ-9 = Patient Health Questionnaire-9 item version, POS = Palliative care outcome scale, PROMIS = Patient-Reported Outcomes Measurement Information System, PTSD = Posttraumatic Stress Disorder, QOL = Quality of life, QOL-AD = Quality of Life - Alzheimer's Disease Questionnaire, QUAL-E = Quality at the end of life questionnaire, RSCL = Rotterdam Symptom Checklist, SF-36 = Medical Outcomes Study Short Form-36 item questionnaire, TOI = Trial outcomes index, VAS = Visual analogue scale, ZBI = Zarit Burden Inventory

*Reference numbers to included studies are different to those from the main publication.

## Table D. Sample characteristics (k = 39 RCTs)

| **Study*** | **Disease** | **Disease group** | **Mean age** | **% Female** | **% Nonwhite** | **% Informal carer** | **% Advanced disease severity** | **Proportion with comorbidity** | **Charlson comorbidity index** | **% ECOG status over 3** | **% Attrition** |
| --- | --- | --- | --- | --- | --- | --- | --- | --- | --- | --- | --- |
| Aiken *et al* 2006^1^ | CHF and COPD | noncancer | 68.5 | 64 | 18 | 87 | - | - | - | - | 62 |
| Bakitas *et al* 2009^2^ | Cancer | cancer | 65.3 | 39.8 | 1.4 | 73.1 | - | - | - | 30 | 32.3 |
| Bakitas *et al* 2015^3^ | Cancer | cancer | 64.3 | 47.3 | 3.4 | 59.9 | 30.4 | - | 6.25 | 5 | 25.1 |
| Bakitas *et al* 2020^4^ | CHF | noncancer | 63.8 | 49.2 | 55.7 | 48.4 | 42.4 | - | 3.301 | - | 41 |
| Bassi *et al* 2021^5^ | ILD | noncancer | 75.9 | 24 | - | - | 100 | 46 | - | - | 24 |
| Bekelman *et al* 2018^6^ | CHF | noncancer | 65.5 | 21.3 | 28.1 | - | 61.8 | - | - | - | 23.7 |
| Bekelman *et al* 2022^7^ | CHF, COPD, ILD | noncancer | 68.9 | 9.8 | 19.9 | - | - | - | - | - | 24 |
| Benthien *et al* 2020^8^ | Cancer | cancer | 65.5 | 51.2 | - | 67.1 | - | - | - | - | 56.8 |
| Brännström *et al* 2014^9^ | CHF | noncancer | 79.3 | 29.2 | - | 61.1 | 97.2 | 86.1 | - | - | 33.3 |
| Brims *et al* 2019^10^ | Mesothelioma | cancer | 72.9 | 20.1 | - | 83.3 | 32.6 | 59.8 | - | 0 | 14.9 |
| Do Carmo *et al* 2017^11^ | Cancer | cancer | 54.9 | 63.6 | 20.5 | 65.9 | - | - | - | - | 18.2 |
| Edmonds *et al* 2010^12^ | Multiple sclerosis | noncancer | 53 | 69.2 | - | - | - | - | - | - | 40.4 |
| El-Jahwari *et al* 2016^13^ | Hematopoietic stem cell transplant | cancer | 57.1 | 56.9 | 13.1 | 73.8 | - | - | 3 | 0 | 1.9 |
| El-Jahwari *et al* 2021^14^ | Acute myeloid leukemia | cancer | 56.94 | 0.4 | 13.75 | 73.8 | 31.9 | - | - | - | 8.1 |
| Evans *et al* 2021^15^ | Older noncancer patients | noncancer | 85.7 | 48 | - | 74 | 80 | 98 | 3 | 50 | 6 |
| Eychmüller *et al* 2021^16^ | Cancer | cancer | 67.3 | 35.3 | - | - | 98 | - | - | - | 22 |
| Franciosi *et al* 2019^17^ | Cancer | cancer | 68.5 | 34.9 | - | 71.5 | 91.1 | - | - | 0 | 23.8 |
| Gao *et al* 2020^18^ | Neurological conditions | noncancer | 66.8 | 48.9 | 9.1 | 65.4 | - | 71.7 | - | 59.4 | 6.6 |
| Given *et al* 2002^19^ | Cancer | cancer | 57.9 | 71.7 | - | 100 | 69 | - | - | - | 1 |
| Goldstein *et al* 2022^20^ | Organ failure | noncancer | 74 | 57 | - | - | - | - | - | - |  |
| Greer *et al* 2022^21^ | Metastatic breast cancer | cancer | 57 | 100 | 12.5 | 68.3 | 26.6 | 26.6 | - | 0 | 34.2 |
| Groenvold *et al* 2017^22^ | Cancer | cancer | 65 | 57.9 | - | - | - | - | - | 12.8 | 12.7 |
| Hoek *et al* 2017^23^ | Cancer | cancer | 62.1 | 33.8 | - | 77 | - | - | - | - | 60.8 |
| Kluger *et al* 2020^24^ | Parkinson’s Disease | noncancer | 70.1 | 35.7 | 8.1 | 83.3 | 42.3 | 29.5 | - | - | 11.9 |
| Liu *et al* 2022^25^ | Cancer | cancer | 69.4 | 51.2 | 100 | - | 100 | - | - | 100 | 0 |
| Maltoni *et al* 2016^26^ | Gastric cancer | cancer | 63.7 | 42.5 | - | 69.4 | - | - | - | 0 | 37.7 |
| Nottelmann *et al* 2021^27^ | Cancer | cancer | 66 | 61 | - | 73 | 40 | - | - | 12 | 27 |
| Patil *et al* 2021^28^ | Cancer | cancer | 49.2 | 15.6 | - | - | 100 | - | - | 0 | 28.9 |
| Rogers *et al* 2017^29^ | CHF | noncancer | 70.9 | 47.3 | 42.7 |  | 54.7 | - | - | 60 | 46 |
| Scarpi *et al* 2019^30^ | Gastric cancer | cancer | 65.1 | 31.8 | - | 68.7 | - | - | - | 0 | 29.6 |
| Sidebottom *et al* 2015^31^ | CHF | noncancer | 73.4 | 47.4 | 6.1 | 53 | - | - | - | - | 28 |
| Slama *et al* 2020^32^ | Cancer | cancer | 62.4 | 40.5 | - | - | 100 | - | - | 0 | 19 |
| Tattersall *et al* 2014^33^  Australia | Cancer | cancer | 63.5 | 51.7 | 54.2 | - | 74.2 | - | - | - | 58.3 |
| Temel *et al* 2010^34^  United States of America | Cancer | cancer | 64.9 | 51.7 | 2.6 | 61.6 | 28.5 | - | - | 0 | 29.1 |
| Temel *et al* 2020^35^ | Cancer | cancer | 65.3 | 43.5 | 22.5 | 69.1 | - | - | - | 0 | 52.3 |
| Vanbutsele *et al* 2020^36^ | Cancer | cancer | 60 | 31.4 | - | - | - | - | - | 4.3 | 0.5 |
| Wong *et al* 2016^37^ | CHF | noncancer | 78.3 | 48.8 | - | 65.4 | 89.3 | - | - |  | 2.4 |
| Woo *et al* 2019^38^ | Cancer | cancer | 66.5 | 55.2 | - | - | - | - | - | 25 | 20.8 |
| Zimmermann *et al* 2014^39^ | Cancer | cancer | 60.7 | 56.6 | - | 81.6 | 28.2 | - | 3 | 0 | 28.6 |

*Abbreviations*: CHF = Chronic Heart Failure, COPD = chronic obstructive pulmonary disease, ECOG = European Cooperative Oncology Group, ILD = interstitial lung disease, k = number of studies, RCT = randomized controlled trial

*Reference numbers to included studies are different to those from the main publication.

**References to S7 Appendix**

1. Aiken LS, Butner J, Lockhart CA, Volk-Craft BE, Hamilton G, Williams FG. Outcome evaluation of a randomized trial of the PhoenixCare intervention: program of case management and coordinated care for the seriously chronically ill. J Palliat Med. 2006;9(1):111-126. doi:10.1089/jpm.2006.9.111

2. Bakitas M, Lyons KD, Hegel MT, et al. Effects of a palliative care intervention on clinical outcomes in patients with advanced cancer: the Project ENABLE II randomized controlled trial. JAMA. 2009;302(7):741-749. doi:10.1001/jama.2009.1198

3. Bakitas MA, Tosteson TD, Li Z, et al. Early Versus Delayed Initiation of Concurrent Palliative Oncology Care: Patient Outcomes in the ENABLE III Randomized Controlled Trial. J Clin Oncol. 2015;33(13):1438-1445. doi:10.1200/JCO.2014.58.6362

4. Bakitas MA, Dionne-Odom JN, Ejem DB, et al. Effect of an Early Palliative Care Telehealth Intervention vs Usual Care on Patients With Heart Failure: The ENABLE CHF-PC Randomized Clinical Trial. JAMA Intern Med. 2020;180(9):1203-1213. doi:10.1001/jamainternmed.2020.2861

5. Bassi I, Guerrieri A, Carpano M, et al. Feasibility and efficacy of a multidisciplinary palliative approach in patients with advanced interstitial lung disease. A pilot randomised controlled trial. Pulmonology. 2023;29(Suppl 4):S54-S62. doi:10.1016/j.pulmoe.2021.11.004

6. Bekelman DB, Allen LA, McBryde CF, et al. Effect of a Collaborative Care Intervention vs Usual Care on Health Status of Patients With Chronic Heart Failure: The CASA Randomized Clinical Trial. JAMA Intern Med. 2018;178(4):511-519. doi:10.1001/jamainternmed.2017.8667

7. Bekelman D, Baron A, Paden G, et al. Effect of a collaborative palliative care intervention vs usual care on quality of life of patients with symptomatic heart and lung diseases: a randomized clinical trial (CO202A). J Pain Symptom Manage. 2022;63(Suppl):1063.

8. Benthien K, Diasso P, von Heymann A, et al. Oncology to specialised palliative home care systematic transition: the Domus randomised trial. BMJ Support Palliat Care. 2020;10(3):350-357. doi:10.1136/bmjspcare-2020-002325

9. Brännström M, Boman K. Effects of person-centred and integrated chronic heart failure and palliative home care. PREFER: a randomized controlled study. Eur J Heart Fail. 2014;16(10):1142-1151. doi:10.1002/ejhf.151

10. Brims F, Gunatilake S, Lawrie I, et al. Early specialist palliative care on quality of life for malignant pleural mesothelioma: a randomised controlled trial. Thorax. 2019;74(4):354-361. doi:10.1136/thoraxjnl-2018-212380

11. do Carmo TM, Paiva BSR, de Oliveira CZ, Nascimento MSA, Paiva CE. The feasibility and benefit of a brief psychosocial intervention in addition to early palliative care in patients with advanced cancer to reduce depressive symptoms: a pilot randomized controlled clinical trial. BMC Cancer. 2017;17(1):564. Published 2017 Aug 23. doi:10.1186/s12885-017-3560-6

12. Edmonds P, Hart S, Wei Gao, et al. Palliative care for people severely affected by multiple sclerosis: evaluation of a novel palliative care service. Mult Scler. 2010;16(5):627-636. doi:10.1177/1352458510364632

13. El-Jawahri A, LeBlanc T, VanDusen H, et al. Effect of Inpatient Palliative Care on Quality of Life 2 Weeks After Hematopoietic Stem Cell Transplantation: A Randomized Clinical Trial. JAMA. 2016;316(20):2094-2103. doi:10.1001/jama.2016.16786

14. El-Jawahri A, LeBlanc TW, Kavanaugh A, et al. Effectiveness of Integrated Palliative and Oncology Care for Patients With Acute Myeloid Leukemia: A Randomized Clinical Trial. JAMA Oncol. 2021;7(2):238-245. doi:10.1001/jamaoncol.2020.6343

15. Evans CJ, Bone AE, Yi D, et al. Community-based short-term integrated palliative and supportive care reduces symptom distress for older people with chronic noncancer conditions compared with usual care: A randomised controlled single-blind mixed method trial. Int J Nurs Stud. 2021;120:103978. doi:10.1016/j.ijnurstu.2021.103978

16. Eychmüller S, Zwahlen S, Fliedner MC, et al. Single early palliative care intervention added to usual oncology care for patients with advanced cancer: A randomized controlled trial (SENS Trial). Palliat Med. 2021;35(6):1108-1117. doi:10.1177/02692163211005340

17. Franciosi V, Maglietta G, Degli Esposti C, et al. Early palliative care and quality of life of advanced cancer patients-a multicenter randomized clinical trial. Ann Palliat Med. 2019;8(4):381-389. doi:10.21037/apm.2019.02.07

18. Gao W, Wilson R, Hepgul N, et al. Effect of Short-term Integrated Palliative Care on Patient-Reported Outcomes Among Patients Severely Affected With Long-term Neurological Conditions: A Randomized Clinical Trial. JAMA Netw Open. 2020;3(8):e2015061. Published 2020 Aug 3. doi:10.1001/jamanetworkopen.2020.15061

19. Given B, Given CW, McCorkle R, et al. Pain and fatigue management: results of a nursing randomized clinical trial. Oncol Nurs Forum. 2002;29(6):949-956. doi:10.1188/02.ONF.949-956

20. Goldstein N, Mather H, DeCherrie L, et al. A randomized controlled trial of a novel home-based palliative care team centered around community health workers improved patient outcomes (RP524). J Pain Symptom Manage. 2022;63(Suppl):1110–1111.

21. Greer JA, Moy B, El-Jawahri A, et al. Randomized Trial of a Palliative Care Intervention to Improve End-of-Life Care Discussions in Patients With Metastatic Breast Cancer. J Natl Compr Canc Netw. 2022;20(2):136-143. doi:10.6004/jnccn.2021.7040

22. Groenvold M, Petersen MA, Damkier A, et al. Randomised clinical trial of early specialist palliative care plus standard care versus standard care alone in patients with advanced cancer: The Danish Palliative Care Trial. Palliat Med. 2017;31(9):814-824. doi:10.1177/0269216317705100

23. Hoek PD, Schers HJ, Bronkhorst EM, Vissers KCP, Hasselaar JGJ. The effect of weekly specialist palliative care teleconsultations in patients with advanced cancer -a randomized clinical trial. BMC Med. 2017;15(1):119. Published 2017 Jun 19. doi:10.1186/s12916-017-0866-9

24. Kluger BM, Miyasaki J, Katz M, et al. Comparison of Integrated Outpatient Palliative Care With Standard Care in Patients With Parkinson Disease and Related Disorders: A Randomized Clinical Trial. JAMA Neurol. 2020;77(5):551-560. doi:10.1001/jamaneurol.2019.4992

25. Liu Y, Shen Y, Pan Q, et al. Application of interdisciplinary collaborative hospice care for terminal geriatric cancer patients: a prospective randomized controlled study. Support Care Cancer. 2022;30(4):3553-3561. doi:10.1007/s00520-022-06816-x

26. Maltoni M, Scarpi E, Dall'Agata M, et al. Systematic versus on-demand early palliative care: results from a multicentre, randomised clinical trial. Eur J Cancer. 2016;65:61-68. doi:10.1016/j.ejca.2016.06.007

27. Nottelmann L, Groenvold M, Vejlgaard TB, Petersen MA, Jensen LH. Early, integrated palliative rehabilitation improves quality of life of patients with newly diagnosed advanced cancer: The Pal-Rehab randomized controlled trial. Palliat Med. 2021;35(7):1344-1355. doi:10.1177/02692163211015574

28. Patil VM, Singhai P, Noronha V, et al. Effect of Early Palliative Care on Quality of Life of Advanced Head and Neck Cancer Patients: A Phase III Trial. J Natl Cancer Inst. 2021;113(9):1228-1237. doi:10.1093/jnci/djab020

29. Rogers JG, Patel CB, Mentz RJ, et al. Palliative Care in Heart Failure: The PAL-HF Randomized, Controlled Clinical Trial. J Am Coll Cardiol. 2017;70(3):331-341. doi:10.1016/j.jacc.2017.05.030

30. Scarpi E, Dall'Agata M, Zagonel V, et al. Systematic vs. on-demand early palliative care in gastric cancer patients: a randomized clinical trial assessing patient and healthcare service outcomes. Support Care Cancer. 2019;27(7):2425-2434. doi:10.1007/s00520-018-4517-2

31. Sidebottom AC, Jorgenson A, Richards H, Kirven J, Sillah A. Inpatient palliative care for patients with acute heart failure: outcomes from a randomized trial. J Palliat Med. 2015;18(2):134-142. doi:10.1089/jpm.2014.0192

32. Slama O, Pochop L, Sedo J, et al. Effects of Early and Systematic Integration of Specialist Palliative Care in Patients with Advanced Cancer: Randomized Controlled Trial PALINT. J Palliat Med. 2020;23(12):1586-1593. doi:10.1089/jpm.2019.0697

33. Tattersall MH, Martin A, Devine R, et al. Early contact with palliative care services: a randomized trial in patients with newly detected incurable metastatic cancer. J Palliat Care Med. 2014;4(1):1. doi:10.4172/2165-7386.1000170

34. Temel JS, Greer JA, Muzikansky A, et al. Early palliative care for patients with metastatic non-small-cell lung cancer. N Engl J Med. 2010;363(8):733-742. doi:10.1056/NEJMoa1000678

35. Temel JS, Sloan J, Zemla T, et al. Multisite, Randomized Trial of Early Integrated Palliative and Oncology Care in Patients with Advanced Lung and Gastrointestinal Cancer: Alliance A221303. J Palliat Med. 2020;23(7):922-929. doi:10.1089/jpm.2019.0377

36. Vanbutsele G, Van Belle S, Surmont V, et al. The effect of early and systematic integration of palliative care in oncology on quality of life and health care use near the end of life: A randomised controlled trial. Eur J Cancer. 2020;124:186-193. doi:10.1016/j.ejca.2019.11.009

37. Wong FK, Ng AY, Lee PH, et al. Effects of a transitional palliative care model on patients with end-stage heart failure: a randomised controlled trial. Heart. 2016;102(14):1100-1108. doi:10.1136/heartjnl-2015-308638

38. Woo SM, Song MK, Lee M, et al. Effect of Early Management on Pain and Depression in Patients with Pancreatobiliary Cancer: A Randomized Clinical Trial. Cancers (Basel). 2019;11(1):79. Published 2019 Jan 11. doi:10.3390/cancers11010079

39. Zimmermann C, Swami N, Krzyzanowska M, et al. Early palliative care for patients with advanced cancer: a cluster-randomised controlled trial. Lancet. 2014;383(9930):1721-1730. doi:10.1016/S0140-6736(13)62416-2
