# Supplementary material for: Benefits of specialist palliative care by identifying active ingredients of service composition, structure, and delivery model: A systematic review with meta-analysis and meta-regression
Source: PLoS Med. 2024 Aug 2;21(8):e1004436. doi: 10.1371/journal.pmed.1004436 (PMC11329153; doi:10.1371/journal.pmed.1004436)
Supplement: S8 Appendix — (DOCX) [file pmed.1004436.s008.docx]

**Benefits of specialist palliative care by identifying active ingredients of service composition, structure, and delivery model: A systematic review with meta-analysis and meta-regression**

**S8 Appendix**

Miriam J. Johnson, Leah Rutherford, Anisha Sunny, Sophie Pask, Susanne de Wolf-Linder, Fliss E. M. Murtagh, Christina Ramsenthaler

[hycr22@hyms.ac.uk](mailto:hycr22@hyms.ac.uk)

**GRADE table and risk of bias table**

## Table A. GRADE evidence profile for quality of life and emotional outcomes, specialist palliative care (SPC) versus usual care

| **No of trials** | **Number of patients**  **(number of events)** | **Outcome time horizon** | **Serious risk of bias^2^** | **Serious Inconsistency**  **(*I^2^*, *τ*)^3^** | **Serious Indirectness** | **Serious Imprecision^4^** | **Publication bias detected** | **Treatment effect, MID^5^**  **(95% CI)** | **Treatment effect, RR**  **(95% CI)** | **NNT^1^** |
| --- | --- | --- | --- | --- | --- | --- | --- | --- | --- | --- |
| **Quality of life** | | | | | | | | | | |
| 20 | 3034  (1125) | 2 to 11 weeks | No | No  (0%, 0) | No | No | No | 0.16  (-0.06, 0.38) | 1.04  (0.95, 1.14) | 69 |
| 27 | 3895  (1588) | 12 weeks | No | Yes  (43.6%, 0.13) | No | No | No | 0.50  (0.06, 0.93) | 1.14  (0.95, 1.36) | 15 |
| 33 | 4493  (1856) | 13 to 36 weeks | No | No  (0%, 0) | No | No | Yes | 0.40  (0.21, 0.59) | 1.13  (1.06, 1.20) | 20 |
| 9 | 777  (236) | 7 months to 1 year | No | No  (0%, 0) | No | Yes | No | 0.58  (-0.09, 1.26) | 1.31  (1.08, 1.59) | 12 |
| **Emotional wellbeing** | | | | | | | | | | |
| 14 | 2073  (670) | 2 to 11 weeks | No | Yes  (69.4%, 0.07) | No | Yes | No | 0.18  (-0.64, 0.99) | 1.07  (0.85, 1.35) | 46 |
| 18 | 2266  (703) | 12 weeks | No | No  (0%, 0) | No | No | No | 0.08  (-0.06, 0.23) | 1.12  (1.01, 1.25) | 28 |
| 21 | 2600  (899) | 13 to 36 weeks | No | No  (0%, 0) | No | No | No | 0.26  (-0.00, 0.52) | 1.16  (1.08, 1.24) | 19 |
| 7 | 481  (176) | 7 months to 1 year | No | No  (0%, 0) | No | No | No | 0.10  (-0.21, 0.41) | 0.97  (0.84, 1.12) | - |

1 Number needed to treat

2 We did not rate down for risk of bias, as we did not detect any significant difference between low and high risk of bias.

3 Serious inconsistency was judged based on the dichotomous effect size (RR, relative risk).

4 Serious imprecision was judged based on the dichotomous effect size (RR).

5 MID, minimal important difference

## Fig A. Risk of bias assessment for the randomized controlled trials included in this systematic review (k = 39)

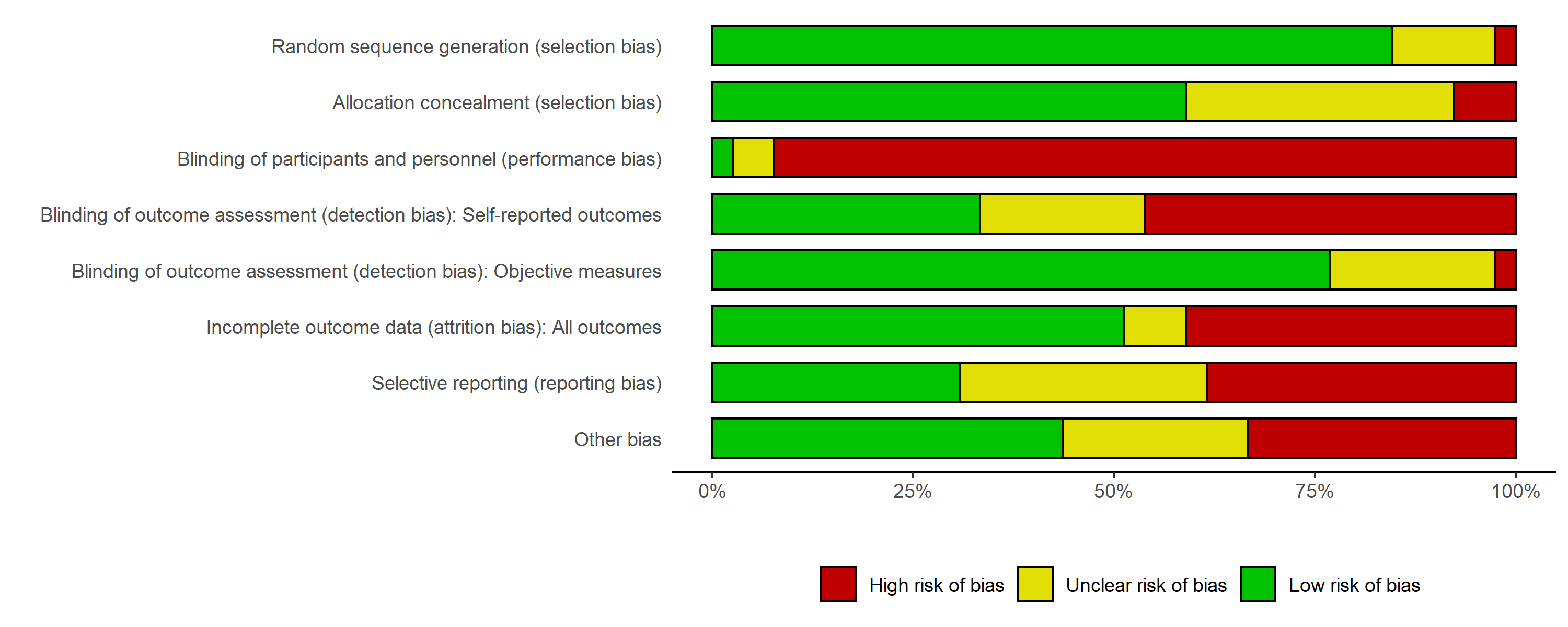


Symbols: (+) low risk of bias, (?) unclear risk of bias, (-) high risk of bias
