# Supplementary material for: Benefits of specialist palliative care by identifying active ingredients of service composition, structure, and delivery model: A systematic review with meta-analysis and meta-regression
Source: PLoS Med. 2024 Aug 2;21(8):e1004436. doi: 10.1371/journal.pmed.1004436 (PMC11329153; doi:10.1371/journal.pmed.1004436)
Supplement: S9 Appendix — (DOCX) [file pmed.1004436.s009.docx]

**Benefits of specialist palliative care by identifying active ingredients of service composition, structure, and delivery model: A systematic review with meta-analysis and meta-regression**

**S9 Appendix**

Miriam J. Johnson, Leah Rutherford, Anisha Sunny, Sophie Pask, Susanne de Wolf-Linder, Fliss E. M. Murtagh, Christina Ramsenthaler

[hycr22@hyms.ac.uk](mailto:hycr22@hyms.ac.uk)

**Detailed results for all meta-analysis and meta-regressions**

## Fig A and Table A: Quality of life at 2 to 11 weeks

**Analysis with effect size SMD (MID units)**

| **Study (k = 20)** | **SPC**  **MD_change_** | | **SPC**  **Total** | | **UC**  **MD_change_** | **UC**  **Total** | | **SMD (MID)** | **95% CI** | | **weight (random,**  **in %)** | |
| --- | --- | --- | --- | --- | --- | --- | --- | --- | --- | --- | --- | --- |
| Bakitas *et al* 2009^55^ | 4.00 | | 108 | | 5.30 | 97 | | -0.26 | -1.51 to 0.99 | | 2.2 | |
| Bakitas *et al* 2020^57^ | 2.40 | | 118 | | 0.70 | 142 | | 0.34 | -0.66 to 1.34 | | 3.2 | |
| Benthien *et al* 2020^59^ | 0.94 | | 119 | | 1.95 | 110 | | -0.18 | -0.93 to 0.57 | | 4.8 | |
| do Carmo *et al* 2017^33^ | -7.14 | | 21 | | -15.79 | 19 | | 1.97 | -2.13 to 6.06 | | 0.2 | |
| Edmonds *et al* 2010^60^ | 0.00 | | 10 | | 0.20 | 15 | | -0.05 | -0.18 to 0.08 | | 15.2 | |
| El-Jahwari *et al* 2016^34^ | -15.97 | | 80 | | -20.70 | 77 | | 1.58 | -0.54 to 3.69 | | 0.8 | |
| El-Jahwari *et al* 2021^35^ | -1.35 | | 78 | | -8.11 | 69 | | 2.70 | -0.58 to 5.98 | | 0.4 | |
| Evans *et al* 2021^61^ | 0.75 | | 23 | | -1.27 | 24 | | 1.01 | 0.32 to 1.70 | | 5.5 | |
| Eychmueller *et al* 2021^40^ | 0.30 | | 61 | | 0.84 | 68 | | -0.11 | -0.64 to 0.42 | | 7.5 | |
| Greer *et al* 2022^36^ | 0.65 | | 54 | | 2.49 | 53 | | -0.26 | -1.26 to 0.73 | | 3.2 | |
| Groenvold *et al* 2017^65^ | -0.30 | | 130 | | 0.80 | 137 | | -0.25 | -1.28 to 0.78 | | 3.0 | |
| Hoek *et al* 2017^66^ | 0.33 | | 22 | | 0.16 | 27 | | 0.03 | -1.62 to 1.68 | | 1.3 | |
| Liu *et al* 2022^32^ | 0.97 | | 83 | | -0.16 | 83 | | 0.26 | -0.35 to 0.87 | | 6.4 | |
| Patil *et al* 2021^67^ | -2.00 | | 77 | | 2.00 | 82 | | -0.67 | -1.69 to 0.36 | | 3.0 | |
| Rogers *et al* 2017^68^ | 13.00 | | 63 | | 17.00 | 60 | | -0.80 | -2.25 to 0.65 | | 1.7 | |
| Sidebottom *et al* 2015^37^ | 12.92 | | 86 | | 8.00 | 89 | | 1.37 | -0.35 to 3.09 | | 1.2 | |
| Tattersall *et al* 2014^46^ | -0.03 | | 58 | | 0.02 | 57 | | -0.02 | -0.14 to 0.10 | | 15.4 | |
| Wong *et al* 2016^53^ | 0.81 | | 43 | | -0.02 | 41 | | 0.59 | 0.24 to 0.95 | | 10.6 | |
| Woo *et al* 2019^48^ | 6.25 | | 112 | | 4.16 | 116 | | 0.48 | -0.81 to 1.76 | | 2.1 | |
| Zimmermann *et al* 2014^49^ | 1.86 | | 154 | | -1.34 | 168 | | 0.36 | 0.09 to 0.63 | | 12.3 | |
|  | |  | |  | | |  | | |  | |  |
| ***Meta-analysis*** | | **SMD (MID)** | | **95% CI** | | | ***t*** | | | ***p*** | |  |
| Random effects model | | 0.16 | | -0.06 to 0.38 | | | 1.56 | | | 0.136 | |  |
|  | |  | |  | | |  | | |  | |  |
| ***Heterogeneity*** | |  | |  | | | ***Q (df)*** | | | ***p*** | |  |
| *τ²* | | 0.06 | | 0.01 to 0.58 | | | 38.32 (19) | | | **0.005** | |  |
| *I²* | | 50.4% | | 17.1 to 70.4% | | |  | | |  | |  |
| *H* | | 1.42 | | 1.10 to 1.84 | | |  | | |  | |  |

**Forest plot**


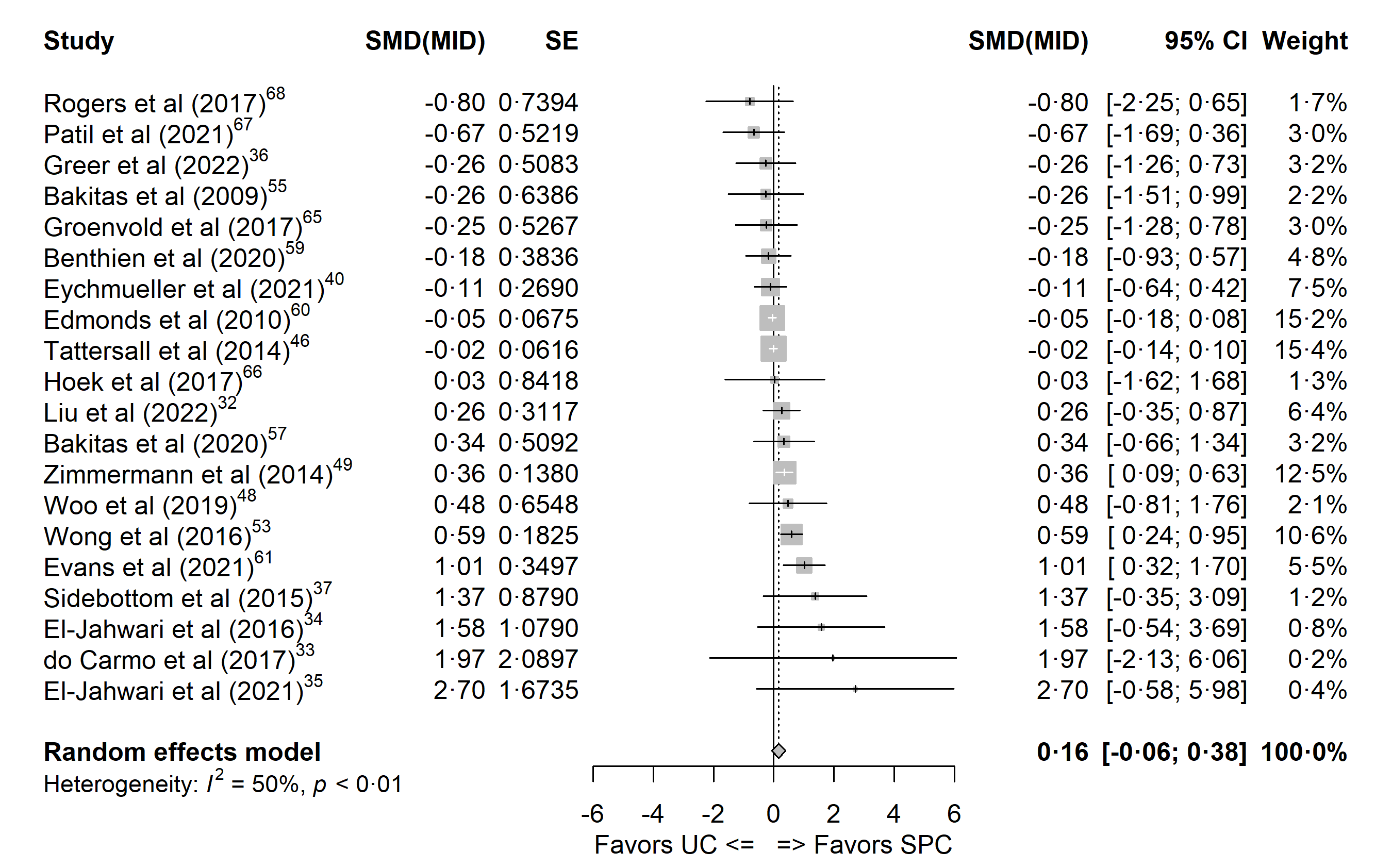


**Publication bias**

Egger’s enhanced funnel plot

| Linear regression test of funnel plot asymmetry  Intercept: 0.643  95% CI: -0.141 to 1.428  *t*(19) = 1.607, *p* = 0.126 | 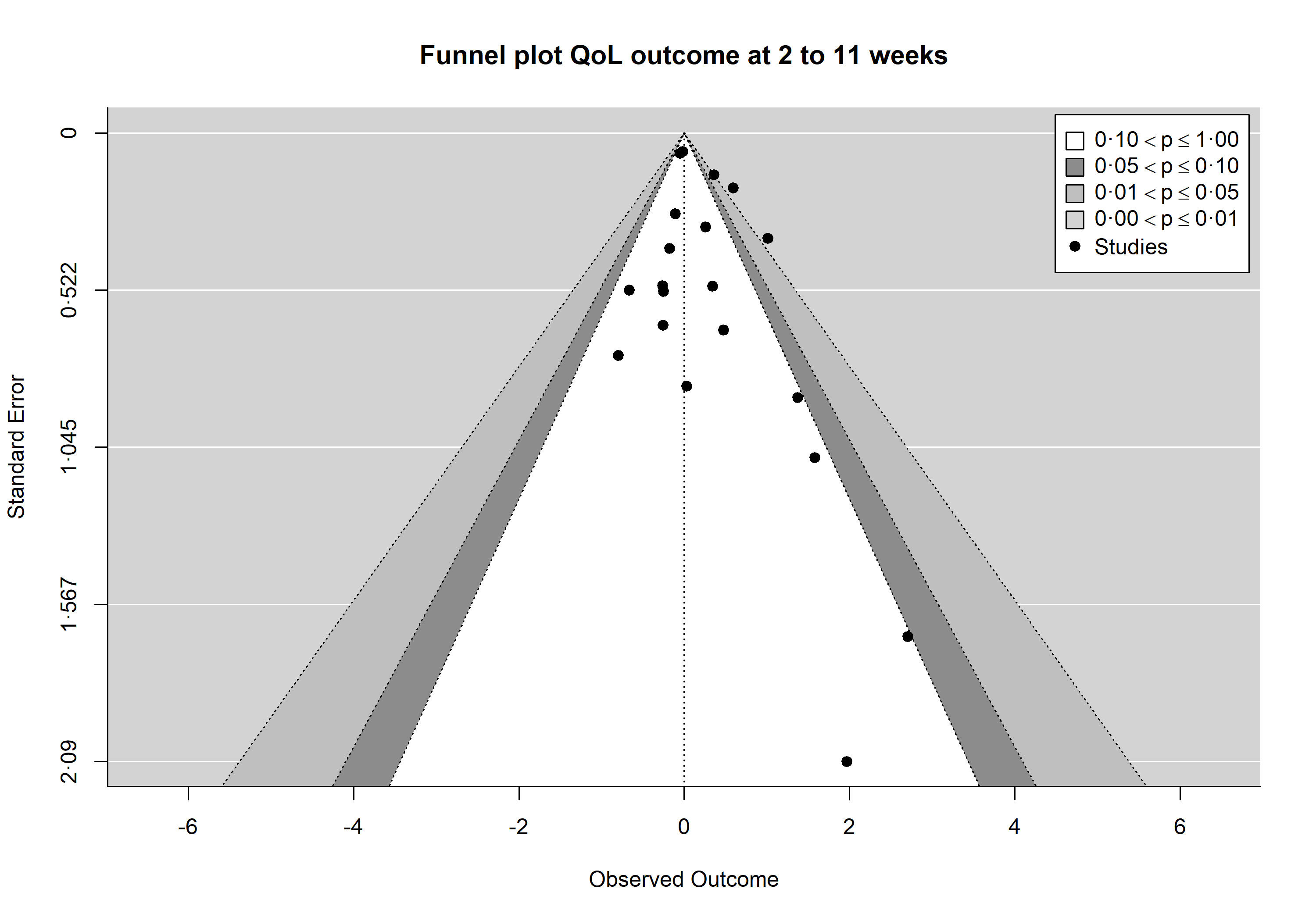 |
| --- | --- |

**Analysis with effect size RR (achieving change ≥1 MID)**

Number of studies combined: k = 20

Number of observations: 3034; Number of events: 1125

| **Study (k = 20)** | **SPC**  **#1MID** | | **SPC**  **Total** | **UC**  **#1MID** | | **UC**  **Total** | **RR**  **(> 1MID)** | | **95% CI** | | **weight (random,**  **in %)** | |
| --- | --- | --- | --- | --- | --- | --- | --- | --- | --- | --- | --- | --- |
| Bakitas *et al* 2009^55^ | 52 | | 108 | 49 | | 97 | 0.95 | | 0.72 to 1.26 | | 9.5 | |
| Bakitas *et al* 2020^57^ | 53 | | 118 | 59 | | 142 | 1.08 | | 0.82 to 1.43 | | 9.4 | |
| Benthien *et al* 2020^59^ | 46 | | 119 | 45 | | 110 | 0.94 | | 0.69 to 1.30 | | 7.2 | |
| do Carmo *et al* 2017^33^ | 7 | | 21 | 5 | | 19 | 1.27 | | 0.48 to 3.33 | | 0.8 | |
| Edmonds *et al* 2010^60^ | 0* | | 10 | 0 | | 15 | 1.50 | | 0.03 to 69.61 | | 0.0 | |
| El-Jahwari *et al* 2016^34^ | 14 | | 80 | 10 | | 77 | 1.35 | | 0.64 to 2.85 | | 1.3 | |
| El-Jahwari *et al* 2021^35^ | 34 | | 78 | 23 | | 69 | 1.31 | | 0.86 to 1.99 | | 4.2 | |
| Evans *et al* 2021^61^ | 6 | | 23 | 3 | | 24 | 2.09 | | 0.59 to 7.38 | | 0.5 | |
| Eychmueller *et al* 2021^40^ | 15 | | 61 | 21 | | 68 | 0.80 | | 0.45 to 1.40 | | 2.3 | |
| Greer *et al* 2022^36^ | 19 | | 54 | 22 | | 53 | 0.85 | | 0.52 to 1.37 | | 3.1 | |
| Groenvold *et al* 2017^65^ | 53 | | 130 | 58 | | 137 | 0.96 | | 0.72 to 1.28 | | 9.0 | |
| Hoek *et al* 2017^66^ | 8 | | 22 | 9 | | 27 | 1.09 | | 0.51 to 2.35 | | 1.2 | |
| Liu *et al* 2022^32^ | 29 | | 83 | 25 | | 83 | 1.16 | | 0.75 to 1.80 | | 3.8 | |
| Patil *et al* 2021^67^ | 24 | | 77 | 35 | | 82 | 0.73 | | 0.48 to 1.11 | | 4.2 | |
| Rogers *et al* 2017^68^ | 41 | | 63 | 44 | | 60 | 0.89 | | 0.70 to 1.12 | | 13.1 | |
| Sidebottom *et al* 2015^37^ | 57 | | 86 | 52 | | 89 | 1.13 | | 0.90 to 1.43 | | 13.7 | |
| Tattersall *et al* 2014^46^ | 0 | | 58 | 0 | | 57 | 0.98 | | 0.02 to 48.69 | | 0.0 | |
| Wong *et al* 2016^53^ | 12 | | 43 | 6 | | 41 | 1.91 | | 0.79 to 4.61 | | 0.9 | |
| Woo *et al* 2019^48^ | 61 | | 112 | 58 | | 116 | 1.09 | | 0.85 to 1.40 | | 11.8 | |
| Zimmermann *et al* 2014^49^ | 42 | | 154 | 26 | | 168 | 1.76 | | 1.14 to 2.73 | | 3.8 | |
|  | |  | | |  | | |  | |  | |  |
| ***Meta-analysis*** | | **RR** | | | **95% CI** | | | ***t*** | | ***p*** | |  |
| Random effects model | | 1.04 | | | 0.95 to 1.14 | | | 0.890 | | 0.384 | |  |
|  | |  | | |  | | |  | |  | |  |
| ***Heterogeneity*** | |  | | |  | | | ***Q (df)*** | | ***p*** | |  |
| *τ²* | | 0.00 | | | 0.00 to 0.06 | | | 18.43 (19) | | 0.494 | |  |
| *I²* | | 0.0% | | | 0.0 to 48.0% | | |  | |  | |  |
| *H* | | 1.00 | | | 1.00 to 1.39 | | |  | |  | |  |

**Forest plot of RR effect size for the QoL outcome 2 to 11 weeks**


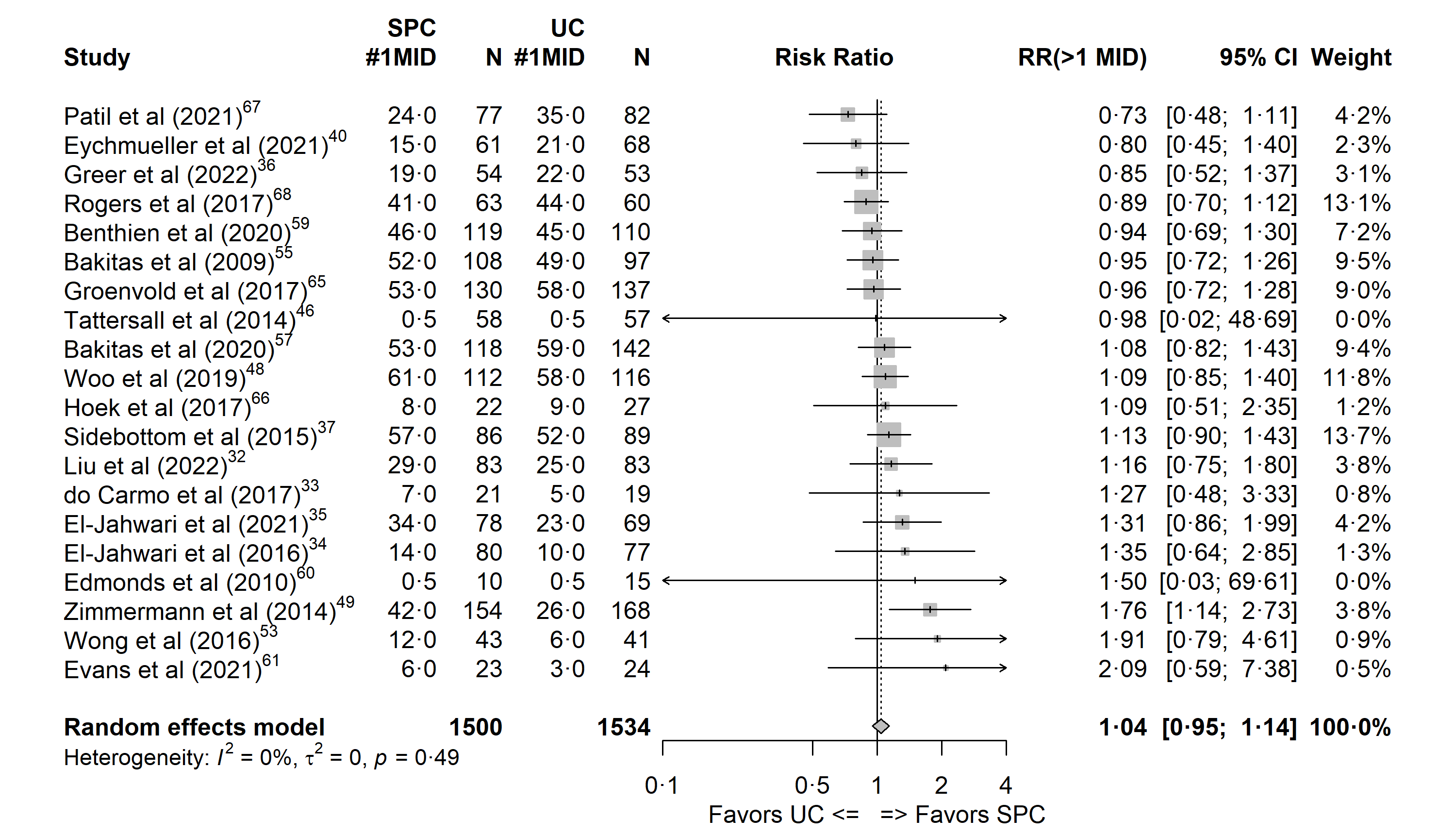


The risk ratio of RR = 1.04 (95% CI = 0.95 to 1.14) translates into a point estimate of a 4% increased probability of experiencing a change in quality of life (QOL) of at least 1 MID size with SPC. This is statistically not significant.

The number needed to treat is calculated as follows (*p_CG_* is the baseline risk of experiencing change of at least 1 MID in the control group):

$$\frac{1}{p_{CG}\cdot(1-RR)}=\frac{1}{\frac{551}{1534}\cdot0.04}=69.601 \to69$$

The NNT is 69, meaning that 69 people need to be treated with SPC in order for one person to have a change in QOL at 2 to 11 weeks of at least 1 MID.

**Meta-regression: Univariate meta-regression analyses with covariates**

| *k = 20* | **Regression** | | | | | **Heterogeneity** | | | **Test of moderators** | |
| --- | --- | --- | --- | --- | --- | --- | --- | --- | --- | --- |
|  | *b* | *SE* | *t* | *p* | 95% CI | *I²* | *Q* | *p* | *F* | *p* |
| ***Attrition (in %)*** |  |  |  |  |  |  |  |  |  |  |
| Intercept | 0.51 | 0.14 | 3.538 | **0.002** | - | 24 | 24.6 | 0.137 | 9.079 | **0.008** |
| Attrition (in %) | -0.01 | 0.00 | -3.013 | **0.008** | -0.02; -0.00 |  |  |  |  |  |
| ***% advanced disease*** |  |  |  |  |  |  |  |  |  |  |
| Intercept | 0.29 | 0.45 | 0.648 | 0.533 | - | 67 | 27.5 | 0.001 | 0.042 | 0.842 |
| % advanced disease | -0.00 | 0.01 | -0.205 | 0.842 | -0.01; 0.01 |  |  |  |  |  |
| ***Disease group (ref: Cancer)*** |  |  |  |  |  |  |  |  |  |  |
| Intercept | 0.06 | 0.13 | 0.440 | 0.665 | - | 50 | 38.2 | 0.004 | 1.751 | 0.202 |
| Non-cancer | 0.28 | 0.22 | 1.323 | 0.202 | -0.17; 0.74 |  |  |  |  |  |
| ***RoB2 score (ref: low risk)*** |  |  |  |  |  |  |  |  |  |  |
| Intercept | 0.54 | 0.25 | 2.194 | **0.042** | - | 46 | 32.0 | 0.015 | 1.426 | 0.268 |
| RoB2: Some risk | -0.46 | 0.29 | -1.598 | 0.128 | -1.08; 0.15 |  |  |  |  |  |
| RoB2: High risk | -0.45 | 0.29 | -1.515 | 0.148 | -1.08; 0.18 |  |  |  |  |  |
| ***Service composition score*** |  |  |  |  |  |  |  |  |  |  |
| Intercept | 0.17 | 0.36 | 0.465 | 0.648 | - | 50 | 37.9 | 0.004 | 0.000 | 0.996 |
| Service composition score | -0.00 | 0.03 | -0.005 | 0.996 | -0.06; 0.06 |  |  |  |  |  |
| ***Setting (ref: multiple settings)*** |  |  |  |  |  |  |  |  |  |  |
| Intercept | -0.01 | 0.17 | 0.042 | 0.967 | - | 53 | 34.5 | 0.007 | 0.930 | 0.414 |
| Inpatient model | 0.44 | 0.35 | 1.266 | 0.223 | -0.29; 1.17 |  |  |  |  |  |
| Home or hospital outreach | 0.22 | 0.23 | 0.929 | 0.366 | -0.28; 0.71 |  |  |  |  |  |
| ***Type of intervention (ref: SPC)*** |  |  |  |  |  |  |  |  |  |  |
| Intercept | 0.20 | 0.16 | 1.217 | 0.241 | - | 48 | 33.1 | 0.007 | 0.814 | 0.505 |
| Early SPC | -0.15 | 0.23 | -0.661 | 0.518 | -0.63; 0.33 |  |  |  |  |  |
| Integrated collaborative care | 0.49 | 0.42 | 1.174 | 0.258 | -0.40; 1.38 |  |  |  |  |  |
| Nurse-led palliative care | -0.10 | 0.49 | -0.207 | 0.839 | -1.13; 0.93 |  |  |  |  |  |
| *k = 20* | **Regression** | | | | | **Heterogeneity** | | | **Test of moderators** | |
|  | *b* | *SE* | *t* | *p* | 95% CI | *I²* | *Q* | *p* | *F* | *p* |
| ***Year*** |  |  |  |  |  |  |  |  |  |  |
| Intercept | -0.03 | 0.44 | -0.067 | 0.948 | - | 57 | 34.2 | 0.012 | 0.200 | 0.660 |
| Year | 0.01 | 0.03 | 0.447 | 0.661 | -0.04; 0.07 |  |  |  |  |  |

.

**Bubble plots of univariate meta-regression analyses**

| **Attrition** | *F*(1,19) = 9.079  *p* = **0.008** | 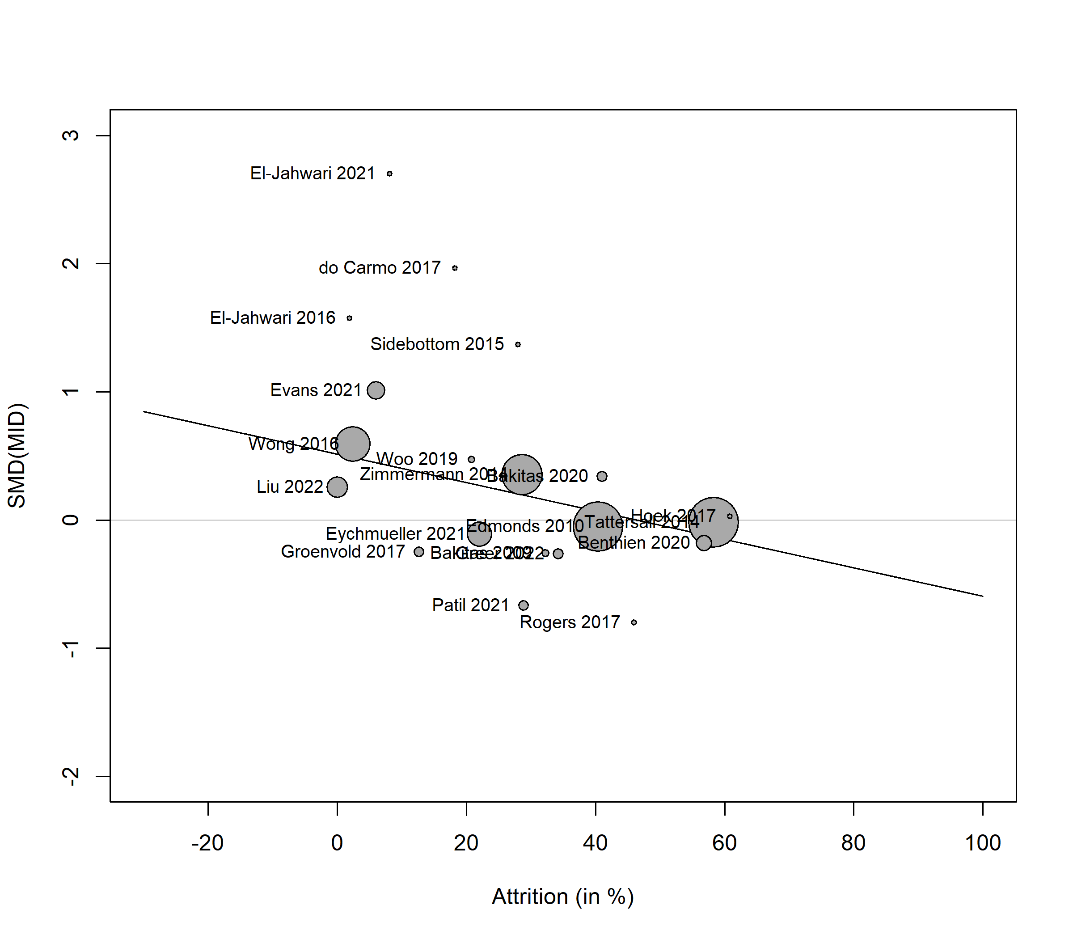 |
| --- | --- | --- |
| **% advanced disease** | *F*(1,9) = 0.042  *p* = 0.842 | 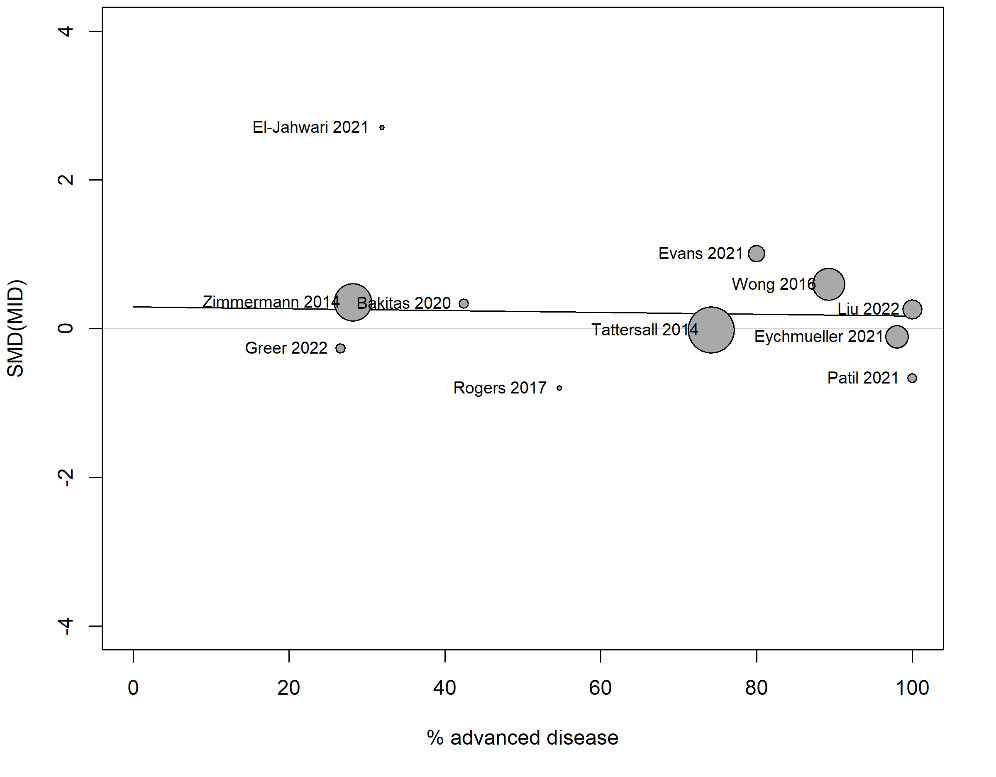 |
| **Disease group** | *F*(1,18) = 1.751  *p* = 0.202 | 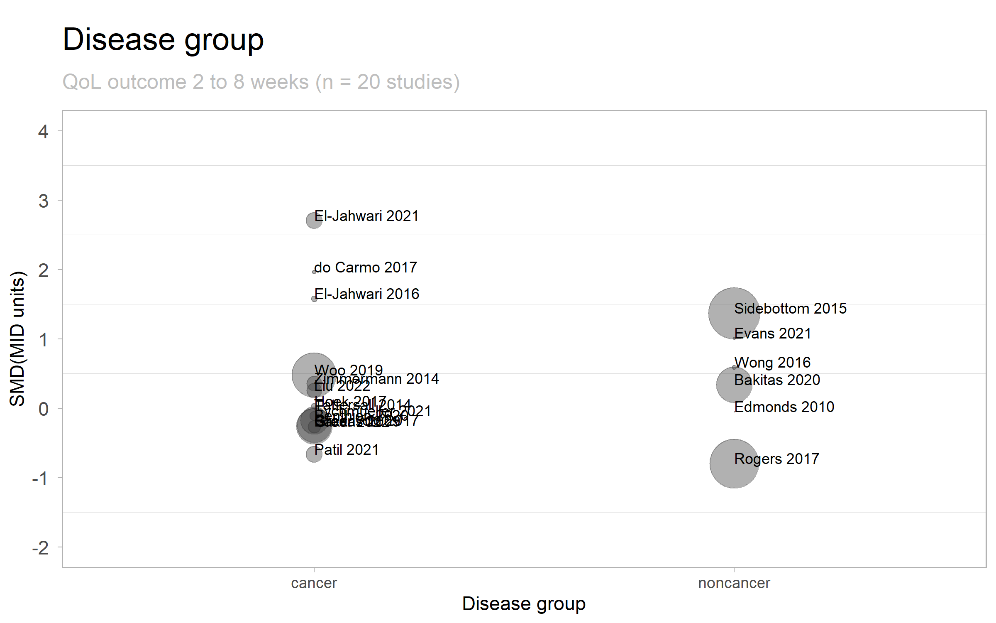 |
| **RoB2 score** | *F*(2,17) = 1.426  *p* = 0.268 | 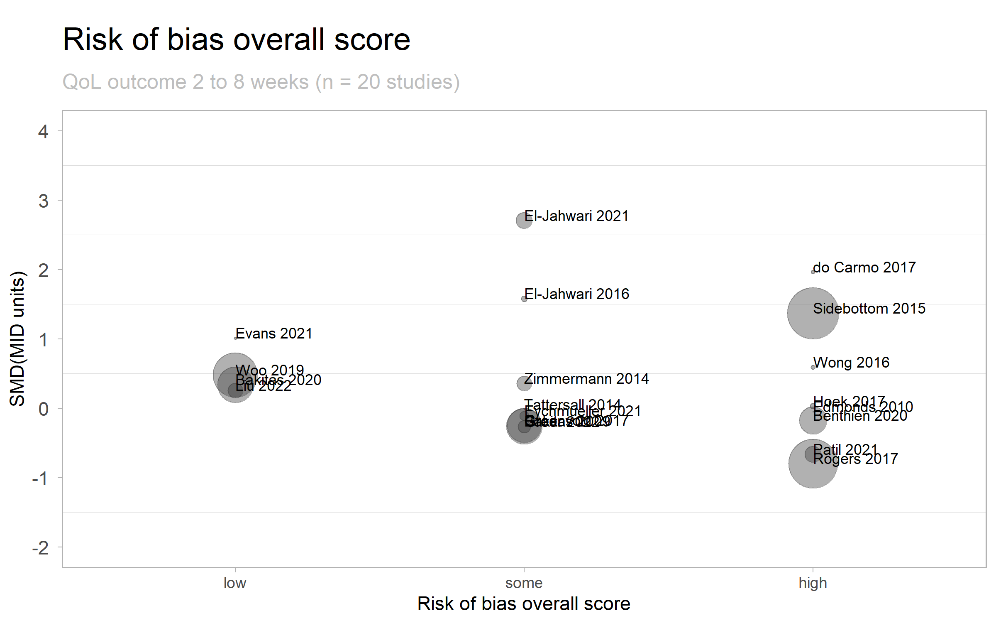 |
| **Service composition score** | *F*(1,18) = 0.000  *p* = 0.996 | 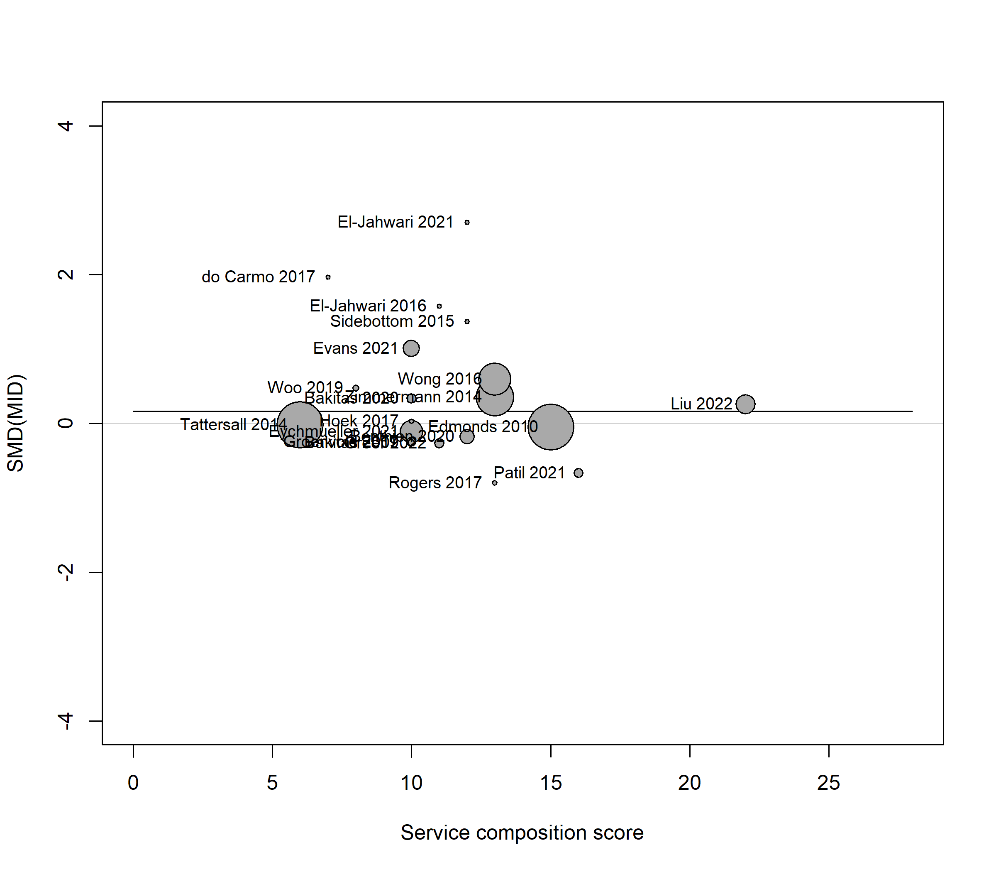 |
| **Setting** | *F*(2,17) = 0.814  *p* = 0.505 | 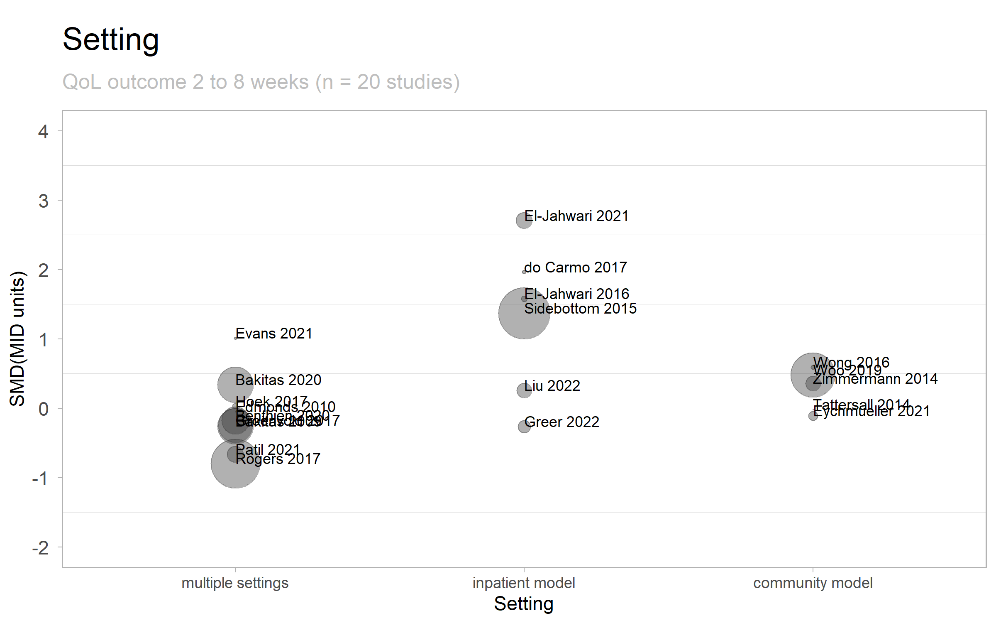 |
| **Type of intervention** | *F*(3,16) = 0.814  *p* = 0.505 | 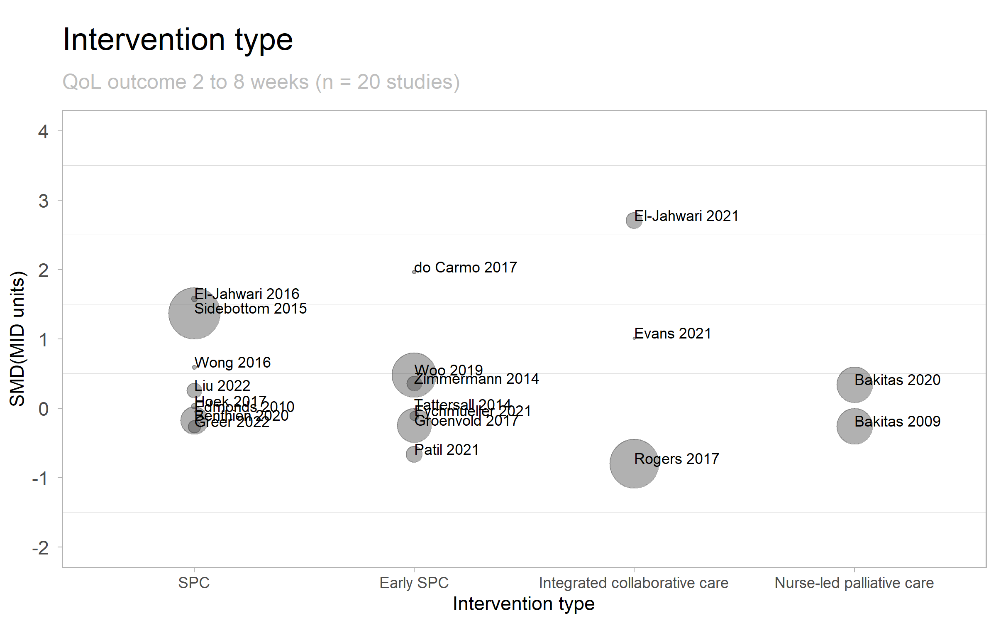 |
| **Year** | *F*(1,18) = 0.200  *p* = 0.660 | 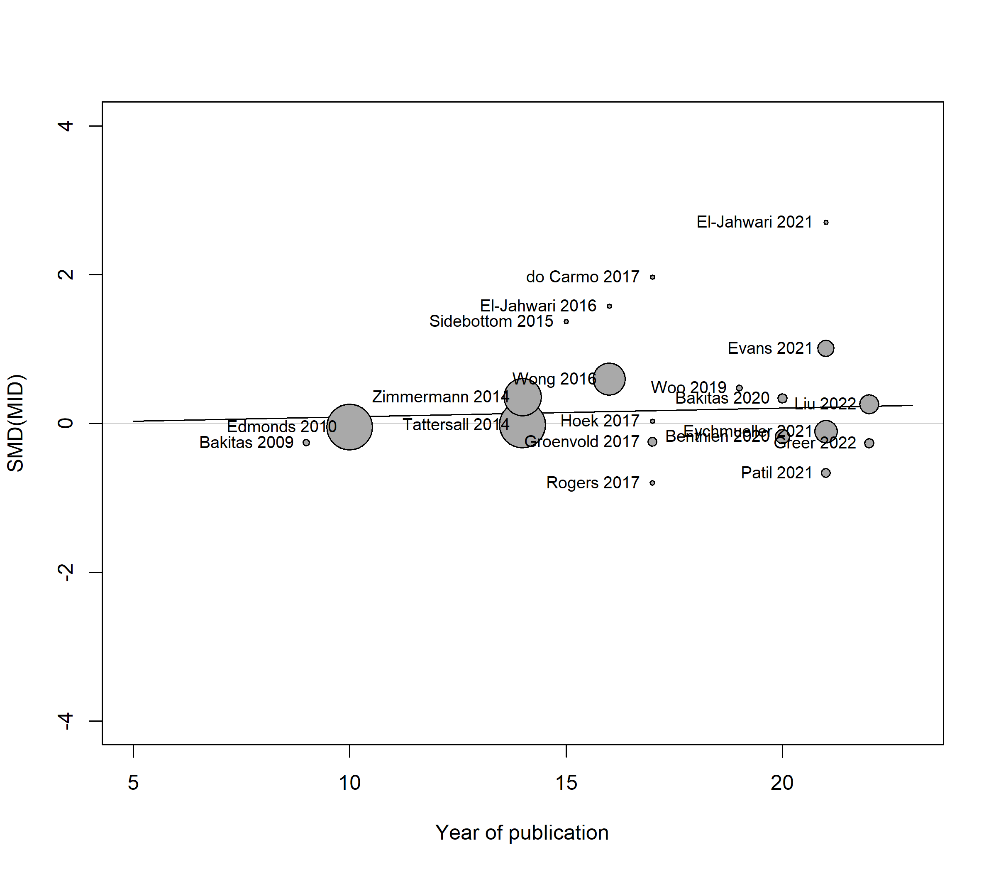 |

## Fig B and Table B: Quality of life at 12 weeks

**Analysis with effect size SMD (MID units)**

| **Study (k = 27)** | **SPC**  **MD_change_** | | **SPC**  **Total** | | **UC**  **MD_change_** | **UC**  **Total** | | **SMD (MID)** | **95% CI** | | **weight**  **(random,**  **in %)** | |
| --- | --- | --- | --- | --- | --- | --- | --- | --- | --- | --- | --- | --- |
| Bakitas *et al* 2015^56^ | 2.99 | | 72 | | 3.61 | 83 | | -0.07 | -0.67 to 0.54 | | 4.8 | |
| Bekelman *et al* 2018^58^ | 4.80 | | 106 | | 3.00 | 108 | | 0.36 | -0.69 to 1.41 | | 4.1 | |
| Brims *et al* 2019^51^ | -4.80 | | 75 | | -6.50 | 73 | | 0.39 | -1.47 to 2.24 | | 2.8 | |
| do Carmo *et al* 2017^33^ | -7.02 | | 19 | | -12.04 | 18 | | 1.14 | -1.63 to 3.91 | | 1.8 | |
| Edmonds *et al* 2010^60^ | 0.30 | | 16 | | 7.10 | 7 | | -0.85 | -3.09 to 1.39 | | 2.3 | |
| El-Jahwari *et al* 2016^34^ | 1.70 | | 75 | | -0.64 | 74 | | 0.78 | -1.19 to 2.75 | | 2.6 | |
| El-Jahwari *et al* 2021^35^ | 11.70 | | 72 | | 8.30 | 65 | | 1.36 | -1.51 to 4.23 | | 1.7 | |
| Evans *et al* 2021^61^ | 0.88 | | 23 | | -0.54 | 24 | | 0.71 | 0.12 to 1.30 | | 4.8 | |
| Franciosi *et al* 2019^62^ | -0.60 | | 111 | | -1.70 | 103 | | 0.22 | -0.57 to 1.01 | | 4.5 | |
| Gao *et al* 2020^63^ | 0.78 | | 176 | | 0.28 | 174 | | 0.46 | -0.22 to 1.13 | | 4.7 | |
| Given *et al* 2002^64^ | 2.10 | | 53 | | 0.70 | 60 | | 0.28 | 0.07 to 0.49 | | 5.1 | |
| Goldstein *et al* 2022^52^ | 10.30 | | 68 | | -2.55 | 68 | | 4.28 | 4.01 to 4.55 | | 5.1 | |
| Greer *et al* 2022^36^ | -0.33 | | 48 | | 2.87 | 47 | | -0.46 | -1.55 to 0.64 | | 4.0 | |
| Hoek *et al* 2017^66^ | -5.61 | | 13 | | 1.95 | 16 | | -1.33 | -3.45 to 0.80 | | 2.4 | |
| Maltoni *et al* 2016^42^ | -0.92 | | 64 | | -4.49 | 65 | | 0.45 | -0.22 to 1.11 | | 4.7 | |
| Nottelmann *et al* 2021^43^ | 3.30 | | 133 | | 3.30 | 146 | | 0.00 | -1.58 to 1.58 | | 3.2 | |
| Patil *et al* 2021^67^ | -2.10 | | 67 | | 3.40 | 61 | | -0.92 | -2.07 to 0.24 | | 3.9 | |
| Rogers *et al* 2017^68^ | 22.40 | | 47 | | 22.40 | 43 | | 0.00 | -1.78 to 1.78 | | 2.9 | |
| Scarpi *et al* 2019^44^ | 1.65 | | 66 | | -1.30 | 65 | | 0.49 | -0.72 to 1.70 | | 3.8 | |
| Sidebottom *et al* 2015^37^ | 14.86 | | 79 | | 11.80 | 88 | | 0.85 | -0.89 to 2.59 | | 2.9 | |
| Slama *et al* 2020^45^ | 3.30 | | 51 | | 4.80 | 51 | | -0.34 | -2.05 to 1.37 | | 3.0 | |
| Tattersall *et al* 2014^46^ | -0.28 | | 49 | | 0.02 | 57 | | -0.10 | -0.23 to 0.03 | | 5.2 | |
| Temel *et al* 2010^47^ | 4.40 | | 60 | | -0.20 | 47 | | 1.92 | -0.64 to 4.47 | | 2.0 | |
| Temel *et al* 2020^69^ | 3.35 | | 92 | | 0.12 | 101 | | 0.65 | -0.13 to 1.42 | | 4.5 | |
| Vanbutsele *et al* 2020^70^ | 2.60 | | 91 | | -5.40 | 94 | | 1.82 | 0.45 to 3.19 | | 3.5 | |
| Woo *et al* 2019^48^ | 1.90 | | 72 | | 1.20 | 78 | | 0.35 | -0.12 to 0.82 | | 4.9 | |
| Zimmermann *et al* 2014^49^ | 1.60 | | 140 | | -2.00 | 141 | | 0.40 | 0.04 to 0.76 | | 5.0 | |
|  | |  | |  | | |  | | |  | |  |
| ***Meta-analysis*** | | **SMD (MID)** | | **95% CI** | | | ***t*** | | | ***p*** | |  |
| Random effects model | | 0.50 | | 0.06 to 0.93 | | | 2.330 | | | **0.028** | |  |
|  | |  | |  | | |  | | |  | |  |
| ***Heterogeneity*** | |  | |  | | | ***Q (df)*** | | | ***p*** | |  |
| *τ²* | | 1.03 | | 0.47 to 1.77 | | | 871.68 (26) | | | **<0.001** | |  |
| *I²* | | 97% | | 96.4 to 97.6% | | |  | | |  | |  |
| *H* | | 5.79 | | 5.24 to 6.40 | | |  | | |  | |  |

**Forest plot**


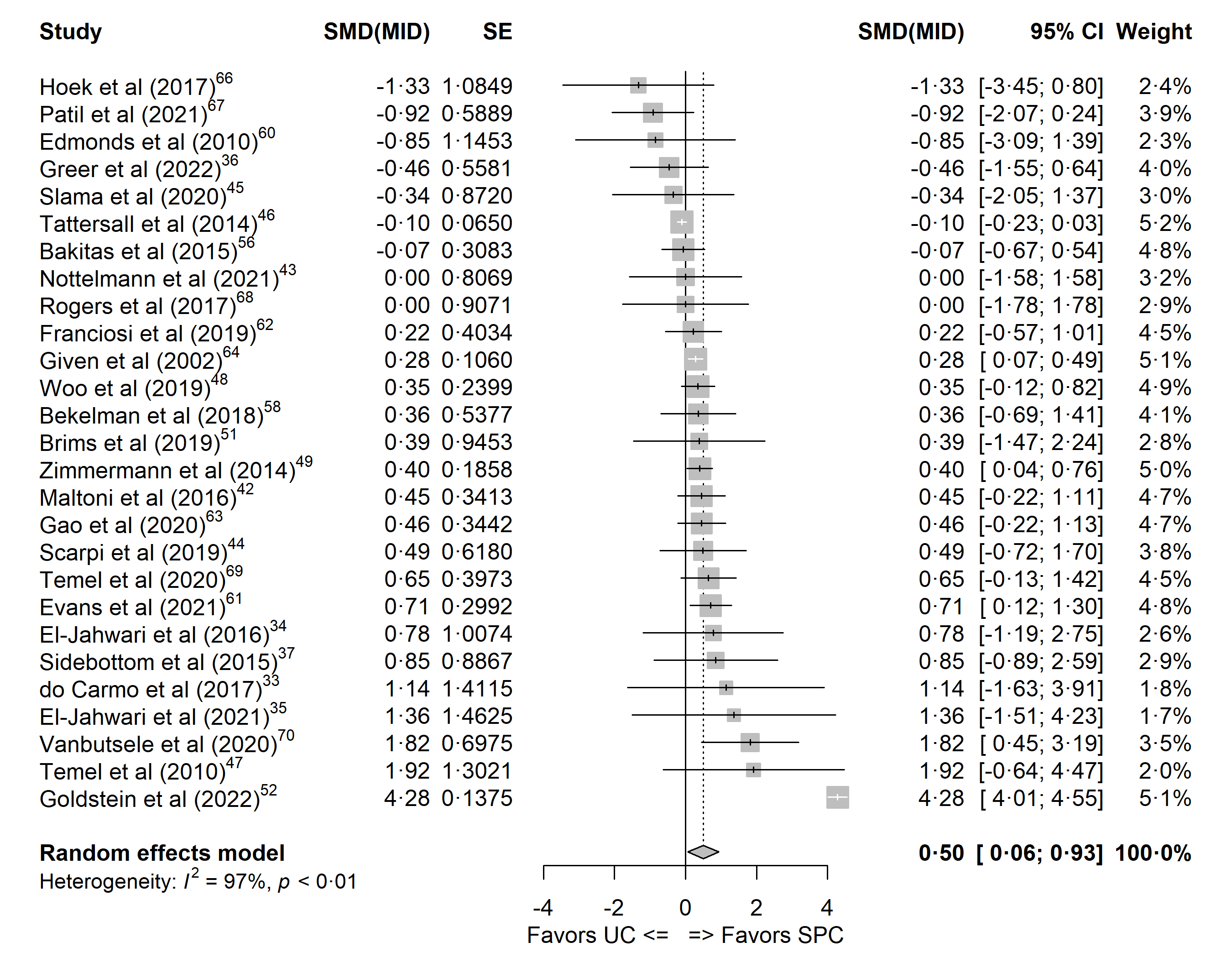


**Publication bias**

Egger’s enhanced funnel plot

| Linear regression test of funnel plot asymmetry  Intercept: 0.362  95% CI: -2.640 to 3.364  *t*(26) = 0.236, *p* = 0.815 | 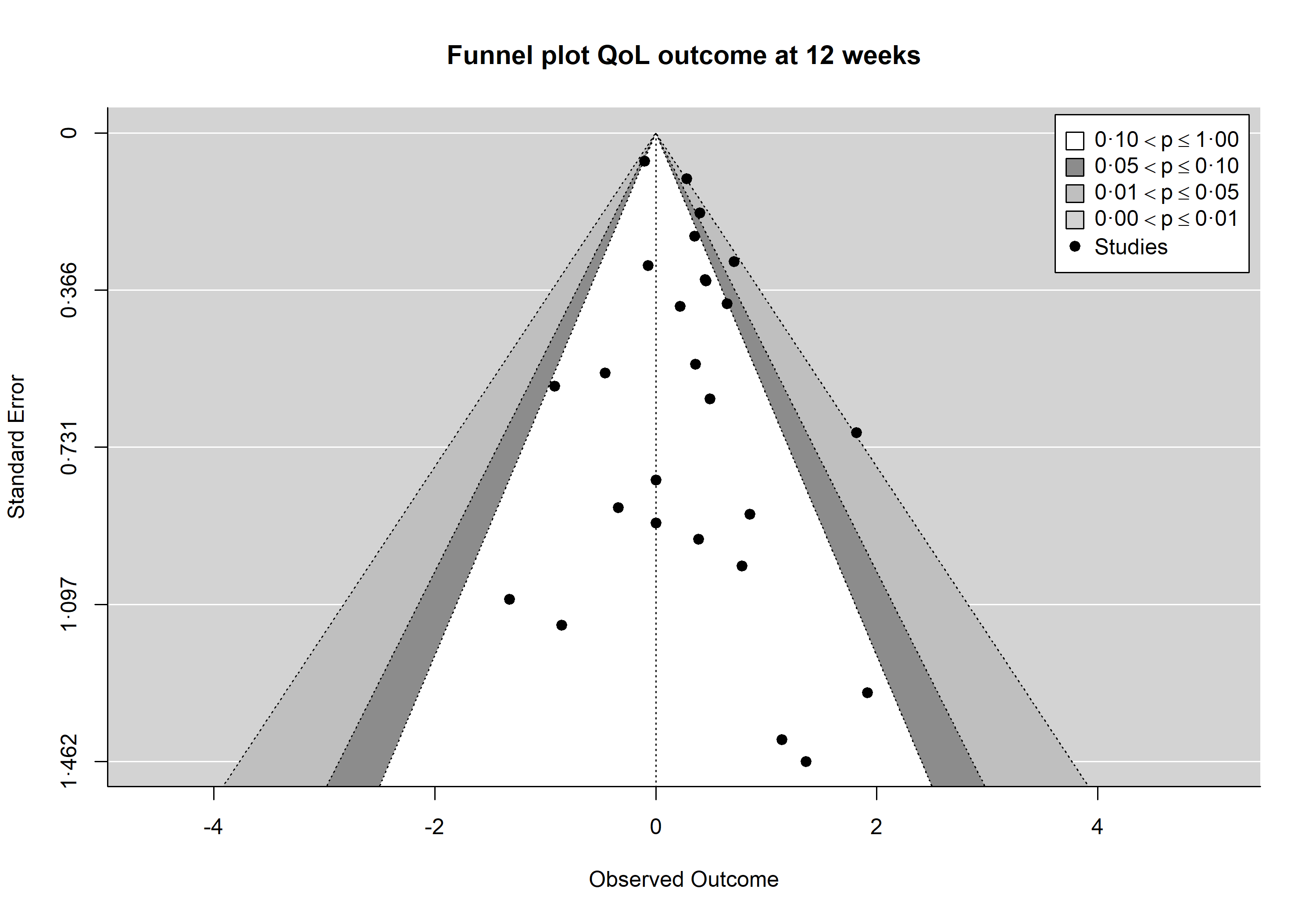 |
| --- | --- |

**Analysis with effect size RR (achieving change ≥1 MID)**

Number of studies combined: k = 27

Number of observations: 3895; Number of events: 1588

| **Study (k = 27)** | **SPC**  **#1MID** | | **SPC**  **Total** | **UC**  **#1MID** | | **UC**  **Total** | **RR**  **(> 1MID)** | | **95% CI** | | **weight (random,**  **in %)** | |
| --- | --- | --- | --- | --- | --- | --- | --- | --- | --- | --- | --- | --- |
| Bakitas *et al* 2015^56^ | 26 | | 72 | 31 | | 83 | 0.97 | | 0.64 to 1.46 | | 4.3 | |
| Bekelman *et al* 2018^58^ | 53 | | 106 | 50 | | 108 | 1.08 | | 0.82 to 1.43 | | 5.0 | |
| Brims *et al* 2019^51^ | 27 | | 75 | 25 | | 73 | 1.05 | | 0.68 to 1.63 | | 4.1 | |
| do Carmo *et al* 2017^33^ | 5 | | 19 | 4 | | 18 | 1.18 | | 0.38 to 3.72 | | 1.6 | |
| Edmonds *et al* 2010^60^ | 5 | | 16 | 3 | | 7 | 0.73 | | 0.24 to 2.24 | | 1.6 | |
| El-Jahwari *et al* 2016^34^ | 35 | | 75 | 31 | | 74 | 1.11 | | 0.78 to 1.60 | | 4.5 | |
| El-Jahwari *et al* 2021^35^ | 48 | | 72 | 39 | | 65 | 1.11 | | 0.86 to 1.44 | | 5.1 | |
| Evans *et al* 2021^61^ | 6 | | 23 | 3 | | 24 | 2.09 | | 0.59 to 7.38 | | 1.4 | |
| Franciosi *et al* 2019^62^ | 39 | | 111 | 34 | | 103 | 1.06 | | 0.73 to 1.55 | | 4.5 | |
| Gao *et al* 2020^63^ | 81 | | 176 | 71 | | 174 | 1.13 | | 0.89 to 1.43 | | 5.1 | |
| Given *et al* 2002^64^ | 8 | | 53 | 4 | | 60 | 2.26 | | 0.72 to 7.09 | | 1.6 | |
| Goldstein *et al* 2022^52^ | 68 | | 68 | 2 | | 68 | 27.40 | | 8.12 to 92.51 | | 1.5 | |
| Greer *et al* 2022^36^ | 16 | | 48 | 20 | | 47 | 0.78 | | 0.47 to 1.32 | | 3.7 | |
| Hoek *et al* 2017^66^ | 4 | | 13 | 6 | | 16 | 0.82 | | 0.29 to 2.30 | | 1.8 | |
| Maltoni *et al* 2016^42^ | 16 | | 64 | 16 | | 65 | 1.02 | | 0.56 to 1.85 | | 3.3 | |
| Nottelmann *et al* 2021^43^ | 64 | | 133 | 71 | | 146 | 0.99 | | 0.78 to 1.26 | | 5.1 | |
| Patil *et al* 2021^67^ | 21 | | 67 | 28 | | 61 | 0.68 | | 0.44 to 1.07 | | 4.1 | |
| Rogers *et al* 2017^68^ | 36 | | 47 | 35 | | 43 | 0.94 | | 0.76 to 1.16 | | 5.3 | |
| Scarpi *et al* 2019^44^ | 28 | | 66 | 23 | | 65 | 1.20 | | 0.78 to 1.85 | | 4.2 | |
| Sidebottom *et al* 2015^37^ | 55 | | 79 | 58 | | 88 | 1.06 | | 0.86 to 1.30 | | 5.3 | |
| Slama *et al* 2020^45^ | 24 | | 51 | 26 | | 51 | 0.92 | | 0.62 to 1.37 | | 4.4 | |
| Tattersall *et al* 2014^46^ | 0.5 | | 49 | 0.5 | | 57 | 1.16 | | 0.02 to 57.54 | | 0.2 | |
| Temel *et al* 2010^47^ | 33 | | 60 | 21 | | 47 | 1.23 | | 0.83 to 1.82 | | 4.4 | |
| Temel *et al* 2020^69^ | 42 | | 92 | 35 | | 101 | 1.32 | | 0.93 to 1.87 | | 4.6 | |
| Vanbutsele *et al* 2020^70^ | 42 | | 91 | 30 | | 94 | 1.45 | | 1.00 to 2.09 | | 4.5 | |
| Woo *et al* 2019^48^ | 34 | | 72 | 33 | | 78 | 1.12 | | 0.78 to 1.59 | | 4.6 | |
| Zimmermann *et al* 2014^49^ | 43 | | 140 | 29 | | 141 | 1.49 | | 0.99 to 2.25 | | 4.3 | |
|  | |  | | |  | | |  | |  | |  |
| ***Meta-analysis*** | | **RR** | | | **95% CI** | | | ***t*** | | ***p*** | |  |
| Random effects model | | 1.14 | | | 0.95 to 1.36 | | | 1.490 | | 0.149 | |  |
|  | |  | | |  | | |  | |  | |  |
| ***Heterogeneity*** | |  | | |  | | | ***Q (df)*** | | ***p*** | |  |
| *τ²* | | 0.13 | | | 0.01 to 0.51 | | | 46.08 (26) | | **0.009** | |  |
| *I²* | | 43.6% | | | 11.0 to 64.2% | | |  | |  | |  |
| *H* | | 1.33 | | | 1.06 to 1.67 | | |  | |  | |  |

**Forest plot of RR effect size for the QoL outcome at 12 weeks**


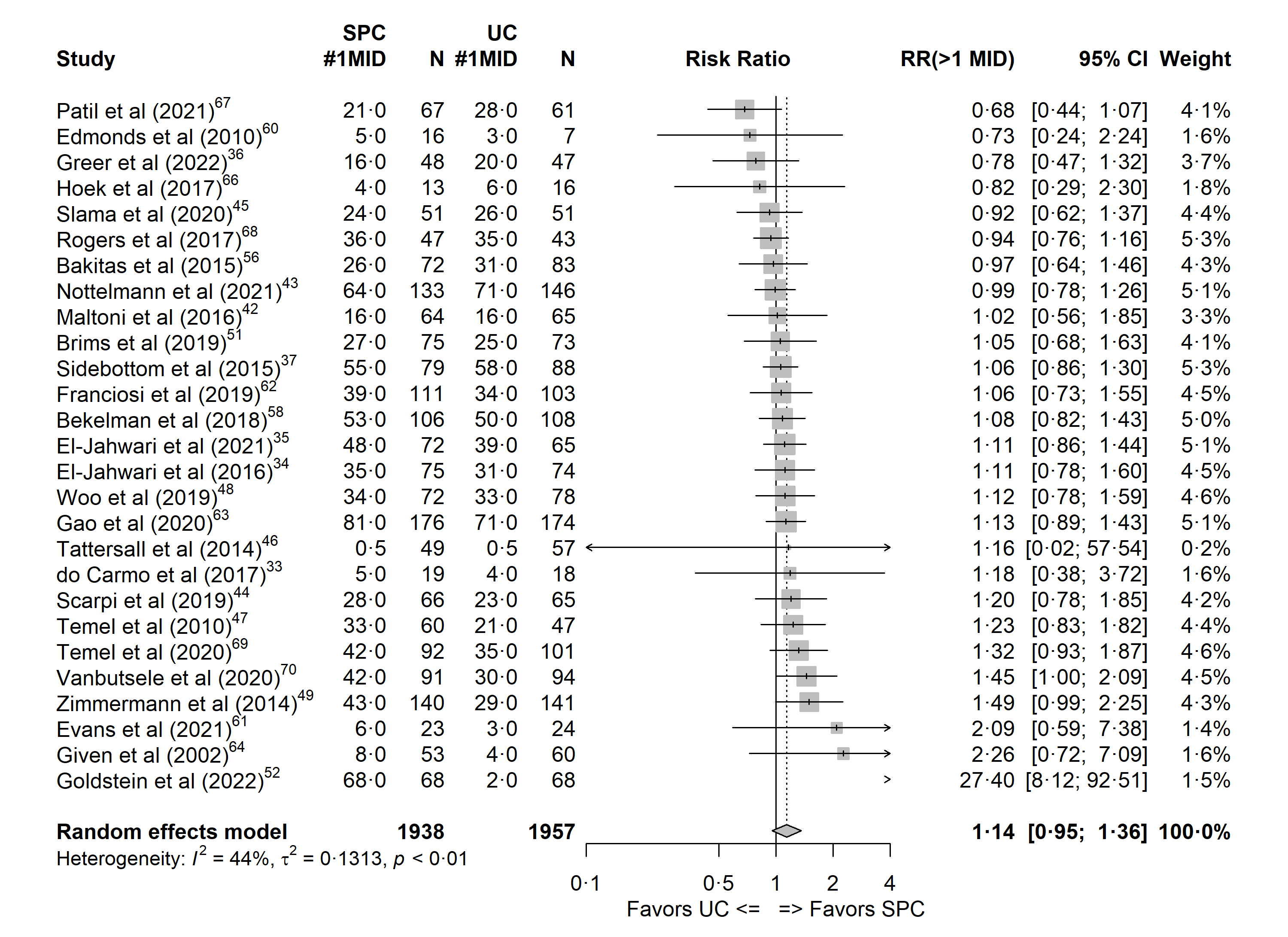


The risk ratio of RR = 1.14 (95% CI = 0.95 to 1.36) translates into a point estimate of a 14% increased probability of experiencing a change in QOL of at least 1 MID size with SPC. This is statistically not significant.

The number needed to treat is calculated as follows (*p_CG_* is the baseline risk of experiencing change of at least 1 MID in the control group):

$$\frac{1}{p_{CG}\cdot(1-RR)}=\frac{1}{\frac{729}{1588}\cdot0.14}=15.559 \to15$$

The NNT is 15, meaning that 15 people need to be treated with SPC in order for one person to have a change in QOL at 12 weeks of at least 1 MID.

**Meta-regression: Univariate meta-regression analyses with covariates**

| *k = 27* | **Regression** | | | | | **Heterogeneity** | | | **Test of moderators** | |
| --- | --- | --- | --- | --- | --- | --- | --- | --- | --- | --- |
|  | *b* | *SE* | *t* | *p* | 95% CI | *I²* | *Q* | *p* | *F* | *p* |
| ***Attrition (in %)*** |  |  |  |  |  |  |  |  |  |  |
| Intercept | 0.47 | 0.12 | 4.053 | **0.001** | - | 9.9 | 24.1 | 0.454 | 7.955 | **0.010** |
| Attrition (in %) | -0.01 | 0.00 | -2.788 | **0.010** | -0.02; -0.00 |  |  |  |  |  |
| ***% advanced disease*** |  |  |  |  |  |  |  |  |  |  |
| Intercept | 0.34 | 0.29 | 1.155 | 0.269 | - | 43 | 22.2 | 0.052 | 0.459 | 0.510 |
| % advanced disease | -0.00 | 0.00 | -0.678 | 0.510 | -0.01; 0.01 |  |  |  |  |  |
| ***Disease group (ref: Cancer)*** |  |  |  |  |  |  |  |  |  |  |
| Intercept | 0.27 | 0.24 | 1.133 | 0.268 | - | 92 | 287.8 | **0.000** | 3.488 | *0.074* |
| Non-cancer | 0.86 | 0.46 | 1.868 | 0.074 | -0.09; 1.81 |  |  |  |  |  |
| ***RoB2 score (ref: low risk)*** |  |  |  |  |  |  |  |  |  |  |
| Intercept | 0.62 | 0.49 | 1.260 | 0.290 | - | 91 | 857.7 | **0.000** | 1.369 | 0.274 |
| RoB2: Some risk | 0.15 | 0.58 | 0.263 | 0.795 | -1.04; 1.34 |  |  |  |  |  |
| RoB2: High risk | -0.62 | 0.62 | -1.005 | 0.325 | -1.89; 0.65 |  |  |  |  |  |
| *k = 27* | **Regression** | | | | | **Heterogeneity** | | | **Test of moderators** | |
|  | *b* | *SE* | *t* | *p* | 95% CI | *I²* | *Q* | *p* | *F* | *p* |
| ***Service composition score*** |  |  |  |  |  |  |  |  |  |  |
| Intercept | -0.03 | 0.79 | -0.041 | 0.968 | - | 92 | 468.0 | **0.000** | 0.479 | 0.495 |
| Service composition score | 0.05 | 0.07 | 0.692 | 0.495 | -0.09; 0.18 |  |  |  |  |  |
| ***Setting (ref: multiple settings)*** |  |  |  |  |  |  |  |  |  |  |
| Intercept | 0.20 | 0.31 | 0.657 | 0.517 | - | 92 | 858.6 | **0.000** | 0.930 | 0.408 |
| Inpatient consulting model | 0.34 | 0.67 | 0.508 | 0.616 | -1.04; 1.71 |  |  |  |  |  |
| Home or hospital outreach | 0.62 | 0.46 | 1.361 | 0.186 | -0.32; 1.57 |  |  |  |  |  |
| ***Type of intervention (ref: SPC)*** |  |  |  |  |  |  |  |  |  |  |
| Intercept | 1.00 | 0.50 | 2.013 | 0.056 | - | 89 | 158.2 | **0.000** | 0.499 | 0.686 |
| Early SPC | -0.63 | 0.58 | -1.077 | 0.293 | -1.83; 0.58 |  |  |  |  |  |
| Integrated collaborative care | -0.49 | 0.72 | -0.682 | 0.502 | -1.97; 0.99 |  |  |  |  |  |
| Nurse-led palliative care | -0.89 | 0.86 | -1.039 | 0.309 | -2.66; 0.88 |  |  |  |  |  |
| ***Year*** |  |  |  |  |  |  |  |  |  |  |
| Intercept | -0.26 | 0.82 | -0.320 | 0.752 | - | 92 | 700.4 | **0.000** | 0.914 | 0.348 |
| Year | 0.04 | 0.05 | 0.956 | 0.348 | -0.05; 0.14 |  |  |  |  |  |

.

**Bubble plots of univariate meta-regression analyses**

| **Attrition** | *F*(1,24) = 7.955  *p* = **0.010** | 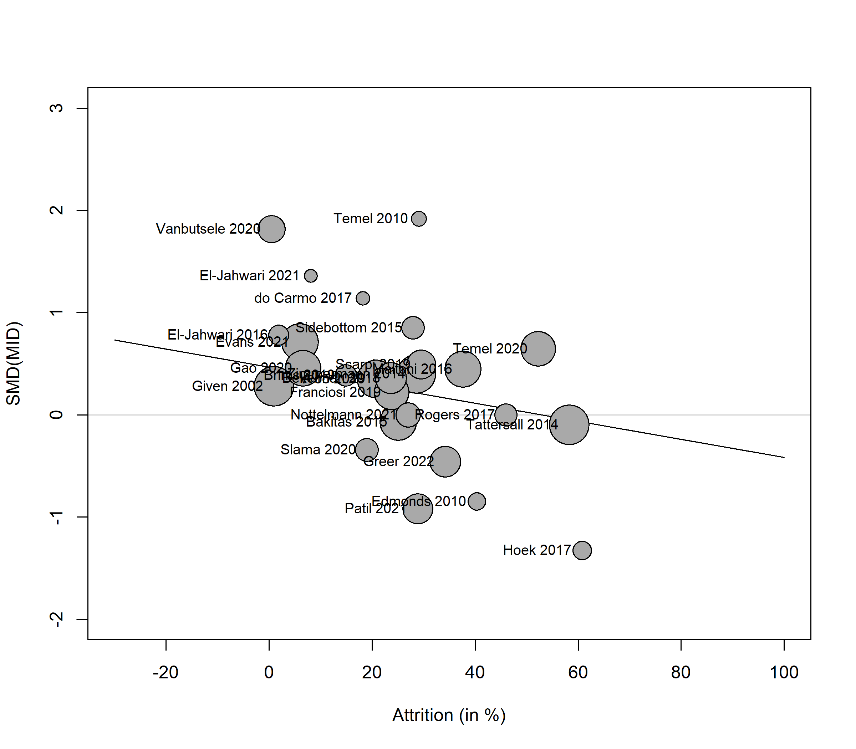 |
| --- | --- | --- |
| **% advanced disease** | *F*(1,13) = 0.459  *p* = 0.510 | 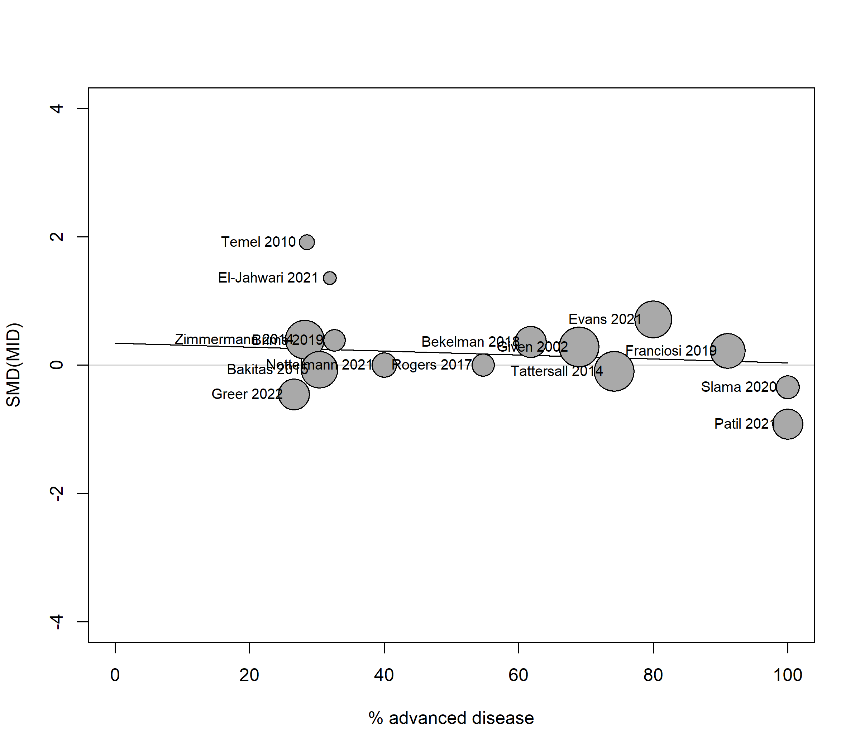 |
| **Disease group** | *F*(1,25) = 3.488  *p* = *0.074* | 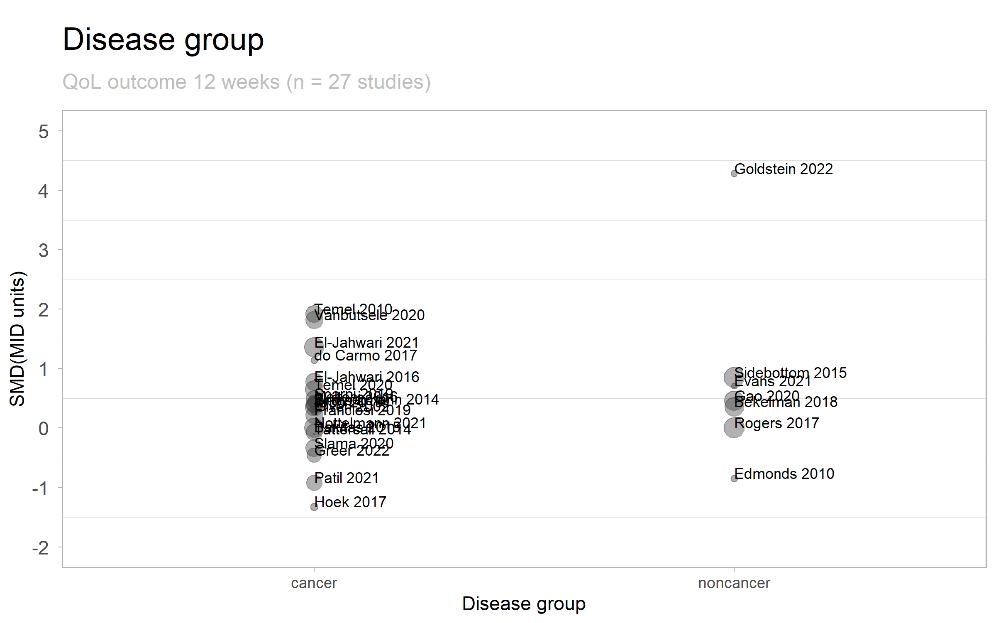 |
| **RoB2 score** | *F*(2,24) = 1.369  *p* = 0.274 | 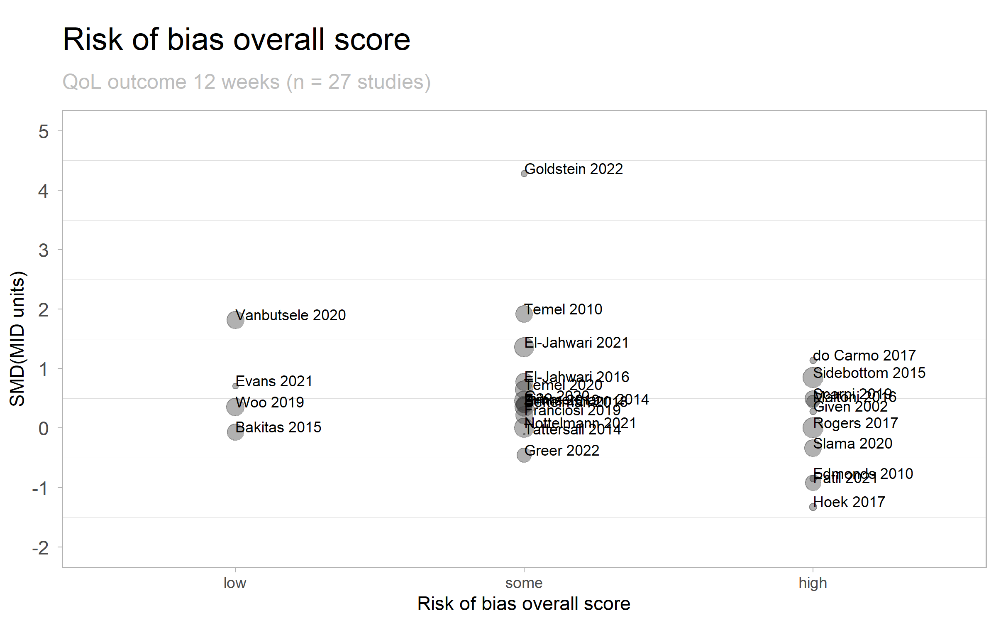 |
| **Service composition score** | *F*(1,25) = 0.479  *p* = 0.495 | 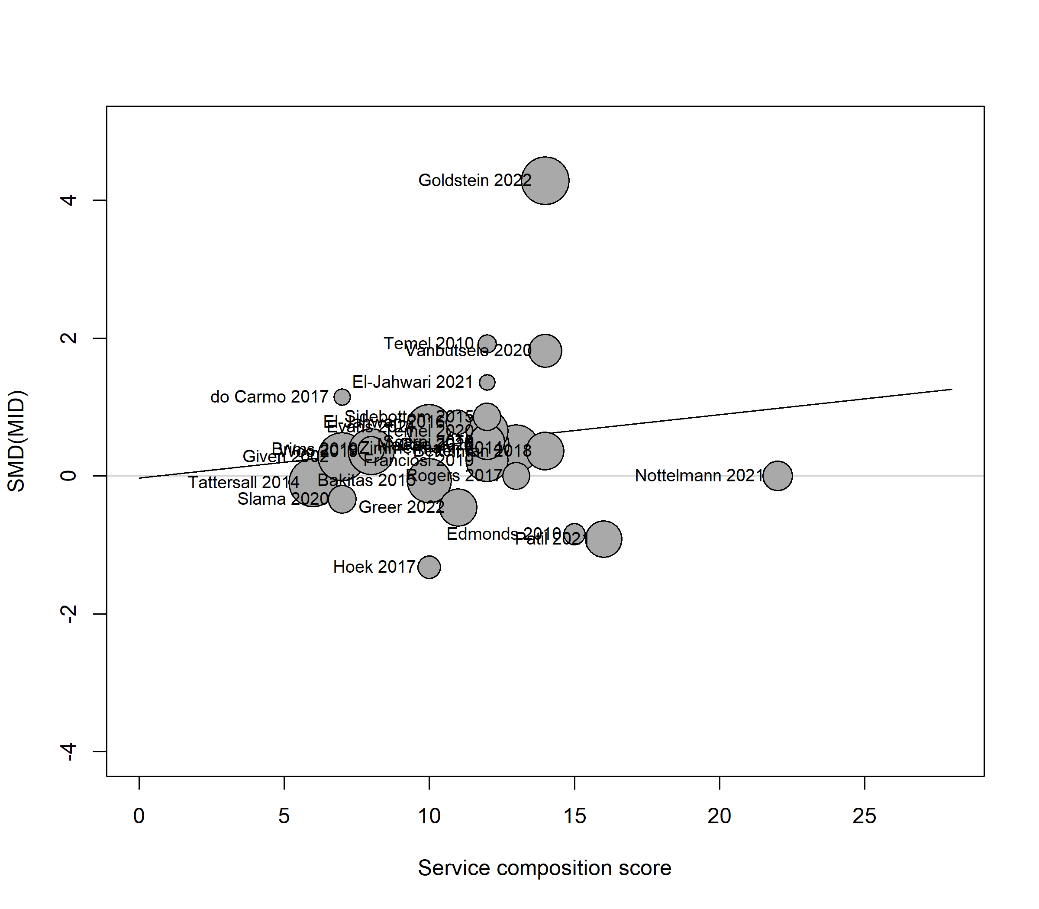 |
| **Setting** | *F*(2,24) = 0.930  *p* = 0.408 | 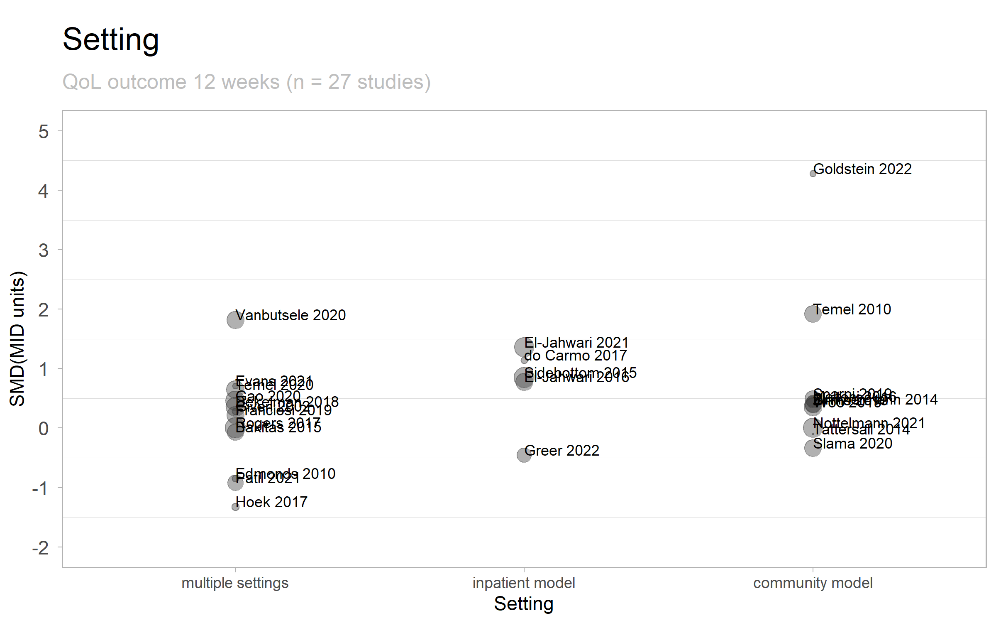 |
| **Type of intervention** | *F*(2,22) = 0.499  *p* = 0.686 | 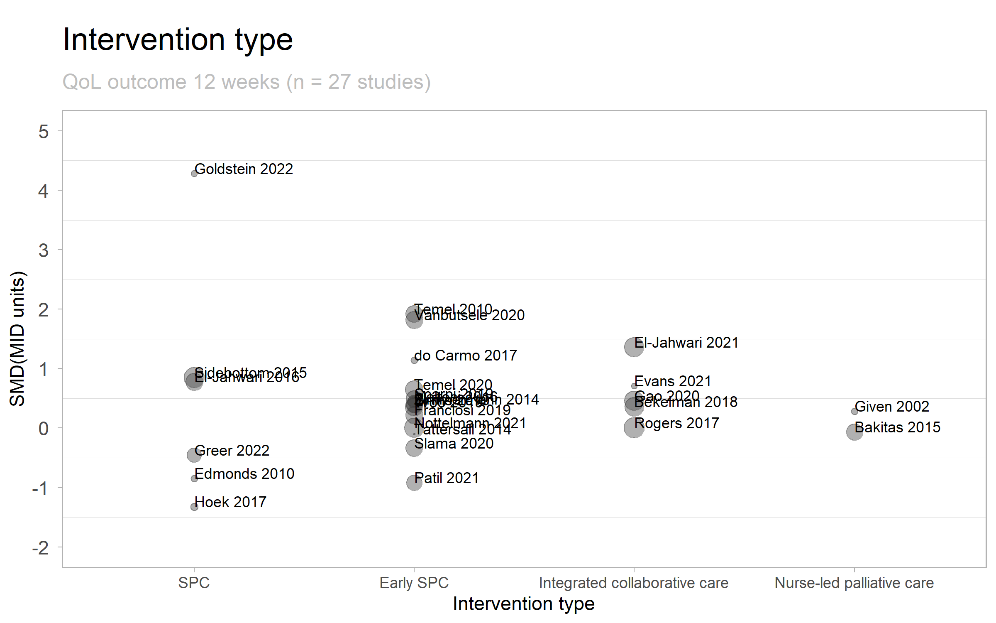 |
| **Year** | *F*(1,25) = 0.914  *p* = 0.348 | 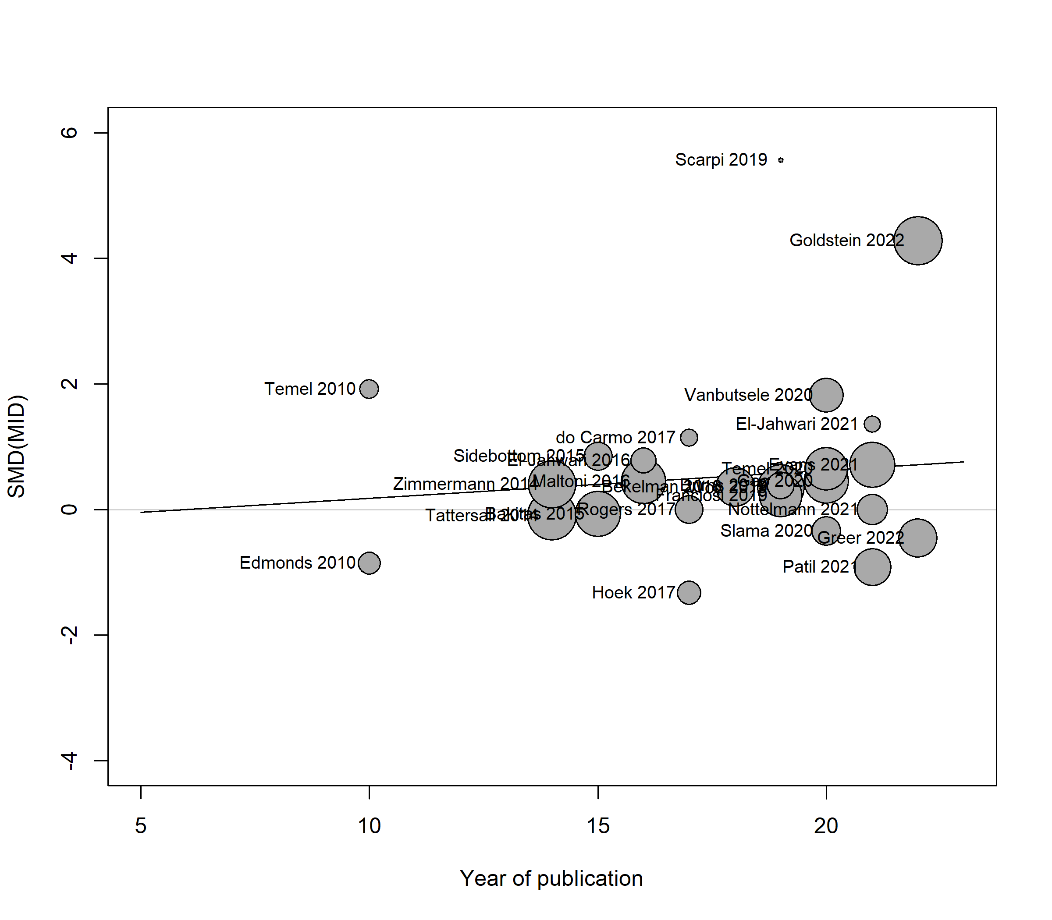 |

## Fig C and Table C: Quality of life at 13 to 36 weeks

**Analysis with effect size SMD (MID units)**

| **Study (k = 33)** | **SPC**  **MD_change_** | | **SPC**  **Total** | | **UC**  **MD_change_** | **UC**  **Total** | | **SMD (MID)** | **95% CI** | | **weight**  **(random,**  **in %)** | |
| --- | --- | --- | --- | --- | --- | --- | --- | --- | --- | --- | --- | --- |
| Bakitas *et al* 2009^55^ | 4.50 | | 69 | | 4.30 | 74 | | 0.04 | -1.39 to 1.47 | | 1.5 | |
| Bakitas *et al* 2015^56^ | 2.99 | | 72 | | 3.61 | 83 | | -0.07 | -0.67 to 0.54 | | 5.1 | |
| Bakitas *et al* 2020^57^ | 5.50 | | 120 | | 3.70 | 125 | | 0.36 | -0.67 to 1.39 | | 2.5 | |
| Bassi *et al* 2021^38^ | 0.90 | | 14 | | -1.20 | 19 | | 0.70 | 0.44 to 0.96 | | 9.1 | |
| Bekelman *et al* 2018^58^ | 5.50 | | 121 | | 2.90 | 121 | | 0.52 | -0.88 to 1.92 | | 1.5 | |
| Bekelman *et al* 2022^39^ | 6.00 | | 117 | | 1.40 | 116 | | 0.92 | 0.32 to 1.52 | | 5.1 | |
| Benthien *et al* 2020^59^ | 2.27 | | 79 | | 0.14 | 68 | | 0.37 | -0.57 to 1.32 | | 2.9 | |
| Braennstroem *et al* 2014^50^ | 12.70 | | 36 | | 4.10 | 36 | | 0.96 | -0.16 to 2.07 | | 2.2 | |
| Brims *et al* 2019^51^ | -3.70 | | 60 | | -2.30 | 65 | | -0.32 | -2.28 to 1.64 | | 0.8 | |
| do Carmo *et al* 2017^33^ | 2.78 | | 12 | | -20.24 | 14 | | 5.23 | -0.35 to 10.81 | | 0.1 | |
| Edmonds *et al* 2010^60^ | 0.30 | | 16 | | 7.10 | 7 | | -0.85 | -3.09 to 1.39 | | 0.6 | |
| El-Jahwari *et al* 2016^34^ | 1.70 | | 75 | | -0.64 | 74 | | 0.78 | -1.19 to 2.75 | | 0.8 | |
| El-Jahwari *et al* 2021^35^ | 13.70 | | 57 | | 5.60 | 48 | | 3.24 | -0.03 to 6.51 | | 0.3 | |
| Evans *et al* 2021^61^ | 0.88 | | 23 | | -0.54 | 24 | | 0.71 | 0.12 to 1.30 | | 5.2 | |
| Eychmueller *et al* 2021^40^ | 3.37 | | 59 | | 5.12 | 58 | | -0.35 | -1.36 to 0.66 | | 2.6 | |
| Franciosi *et al* 2019^62^ | -0.60 | | 111 | | -1.70 | 103 | | 0.22 | -0.57 to 1.01 | | 3.7 | |
| Gao *et al* 2020^63^ | 0.78 | | 176 | | 0.28 | 174 | | 0.46 | -0.22 to 1.13 | | 4.5 | |
| Given *et al* 2002^64^ | 12.00 | | 53 | | 5.00 | 59 | | 0.50 | -0.29 to 1.29 | | 3.7 | |
| Greer *et al* 2022^52^ | -0.21 | | 40 | | -0.12 | 39 | | -0.01 | -1.22 to 1.20 | | 1.9 | |
| Hoek *et al* 2017^36^ | -5.61 | | 13 | | 1.95 | 16 | | -1.33 | -3.45 to 0.80 | | 0.7 | |
| Kluger *et al* 2020^65^ | 0.66 | | 91 | | -0.84 | 94 | | 0.38 | 0.03 to 0.74 | | 7.9 | |
| Maltoni *et al* 2016^66^ | -0.92 | | 64 | | -4.49 | 65 | | 0.45 | -0.22 to 1.11 | | 4.5 | |
| Nottelmann *et al* 2021^41^ | 3.30 | | 133 | | 3.30 | 146 | | 0.00 | -1.58 to 1.58 | | 1.2 | |
| Patil *et al* 2021^32^ | 2.00 | | 34 | | 5.00 | 30 | | -0.50 | -2.14 to 1.14 | | 1.1 | |
| Rogers *et al* 2014^27^ | 27.00 | | 41 | | 20.70 | 40 | | 1.26 | -0.58 to 3.10 | | 0.9 | |
| Scarpi *et al* 2019^43^ | 1.65 | | 66 | | -1.30 | 65 | | 0.49 | -0.72 to 1.70 | | 1.9 | |
| Sidebottom *et al* 2015^67^ | 14.86 | | 79 | | 11.80 | 88 | | 0.85 | -0.89 to 2.59 | | 1.0 | |
| Slama *et al* 2020^68^ | 8.10 | | 37 | | 8.60 | 43 | | -0.11 | -2.08 to 1.85 | | 0.8 | |
| Tattersall *et al* 2014^44^ | -0.38 | | 33 | | 0.12 | 45 | | -0.17 | -0.32 to -0.02 | | 10.3 | |
| Temel *et al* 2010^37^ | 4.40 | | 60 | | -0.20 | 47 | | 1.92 | -0.64 to 4.47 | | 0.5 | |
| Vanbutsele *et al* 2020^45^ | -2.40 | | 91 | | -10.90 | 94 | | 1.93 | 0.65 to 3.21 | | 1.8 | |
| Woo *et al* 2019^46^ | 1.34 | | 45 | | 1.05 | 45 | | 0.14 | -0.39 to 0.68 | | 5.8 | |
| Zimmermann *et al* 2014^47^ | 2.46 | | 122 | | -3.95 | 149 | | 0.71 | 0.32 to 1.11 | | 7.3 | |
|  | |  | |  | | |  | | |  | |  |
| ***Meta-analysis*** | | **SMD (MID)** | | **95% CI** | | | ***t*** | | | ***p*** | |  |
| Random effects model | | 0.40 | | 0.21 to 0.59 | | | 4.300 | | | **<0.001** | |  |
|  | |  | |  | | |  | | |  | |  |
| ***Heterogeneity*** | |  | |  | | | ***Q (df)*** | | | ***p*** | |  |
| *τ²* | | 0.08 | | 0.01 to 0.42 | | | 80.11 (32) | | | **<0.001** | |  |
| *I²* | | 60.1% | | 41.5 to 72.7% | | |  | | |  | |  |
| *H* | | 1.58 | | 1.31 to 1.91 | | |  | | |  | |  |

**Forest plot**


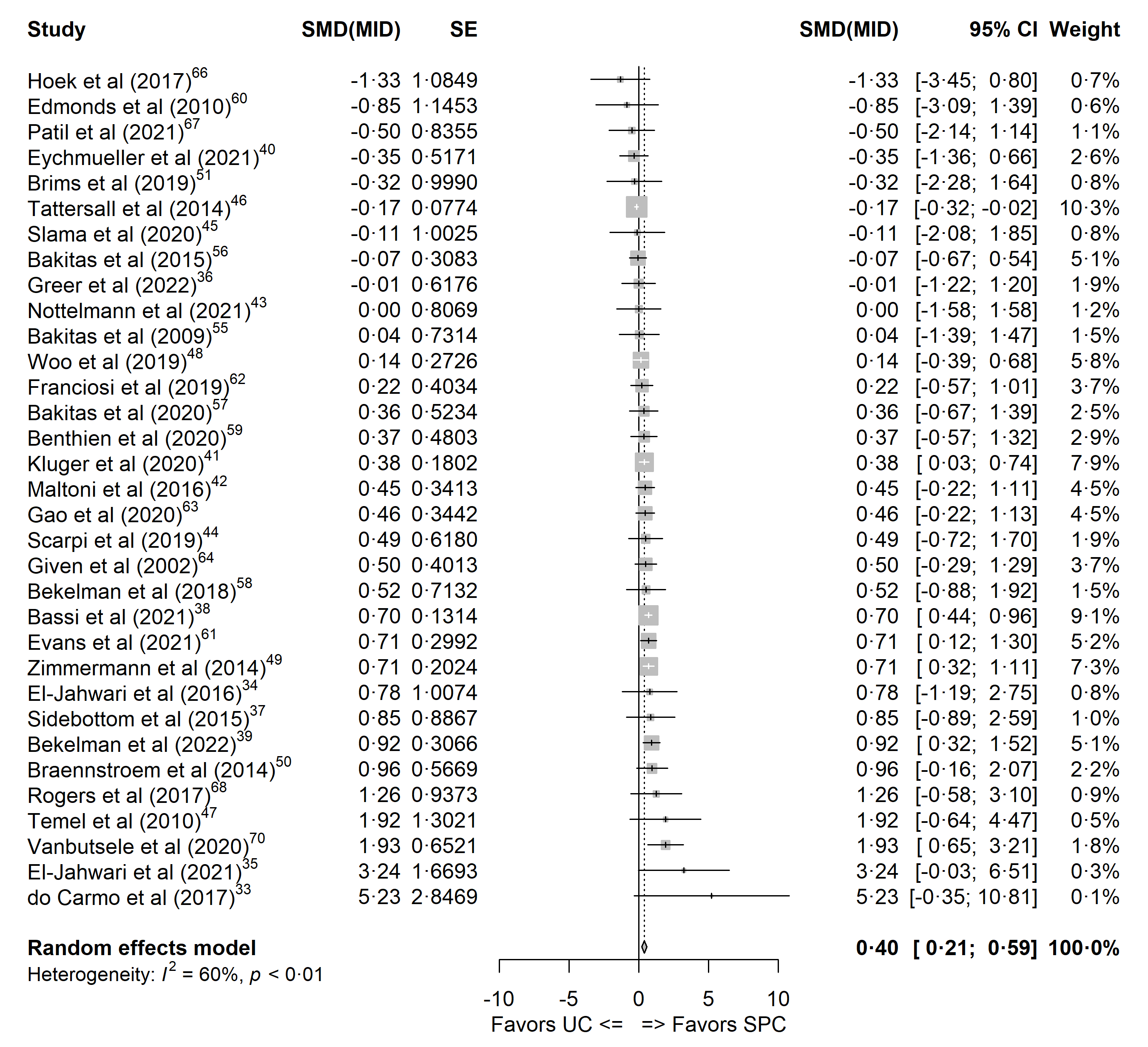


**Publication bias**

Egger’s enhanced funnel plot

| Linear regression test of funnel plot asymmetry  Intercept: 0.812  95% CI: 0.078 to 1.546  *t*(32) = 2.169, *p* = **0.038** | 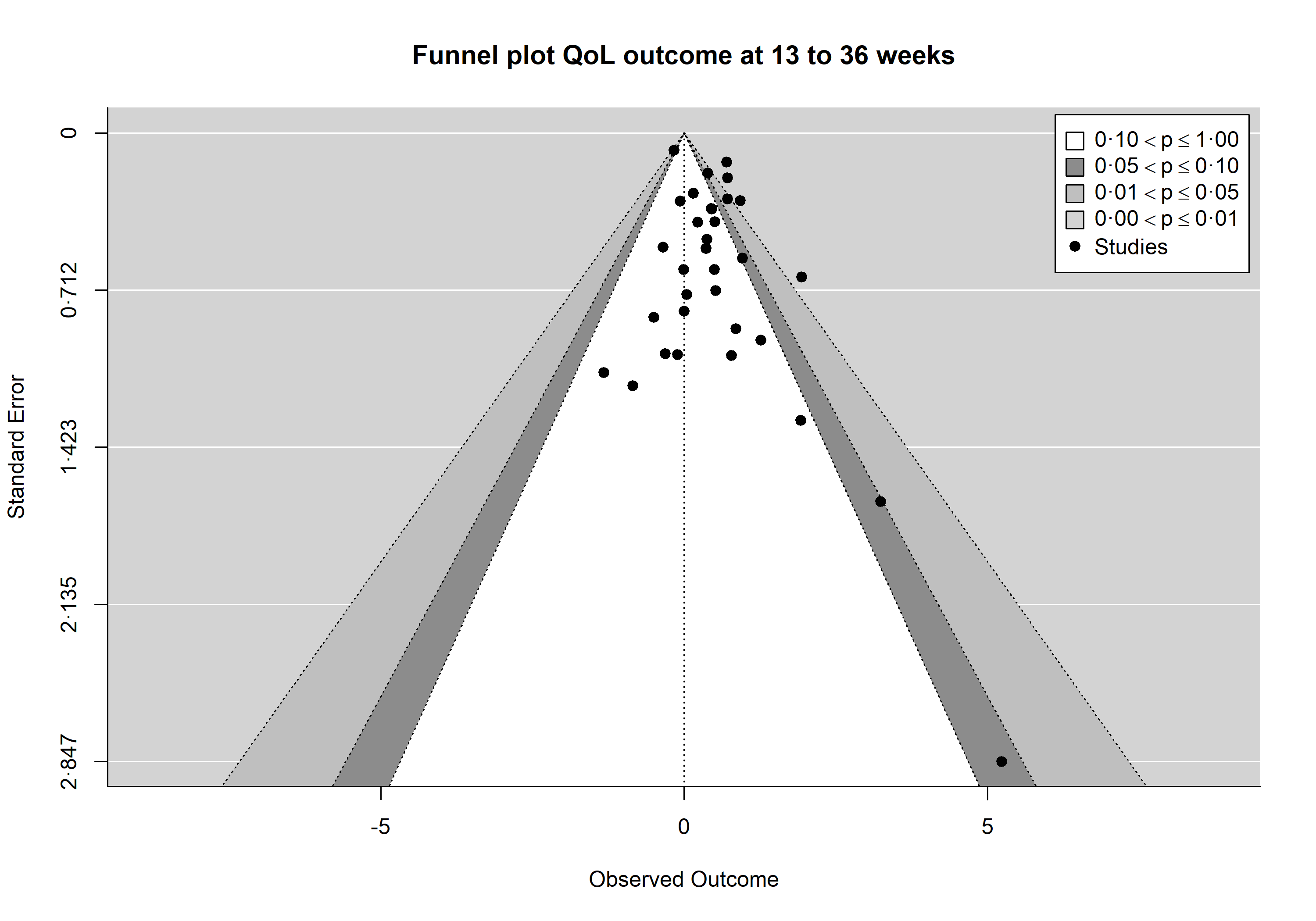 |
| --- | --- |

**Analysis with effect size RR (achieving change ≥1 MID)**

Number of studies combined: k = 33

Number of observations: 4493; Number of events: 1856

| **Study (k = 33)** | **SPC**  **#1MID** | | **SPC**  **Total** | **UC**  **#1MID** | | **UC**  **Total** | **RR**  **(> 1MID)** | | **95% CI** | | **weight (random,**  **in %)** | |
| --- | --- | --- | --- | --- | --- | --- | --- | --- | --- | --- | --- | --- |
| Bakitas *et al* 2009^55^ | 34 | | 69 | 36 | | 74 | 1.01 | | 0.72 to 1.42 | | 3.8 | |
| Bakitas *et al* 2015^56^ | 26 | | 72 | 31 | | 83 | 0.97 | | 0.64 to 1.46 | | 2.5 | |
| Bakitas *et al* 2020^57^ | 61 | | 120 | 59 | | 125 | 1.08 | | 0.83 to 1.39 | | 6.5 | |
| Bassi *et al* 2021^38^ | 1.5 | | 14 | 0.5 | | 19 | 4.07 | | 0.18 to 92.69 | | 0.0 | |
| Bekelman *et al* 2018^58^ | 61 | | 121 | 57 | | 121 | 1.07 | | 0.83 to 1.39 | | 6.4 | |
| Bekelman *et al* 2022^39^ | 62 | | 117 | 44 | | 116 | 1.40 | | 1.05 to 1.86 | | 5.1 | |
| Benthien *et al* 2020^59^ | 33 | | 79 | 25 | | 68 | 1.14 | | 0.76 to 1.71 | | 2.6 | |
| Braennstroem *et al* 2014^50^ | 21 | | 36 | 15 | | 36 | 1.40 | | 0.87 to 2.25 | | 1.9 | |
| Brims *et al* 2019^51^ | 22 | | 60 | 26 | | 65 | 0.92 | | 0.59 to 1.43 | | 2.1 | |
| do Carmo *et al* 2017^33^ | 6 | | 12 | 3 | | 14 | 2.33 | | 0.74 to 7.38 | | 0.3 | |
| Edmonds *et al* 2010^60^ | 5 | | 16 | 3 | | 7 | 0.73 | | 0.24 to 2.24 | | 0.3 | |
| El-Jahwari *et al* 2016^34^ | 35 | | 75 | 31 | | 74 | 1.11 | | 0.78 to 1.60 | | 3.3 | |
| El-Jahwari *et al* 2021^35^ | 40 | | 57 | 27 | | 48 | 1.25 | | 0.92 to 1.69 | | 4.7 | |
| Evans *et al* 2021^61^ | 6 | | 23 | 3 | | 24 | 2.09 | | 0.59 to 7.38 | | 0.3 | |
| Eychmueller *et al* 2021^40^ | 27 | | 59 | 29 | | 58 | 0.92 | | 0.63 to 1.34 | | 3.0 | |
| Franciosi *et al* 2019^62^ | 39 | | 111 | 34 | | 103 | 1.06 | | 0.73 to 1.55 | | 3.0 | |
| Gao *et al* 2020^63^ | 81 | | 176 | 71 | | 174 | 1.13 | | 0.89 to 1.43 | | 7.4 | |
| Given *et al* 2002^64^ | 25 | | 53 | 21 | | 59 | 1.33 | | 0.85 to 2.07 | | 2.1 | |
| Greer *et al* 2022^52^ | 14 | | 40 | 14 | | 39 | 0.98 | | 0.54 to 1.77 | | 1.2 | |
| Hoek *et al* 2017^36^ | 4 | | 13 | 6 | | 16 | 0.82 | | 0.29 to 2.30 | | 0.4 | |
| Kluger *et al* 2020^65^ | 25 | | 91 | 12 | | 94 | 2.15 | | 1.15 to 4.02 | | 1.1 | |
| Maltoni *et al* 2016^66^ | 16 | | 64 | 16 | | 65 | 1.02 | | 0.56 to 1.85 | | 1.2 | |
| Nottelmann *et al* 2021^41^ | 64 | | 133 | 71 | | 146 | 0.99 | | 0.78 to 1.26 | | 7.2 | |
| Patil *et al* 2021^32^ | 14 | | 34 | 14 | | 30 | 0.88 | | 0.51 to 1.54 | | 1.4 | |
| Rogers *et al* 2014^27^ | 35 | | 41 | 30 | | 40 | 1.14 | | 0.91 to 1.42 | | 8.8 | |
| Scarpi *et al* 2019^43^ | 28 | | 66 | 23 | | 65 | 1.20 | | 0.78 to 1.85 | | 2.3 | |
| Sidebottom *et al* 2015^67^ | 55 | | 79 | 58 | | 88 | 1.06 | | 0.86 to 1.30 | | 9.7 | |
| Slama *et al* 2020^68^ | 21 | | 37 | 25 | | 43 | 0.98 | | 0.67 to 1.43 | | 3.0 | |
| Tattersall *et al* 2014^44^ | 0.5 | | 33 | 1.5 | | 45 | 0.45 | | 0.02 to 10.81 | | 0.0 | |
| Temel *et al* 2010^37^ | 33 | | 60 | 21 | | 47 | 1.23 | | 0.83 to 1.82 | | 2.8 | |
| Vanbutsele *et al* 2020^45^ | 33 | | 91 | 20 | | 94 | 1.70 | | 1.06 to 2.74 | | 1.9 | |
| Woo *et al* 2019^46^ | 16 | | 45 | 17 | | 45 | 0.94 | | 0.55 to 1.62 | | 1.4 | |
| Zimmermann *et al* 2014^47^ | 41 | | 122 | 27 | | 149 | 1.85 | | 1.22 to 2.83 | | 2.4 | |
|  | |  | | |  | | |  | |  | |  |
| ***Meta-analysis*** | | **RR** | | | **95% CI** | | | ***t*** | | ***p*** | |  |
| Random effects model | | 1.13 | | | 1.06 to 1.20 | | | 3.890 | | **<0.001** | |  |
|  | |  | | |  | | |  | |  | |  |
| ***Heterogeneity*** | |  | | |  | | | ***Q (df)*** | | ***p*** | |  |
| *τ²* | | 0.00 | | | 0.00 to 0.03 | | | 27.68 (32) | | 0.685 | |  |
| *I²* | | 0.0% | | | 0.0 to 39.2% | | |  | |  | |  |
| *H* | | 1.00 | | | 1.00 to 1.28 | | |  | |  | |  |

**Forest plot of RR effect size for the QoL outcome 13 to 36 weeks**


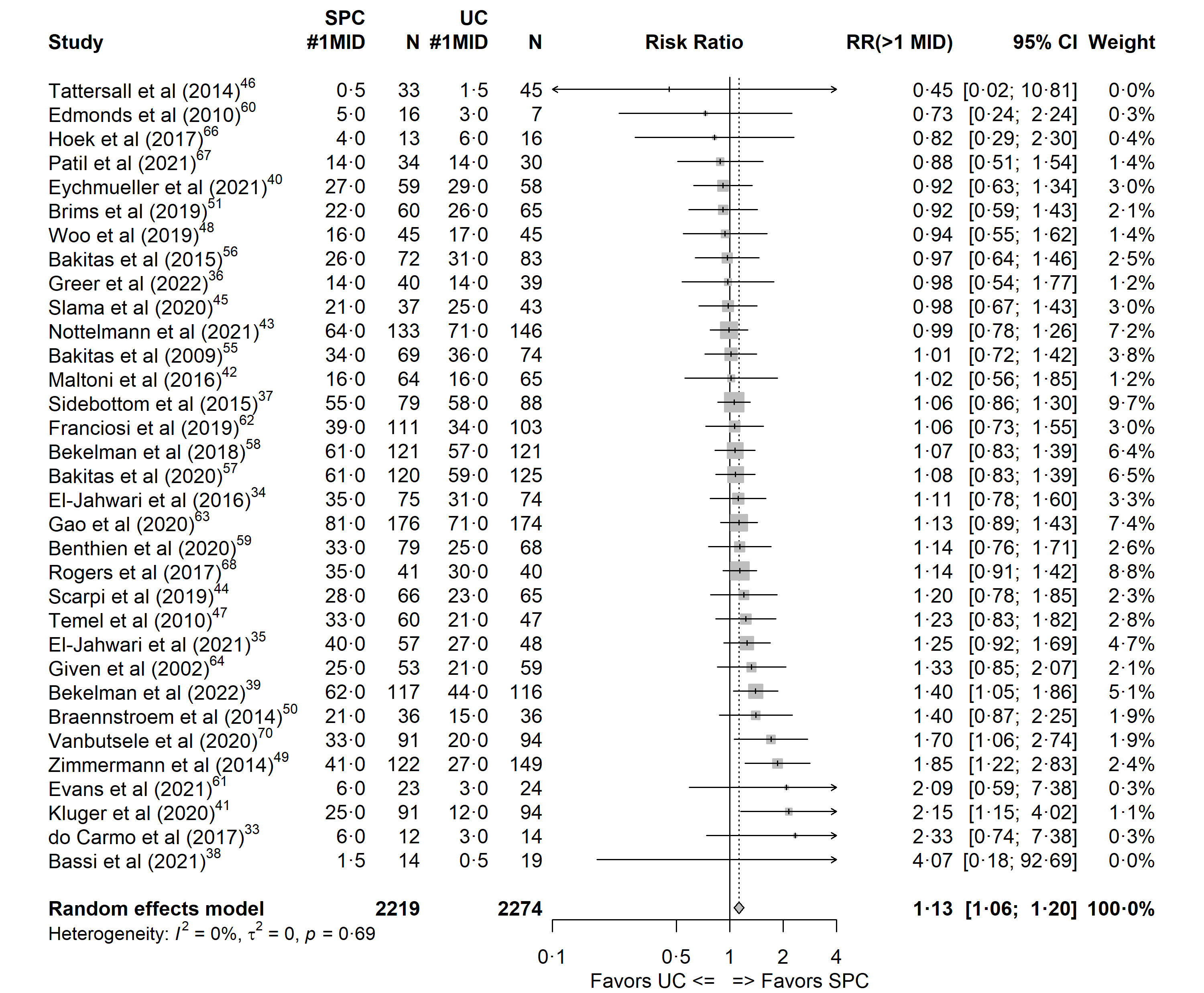


The risk ratio of RR = 1.13 (95% CI = 1.06 to 1.20) translates into a point estimate of a 13% increased probability of experiencing a change in QOL of at least 1 MID size with SPC. This is statistically significant.

The number needed to treat is calculated as follows (*p_CG_* is the baseline risk of experiencing change of at least 1 MID in the control group):

$$\frac{1}{p_{CG}\cdot(1-RR)}=\frac{1}{\frac{871}{2274}\cdot0.13}=20.083\to20$$

The NNT is 20, meaning that 20 people need to be treated with SPC in order for one person to have a change in QOL at 13 to 36 weeks of at least 1 MID.

**Meta-regression: Univariate meta-regression analyses with covariates**

| *k = 33* | **Regression** | | | | | **Heterogeneity** | | | **Test of moderators** | |
| --- | --- | --- | --- | --- | --- | --- | --- | --- | --- | --- |
|  | *b* | *SE* | *t* | *p* | 95% CI | *I²* | *Q* | *p* | *F* | *p* |
| ***Attrition (in %)*** |  |  |  |  |  |  |  |  |  |  |
| Intercept | 0.76 | 0.15 | 5.073 | **0.000** | - | 21 | 38.9 | 0.156 | 8.799 | **0.006** |
| Attrition (in %) | -0.01 | 0.00 | -2.966 | **0.006** | -0.02; -0.00 |  |  |  |  |  |
| ***% advanced disease*** |  |  |  |  |  |  |  |  |  |  |
| Intercept | 0.40 | 0.29 | 1.363 | 0.189 | - | 54 | 59.3 | **0.002** | 0.027 | 0.871 |
| % advanced disease | -0.00 | 0.00 | -0.164 | 0.871 | -0.01; 0.01 |  |  |  |  |  |
| ***Disease group (ref: Cancer)*** |  |  |  |  |  |  |  |  |  |  |
| Intercept | 0.27 | 0.24 | 1.133 | 0.268 | - | 91 | 287.8 | **0.000** | 3.488 | *0.073* |
| Non-cancer | 0.86 | 0.46 | 1.868 | *0.074* | -0.09; 1.81 |  |  |  |  |  |
| ***RoB2 score (ref: low risk)*** |  |  |  |  |  |  |  |  |  |  |
| Intercept | 0.42 | 0.21 | 1.985 | *0.056* | - | 45 | 77.5 | **0.000** | 0.013 | 0.987 |
| RoB2: Some risk | -0.04 | 0.25 | -0.151 | 0.881 | -0.54; 0.47 |  |  |  |  |  |
| RoB2: High risk | -0.02 | 0.30 | -0.050 | 0.960 | -0.63; 0.60 |  |  |  |  |  |
| ***Service composition score*** |  |  |  |  |  |  |  |  |  |  |
| Intercept | -0.46 | 0.24 | -1.938 | **0.062** | - | 12 | 36.3 | 0.234 | 13.180 | **0.001** |
| Service composition score | 0.07 | 0.02 | 3.631 | **0.001** | 0.03; 0.11 |  |  |  |  |  |
| ***Setting (ref: multiple settings)*** |  |  |  |  |  |  |  |  |  |  |
| Intercept | 0.38 | 0.16 | 2.364 | **0.025** | - | 46 | 77.1 | **0.000** | 0.287 | 0.753 |
| Inpatient consulting model | 0.36 | 0.48 | 0.746 | 0.461 | -0.63; 1.35 |  |  |  |  |  |
| Home or hospital outreach | 0.01 | 0.20 | 0.066 | 0.948 | -0.39; 0.42 |  |  |  |  |  |
| ***Type of intervention (ref: SPC)*** |  |  |  |  |  |  |  |  |  |  |
| Intercept | 0.32 | 0.29 | 1.113 | 0.275 | - | 32 | 48.4 | **0.013** | 1.674 | 0.194 |
| Early SPC | -0.07 | 0.31 | -0.208 | 0.836 | -0.71; 0.58 |  |  |  |  |  |
| Integrated collaborative care | 0.34 | 0.32 | 1.052 | 0.301 | -0.32; 0.99 |  |  |  |  |  |
| Nurse-led palliative care | -0.13 | 0.38 | -0.336 | 0.739 | -0.90; 0.65 |  |  |  |  |  |
| ***Year*** |  |  |  |  |  |  |  |  |  |  |
| Intercept | 0.13 | 0.39 | 0.350 | 0.729 | - | 38 | 61.1 | **0.001** | 0.491 | 0.489 |
| Year | 0.02 | 0.02 | 0.700 | 0.489 | -0.03; 0.06 |  |  |  |  |  |

.

**Bubble plots of univariate meta-regression analyses**

| **Attrition** | *F*(1,31) = 8.799  *p* = **0.006** | 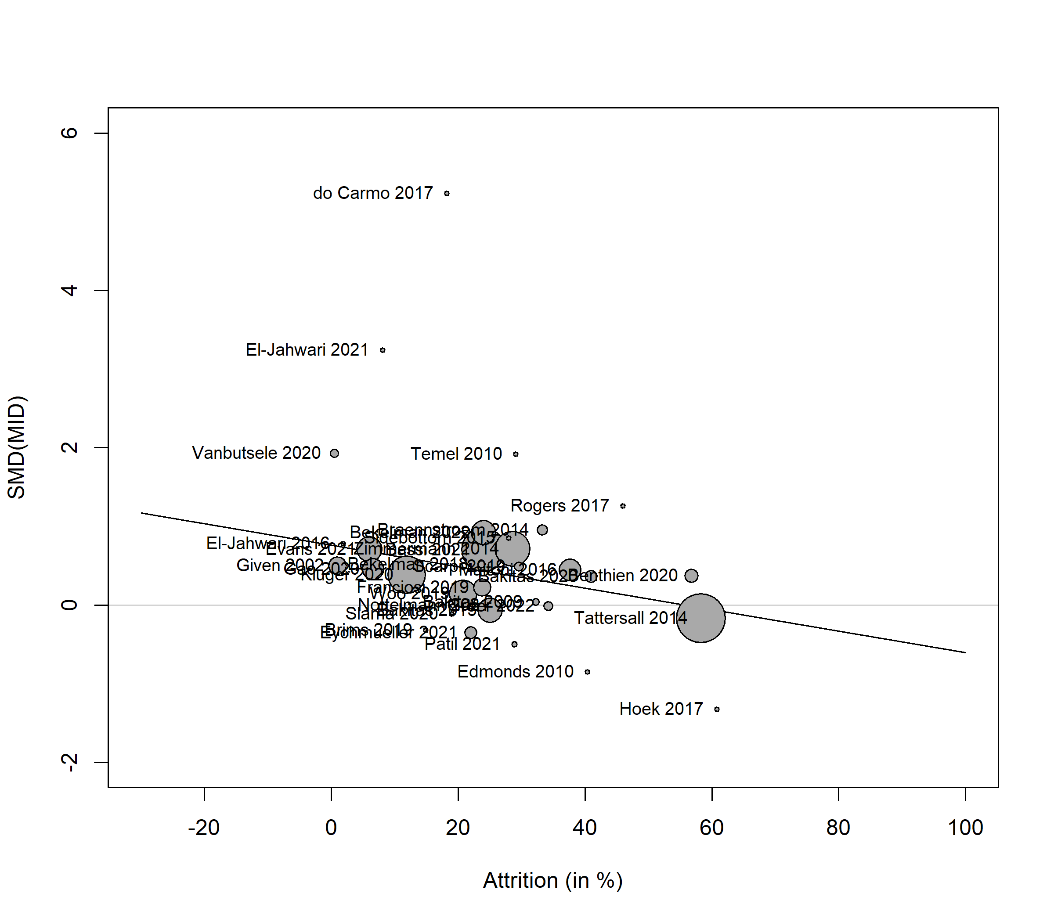 |
| --- | --- | --- |
| **% advanced disease** | *F*(1,18) = 0.027  *p* = 0.871 | 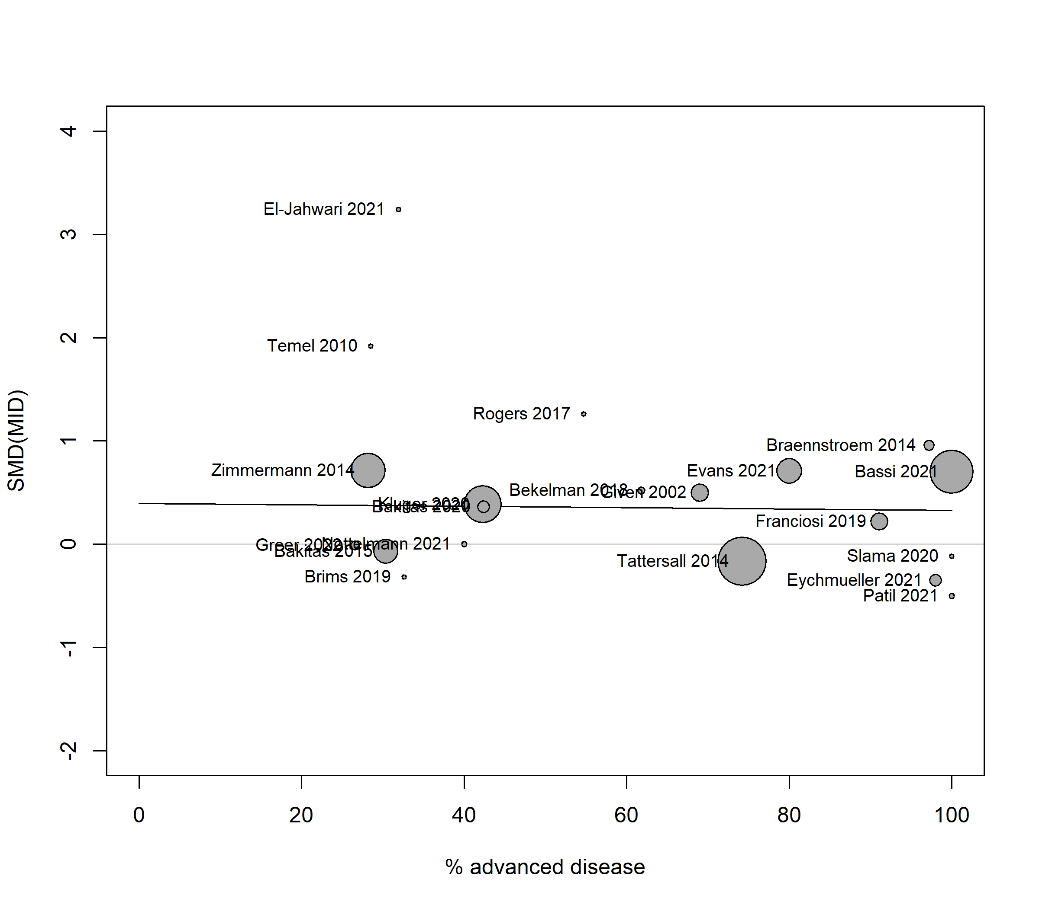 |
| **Disease group** | *F*(1,31) = 3.488  *p* = *0.073* | 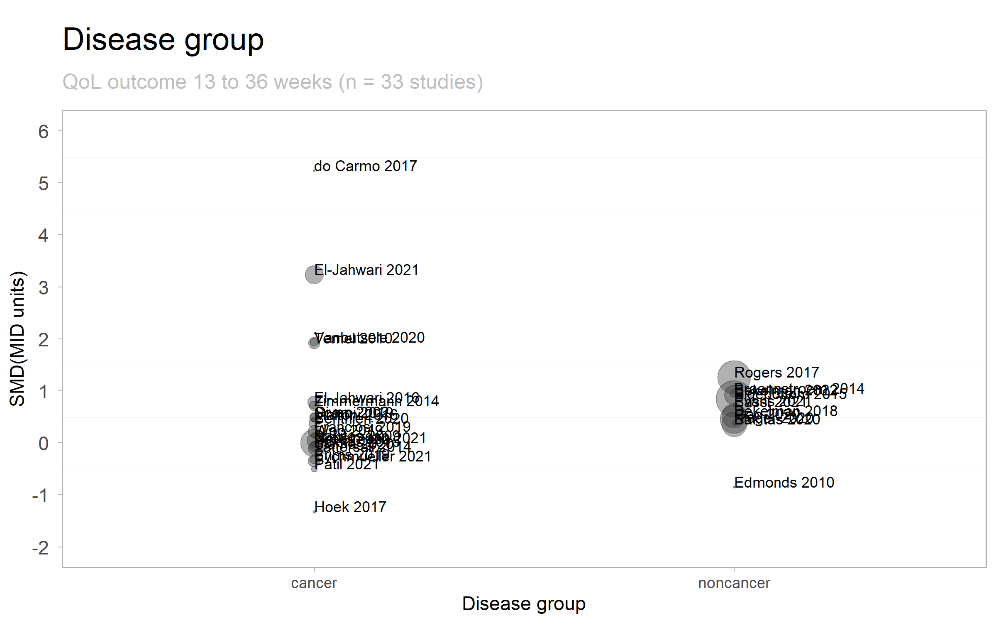 |
| **RoB2 score** | *F*(2,30) = 0.013  *p* = 0.987 | 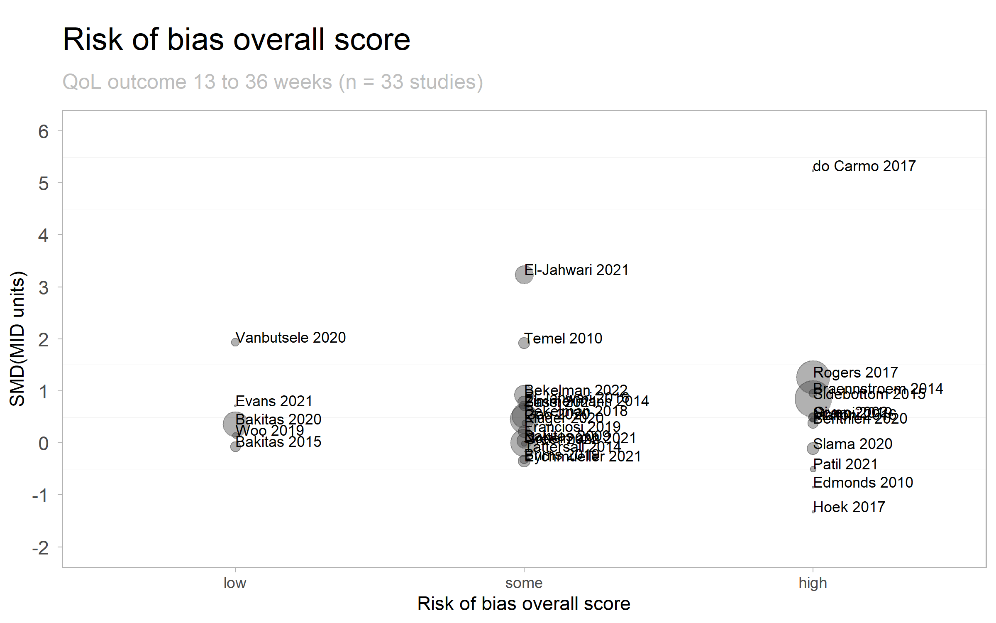 |
| **Service composition score** | *F*(1,31) = 13.180  *p* **= 0.001** | 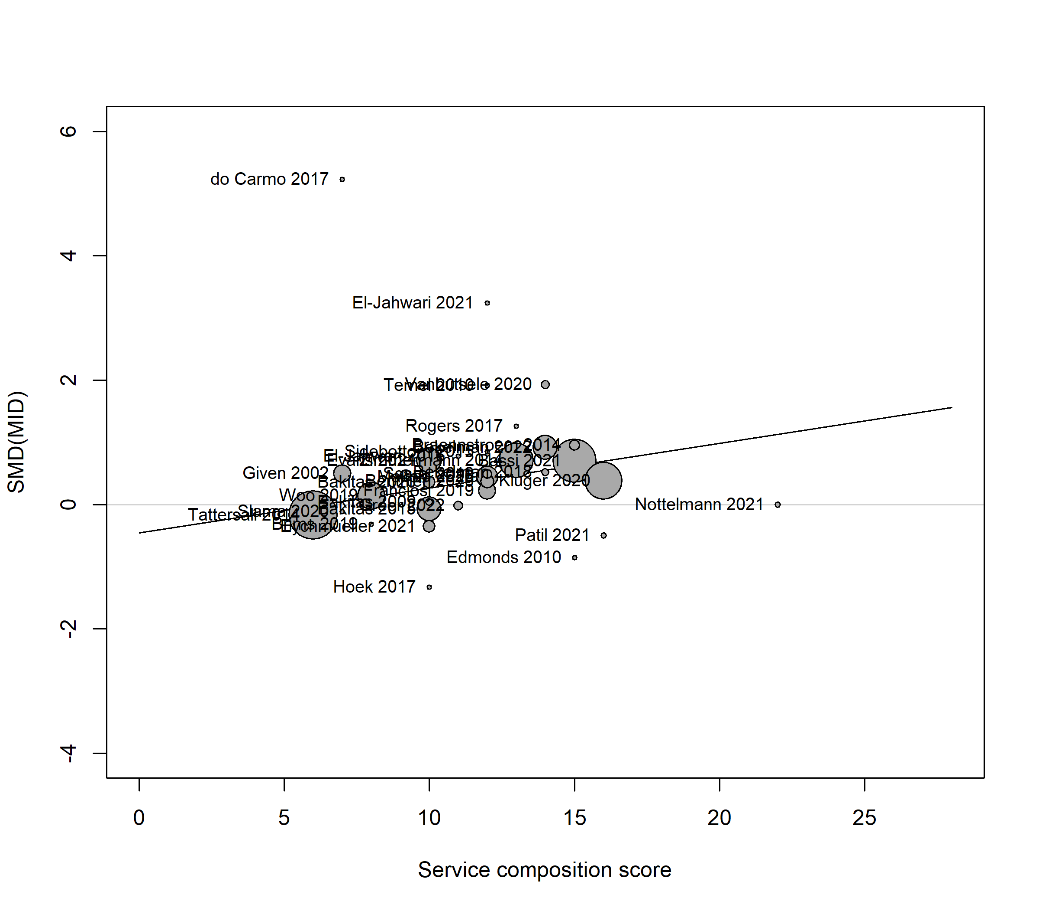 |
| **Setting** | *F*(2,30) = 0.287  *p* = 0.753 | 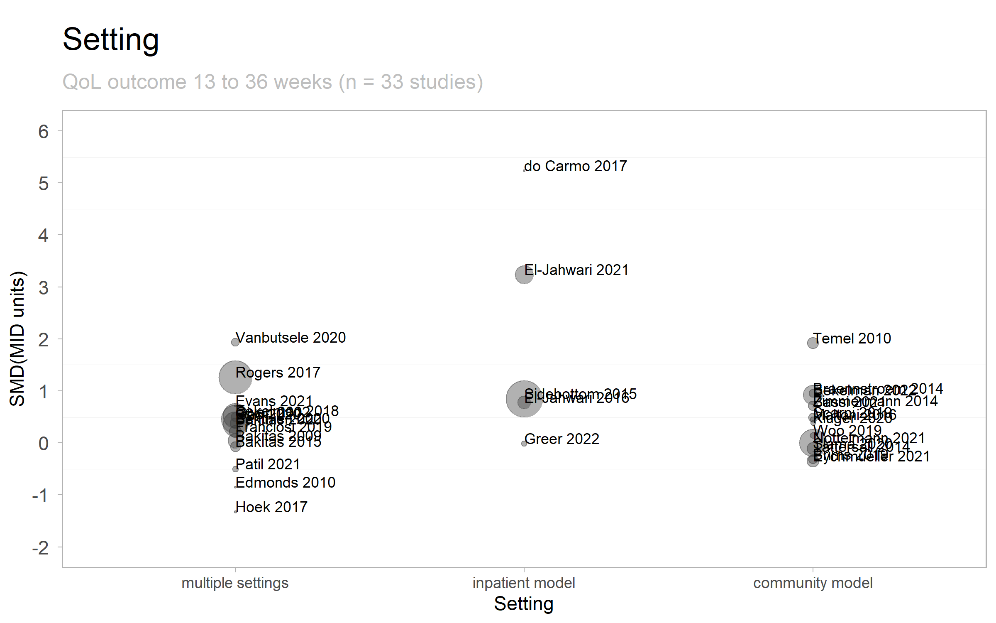 |
| **Type of intervention** | *F*(3,29) = 1.674  *p* = 0.194 | 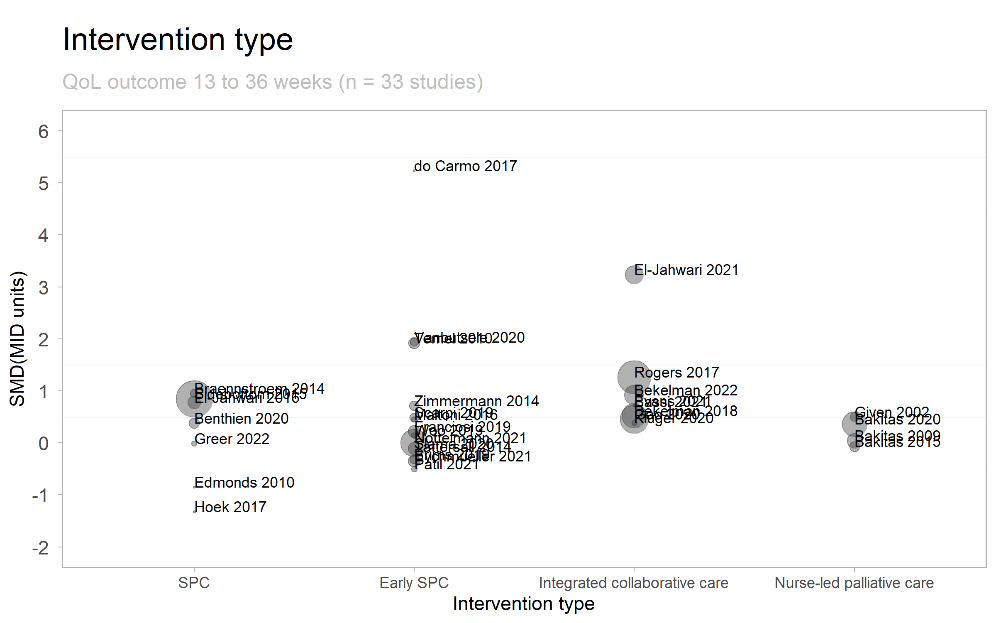 |
| **Year** | *F*(1,31) = 0.491  *p* = 0.489 | 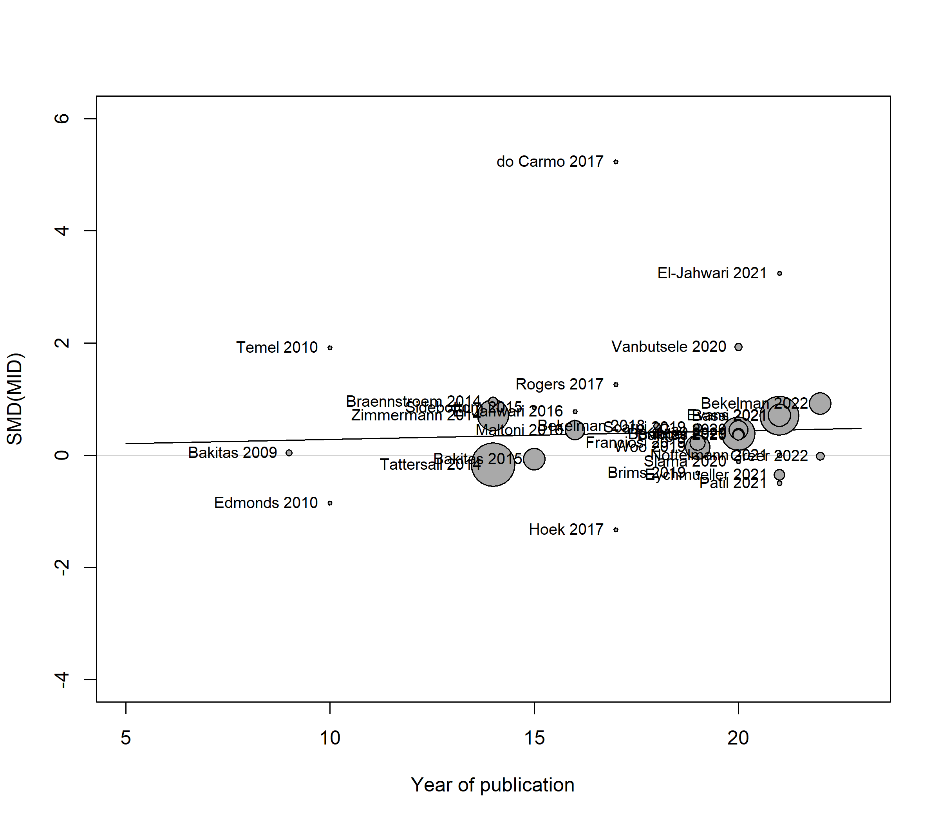 |

## Fig D and Table D: Quality of life at 37 to 52 weeks

**Analysis with effect size SMD (MID units)**

| **Study (k = 9)** | **SPC**  **MD_change_** | | **SPC**  **Total** | **UC**  **MD_change_** | | **UC**  **Total** | **SMD (MID)** | | **95% CI** | | **weight**  **(random,**  **in %)** | |
| --- | --- | --- | --- | --- | --- | --- | --- | --- | --- | --- | --- | --- |
| Aiken *et al* 2006^54^ | 0.35 | | 43 | -0.92 | | 30 | 0.16 | | 0.06 to 0.26 | | 16.6 | |
| Bakitas *et al* 2009^55^ | 5.00 | | 27 | 3.30 | | 31 | 0.34 | | -1.87 to 2.55 | | 4.6 | |
| Bakitas *et al* 2015^56^ | 2.23 | | 29 | 4.42 | | 28 | -0.44 | | -2.36 to 1.48 | | 5.6 | |
| Bassi *et al* 2021^38^ | 0.80 | | 14 | -2.90 | | 19 | 1.23 | | 0.00 to 2.47 | | 9.1 | |
| Given *et al* 2002^64^ | 39.00 | | 53 | 20.00 | | 59 | 1.36 | | 0.47 to 2.25 | | 11.6 | |
| Kluger *et al* 2020^41^ | 0.68 | | 92 | -0.43 | | 84 | 0.28 | | -0.08 to 0.65 | | 15.5 | |
| Tattersall *et al* 2014^46^ | -0.38 | | 21 | -0.28 | | 29 | -0.03 | | -0.22 to 0.16 | | 16.3 | |
| Vanbutsele *et al* 2020^70^ | -4.40 | | 91 | -15.90 | | 94 | 2.61 | | 1.40 to 3.83 | | 9.2 | |
| Woo *et al* 2019^48^ | 1.80 | | 19 | 1.50 | | 14 | 0.15 | | -0.77 to 1.07 | | 11.4 | |
|  | |  | | |  | | |  | |  | |  |
| ***Meta-analysis*** | | **SMD (MID)** | | | **95% CI** | | | ***t*** | | ***p*** | |  |
| Random effects model | | 0.58 | | | -0.09 to 1.26 | | | 2.010 | | 0.079 | |  |
|  | |  | | |  | | |  | |  | |  |
| ***Heterogeneity*** | |  | | |  | | | ***Q (df)*** | | ***p*** | |  |
| *τ²* | | 0.47 | | | 0.10 to 2.83 | | | 30.16 (8) | | **0.000** | |  |
| *I²* | | 73.5% | | | 48.3 to 86.4% | | |  | |  | |  |
| *H* | | 1.94 | | | 1.39 to 2.71 | | |  | |  | |  |

**Forest plot**


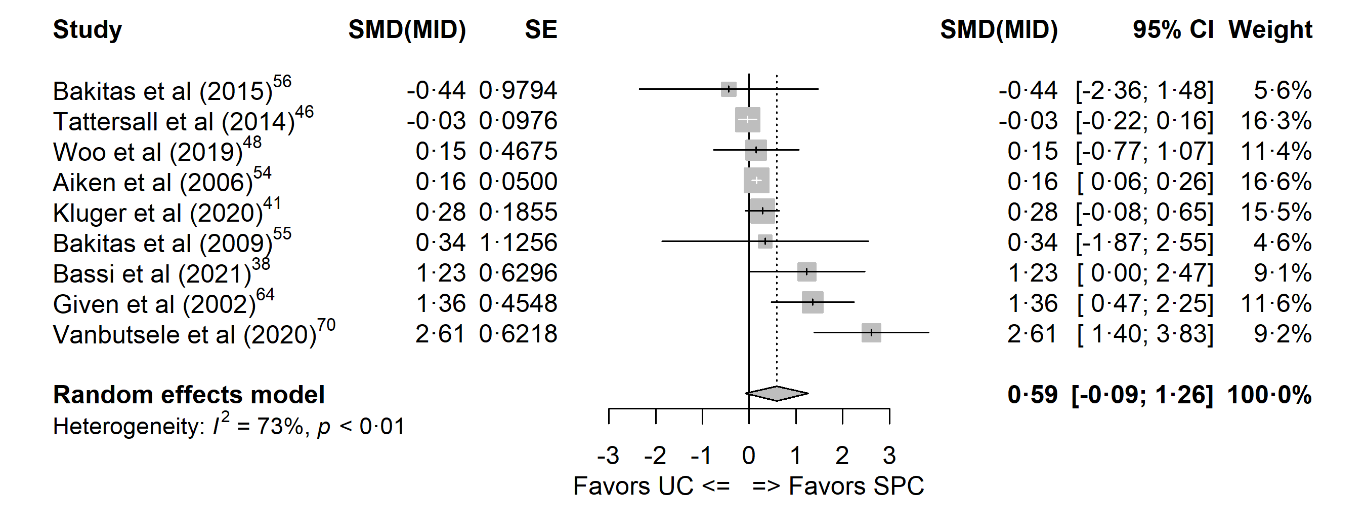


**Publication bias**

Egger’s enhanced funnel plot

| Linear regression test of funnel plot asymmetry  Intercept: 1.265  95% CI: -0.233 to 2.762  *t*(8) = 1.656, *p* = 0.141 | 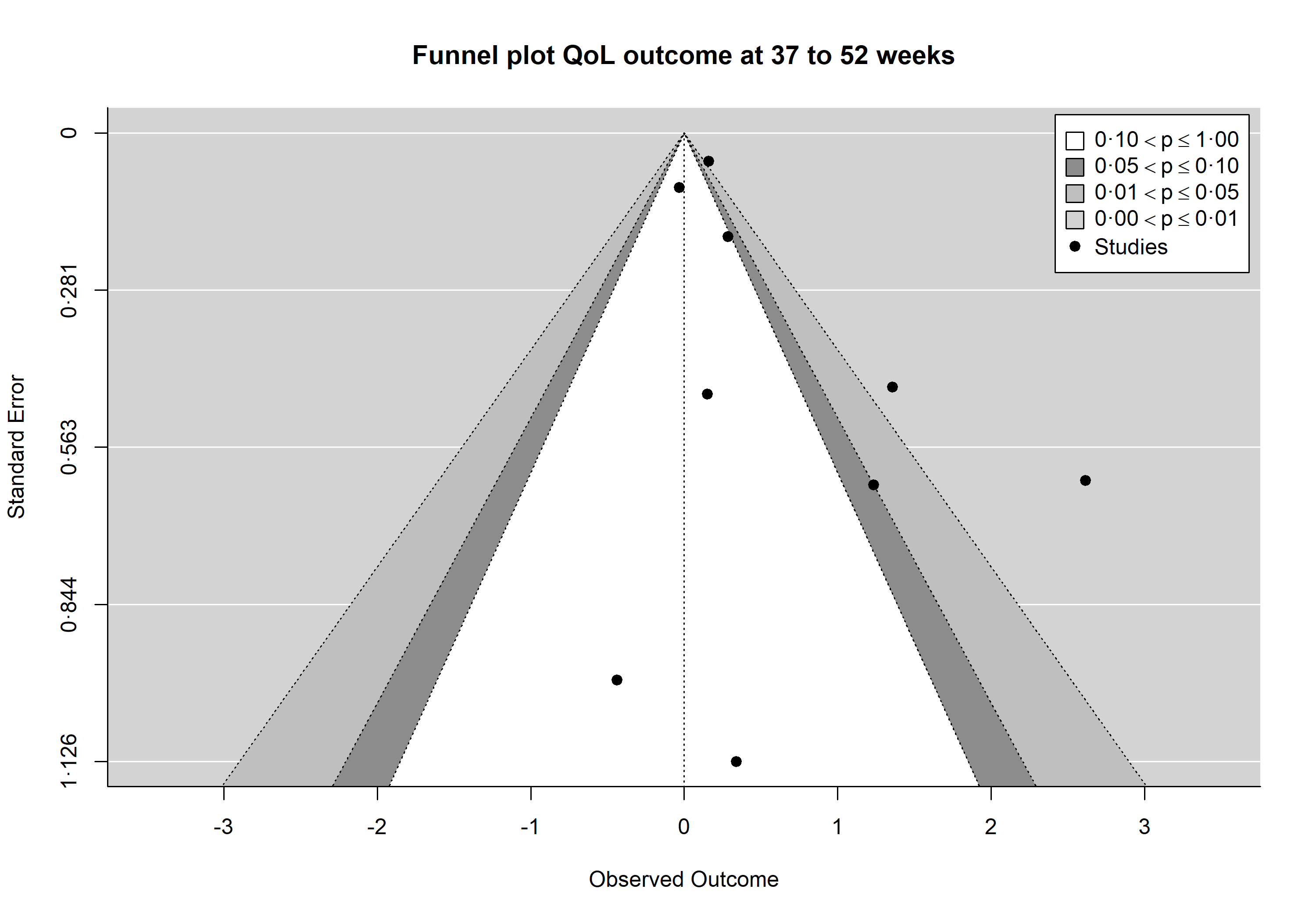 |
| --- | --- |

**Analysis with effect size RR (achieving change ≥1 MID)**

Number of studies combined: k = 9

Number of observations: 777; Number of events: 236

| **Study (k = 09)** | **SPC**  **#1MID** | | **SPC**  **Total** | **UC**  **#1MID** | | **UC**  **Total** | **RR**  **(> 1MID)** | | **95% CI** | | **weight (random,**  **in %)** | |
| --- | --- | --- | --- | --- | --- | --- | --- | --- | --- | --- | --- | --- |
| Aiken *et al* 2006^54^ | 0.5 | | 43 | 0.5 | | 30 | 0.70 | | 0.01 to 34.2 | | 0.2 | |
| Bakitas *et al* 2009^55^ | 14 | | 27 | 15 | | 31 | 1.07 | | 0.64 to 1.79 | | 12.8 | |
| Bakitas *et al* 2015^56^ | 13 | | 29 | 14 | | 28 | 0.90 | | 0.52 to 1.55 | | 11.3 | |
| Bassi *et al* 2021^38^ | 5 | | 14 | 3 | | 19 | 2.26 | | 0.65 to 7.93 | | 2.2 | |
| Given *et al* 2002^64^ | 40 | | 53 | 34 | | 59 | 1.31 | | 1.00 to 1.71 | | 47.4 | |
| Kluger *et al* 2020^41^ | 25 | | 92 | 14 | | 84 | 1.63 | | 0.91 to 2.92 | | 9.9 | |
| Tattersall *et al* 2014^46^ | 0.5 | | 21 | 0.5 | | 29 | 1.38 | | 0.03 to 66.84 | | 0.2 | |
| Vanbutsele *et al* 2020^70^ | 28 | | 91 | 14 | | 94 | 2.07 | | 1.16 to 3.66 | | 10.3 | |
| Woo *et al* 2019^48^ | 9 | | 19 | 6 | | 14 | 1.11 | | 0.51 to 2.38 | | 5.7 | |
|  | |  | | |  | | |  | |  | |  |
| ***Meta-analysis*** | | **RR** | | | **95% CI** | | | ***t*** | | ***p*** | |  |
| Random effects model | | 1.31 | | | 1.08 to 1.59 | | | 3.220 | | **0.012** | |  |
|  | |  | | |  | | |  | |  | |  |
| ***Heterogeneity*** | |  | | |  | | | ***Q (df)*** | | ***p*** | |  |
| *τ²* | | 0.00 | | | 0.00 to 0.43 | | | 6.41 (8) | | 0.601 | |  |
| *I²* | | 0.0% | | | 0.0 to 64.8% | | |  | |  | |  |
| *H* | | 1.00 | | | 1.00 to 1.69 | | |  | |  | |  |

**Forest plot of RR effect size for the QoL outcome 37 to 52 weeks**


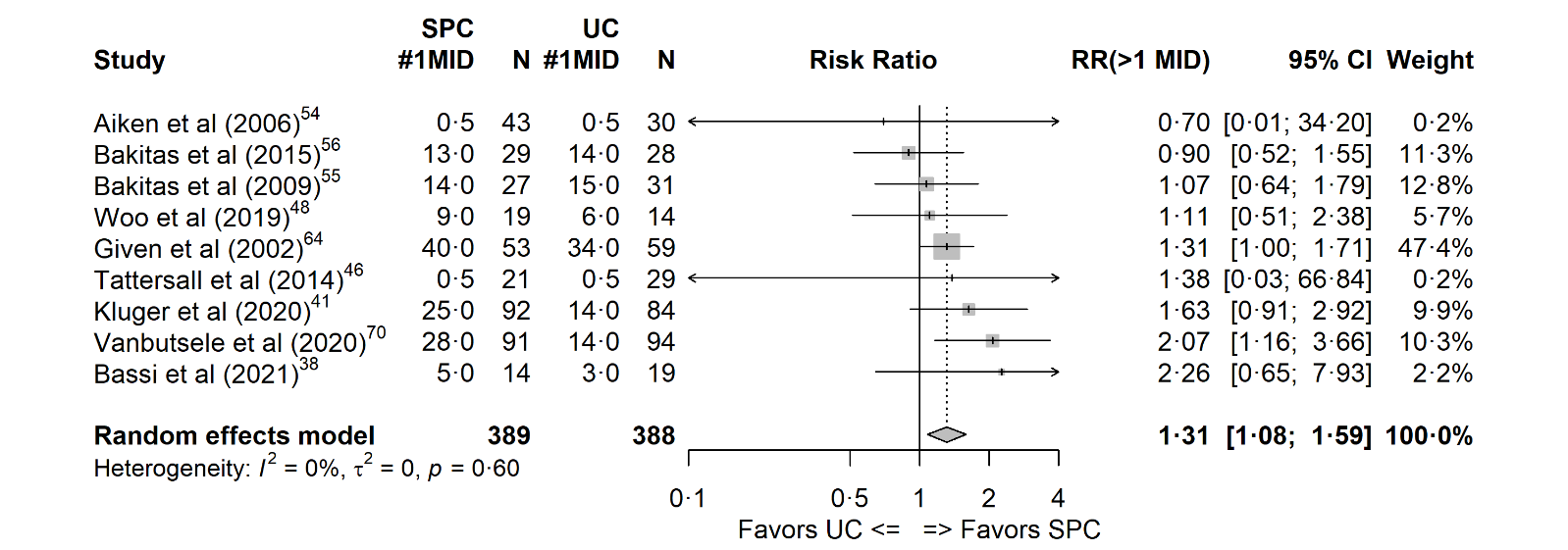


The risk ratio of RR = 1.31 (95% CI = 1.08to 1.59) translates into a point estimate of a 31% increased probability of experiencing a change in QOL of at least 1 MID size with SPC. This is statistically significant.

The number needed to treat is calculated as follows (*p_CG_* is the baseline risk of experiencing change of at least 1 MID in the control group):

$$\frac{1}{p_{CG}\cdot(1-RR)}=\frac{1}{\frac{101}{388}\cdot0.31}=12.392 \to12$$

The NNT is 12, meaning that 12 people need to be treated with SPC in order for one person to have a change in QOL at 36 to 52 weeks of at least 1 MID.

**Meta-regression: Univariate meta-regression analyses with covariates**

| *k = 9* | **Regression** | | | | | **Heterogeneity** | | | **Test of moderators** | |
| --- | --- | --- | --- | --- | --- | --- | --- | --- | --- | --- |
|  | *b* | *SE* | *t* | *p* | 95% CI | *I²* | *Q* | *p* | *F* | *p* |
| ***Attrition (in %)*** |  |  |  |  |  |  |  |  |  |  |
| Intercept | 1.17 | 0.38 | 3.112 | **0.017** | - | 87 | 21.5 | **0.003** | 4.569 | *0.069* |
| Attrition (in %) | -0.02 | 0.01 | -2.138 | *0.069* | -0.04; 0.00 |  |  |  |  |  |
| ***% advanced disease*** |  |  |  |  |  |  |  |  |  |  |
| Intercept | -0.53 | 0.98 | -0.545 | 0.624 | - | 73 | 13.6 | **0.004** | 1.188 | 0.355 |
| % advanced disease | 0.02 | 0.01 | 1.090 | 0.355 | -0.03; 0.06 |  |  |  |  |  |
| ***Disease group (ref: Cancer)*** |  |  |  |  |  |  |  |  |  |  |
| Intercept | 0.69 | 0.40 | 1.714 | 0.130 | - | 88 | 29.5 | **0.000** | 0.135 | 0.725 |
| Non-cancer | -0.23 | 0.64 | -0.367 | 0.725 | -1.74; 1.27 |  |  |  |  |  |
| ***RoB2 score (ref: low risk)*** |  |  |  |  |  |  |  |  |  |  |
| Intercept | 0.88 | 0.63 | 1.403 | 0.210 | - | 88 | 24.8 | **0.000** | 0.199 | 0.825 |
| RoB2: Some risk | -0.49 | 0.80 | -0.616 | 0.561 | -2.45; 1.47 |  |  |  |  |  |
| RoB2: High risk | -0.20 | 0.89 | -0.226 | 0.829 | -2.39; 1.99 |  |  |  |  |  |
| ***Service composition score*** |  |  |  |  |  |  |  |  |  |  |
| Intercept | 0.18 | 0.93 | 0.191 | 0.854 | - | 88 | 28.2 | **0.000** | 0.235 | 0.643 |
| Service composition score | 0.04 | 0.08 | 0.484 | 0.643 | -0.14; 0.22 |  |  |  |  |  |
| ***Setting (ref: multiple settings)*** |  |  |  |  |  |  |  |  |  |  |
| Intercept | 1.41 | 0.46 | 3.104 | **0.017** | - | 26 | 15.1 | **0.034** | 7.487 | **0.029** |
| Inpatient consulting model | - | - | - | - | - |  |  |  |  |  |
| Home or hospital outreach | -1.28 | 0.47 | -2.736 | **0.029** | -2.39; -0.17 |  |  |  |  |  |
| ***Type of intervention (ref: SPC)*** |  |  |  |  |  |  |  |  |  |  |
| Intercept (Nurse-led PC) | 0.44 | 0.54 | 0.815 | 0.447 | - | 81 | 27.1 | **0.000** | 0.084 | 0.920 |
| Early SPC | 0.31 | 0.78 | 0.398 | 0.704 | -1.61; 2.23 |  |  |  |  |  |
| Integrated collaborative care | 0.23 | 0.88 | 0.263 | 0.802 | -1.93; 2.39 |  |  |  |  |  |
| ***Year*** |  |  |  |  |  |  |  |  |  |  |
| Intercept | 0.44 | 0.72 | 0.601 | 0.567 | - | 90 | 30.2 | **0.000** | 0.065 | 0.807 |
| Year | 0.01 | 0.05 | 0.254 | 0.807 | -0.10; 0.12 |  |  |  |  |  |

.

**Bubble plots of univariate meta-regression analyses**

| **Attrition** | *F*(1,7) = 4.569  *p* = *0.069* | 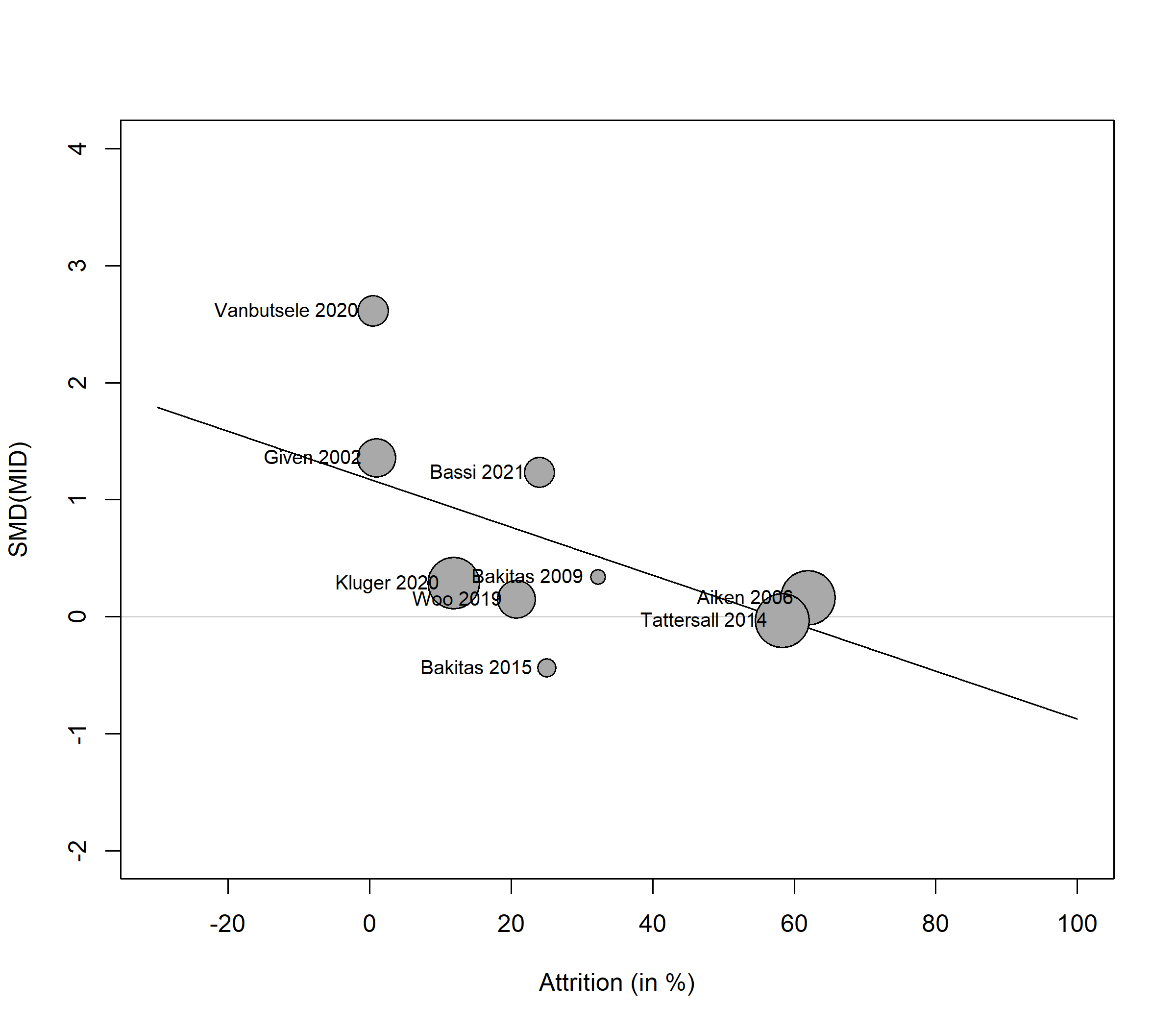 |
| --- | --- | --- |
| **% advanced disease** | *F*(1,3) = 1.188  *p* = 0.355 | 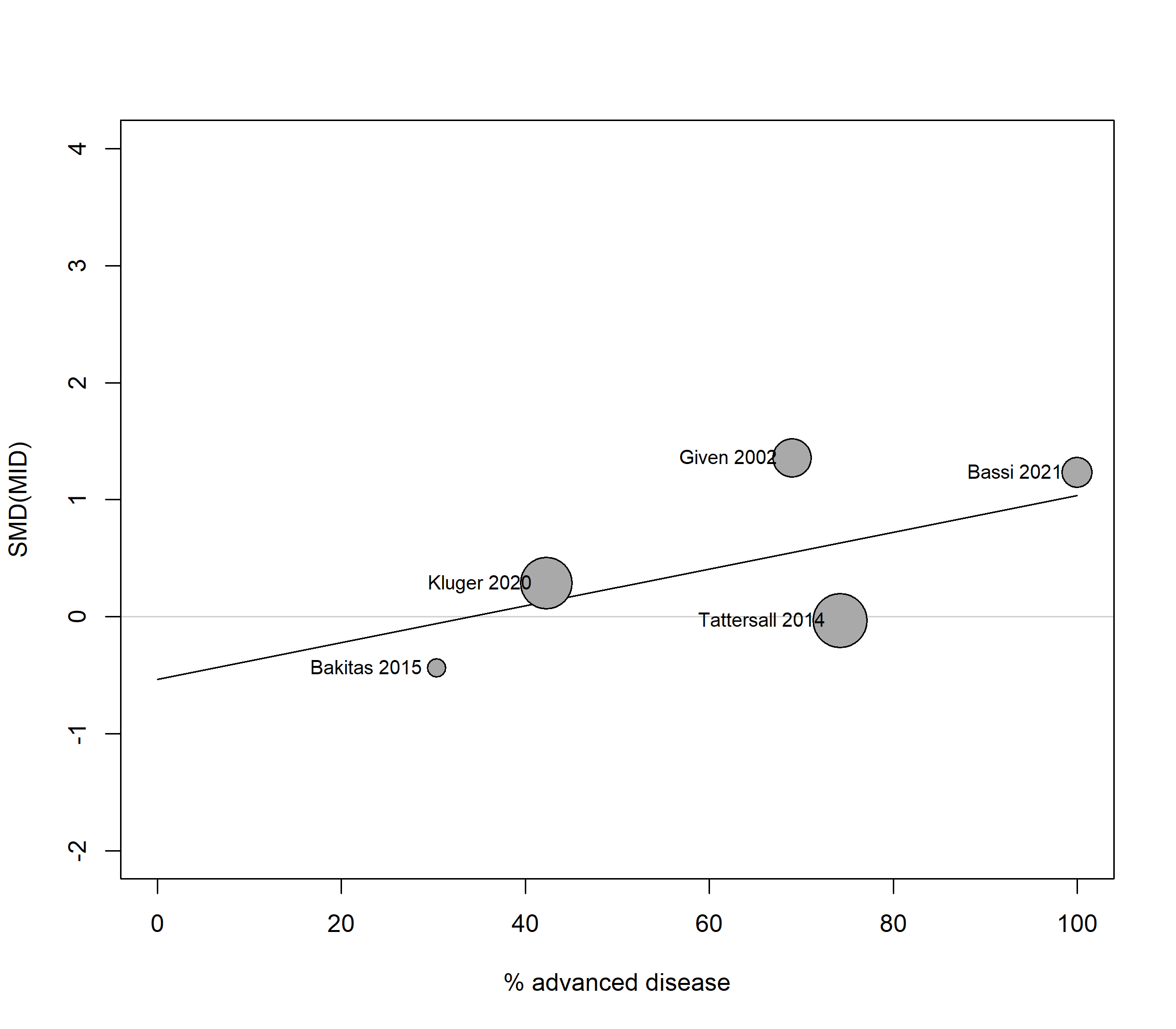 |
| **Disease group** | *F*(1,7) = 0.135  *p* = 0.725 | 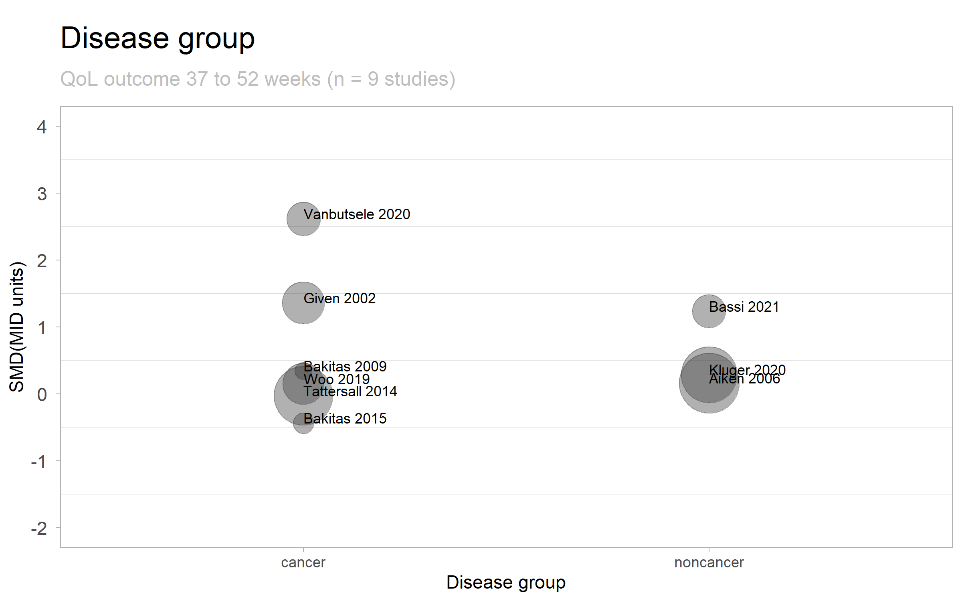 |
| **RoB2 score** | *F*(2,6) = 0.199  *p* = 0.825 | 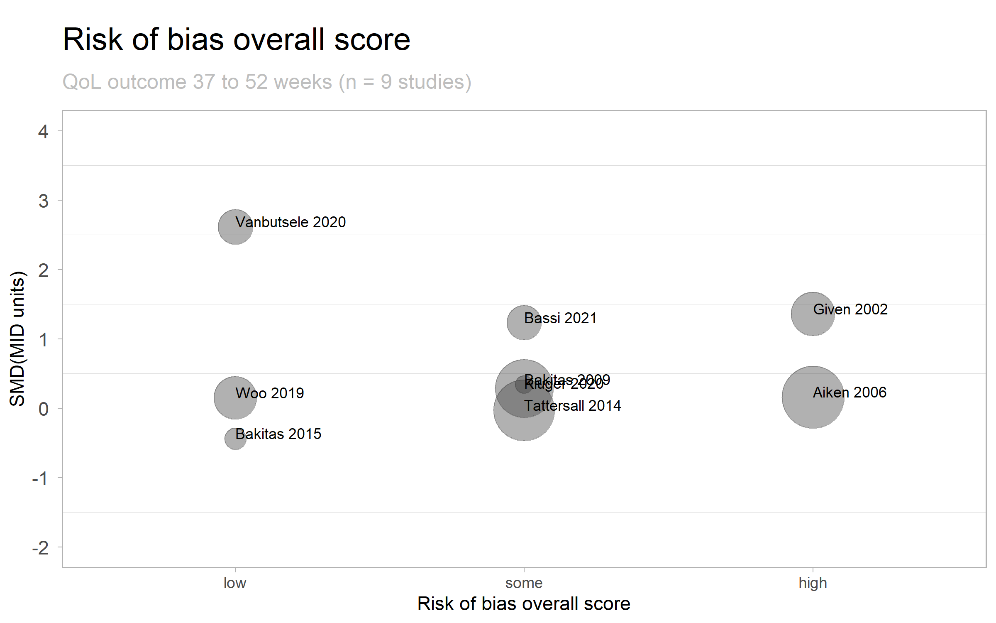 |
| **Service composition score** | *F*(1,7) = 0.235  *p* = 0.643 | 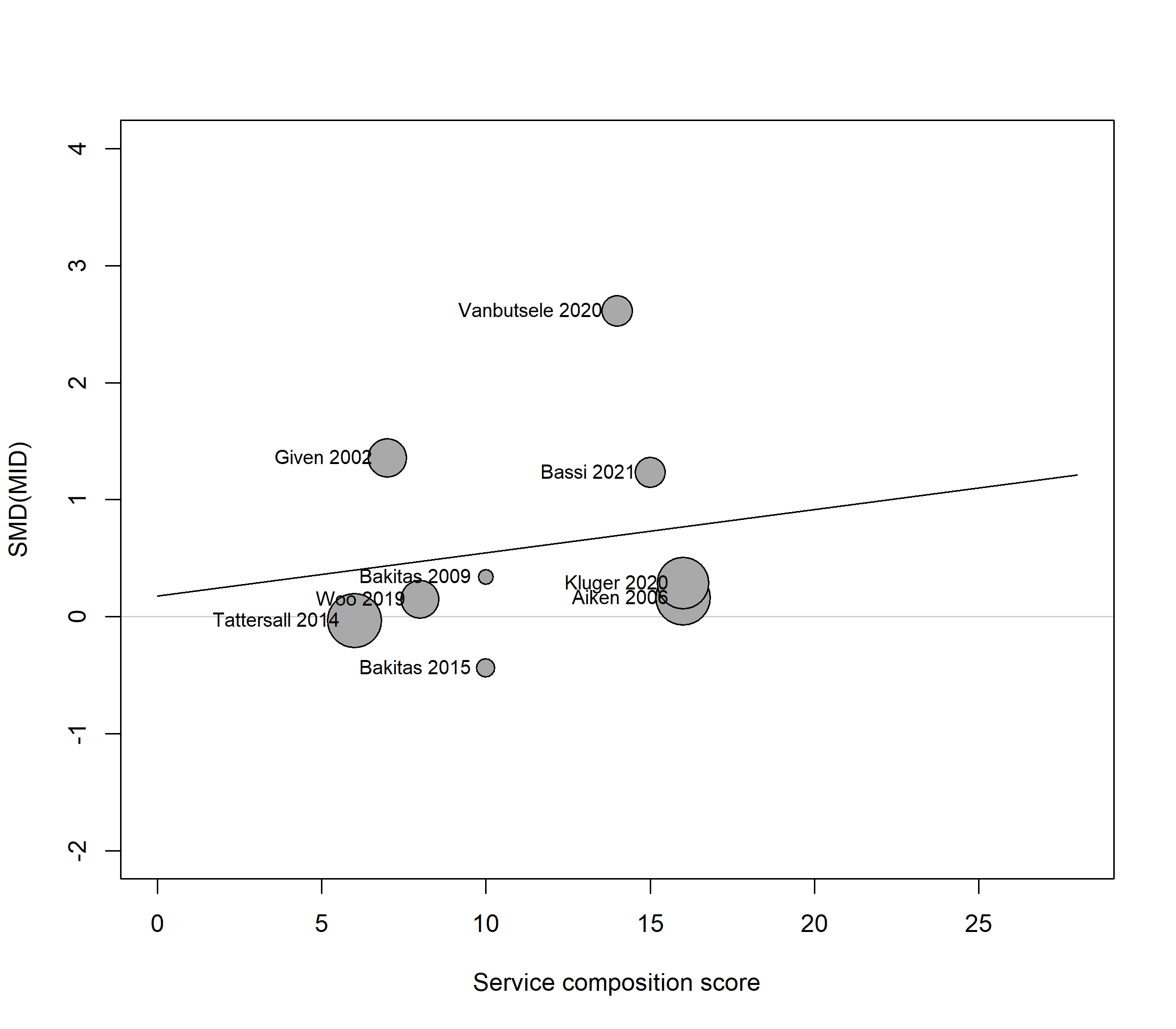 |
| **Setting** | *F*(1,7) = 7.487  *p* = **0.029** | 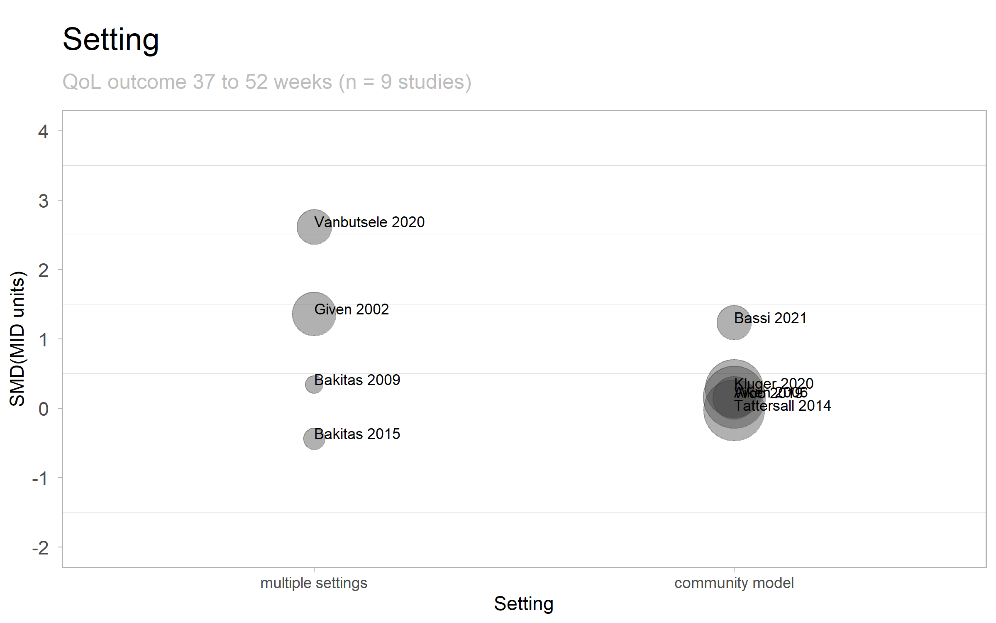 |
| **Type of intervention** | *F*(2,6) = 0.084  *p* = 0.920 | 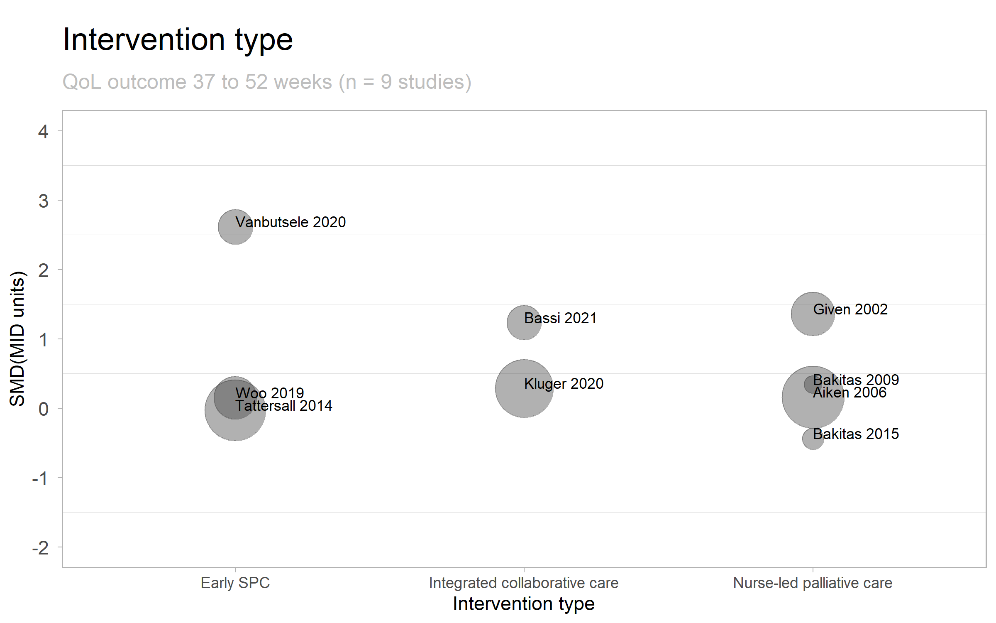 |
| **Year** | *F*(1,7) = 0.065  *p* = 0.807 | 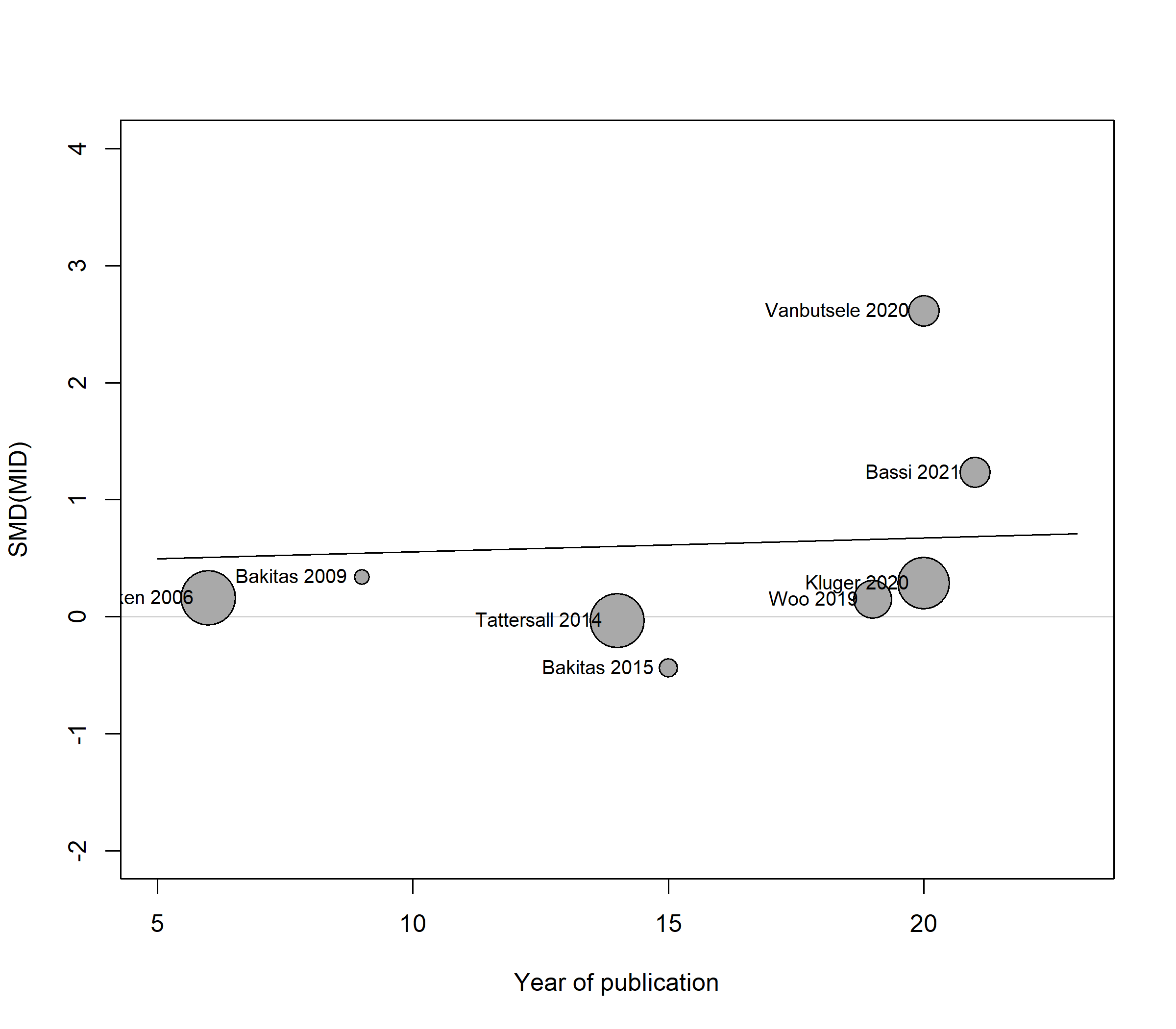 |

## Fig E and Table E: Emotional wellbeing at 2 to 11 weeks

**Analysis with effect size SMD (MID units)**

| **Study (k = 14)** | **SPC**  **MD_change_** | | **SPC**  **Total** | **UC**  **MD_change_** | **UC**  **Total** | | **SMD (MID)** | **95% CI** | | **weight**  **(random,**  **in %)** | |
| --- | --- | --- | --- | --- | --- | --- | --- | --- | --- | --- | --- |
| Bakitas *et al* 2009^55^ | 1.60 | | 102 | 2.00 | 98 | | -0.13 | -0.94 to 0.67 | | 7.3 | |
| Bakitas *et al* 2020^57^ | 0.10 | | 116 | 0.10 | 142 | | 0.00 | -0.65 to 0.65 | | 7.5 | |
| do Carmo *et al* 2017^33^ | -1.90 | | 21 | -0.89 | 19 | | -0.76 | -2.72 to 1.19 | | 5.2 | |
| Edmonds *et al* 2010^60^ | -1.30 | | 19 | 2.30 | 16 | | -0.45 | -0.91 to 0.01 | | 7.8 | |
| El-Jahwari *et al* 2016^34^ | -3.45 | | 80 | -4.12 | 77 | | 0.13 | -0.16 to 0.43 | | 7.9 | |
| El-Jahwari *et al* 2021^35^ | -0.04 | | 78 | -1.10 | 69 | | 0.21 | -0.11 to 0.53 | | 7.9 | |
| Eychmueller *et al* 2021^40^ | 0.02 | | 61 | 0.00 | 68 | | 0.01 | -0.30 to 0.32 | | 7.9 | |
| Greer *et al* 2022^52^ | 0.16 | | 54 | 0.98 | 53 | | -0.62 | -1.74 to 0.50 | | 6.8 | |
| Groenvold *et al* 2017^65^ | -0.40 | | 130 | 0.20 | 137 | | -0.46 | -1.22 to 0.31 | | 7.4 | |
| Hoek *et al* 2017^36^ | -0.24 | | 23 | 0.99 | 26 | | -0.93 | -2.68 to 0.82 | | 5.5 | |
| Liu *et al* 2022^32^ | 21.88 | | 83 | 2.53 | 83 | | 4.84 | 4.08 to 5.60 | | 7.4 | |
| Sidebottom *et al* 2015^67^ | 2.99 | | 86 | 1.56 | 89 | | 0.29 | 0.01 to 0.56 | | 7.9 | |
| Tattersall *et al* 2014^44^ | -0.50 | | 58 | 1.80 | 57 | | -0.22 | -0.44 to 0.00 | | 7.9 | |
| Woo *et al* 2019^46^ | -2.09 | | 112 | -2.09 | 116 | | 0.00 | -1.65 to 1.65 | | 5.7 | |
|  | |  | |  | |  | | |  | |  |
| ***Meta-analysis*** | | **SMD (MID)** | | **95% CI** | | ***t*** | | | ***p*** | |  |
| Random effects model | | 0.18 | | -0.64 to 0.99 | | 0.470 | | | 0.643 | |  |
|  | |  | |  | |  | | |  | |  |
| ***Heterogeneity*** | |  | |  | | ***Q (df)*** | | | ***p*** | |  |
| *τ²* | | 1.81 | | 0.85 to 4.91 | | 172.18 (13) | | | **<0.001** | |  |
| *I²* | | 92.4% | | 89.0 to 94.8% | |  | | |  | |  |
| *H* | | 3.64 | | 3.02 to 4.38 | |  | | |  | |  |

**Forest plot**


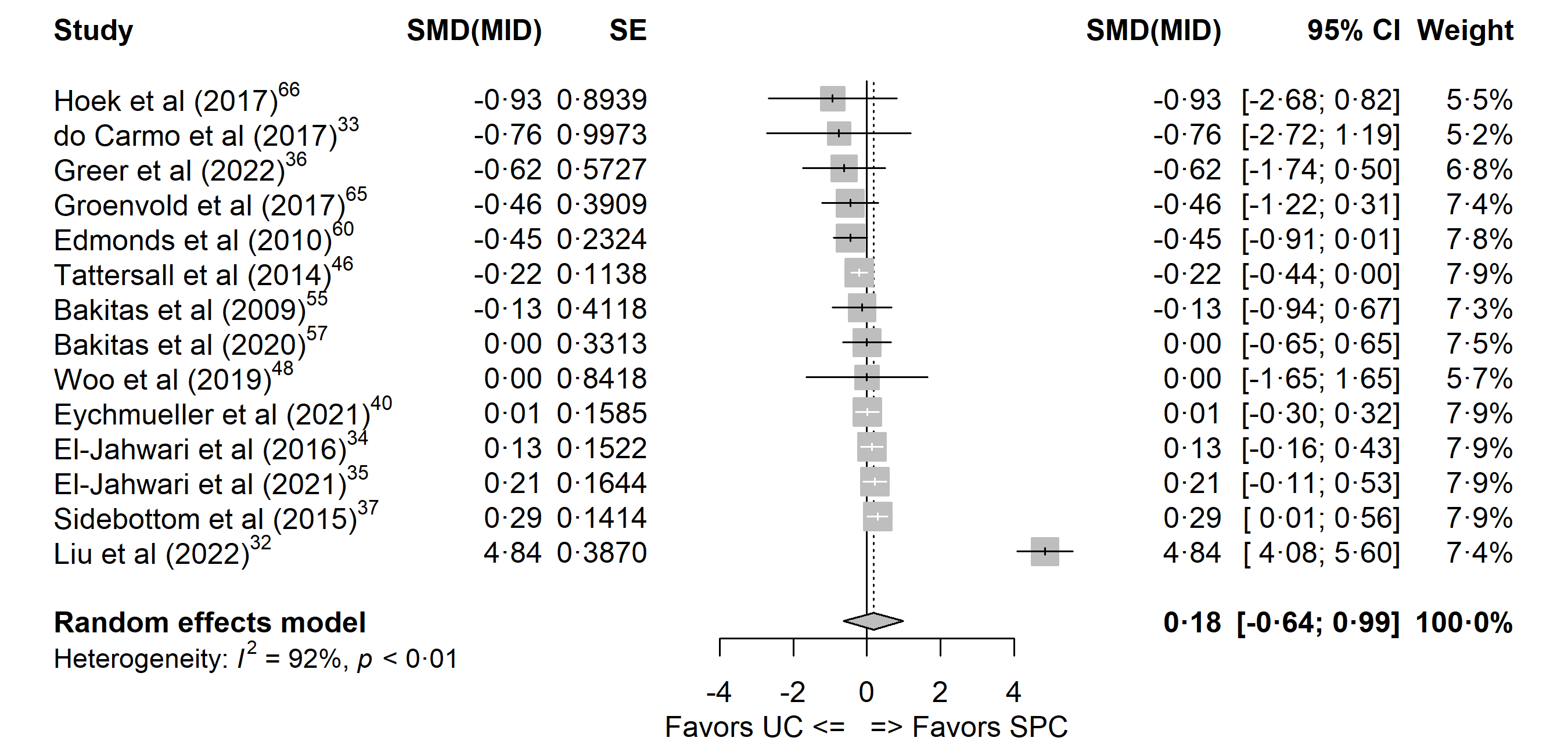


**Publication bias**

Egger’s enhanced funnel plot

| Linear regression test of funnel plot asymmetry  Intercept: 0.858  95% CI: -2.809 to 4.526  *t*(13) = 0.459, *p* = 0.655 | 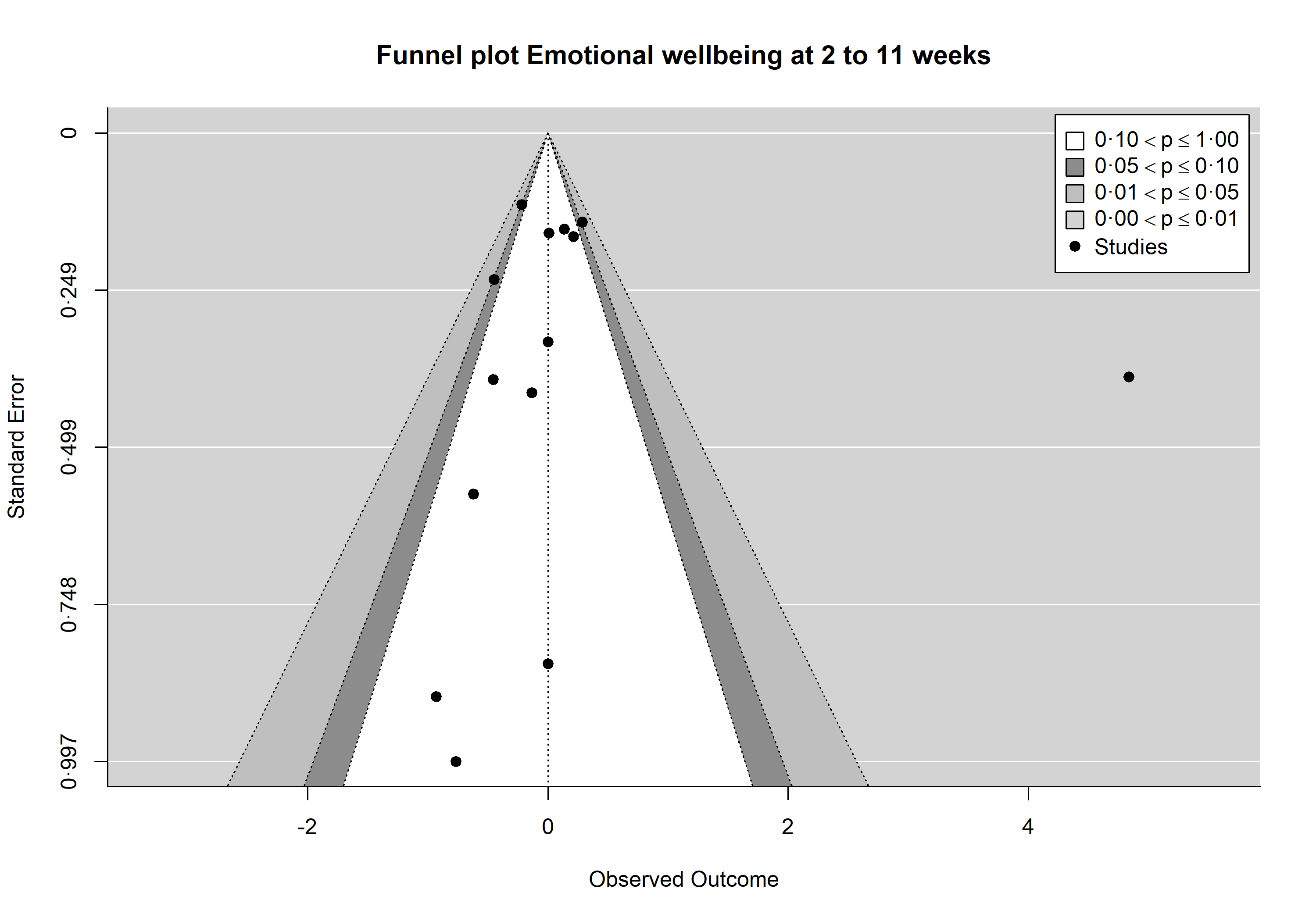 |
| --- | --- |

**Analysis with effect size RR (achieving change ≥1 MID)**

Number of studies combined: k = 14

Number of observations: 2073; Number of events: 670

| **Study (k = 14)** | **SPC**  **#1MID** | | **SPC**  **Total** | **UC**  **#1MID** | | **UC**  **Total** | **RR**  **(> 1MID)** | | **95% CI** | | **weight (random,**  **in %)** | |
| --- | --- | --- | --- | --- | --- | --- | --- | --- | --- | --- | --- | --- |
| Bakitas *et al* 2009^55^ | 44 | | 102 | 45 | | 98 | 0.94 | | 0.69 to 1.28 | | 12.1 | |
| Bakitas *et al* 2020^57^ | 42 | | 116 | 52 | | 142 | 0.99 | | 0.71 to 1.37 | | 11.7 | |
| do Carmo *et al* 2017^33^ | 4 | | 21 | 6 | | 19 | 0.60 | | 0.20 to 1.82 | | 2.9 | |
| Edmonds *et al* 2010^60^ | 1 | | 19 | 2 | | 16 | 0.42 | | 0.04 to 4.23 | | 0.8 | |
| El-Jahwari *et al* 2016^34^ | 3 | | 80 | 2 | | 77 | 1.44 | | 0.25 to 8.41 | | 1.3 | |
| El-Jahwari *et al* 2021^35^ | 12 | | 78 | 8 | | 69 | 1.33 | | 0.58 to 3.05 | | 4.5 | |
| Eychmueller *et al* 2021^40^ | 9 | | 61 | 9 | | 68 | 1.11 | | 0.47 to 2.63 | | 4.3 | |
| Greer *et al* 2022^52^ | 20 | | 54 | 25 | | 53 | 0.79 | | 0.50 to 1.23 | | 9.3 | |
| Groenvold *et al* 2017^65^ | 45 | | 130 | 54 | | 137 | 0.88 | | 0.64 to 1.20 | | 12.0 | |
| Hoek *et al* 2017^36^ | 8 | | 23 | 12 | | 26 | 0.75 | | 0.38 to 1.51 | | 5.7 | |
| Liu *et al* 2022^32^ | 81 | | 83 | 37 | | 83 | 2.19 | | 1.72 to 2.79 | | 13.5 | |
| Sidebottom *et al* 2015^67^ | 29 | | 86 | 20 | | 89 | 1.50 | | 0.92 to 2.44 | | 8.6 | |
| Tattersall *et al* 2014^44^ | 2 | | 58 | 6 | | 57 | 0.33 | | 0.07 to 1.56 | | 1.6 | |
| Woo *et al* 2019^46^ | 47 | | 112 | 45 | | 116 | 1.08 | | 0.79 to 1.48 | | 11.9 | |
|  | |  | | |  | | |  | |  | |  |
| ***Meta-analysis*** | | **RR** | | | **95% CI** | | | ***t*** | | ***p*** | |  |
| Random effects model | | 1.07 | | | 0.85 to 1.35 | | | 0.670 | | 0.517 | |  |
|  | |  | | |  | | |  | |  | |  |
| ***Heterogeneity*** | |  | | |  | | | ***Q (df)*** | | ***p*** | |  |
| *τ²* | | 0.07 | | | 0.02 to 0.33 | | | 42.51 (13) | | **<0.001** | |  |
| *I²* | | 69.4% | | | 47.0 to 82.4% | | |  | |  | |  |
| *H* | | 1.81 | | | 1.37 to 2.38 | | |  | |  | |  |

**Forest plot of RR effect size for the QoL outcome 2 to 11 weeks**


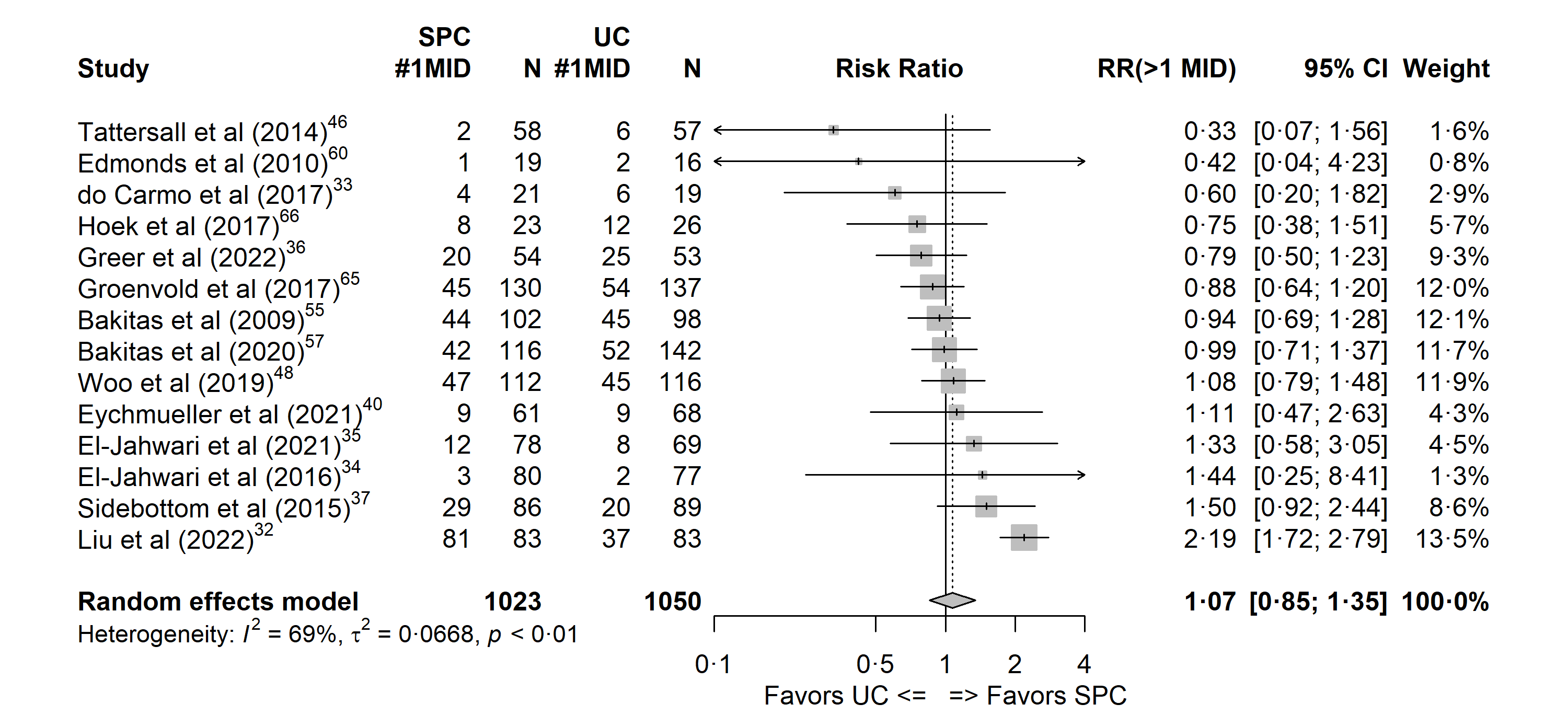


The risk ratio of RR = 1.07 (95% CI = 0.85 to 1.35) translates into a point estimate of a 7% increased probability of experiencing a change in emotional wellbeing of at least 1 MID size with SPC. This is statistically not significant.

The number needed to treat is calculated as follows (*p_CG_* is the baseline risk of experiencing change of at least 1 MID in the control group):

$$\frac{1}{p_{CG}\cdot(1-RR)}=\frac{1}{\frac{323}{1050}\cdot0.07}=46.440 \to46$$

The NNT is 46, meaning that 46 people need to be treated with SPC in order for one person to have a change in emotional wellbeing at 2 to 11 weeks of at least 1 MID.

**Meta-regression: Univariate meta-regression analyses with covariates**

| *k = 14* | **Regression** | | | | | **Heterogeneity** | | | **Test of moderators** | |
| --- | --- | --- | --- | --- | --- | --- | --- | --- | --- | --- |
|  | *b* | *SE* | *t* | *p* | 95% CI | *I²* | *Q* | *p* | *F* | *p* |
| ***Attrition (in %)*** |  |  |  |  |  |  |  |  |  |  |
| Intercept | 1.19 | 0.60 | 1.975 | *0.072* | - | 96 | 145.1 | **0.000** | 4.041 | *0.067* |
| Attrition (in %) | -0.04 | 0.02 | -2.010 | *0.067* | -0.08; -0.00 |  |  |  |  |  |
| ***% advanced disease*** |  |  |  |  |  |  |  |  |  |  |
| Intercept | -1.52 | 1.76 | -0.861 | 0.438 | - | 99 | 155.3 | **0.000** | 1.947 | 0.235 |
| % advanced disease | 0.04 | 0.03 | 1.396 | 0.235 | -0.04; 0.11 |  |  |  |  |  |
| ***Disease group (ref: Cancer)*** |  |  |  |  |  |  |  |  |  |  |
| Intercept | 0.25 | 0.45 | 0.551 | 0.592 | - | 97 | 172.0 | **0.000** | 0.106 | 0.753 |
| Non-cancer | -0.30 | 0.93 | -0.323 | 0.753 | -2.32; 1.72 |  |  |  |  |  |
| ***RoB2 score (ref: low risk)*** |  |  |  |  |  |  |  |  |  |  |
| Intercept | 1.76 | 0.73 | 2.398 | **0.035** | - | 96 | 113.3 | **0.000** | 2.923 | *0.096* |
| RoB2: Some risk | -1.89 | 0.86 | -2.201 | *0.050* | -3.79; 0.00 |  |  |  |  |  |
| RoB2: High risk | -2.13 | 0.98 | -2.174 | *0.052* | -4.29; 0.03 |  |  |  |  |  |
| ***Service composition score*** |  |  |  |  |  |  |  |  |  |  |
| Intercept | -3.06 | 0.73 | -4.198 | **0.001** | - | 92 | 103.5 | **0.000** | 22.336 | **<0.001** |
| Service composition score | 0.29 | 0.06 | 4.726 | **0.001** | 0.16; 0.43 |  |  |  |  |  |
| ***Setting (ref: multiple settings)*** |  |  |  |  |  |  |  |  |  |  |
| Intercept | -0.37 | 0.64 | -0.574 | 0.577 | - | 97 | 144.4 | **0.000** | 0.914 | 0.429 |
| Inpatient consulting model | 1.12 | 0.86 | 1.305 | 0.219 | -0.77; 3.02 |  |  |  |  |  |
| Home or hospital outreach | 0.29 | 1.03 | 0.278 | 0.787 | -1.99; 2.57 |  |  |  |  |  |
| ***Type of intervention (ref: SPC)*** |  |  |  |  |  |  |  |  |  |  |
| Intercept | 0.60 | 0.63 | 0.954 | 0.363 | - | 97 | 155.8 | **0.000** | 0.300 | 0.825 |
| Early SPC | -0.86 | 0.94 | -0.917 | 0.381 | -2.96; 1.23 |  |  |  |  |  |
| Integrated collaborative care | -0.39 | 1.60 | -0.240 | 0.815 | -3.96; 3.19 |  |  |  |  |  |
| Nurse-led palliative care | -0.66 | 1.24 | -0.534 | 0.605 | -3.38; 2.10 |  |  |  |  |  |
| ***Year*** |  |  |  |  |  |  |  |  |  |  |
| Intercept | -1.83 | 1.58 | -1.157 | 0.269 | - | 97 | 150.7 | **0.000** | 1.709 | 0.216 |
| Year | 0.12 | 0.09 | 1.307 | 0.216 | -0.08; 0.31 |  |  |  |  |  |

.

**Bubble plots of univariate meta-regression analyses**

| **Attrition** | *F*(1,12) = 4.041  *p* = *0.067* | 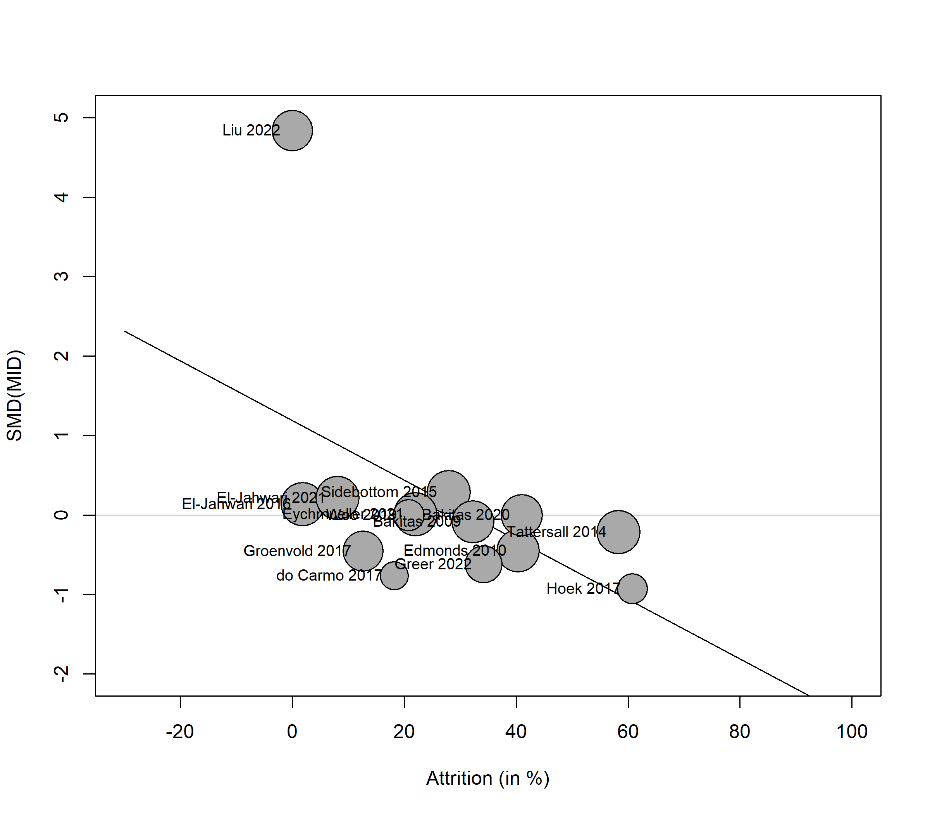 |
| --- | --- | --- |
| **% advanced disease** | *F*(1,4) = 1.947  *p* = 0.235 | 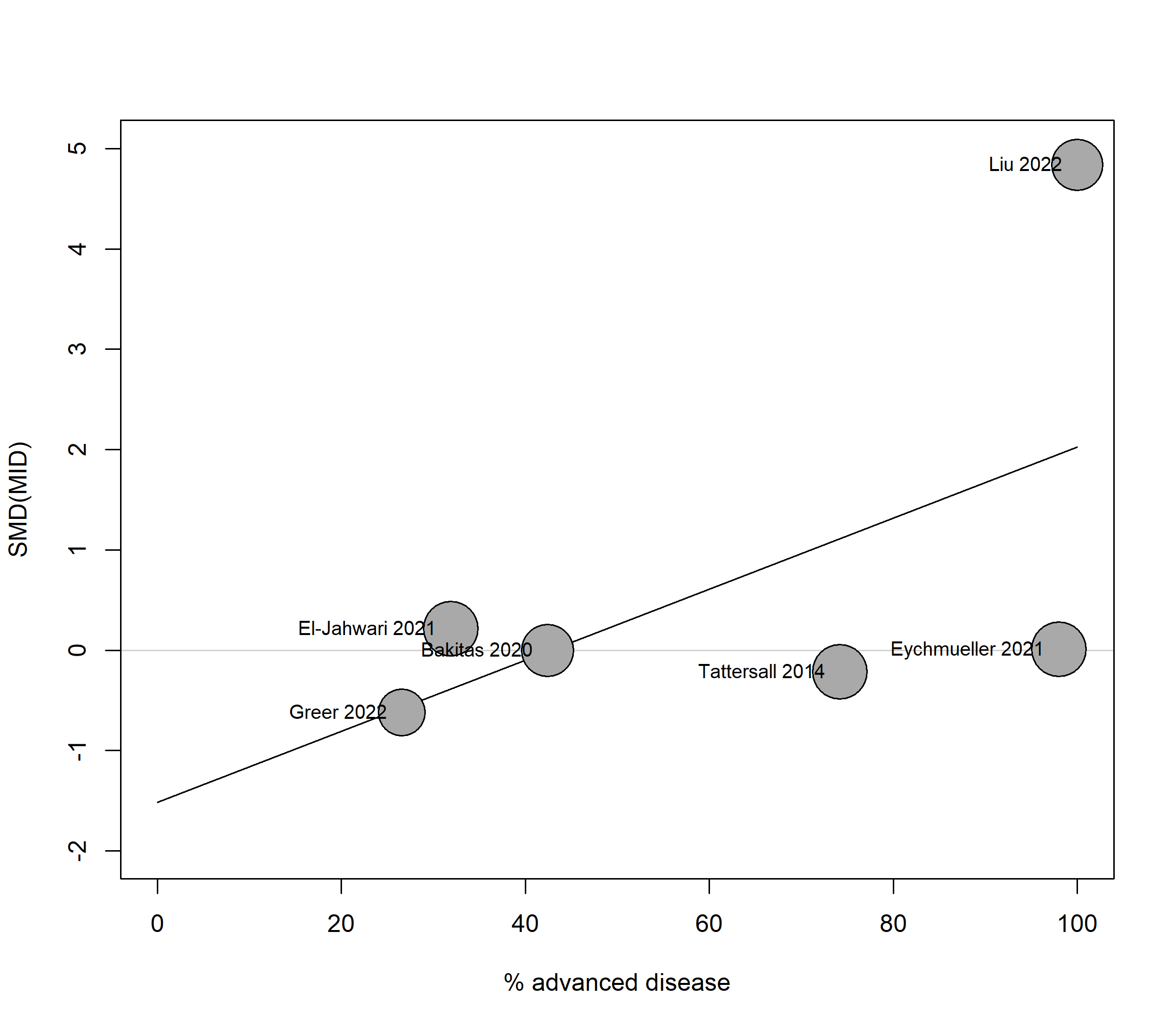 |
| **Disease group** | *F*(1,12) = 0.106  *p* = 0.753 | 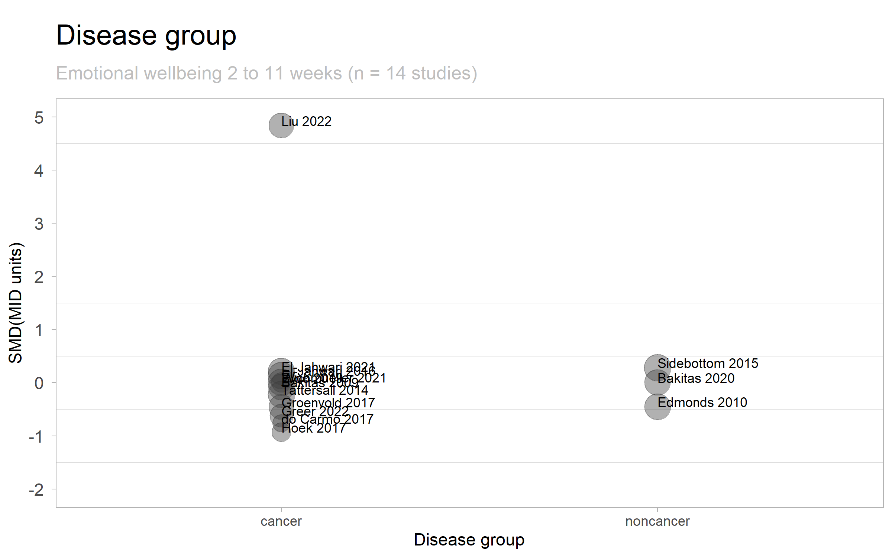 |
| **RoB2 score** | *F*(2,11) = 2.923  *p* = *0.096* | 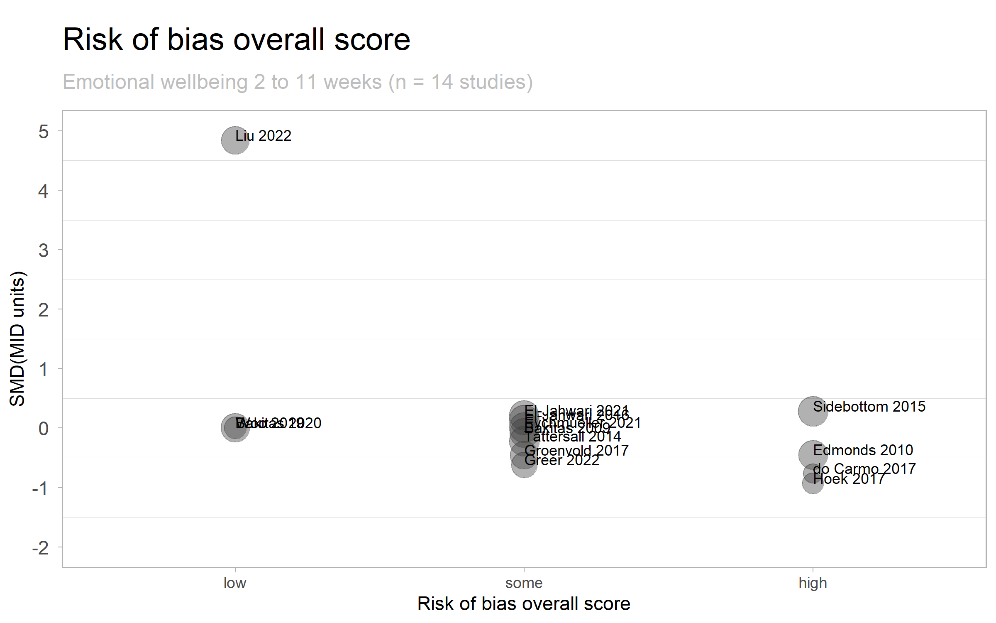 |
| **Service composition score** | *F*(1,12) = 22.336  *p* = <**0.001** | 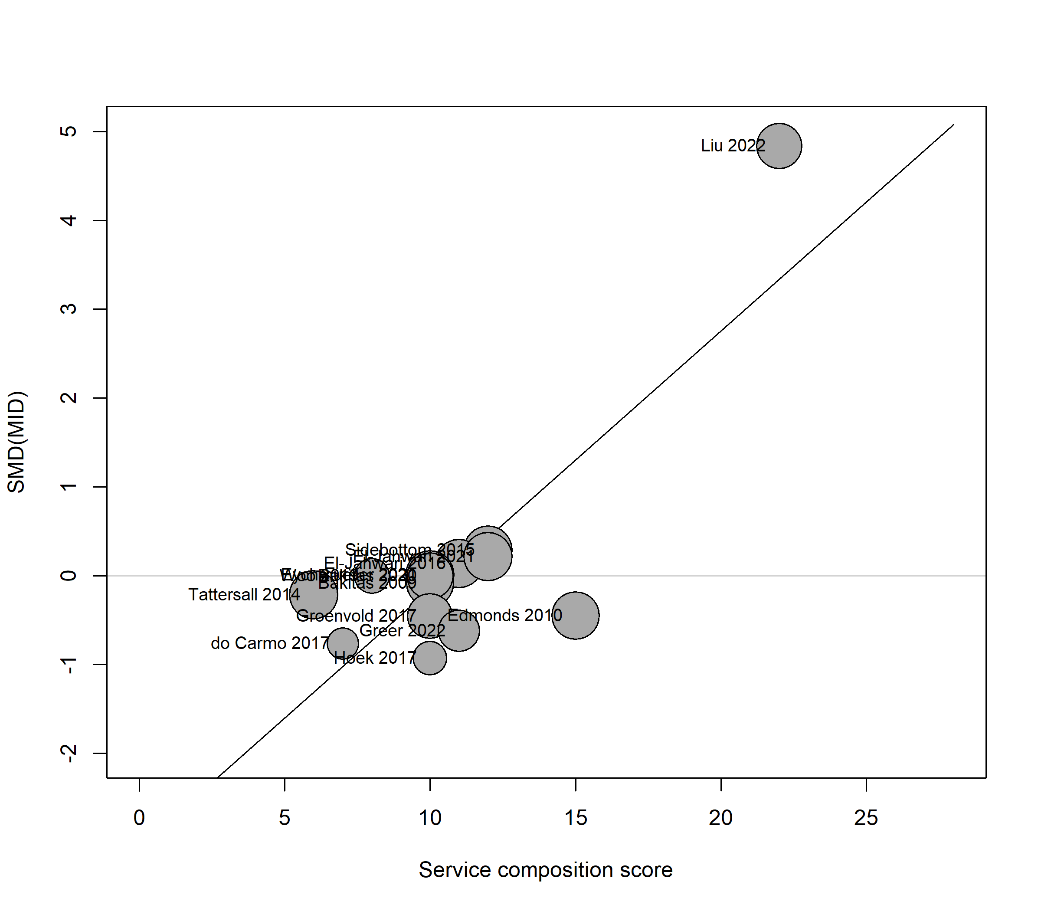 |
| **Setting** | *F*(2,11) = 0.914  *p* = 0.429 | 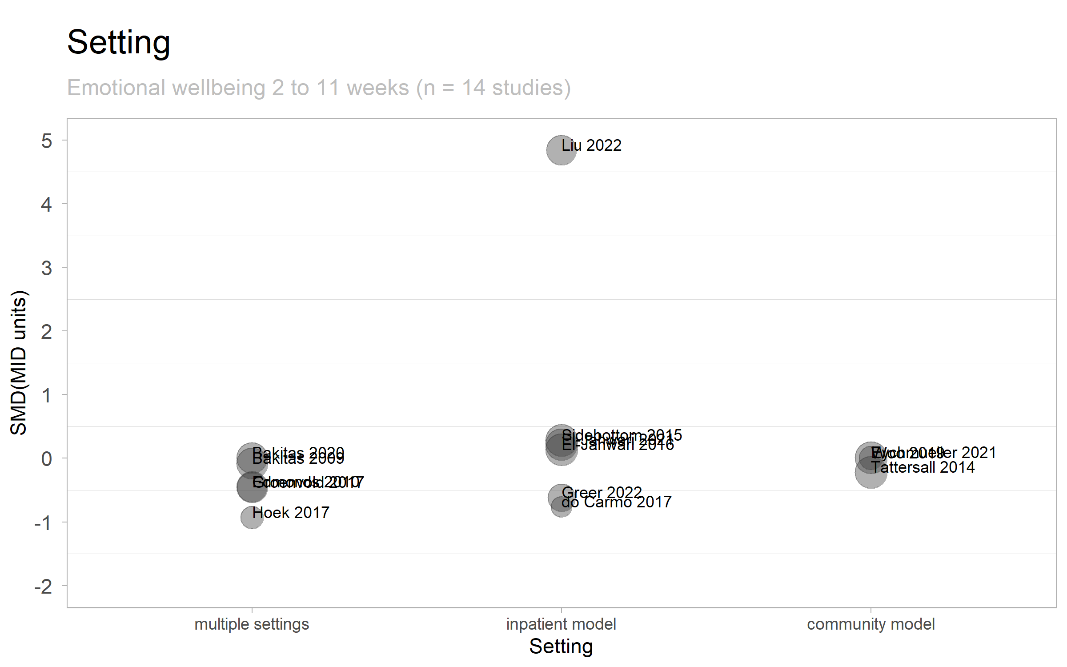 |
| **Type of intervention** | *F*(3,10) = 0.300  *p* = 0.825 | 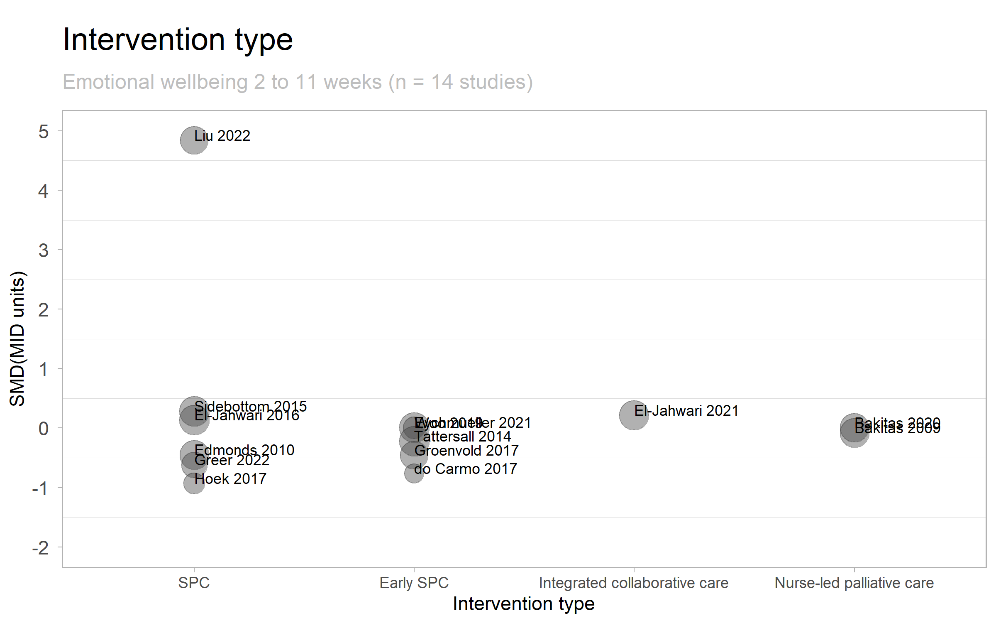 |
| **Year** | *F*(1,12) = 1.709  *p* = 0.216 | 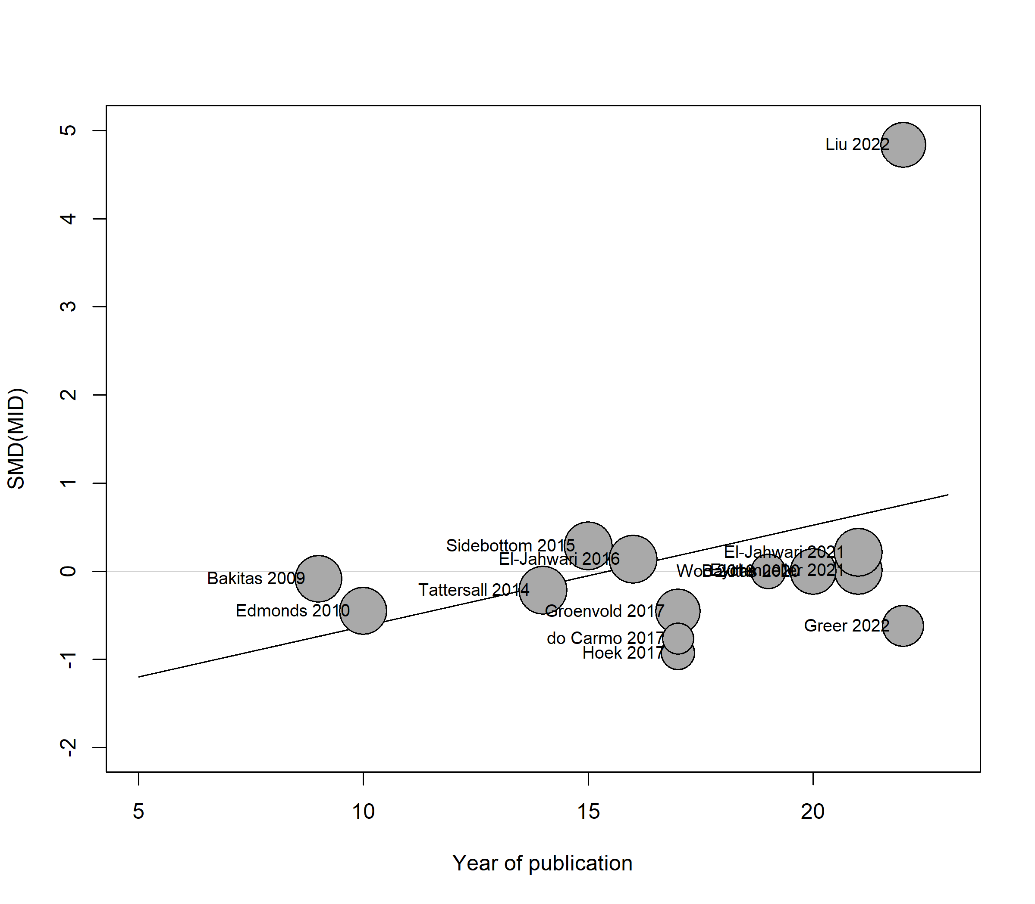 |

## Fig F and Table F: Emotional wellbeing at 12 weeks

**Analysis with effect size SMD (MID units)**

| **Study (k = 18)** | **SPC**  **MD_change_** | | **SPC**  **Total** | **UC**  **MD_change_** | | **UC**  **Total** | **SMD (MID)** | | **95% CI** | | **weight**  **(random,**  **in %)** | |
| --- | --- | --- | --- | --- | --- | --- | --- | --- | --- | --- | --- | --- |
| Aiken *et al* 2006^54^ | 0.59 | | 43 | -1.17 | | 30 | 0.13 | | 0.09 to 0.16 | | 16.1 | |
| Bakitas *et al* 2015^56^ | 3.79 | | 72 | 2.62 | | 83 | 0.39 | | -0.28 to 1.06 | | 3.3 | |
| Bekelman *et al* 2018^58^ | 2.10 | | 105 | 0.50 | | 105 | 0.32 | | -0.11 to 0.75 | | 6.2 | |
| Brims *et al* 2019^51^ | 0.60 | | 63 | 0.60 | | 57 | 0.00 | | -0.28 to 0.28 | | 9.7 | |
| do Carmo *et al* 2017^33^ | -1.95 | | 19 | -0.94 | | 18 | -0.20 | | -0.85 to 0.45 | | 3.5 | |
| Edmonds *et al* 2010^60^ | -0.10 | | 21 | 0.80 | | 17 | -0.11 | | -0.62 to 0.39 | | 5.1 | |
| El-Jahwari *et al* 2016^34^ | 0.98 | | 75 | -0.54 | | 74 | 0.30 | | 0.03 to 0.58 | | 9.7 | |
| El-Jahwari *et al* 2021^35^ | 2.20 | | 72 | 1.30 | | 65 | 0.68 | | -0.28 to 1.65 | | 1.8 | |
| Evans *et al* 2021^61^ | 1.43 | | 23 | 0.05 | | 24 | 0.35 | | -0.27 to 0.96 | | 3.8 | |
| Gao *et al* 2020^63^ | 0.35 | | 176 | -0.08 | | 174 | 0.33 | | -0.48 to 1.13 | | 2.5 | |
| Greer *et al* 2022^52^ | 0.13 | | 48 | 1.12 | | 47 | -0.75 | | -1.92 to 0.42 | | 1.3 | |
| Hoek *et al* 2017^36^ | -1.22 | | 13 | 1.16 | | 16 | -1.80 | | -4.09 to 0.48 | | 0.3 | |
| Rogers *et al* 2017^27^ | 0.70 | | 47 | 1.20 | | 43 | -0.38 | | -1.80 to 1.04 | | 0.9 | |
| Sidebottom *et al* 2015^67^ | 2.90 | | 79 | 2.18 | | 88 | 0.14 | | -0.14 to 0.43 | | 9.6 | |
| Slama *et al* 2020^68^ | -1.00 | | 51 | -1.00 | | 51 | 0.00 | | -1.38 to 1.38 | | 0.9 | |
| Tattersall *et al* 2014^44^ | -1.30 | | 49 | 2.20 | | 57 | -0.33 | | -0.56 to -0.11 | | 11.3 | |
| Temel *et al* 2020^69^ | 1.13 | | 100 | 0.32 | | 111 | 0.61 | | 0.05 to 1.18 | | 4.3 | |
| Woo *et al* 2019^46^ | -0.10 | | 72 | 0.00 | | 78 | -0.03 | | -0.30 to 0.24 | | 9.9 | |
|  | |  | | |  | | |  | |  | |  |
| ***Meta-analysis*** | | **SMD (MID)** | | | **95% CI** | | | ***t*** | | ***p*** | |  |
| Random effects model | | 0.08 | | | -0.06 to 0.23 | | | 1.180 | | 0.254 | |  |
|  | |  | | |  | | |  | |  | |  |
| ***Heterogeneity*** | |  | | |  | | | ***Q (df)*** | | ***p*** | |  |
| *τ²* | | 0.03 | | | 0.00 to 0.23 | | | 33.28 (17) | | **0.010** | |  |
| *I²* | | 48.9% | | | 11.9 to 70.4% | | |  | |  | |  |
| *H* | | 1.40 | | | 1.07 to 1.84 | | |  | |  | |  |

**Forest plot**


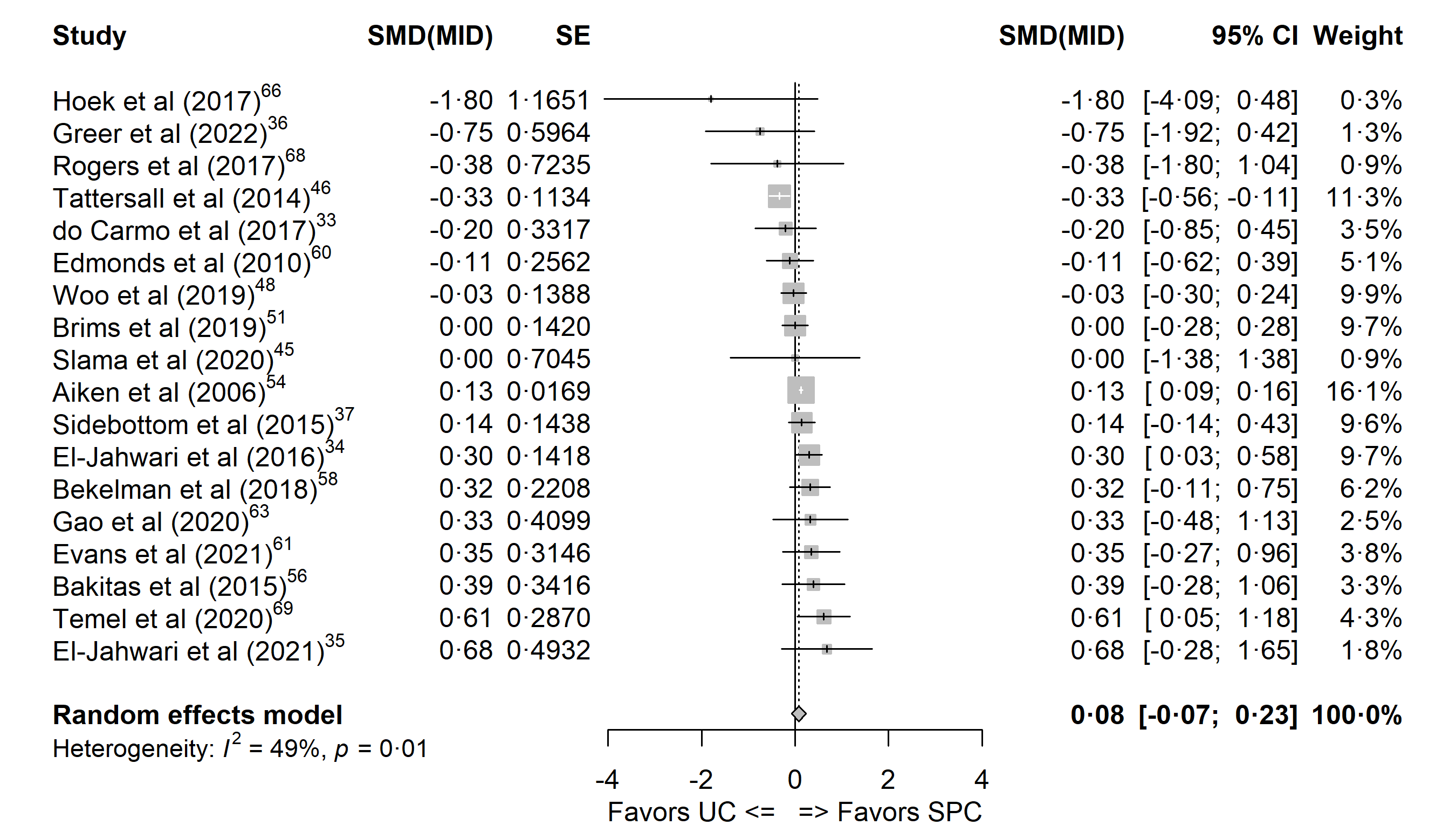


**Publication bias**

Egger’s enhanced funnel plot

| Linear regression test of funnel plot asymmetry  Intercept: -0.277  95% CI: -1.025 to 0.471  *t*(17) = -0.725, *p* = 0.479 | 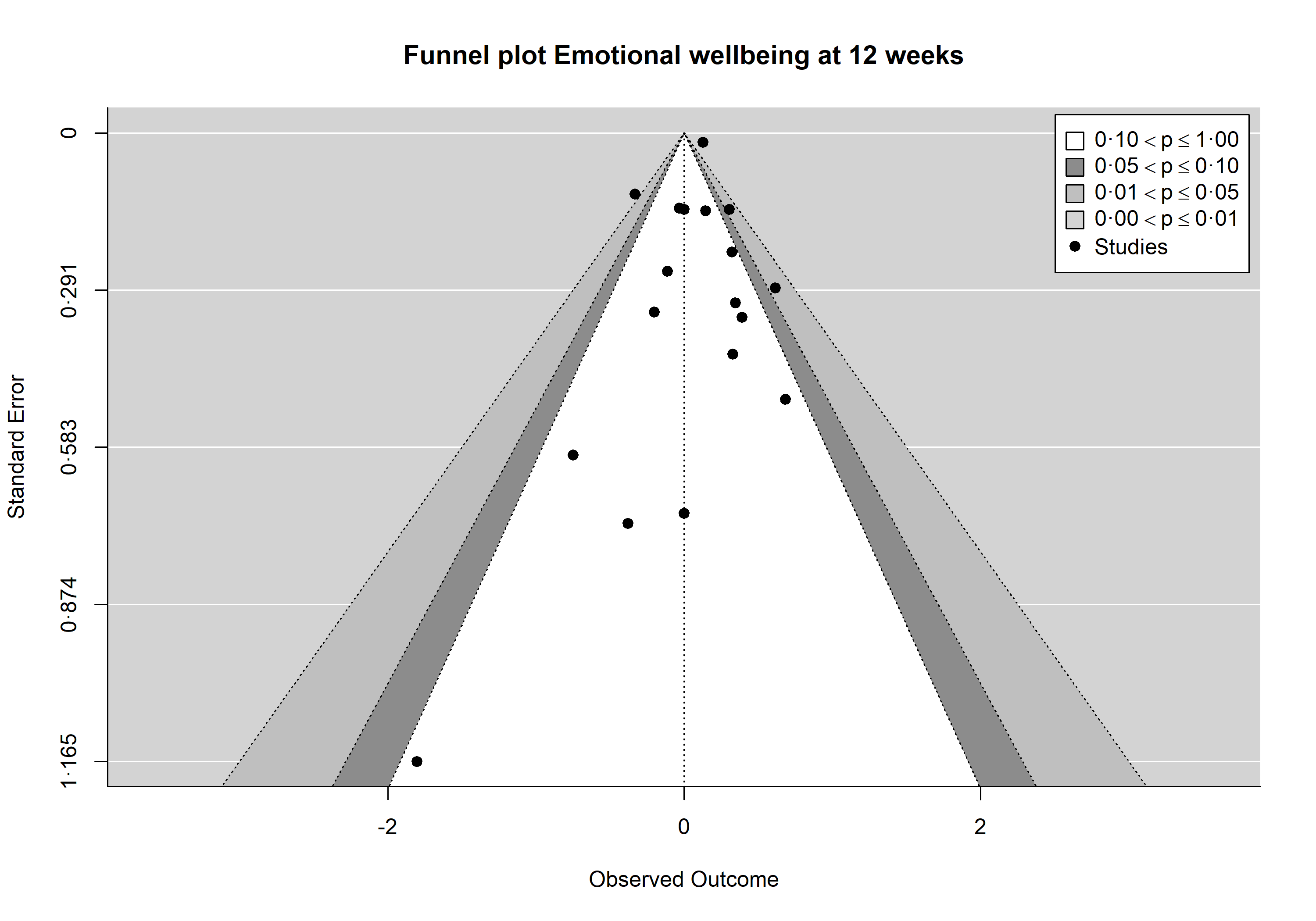 |
| --- | --- |

**Analysis with effect size RR (achieving change ≥1 MID)**

Number of studies combined: k = 18

Number of observations: 2266; Number of events: 703

| **Study (k = 18)** | **SPC**  **#1MID** | | **SPC**  **Total** | **UC**  **#1MID** | | **UC**  **Total** | **RR**  **(> 1MID)** | | **95% CI** | | **weight (random,**  **in %)** | |
| --- | --- | --- | --- | --- | --- | --- | --- | --- | --- | --- | --- | --- |
| Aiken *et al* 2006^54^ | 0.5 | | 43 | 0.5 | | 30 | 0.70 | | 0.01 to 34.20 | | 0.1 | |
| Bakitas *et al* 2015^56^ | 39 | | 72 | 39 | | 83 | 1.15 | | 0.84 to 1.57 | | 13.6 | |
| Bekelman *et al* 2018^58^ | 38 | | 105 | 30 | | 105 | 1.27 | | 0.85 to 1.88 | | 8.5 | |
| Brims *et al* 2019^51^ | 8 | | 63 | 8 | | 57 | 0.90 | | 0.36 to 2.25 | | 1.6 | |
| do Carmo *et al* 2017^33^ | 2 | | 19 | 1 | | 18 | 1.89 | | 0.19 to 19.13 | | 0.2 | |
| Edmonds *et al* 2010^60^ | 2 | | 21 | 2 | | 17 | 0.81 | | 0.13 to 5.16 | | 0.4 | |
| El-Jahwari *et al* 2016^34^ | 13 | | 75 | 8 | | 74 | 1.60 | | 0.71 to 3.64 | | 2.0 | |
| El-Jahwari *et al* 2021^35^ | 42 | | 72 | 32 | | 65 | 1.18 | | 0.86 to 1.62 | | 13.4 | |
| Evans *et al* 2021^61^ | 5 | | 23 | 5 | | 24 | 1.04 | | 0.35 to 3.13 | | 1.1 | |
| Gao *et al* 2020^63^ | 75 | | 176 | 67 | | 174 | 1.11 | | 0.86 to 1.43 | | 20.5 | |
| Greer *et al* 2022^52^ | 17 | | 48 | 23 | | 47 | 0.72 | | 0.45 to 1.17 | | 5.7 | |
| Hoek *et al* 2017^36^ | 4 | | 13 | 8 | | 16 | 0.62 | | 0.24 to 1.59 | | 1.5 | |
| Rogers *et al* 2017^27^ | 21 | | 47 | 21 | | 43 | 0.91 | | 0.59 to 1.42 | | 6.8 | |
| Sidebottom *et al* 2015^67^ | 26 | | 79 | 23 | | 88 | 1.26 | | 0.79 to 2.02 | | 6.0 | |
| Slama *et al* 2020^68^ | 17 | | 51 | 17 | | 51 | 1.00 | | 0.58 to 1.73 | | 4.4 | |
| Tattersall *et al* 2014^44^ | 1 | | 49 | 5 | | 57 | 0.23 | | 0.03 to 1.92 | | 0.3 | |
| Temel *et al* 2020^69^ | 47 | | 100 | 40 | | 111 | 1.30 | | 0.94 to 1.80 | | 12.7 | |
| Woo *et al* 2019^46^ | 11 | | 72 | 5 | | 78 | 2.38 | | 0.87 to 6.53 | | 1.3 | |
|  | |  | | |  | | |  | |  | |  |
| ***Meta-analysis*** | | **RR** | | | **95% CI** | | | ***t*** | | ***p*** | |  |
| Random effects model | | 1.12 | | | 1.01 to 1.25 | | | 2.230 | | **0.039** | |  |
|  | |  | | |  | | |  | |  | |  |
| ***Heterogeneity*** | |  | | |  | | | ***Q (df)*** | | ***p*** | |  |
| *τ²* | | 0.00 | | | 0.00 to 0.09 | | | 12.90 (17) | | 0.743 | |  |
| *I²* | | 0.0% | | | 0.0 to 50.0% | | |  | |  | |  |
| *H* | | 1.00 | | | 1.00 to 1.41 | | |  | |  | |  |

**Forest plot of RR effect size for the QoL outcome at 12 weeks**


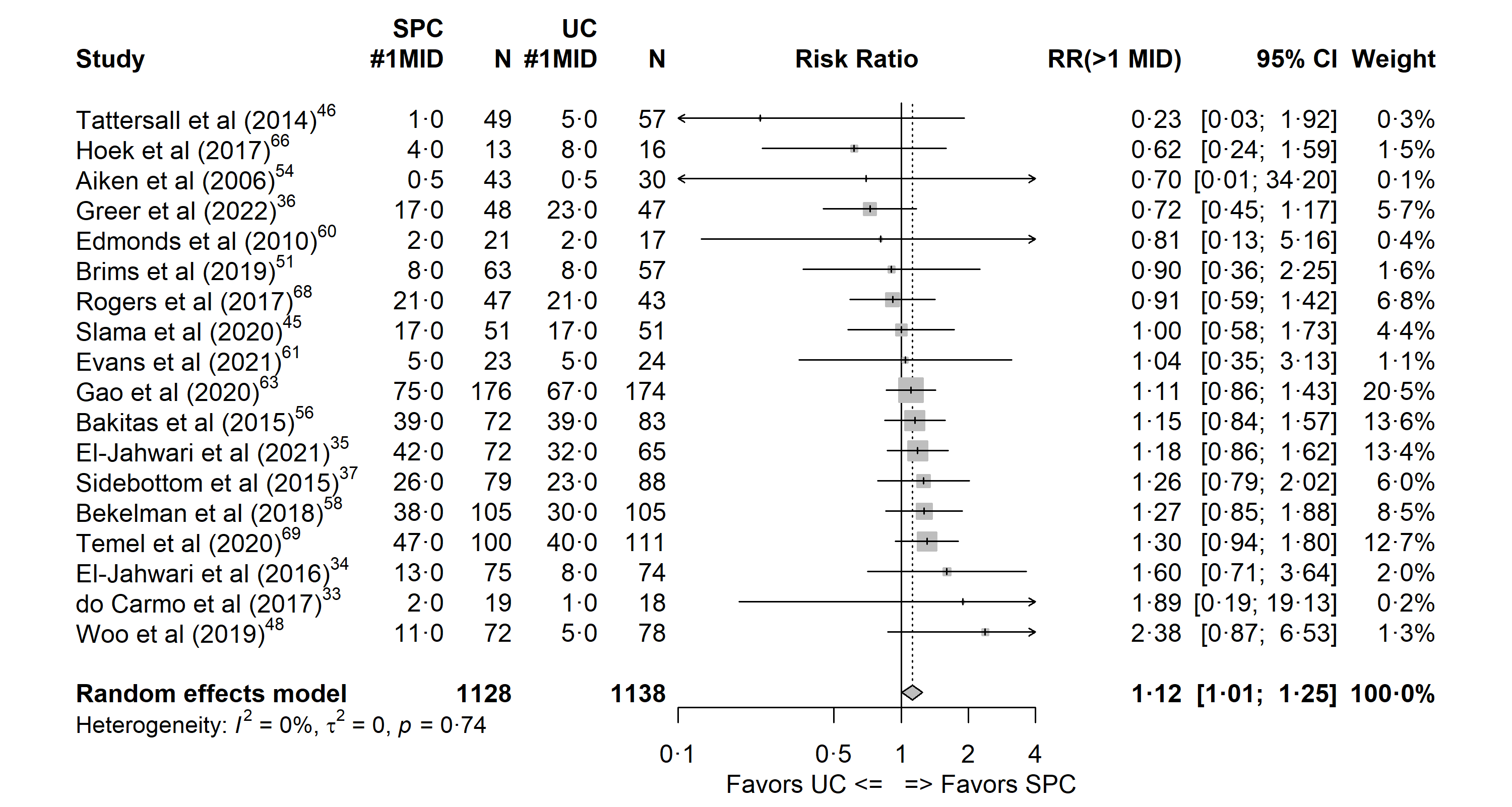


The risk ratio of RR = 1.12 (95% CI = 1.00 to 1.25) translates into a point estimate of a 12% increased probability of experiencing a change in emotional wellbeing of at least 1 MID size with SPC. This is statistically significant.

The number needed to treat is calculated as follows (*p_CG_* is the baseline risk of experiencing change of at least 1 MID in the control group):

$$\frac{1}{p_{CG}\cdot(1-RR)}=\frac{1}{\frac{335}{1138}\cdot0.12}=28.308\to28$$

The NNT is 28, meaning that 28 people need to be treated with SPC in order for one person to have a change in emotional wellbeing at 12 weeks of at least 1 MID.

**Meta-regression: Univariate meta-regression analyses with covariates**

| *k = 18* | **Regression** | | | | | **Heterogeneity** | | | **Test of moderators** | |
| --- | --- | --- | --- | --- | --- | --- | --- | --- | --- | --- |
|  | *b* | *SE* | *t* | *p* | 95% CI | *I²* | *Q* | *p* | *F* | *p* |
| ***Attrition (in %)*** |  |  |  |  |  |  |  |  |  |  |
| Intercept | 0.23 | 0.12 | 1.847 | *0.083* | - | 45 | 33.2 | **0.007** | 1.991 | 0.177 |
| Attrition (in %) | -0.00 | 0.00 | -1.411 | 0.177 | -0.01; 0.00 |  |  |  |  |  |
| ***% advanced disease*** |  |  |  |  |  |  |  |  |  |  |
| Intercept | 0.18 | 0.36 | 0.491 | 0.638 | - | 47 | 13.7 | 0.056 | 0.140 | 0.719 |
| % advanced disease | -0.00 | 0.01 | -0.374 | 0.719 | -0.02; 0.01 |  |  |  |  |  |
| ***Disease group (ref: Cancer)*** |  |  |  |  |  |  |  |  |  |  |
| Intercept | 0.03 | 0.09 | 0.325 | 0.749 | - | 44 | 28.2 | **0.029** | 0.689 | 0.419 |
| Non-cancer | 0.12 | 0.14 | 0.830 | 0.419 | -0.18; 0.42 |  |  |  |  |  |
| ***RoB2 score (ref: low risk)*** |  |  |  |  |  |  |  |  |  |  |
| Intercept | 0.15 | 0.18 | 0.827 | 0.421 | - | 53 | 31.2 | **0.008** | 0.287 | 0.755 |
| RoB2: Some risk | -0.03 | 0.22 | -0.145 | 0.887 | -0.49; 0.43 |  |  |  |  |  |
| RoB2: High risk | -0.14 | 0.23 | -0.635 | 0.535 | -0.63; 0.34 |  |  |  |  |  |
| ***Service composition score*** |  |  |  |  |  |  |  |  |  |  |
| Intercept | -0.31 | 0.21 | -1.477 | 0.159 | - | 31 | 24.4 | 0.081 | 3.754 | *0.065* |
| Service composition score | 0.04 | 0.02 | 1.938 | *0.065* | -0.00; 0.07 |  |  |  |  |  |
| ***Setting (ref: multiple settings)*** |  |  |  |  |  |  |  |  |  |  |
| Intercept | 0.17 | 0.07 | 2.223 | **0.042** | - | 17 | 19.2 | 0.207 | 3.683 | **0.049** |
| Inpatient consulting model | -0.00 | 0.14 | -0.023 | 0.982 | -0.29; 0.29 |  |  |  |  |  |
| Home or hospital outreach | -0.31 | 0.12 | -2.539 | **0.023** | -0.58; -0.05 |  |  |  |  |  |
| ***Type of intervention (ref: SPC)*** | | | | | | | | | | |
| Intercept | 0.09 | 0.13 | 0.737 | 0.473 | - | 29 | 20.6 | 0.112 | 1.390 | 0.287 |
| Early SPC | -0.16 | 0.17 | -0.945 | 0.361 | -0.52; 0.20 |  |  |  |  |  |
| Integrated collaborative care | 0.23 | 0.22 | 1.062 | 0.306 | -0.24; 0.71 |  |  |  |  |  |
| Nurse-led palliative care | 0.07 | 0.19 | 0.347 | 0.734 | -0.35; 0.48 |  |  |  |  |  |
| ***Year*** |  |  |  |  |  |  |  |  |  |  |
| Intercept | -0.05 | 0.25 | -0.188 | 0.853 | - | 46 | 32.0 | **0.010** | 0.301 | 0.591 |
| Year | 0.01 | 0.02 | 0.549 | 0.591 | -0.02; 0.04 |  |  |  |  |  |

.

**Bubble plots of univariate meta-regression analyses**

| **Attrition** | *F*(1,15) = 1.991  *p* = 0.177 | 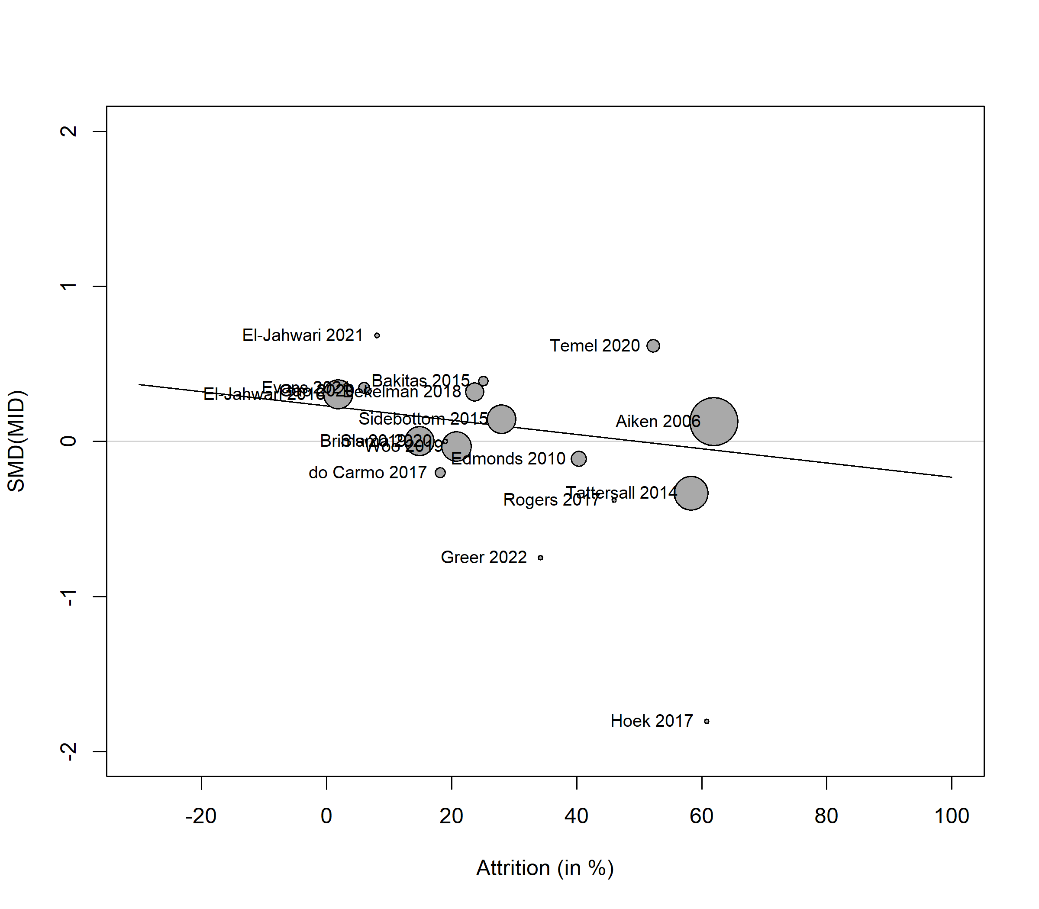 |
| --- | --- | --- |
| **% advanced disease** | *F*(1,7) = 0.140  *p* = 0.719 | 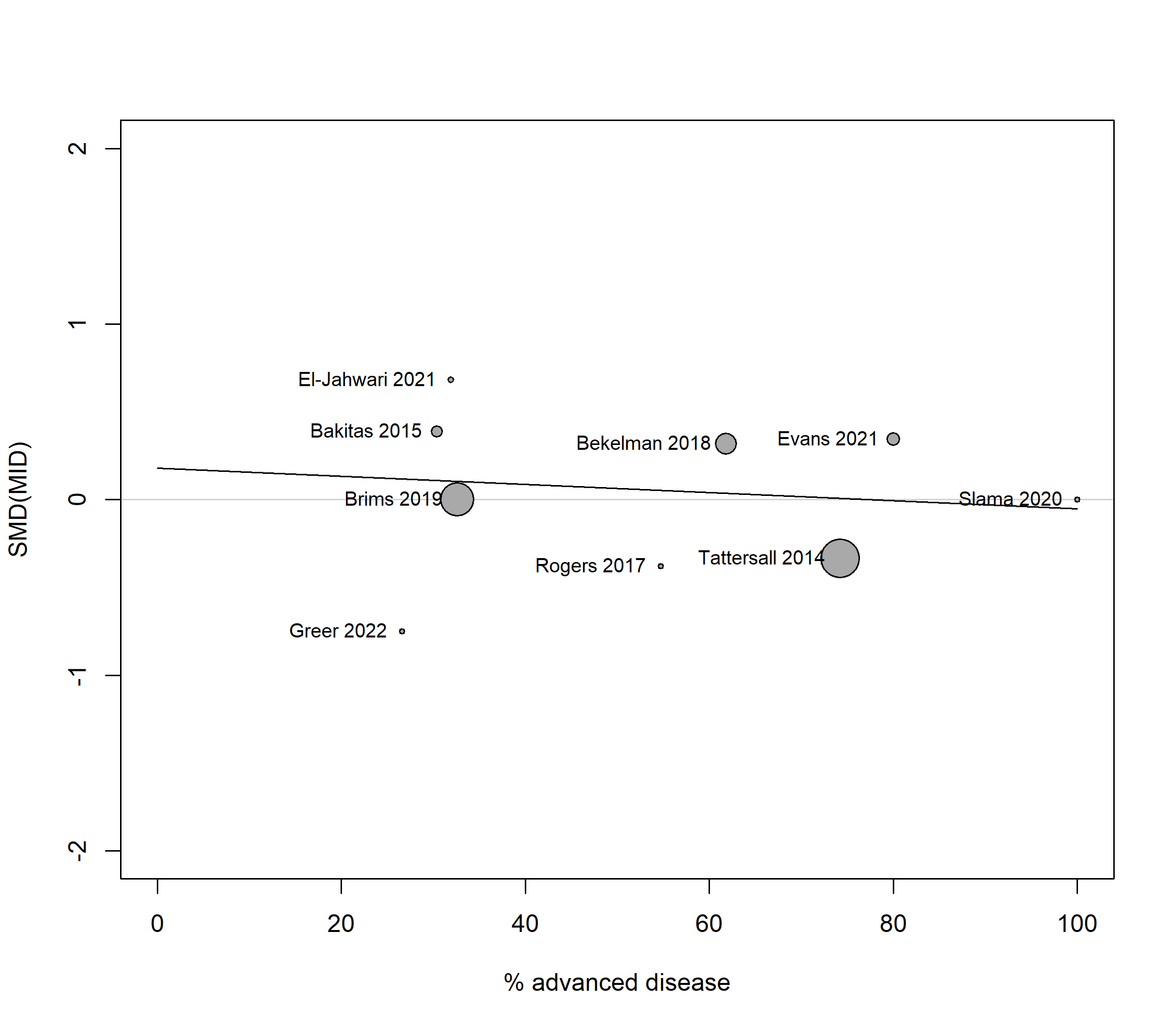 |
| **Disease group** | *F*(1,15) = 0.689  *p* = 0.419 | 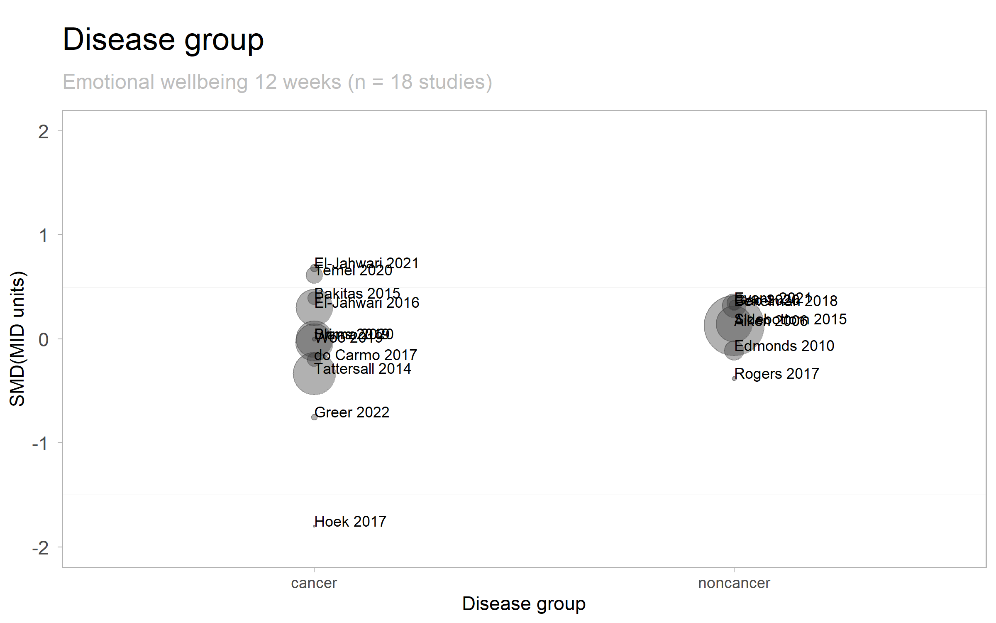 |
| **RoB2 score** | *F*(2,14) = 0.287  *p* = 0.755 | 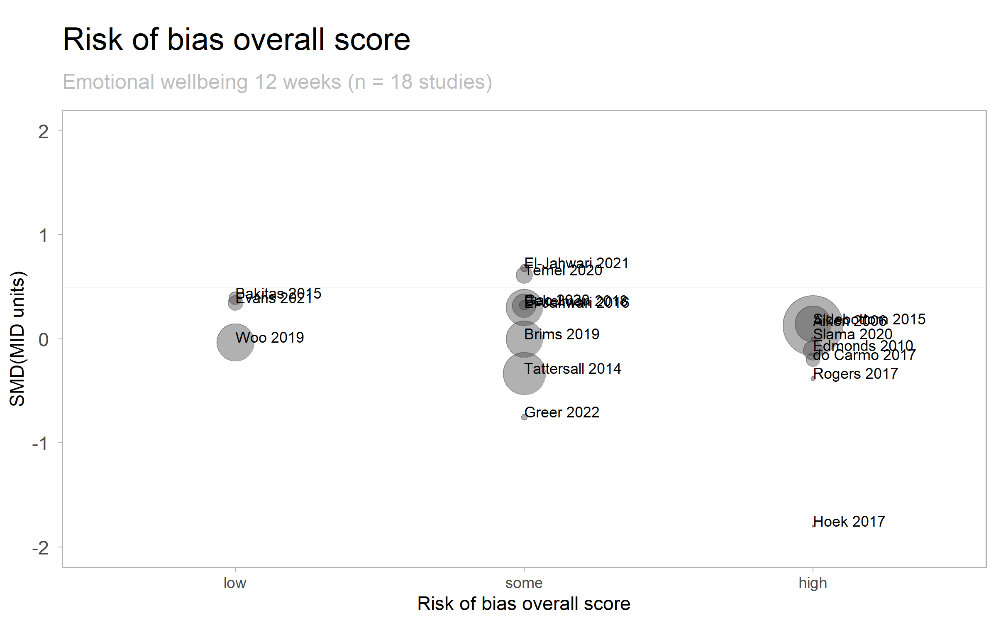 |
| **Service composition score** | *F*(1,15) = 3.754  *p* = *0.065* | 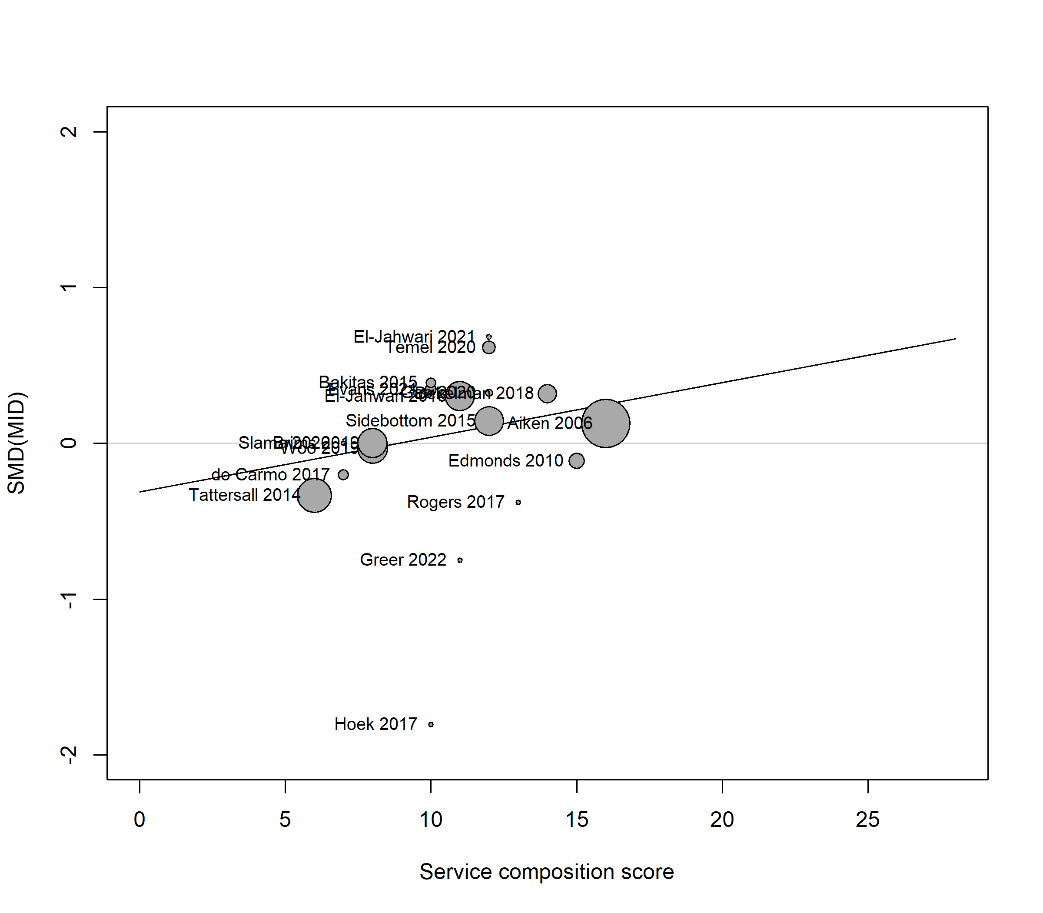 |
| **Setting** | *F*(2,14) = 3.683  *p* = **0.049** | 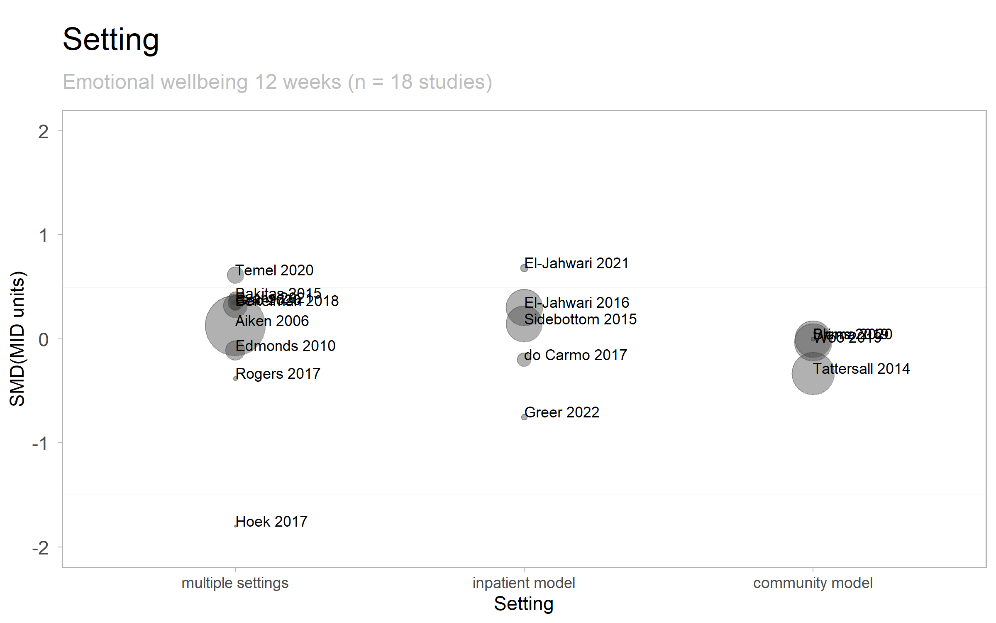 |
| **Type of intervention** | *F*(3,13) = 1.390  *p* = 0.287 | 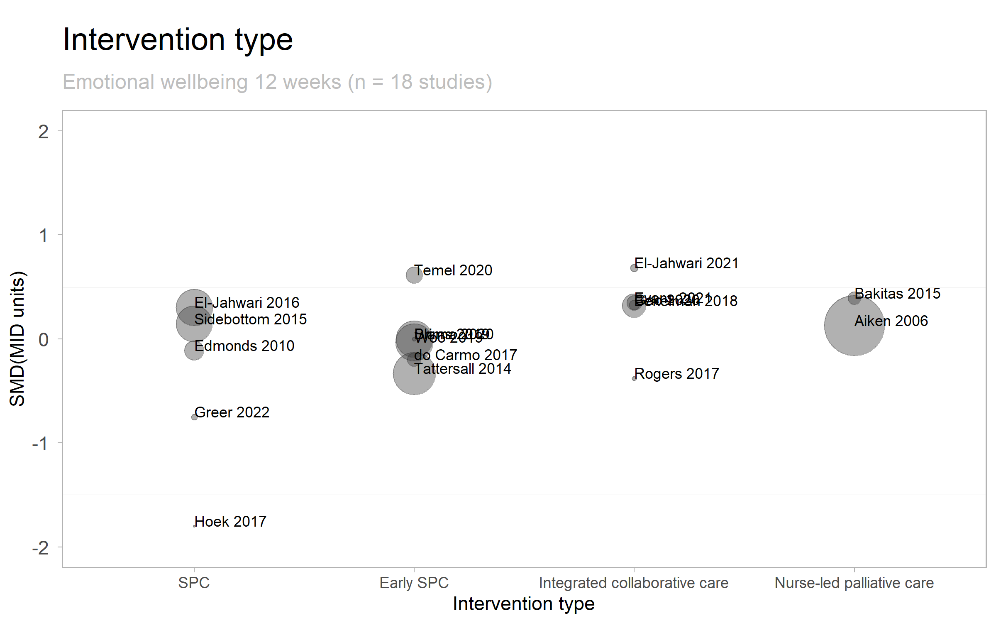 |
| **Year** | *F*(1,15) = 0.301  *p* = 0.591 | 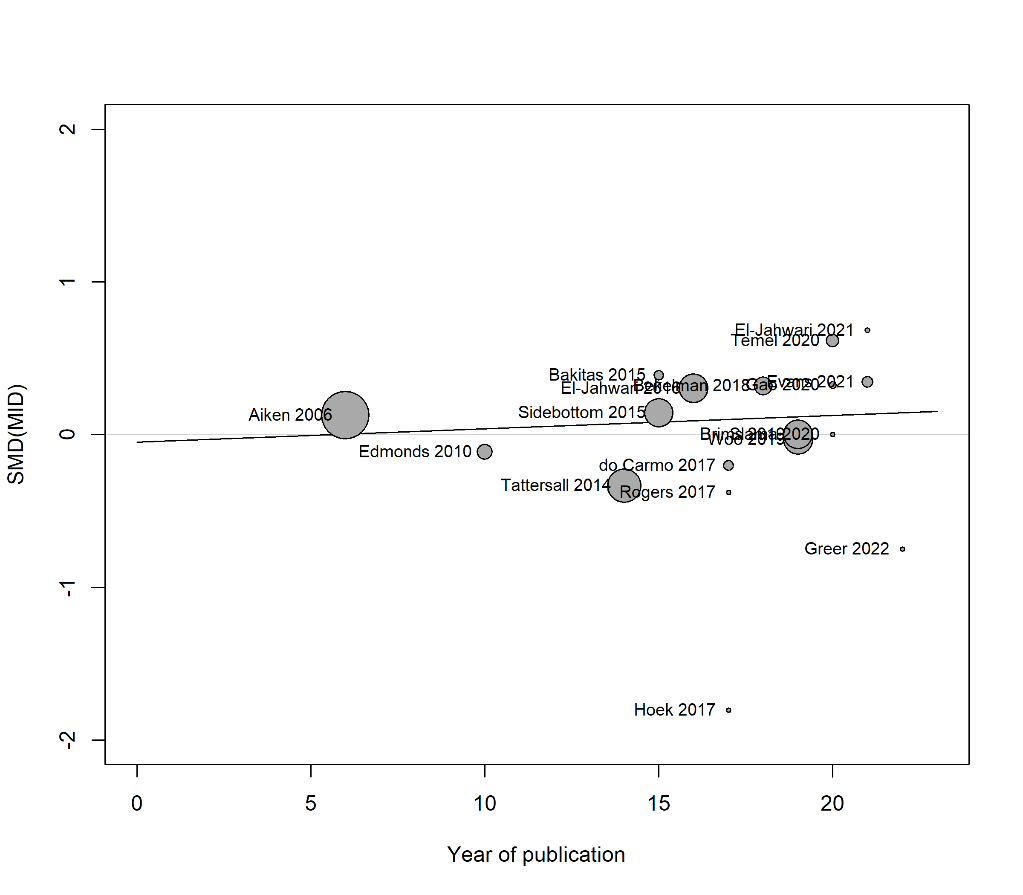 |

## Fig G and Table G: Emotional wellbeing at 13 to 36 weeks

**Analysis with effect size SMD (MID units)**

| **Study (k = 21)** | **SPC**  **MD_change_** | | **SPC**  **Total** | **UC**  **MD_change_** | **UC**  **Total** | | **SMD (MID)** | **95% CI** | | **weight**  **(random,**  **in %)** | |
| --- | --- | --- | --- | --- | --- | --- | --- | --- | --- | --- | --- |
| Bakitas *et al* 2009^55^ | 1.60 | | 72 | 0.80 | 76 | | 0.27 | -0.67 to 1.21 | | 3.7 | |
| Bakitas *et al* 2015^56^ | 3.79 | | 72 | 2.62 | 83 | | 0.39 | -0.28 to 1.06 | | 4.9 | |
| Bakitas *et al* 2020^57^ | 0.00 | | 119 | -0.30 | 122 | | 0.23 | -0.43 to 0.89 | | 4.9 | |
| Bassi *et al* 2021^38^ | 0.80 | | 14 | -4.20 | 19 | | 1.67 | 1.29 to 2.04 | | 6.4 | |
| Bekelman *et al* 2018^58^ | 2.20 | | 121 | 0.80 | 119 | | 0.28 | -0.15 to 0.71 | | 6.1 | |
| Brims *et al* 2019^51^ | 2.10 | | 45 | 0.10 | 42 | | 0.40 | -0.55 to 1.35 | | 3.6 | |
| do Carmo *et al* 2017^33^ | -3.83 | | 12 | -2.07 | 14 | | -0.35 | -0.95 to 0.24 | | 5.2 | |
| Edmonds *et al* 2010^60^ | -0.10 | | 21 | 0.80 | 17 | | -0.11 | -0.62 to 0.39 | | 5.7 | |
| El-Jahwari *et al* 2016^34^ | 0.98 | | 75 | -0.54 | 74 | | 0.30 | 0.03 to 0.58 | | 6.8 | |
| El-Jahwari *et al* 2021^35^ | 2.60 | | 57 | 0.90 | 48 | | 1.29 | 0.19 to 2.39 | | 3.1 | |
| Evans *et al* 2021^61^ | 1.43 | | 23 | 0.05 | 24 | | 0.35 | -0.27 to 0.96 | | 5.1 | |
| Eychmueller *et al* 2021^40^ | -0.73 | | 59 | 0.10 | 58 | | -0.28 | -0.67 to 0.12 | | 6.2 | |
| Gao *et al* 2020^63^ | 0.35 | | 176 | -0.08 | 174 | | 0.33 | -0.48 to 1.13 | | 4.2 | |
| Hoek *et al* 2017^36^ | -1.22 | | 13 | 1.16 | 16 | | -1.80 | -4.09 to 0.48 | | 1.0 | |
| Kluger *et al* 2020^65^ | 1.19 | | 93 | 0.73 | 87 | | 0.35 | -0.27 to 0.96 | | 5.1 | |
| Rogers *et al* 2014^27^ | 2.00 | | 41 | 1.00 | 40 | | 0.76 | -0.75 to 2.26 | | 2.0 | |
| Sidebottom *et al* 2015^67^ | 2.90 | | 79 | 2.18 | 88 | | 0.14 | -0.14 to 0.43 | | 6.7 | |
| Slama *et al* 2020^68^ | 1.25 | | 37 | 0.75 | 43 | | 0.25 | -1.27 to 1.77 | | 2.0 | |
| Tattersall *et al* 2014^44^ | -2.30 | | 33 | 3.00 | 45 | | -0.50 | -0.76 to -0.25 | | 6.9 | |
| Temel *et al* 2020^69^ | 1.23 | | 75 | 0.21 | 84 | | 0.77 | -0.03 to 1.58 | | 4.2 | |
| Woo *et al* 2019^46^ | -0.05 | | 45 | -0.20 | 45 | | 0.05 | -0.37 to 0.47 | | 6.1 | |
|  | |  | |  | |  | | |  | |  |
| ***Meta-analysis*** | | **SMD (MID)** | | **95% CI** | | ***t*** | | | ***p*** | |  |
| Random effects model | | 0.26 | | -0.00 to 0.52 | | 2.060 | | | 0.053 | |  |
|  | |  | |  | |  | | |  | |  |
| ***Heterogeneity*** | |  | |  | | ***Q (df)*** | | | ***p*** | |  |
| *τ²* | | 0.23 | | 0.09 to 0.58 | | 111.70 (20) | | | **<0.001** | |  |
| *I²* | | 82.1% | | 73.6 to 87.8% | |  | | |  | |  |
| *H* | | 2.36 | | 1.95 to 2.87 | |  | | |  | |  |

**Forest plot**


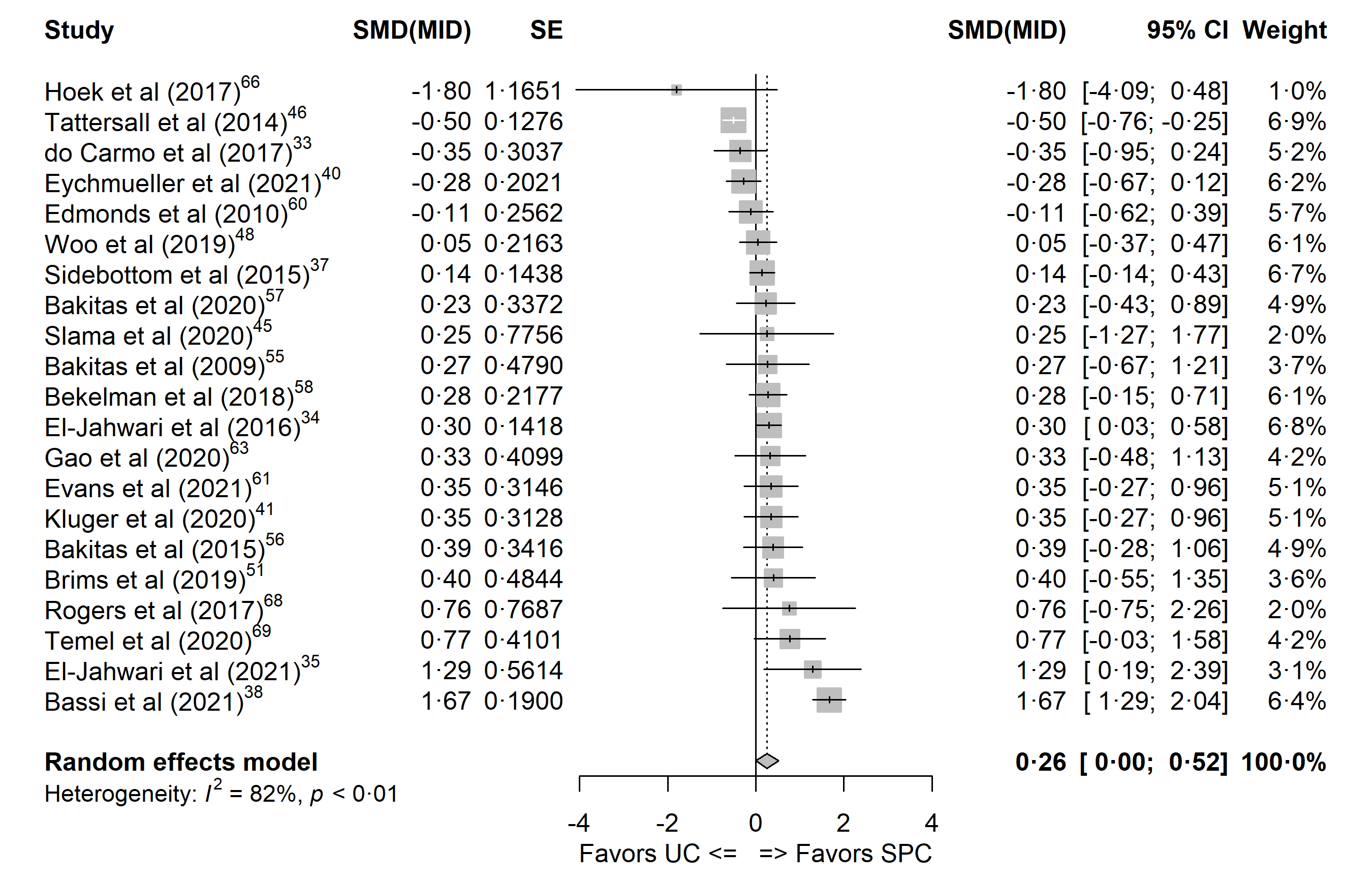


**Publication bias**

Egger’s enhanced funnel plot

| Linear regression test of funnel plot asymmetry  Intercept: 0.853  95% CI: -1.298 to 3.004  *t*(20) = 0.777, *p* = 0.447 | 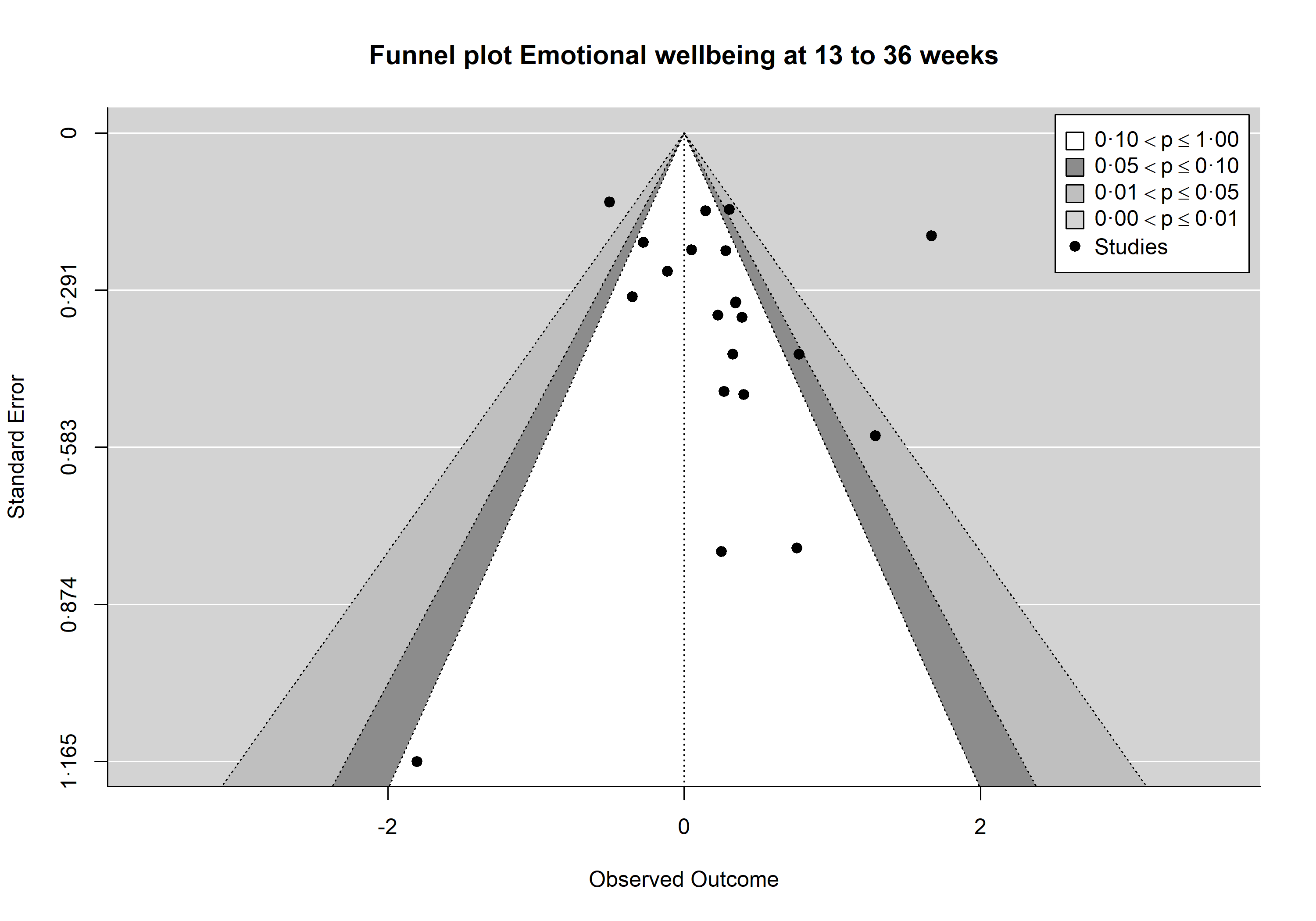 |
| --- | --- |

**Analysis with effect size RR (achieving change ≥1 MID)**

Number of studies combined: k = 21

Number of observations: 2600; Number of events: 899

| **Study (k = 21)** | **SPC**  **#1MID** | | **SPC**  **Total** | **UC**  **#1MID** | | **UC**  **Total** | **RR**  **(> 1MID)** | | **95% CI** | | **weight (random,**  **in %)** | |
| --- | --- | --- | --- | --- | --- | --- | --- | --- | --- | --- | --- | --- |
| Bakitas *et al* 2009^55^ | 31 | | 72 | 31 | | 76 | 1.06 | | 0.72 to 1.54 | | 7.2 | |
| Bakitas *et al* 2015^56^ | 39 | | 72 | 39 | | 83 | 1.15 | | 0.84 to 1.57 | | 10.6 | |
| Bakitas *et al* 2020^57^ | 41 | | 119 | 39 | | 122 | 1.08 | | 0.75 to 1.54 | | 8.0 | |
| Bassi *et al* 2021^38^ | 1.5 | | 14 | 0.5 | | 19 | 4.07 | | 0.18 to 92.69 | | 0.1 | |
| Bekelman *et al* 2018^58^ | 45 | | 121 | 37 | | 119 | 1.20 | | 0.84 to 1.70 | | 8.2 | |
| Brims *et al* 2019^51^ | 18 | | 45 | 14 | | 42 | 1.20 | | 0.69 to 2.10 | | 3.3 | |
| do Carmo *et al* 2017^33^ | 0.5 | | 12 | 0.5 | | 14 | 1.17 | | 0.02 to 54.46 | | 0.1 | |
| Edmonds *et al* 2010^60^ | 2 | | 21 | 2 | | 17 | 0.81 | | 0.13 to 5.16 | | 0.3 | |
| El-Jahwari *et al* 2016^34^ | 13 | | 75 | 8 | | 74 | 1.60 | | 0.71 to 3.64 | | 1.5 | |
| El-Jahwari *et al* 2021^35^ | 36 | | 57 | 22 | | 48 | 1.38 | | 0.96 to 1.99 | | 7.7 | |
| Evans *et al* 2021^61^ | 5 | | 23 | 5 | | 24 | 1.04 | | 0.35 to 3.13 | | 0.9 | |
| Eychmueller *et al* 2021^40^ | 9 | | 59 | 10 | | 58 | 0.88 | | 0.39 to 2.02 | | 1.5 | |
| Gao *et al* 2020^63^ | 75 | | 176 | 67 | | 174 | 1.11 | | 0.86 to 1.43 | | 15.9 | |
| Hoek *et al* 2017^36^ | 4 | | 13 | 8 | | 16 | 0.62 | | 0.24 to 1.59 | | 1.1 | |
| Kluger *et al* 2020^65^ | 45 | | 93 | 37 | | 87 | 1.14 | | 0.82 to 1.57 | | 9.9 | |
| Rogers *et al* 2014^27^ | 23 | | 41 | 19 | | 40 | 1.18 | | 0.77 to 1.80 | | 5.7 | |
| Sidebottom *et al* 2015^67^ | 26 | | 79 | 23 | | 88 | 1.26 | | 0.79 to 2.02 | | 4.6 | |
| Slama *et al* 2020^68^ | 17 | | 37 | 18 | | 43 | 1.10 | | 0.67 to 1.80 | | 4.2 | |
| Tattersall *et al* 2014^44^ | 0.5 | | 33 | 5 | | 45 | 0.14 | | 0.01 to 2.41 | | 0.1 | |
| Temel *et al* 2020^69^ | 37 | | 75 | 31 | | 84 | 1.34 | | 0.93 to 1.92 | | 7.9 | |
| Woo *et al* 2019^46^ | 8 | | 45 | 6 | | 45 | 1.33 | | 0.50 to 3.53 | | 1.1 | |
|  | |  | | |  | | |  | |  | |  |
| ***Meta-analysis*** | | **RR** | | | **95% CI** | | | ***t*** | | ***p*** | |  |
| Random effects model | | 1.16 | | | 1.08 to 1.24 | | | 4.540 | | **<0.001** | |  |
|  | |  | | |  | | |  | |  | |  |
| ***Heterogeneity*** | |  | | |  | | | ***Q (df)*** | | ***p*** | |  |
| *τ²* | | 0.00 | | | 0.00 to 0.00 | | | 10.58 (21) | | 0.970 | |  |
| *I²* | | 0.0% | | | 0.0 to 46.2% | | |  | |  | |  |
| *H* | | 1.00 | | | 1.00 to 1.36 | | |  | |  | |  |

**Forest plot of RR effect size for the QoL outcome 13 to 36 weeks**


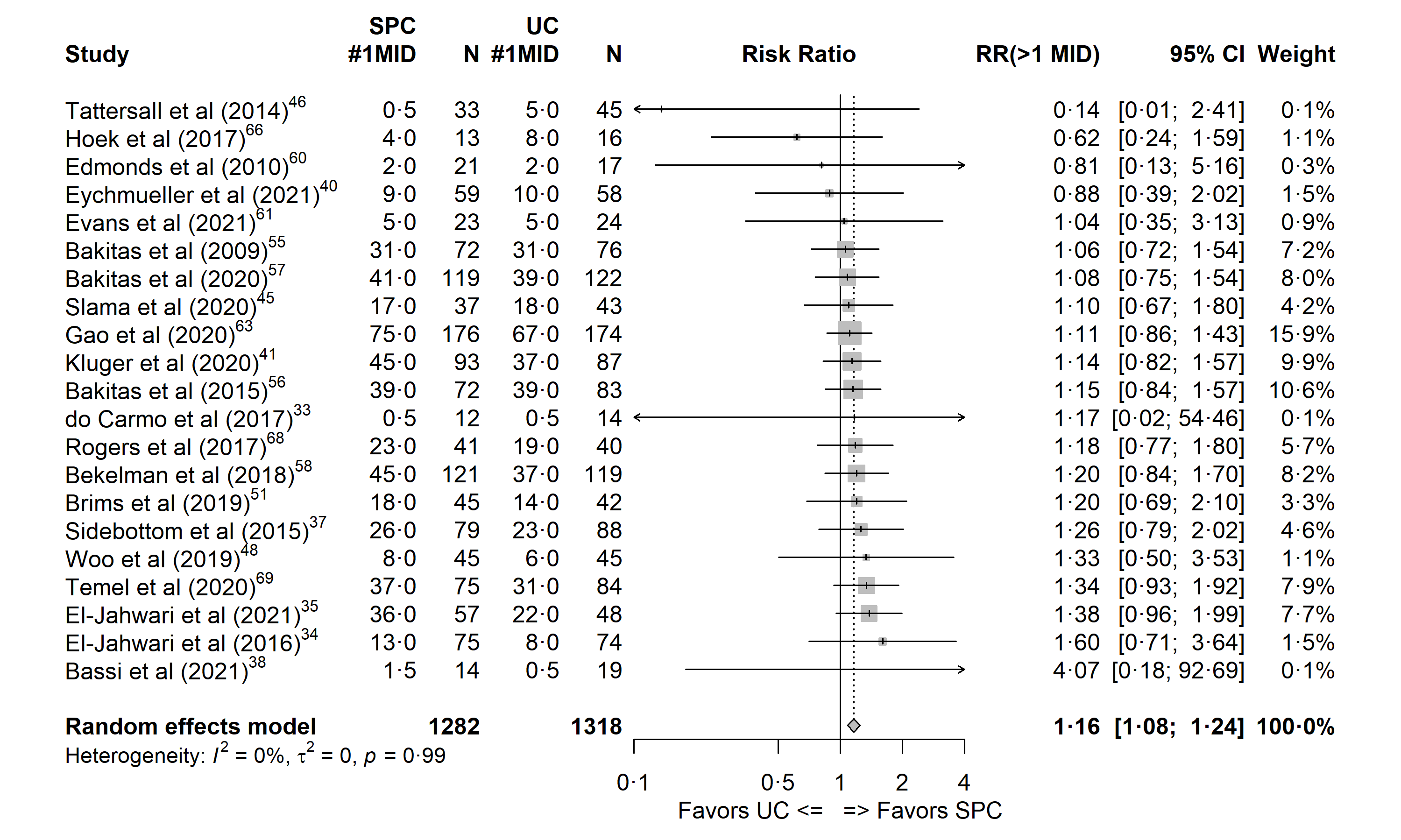


The risk ratio of RR = 1.16 (95% CI = 1.08 to 1.24) translates into a point estimate of a 16% increased probability of experiencing a change in emotional wellbeing of at least 1 MID size with SPC. This is statistically significant.

The number needed to treat is calculated as follows (*p_CG_* is the baseline risk of experiencing change of at least 1 MID in the control group):

$$\frac{1}{p_{CG}\cdot(1-RR)}=\frac{1}{\frac{422}{1318}\cdot0.16}=19.520 \to19$$

The NNT is 19, meaning that 19 people need to be treated with SPC in order for one person to have a change in emotional wellbeing at 13 to 36 weeks of at least 1 MID.

**Meta-regression: Univariate meta-regression analyses with covariates**

| *k = 21* | **Regression** | | | | | **Heterogeneity** | | | **Test of moderators** | |
| --- | --- | --- | --- | --- | --- | --- | --- | --- | --- | --- |
|  | *b* | *SE* | *t* | *p* | 95% CI | *I²* | *Q* | *p* | *F* | *p* |
| ***Attrition (in %)*** |  |  |  |  |  |  |  |  |  |  |
| Intercept | 0.52 | 0.23 | 2.275 | **0.035** | - | 75 | 88.6 | **0.000** | 1.873 | 0.187 |
| Attrition (in %) | -0.01 | 0.01 | -1.369 | 0.187 | -0.03; -0.01 |  |  |  |  |  |
| ***% advanced disease*** |  |  |  |  |  |  |  |  |  |  |
| Intercept | 0.43 | 0.51 | 0.842 | 0.419 | - | 84 | 98.9 | **0.000** | 0.006 | 0.938 |
| % advanced disease | -0.00 | 0.01 | -0.080 | 0.938 | -0.02; 0.02 |  |  |  |  |  |
| ***Disease group (ref: Cancer)*** |  |  |  |  |  |  |  |  |  |  |
| Intercept | 0.09 | 0.17 | 0.592 | 0.561 | - | 75 | 88.3 | **0.000** | 1.926 | 0.181 |
| Non-cancer | 0.34 | 0.24 | 1.388 | 0.181 | -0.17; 0.85 |  |  |  |  |  |
| ***RoB2 score (ref: low risk)*** |  |  |  |  |  |  |  |  |  |  |
| Intercept | 0.24 | 0.27 | 0.893 | 0.383 | - | 77 | 109.6 | **0.000** | 1.074 | 0.363 |
| RoB2: Some risk | 0.15 | 0.32 | 0.469 | 0.645 | -0.52; 0.82 |  |  |  |  |  |
| RoB2: High risk | -0.30 | 0.38 | -0.806 | 0.431 | -1.09; 0.49 |  |  |  |  |  |
| ***Service composition score*** |  |  |  |  |  |  |  |  |  |  |
| Intercept | -0.91 | 0.42 | -2.155 | **0.044** | - | 67 | 57.3 | **0.000** | 8.057 | **0.011** |
| Service composition score | 0.11 | 0.04 | 2.839 | **0.011** | 0.03; 0.18 |  |  |  |  |  |
| ***Setting (ref: multiple settings)*** |  |  |  |  |  |  |  |  |  |  |
| Intercept | 0.26 | 0.20 | 1.303 | 0.209 | - | 78 | 110.3 | **0.000** | 0.002 | 0.998 |
| Inpatient consulting model | -0.03 | 0.35 | -0.063 | 0.951 | -0.75; 0.71 |  |  |  |  |  |
| Home or hospital outreach | -0.00 | 0.30 | -0.009 | 0.993 | -0.63; 0.63 |  |  |  |  |  |
| ***Type of intervention (ref: SPC)*** |  |  |  |  |  |  |  |  |  |  |
| Intercept | 0.05 | 0.23 | 0.235 | 0.817 | - | 67 | 52.3 | **0.000** | 3.231 | **0.048** |
| Early SPC | -0.12 | 0.30 | -0.419 | 0.680 | -0.75; 0.50 |  |  |  |  |  |
| Integrated collaborative care | 0.66 | 0.30 | 2.196 | **0.042** | 0.02; 1.29 |  |  |  |  |  |
| Nurse-led palliative care | 0.24 | 0.38 | 0.638 | 0.532 | -0.56; 1.05 |  |  |  |  |  |
| ***Year*** |  |  |  |  |  |  |  |  |  |  |
| Intercept | -0.87 | 0.61 | -1.418 | 0.172 | - | 74 | 83.3 | **0.000** | 3.488 | *0.077* |
| Year | 0.06 | 0.03 | 1.867 | *0.077* | -0.01; 0.13 |  |  |  |  |  |

.

**Bubble plots of univariate meta-regression analyses**

| **Attrition** | *F*(1,19) = 1.873  *p* = 0.187 | 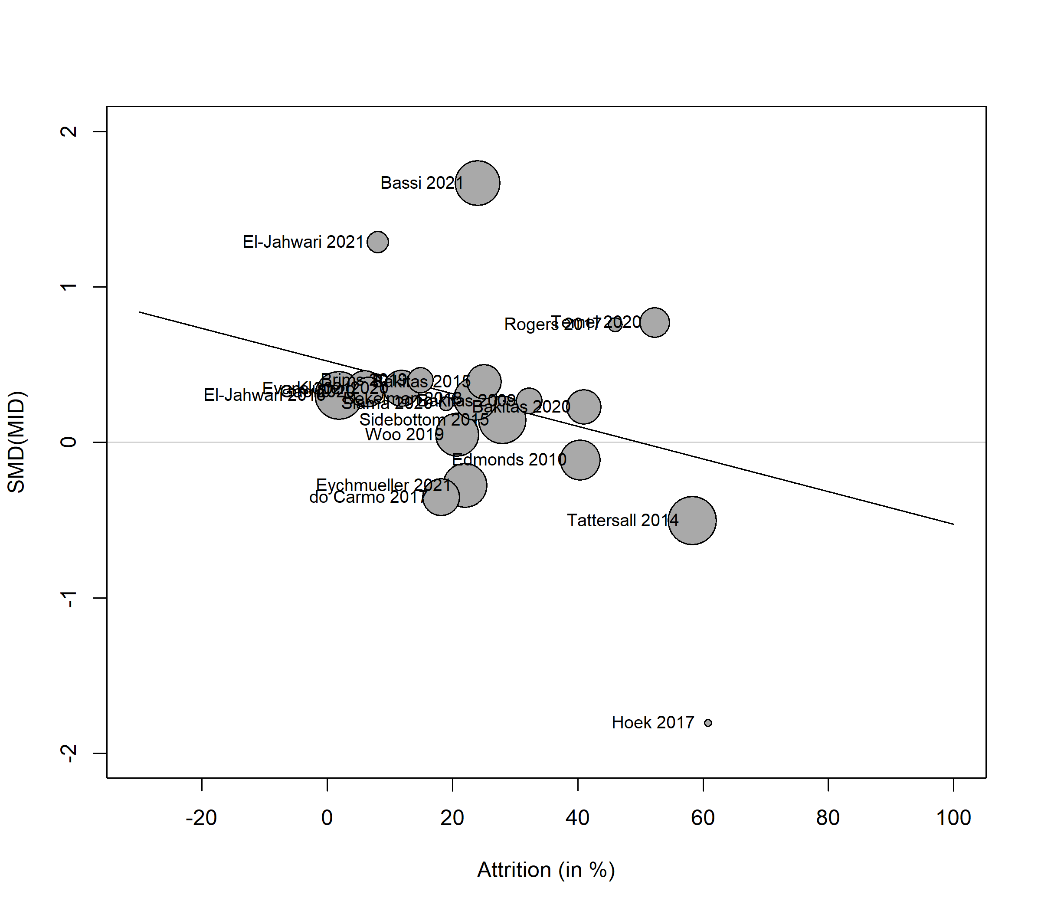 |
| --- | --- | --- |
| **% advanced disease** | *F*(1,10) = 0.006  *p* = 0.938 | 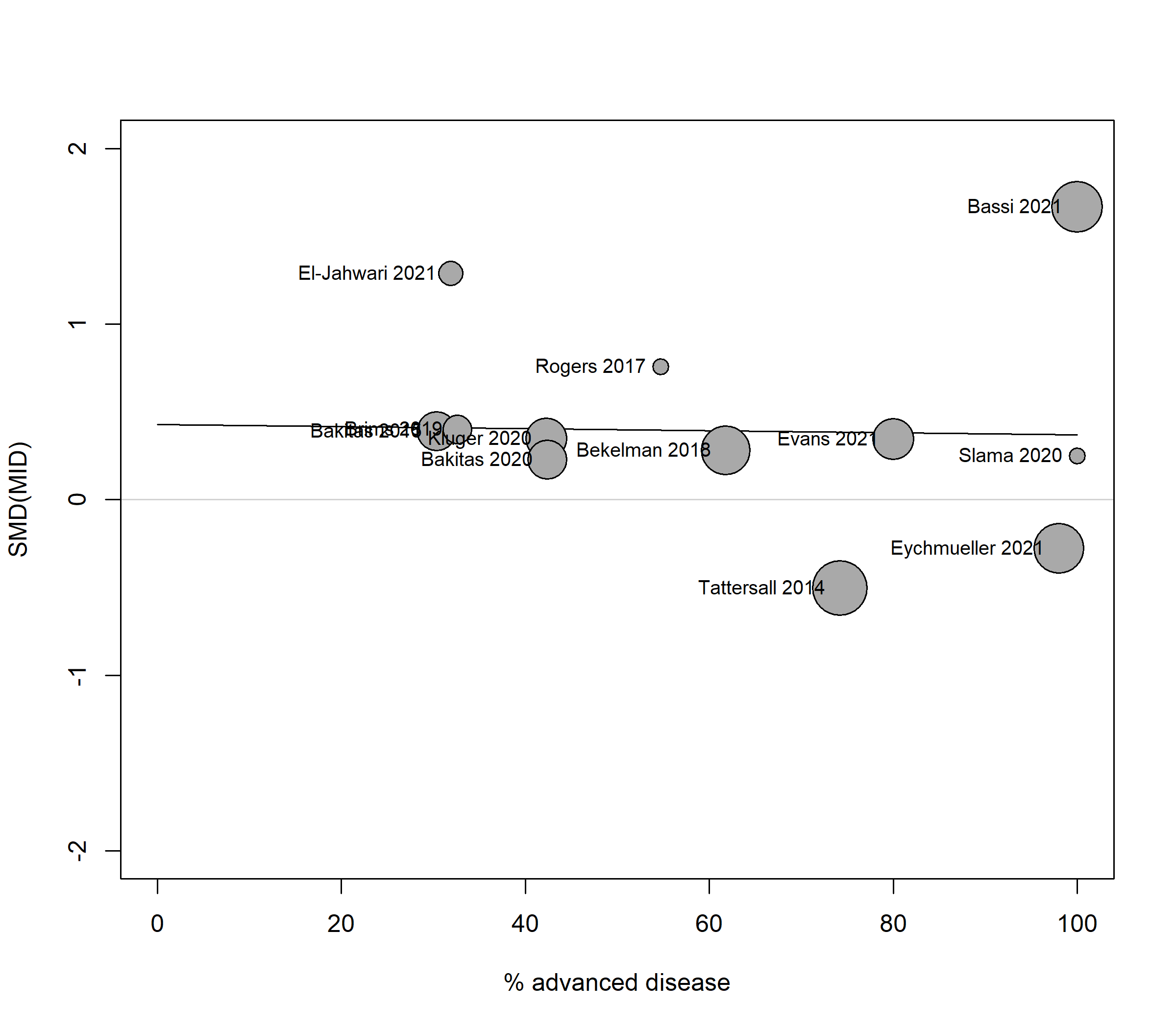 |
| **Disease group** | *F*(1,19) = 1.926  *p* = 0.181 | 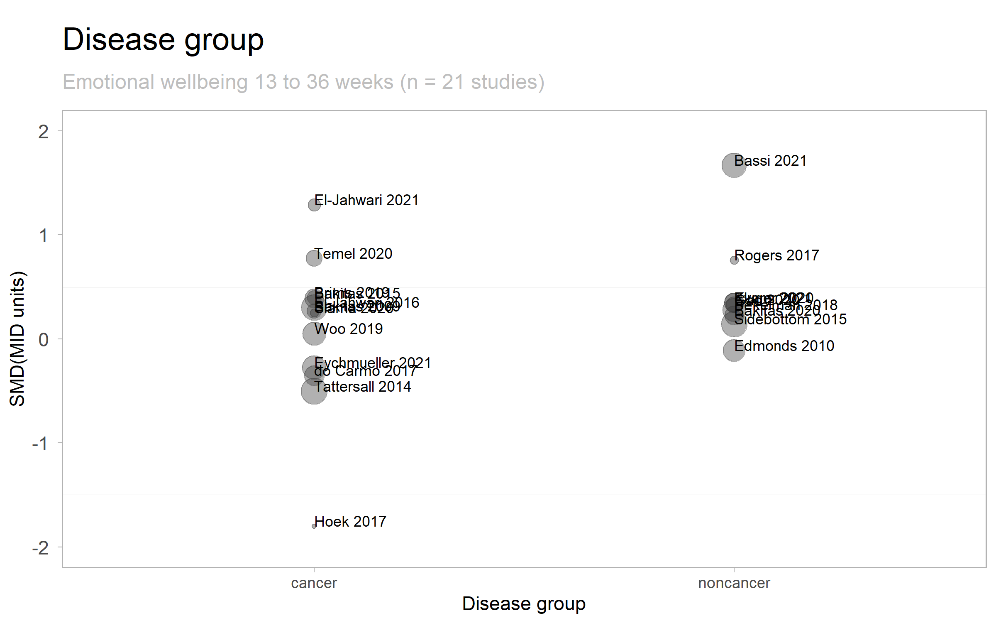 |
| **RoB2 score** | *F*(2,18) = 1.074  *p* = 0.363 | 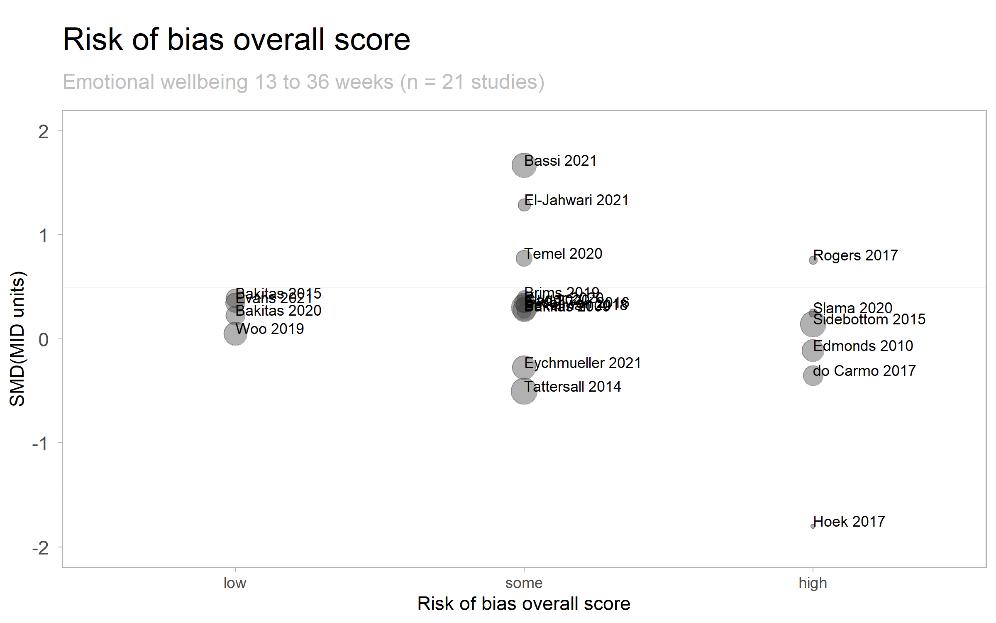 |
| **Service composition score** | *F*(1,19) = 8.057  *p* = **0.011** | 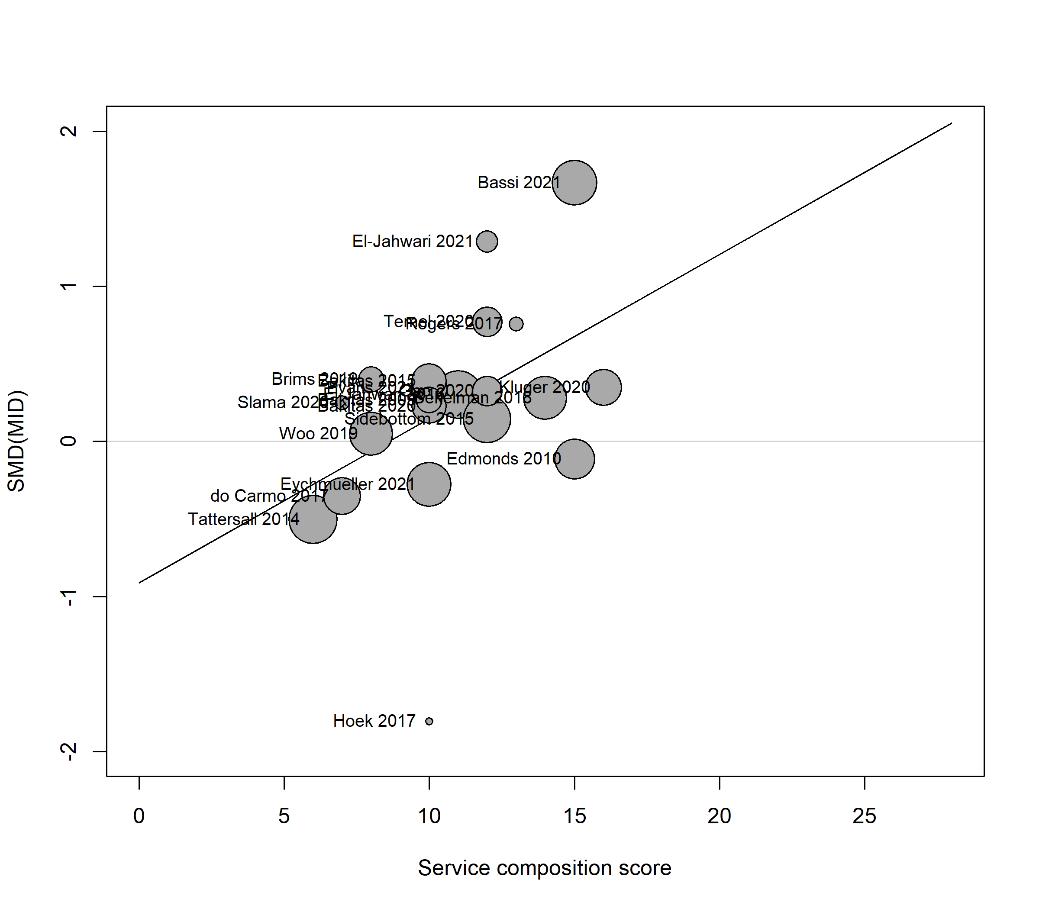 |
| **Setting** | *F*(2,18) = 0.002  *p* = 0.998 | 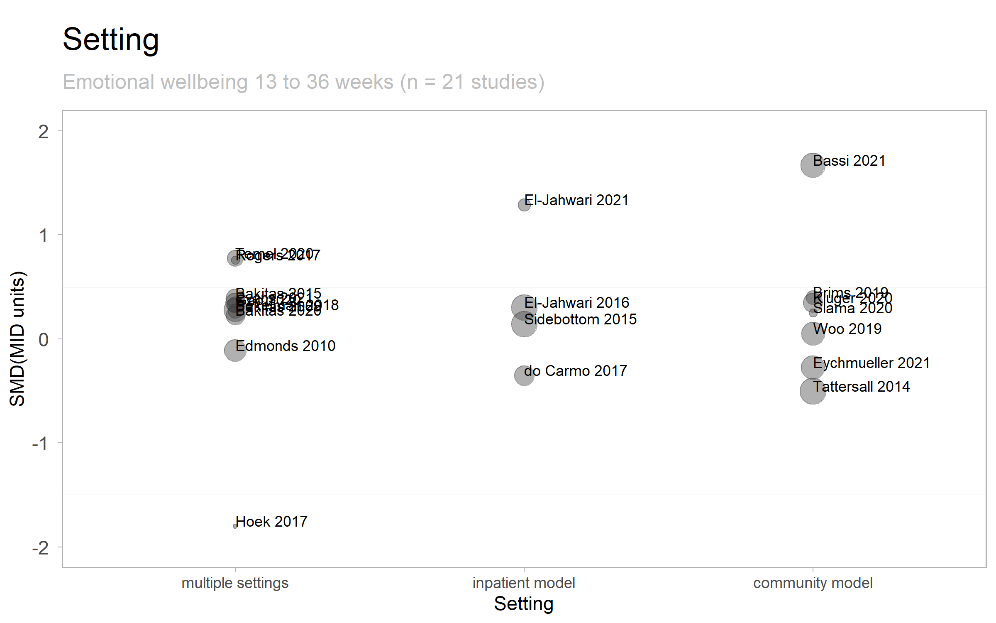 |
| **Type of intervention** | *F*(3,17) = 3.231  *p* = **0.048** | 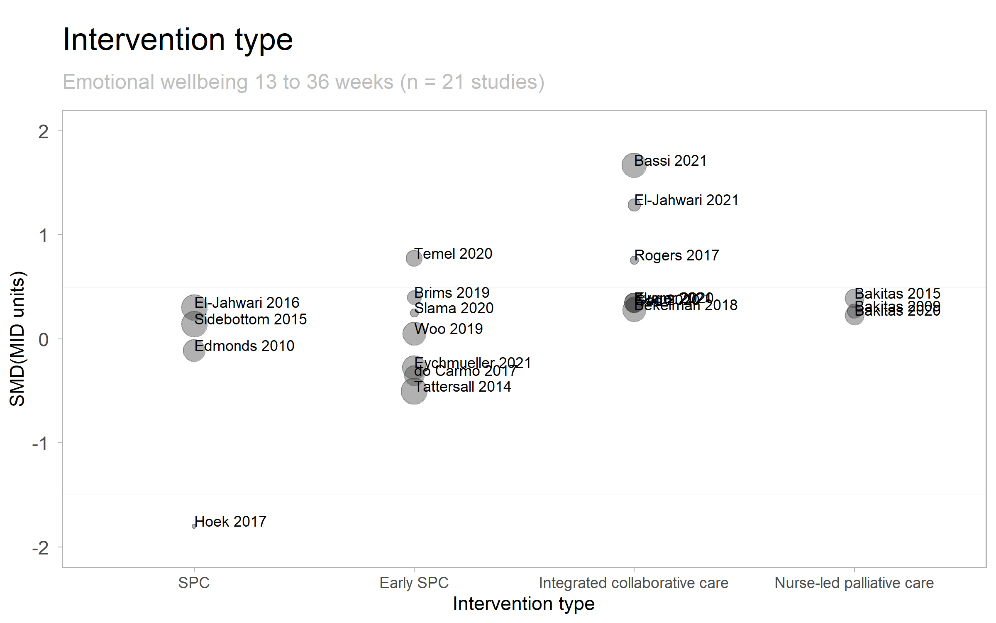 |
| **Year** | *F*(1,19) = 3.488  *p* = *0.077* | 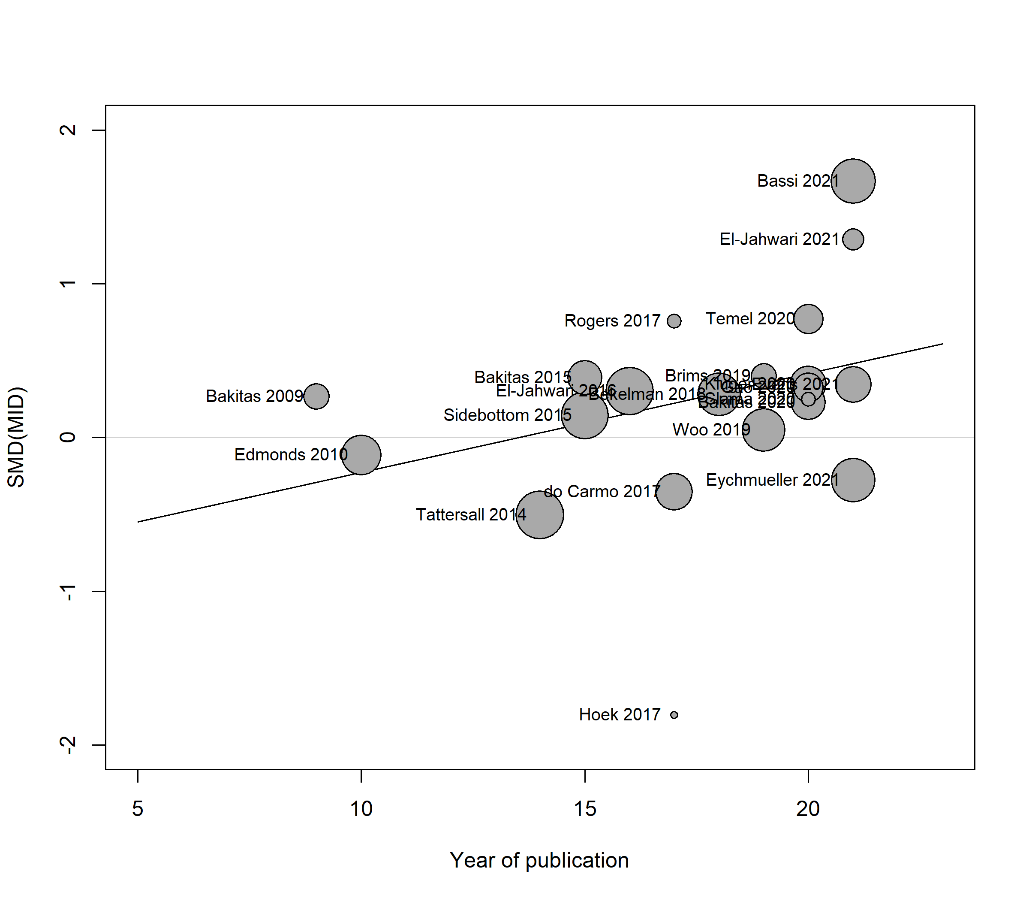 |

## Fig H and Table H: Emotional wellbeing at 37 to 52 weeks

**Analysis with effect size SMD (MID units)**

| **Study (k = 7)** | **SPC**  **MD_change_** | | **SPC**  **Total** | **UC**  **MD_change_** | | **UC**  **Total** | **SMD (MID)** | | **95% CI** | | **weight**  **(random,**  **in %)** | |
| --- | --- | --- | --- | --- | --- | --- | --- | --- | --- | --- | --- | --- |
| Bakitas *et al* 2009^55^ | 0.59 | | 43 | -1.17 | | 30 | -0.16 | | -1.41 to 1.10 | | 4.3 | |
| Bakitas *et al* 2015^56^ | 3.79 | | 72 | 2.62 | | 83 | 0.15 | | -0.49 to 0.79 | | 12.3 | |
| Bassi *et al* 202^38^ | 2.10 | | 105 | 0.50 | | 105 | 0.75 | | 0.23 to 1.26 | | 16.0 | |
| Greer *et al* 2022^36^ | 0.60 | | 63 | 0.60 | | 57 | -0.17 | | -1.59 to 1.25 | | 3.5 | |
| Kluger *et al* 2020^41^ | -1.95 | | 19 | -0.94 | | 18 | -0.09 | | -0.75 to 0.57 | | 11.8 | |
| Tattersall *et al* 2014^46^ | -0.10 | | 21 | 0.80 | | 17 | -0.19 | | -0.51 to 0.13 | | 23.8 | |
| Woo *et al* 2019^48^ | 0.98 | | 75 | -0.54 | | 74 | 0.11 | | -0.12 to 0.33 | | 28.4 | |
|  | |  | | |  | | |  | |  | |  |
| ***Meta-analysis*** | | **SMD (MID)** | | | **95% CI** | | | ***t*** | | ***p*** | |  |
| Random effects model | | 0.10 | | | -0.21 to 0.41 | | | 0.790 | | 0.461 | |  |
|  | |  | | |  | | |  | |  | |  |
| ***Heterogeneity*** | |  | | |  | | | ***Q (df)*** | | ***p*** | |  |
| *τ²* | | 0.06 | | | 0.00 to 0.43 | | | 9.75 (6) | | 0.136 | |  |
| *I²* | | 38.4% | | | 0.0 to 74.1% | | |  | |  | |  |
| *H* | | 1.27 | | | 1.00 to 1.96 | | |  | |  | |  |

**Forest plot**


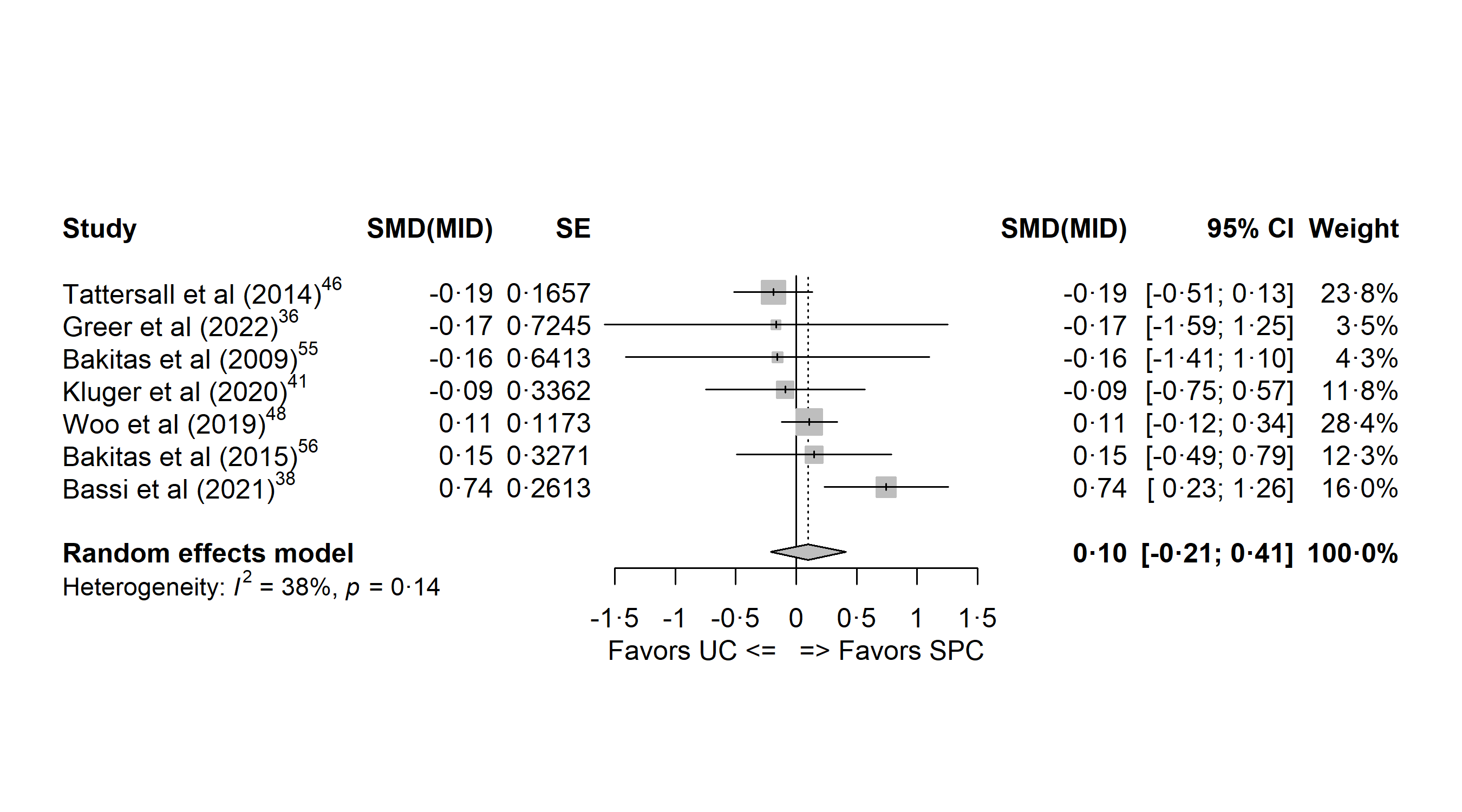


**Publication bias**

Egger’s enhanced funnel plot

| Linear regression test of funnel plot asymmetry  Intercept: 0.071  95% CI: -1.922 to 2.064  *t*(6) = 0.070, *p* = 0.947 | 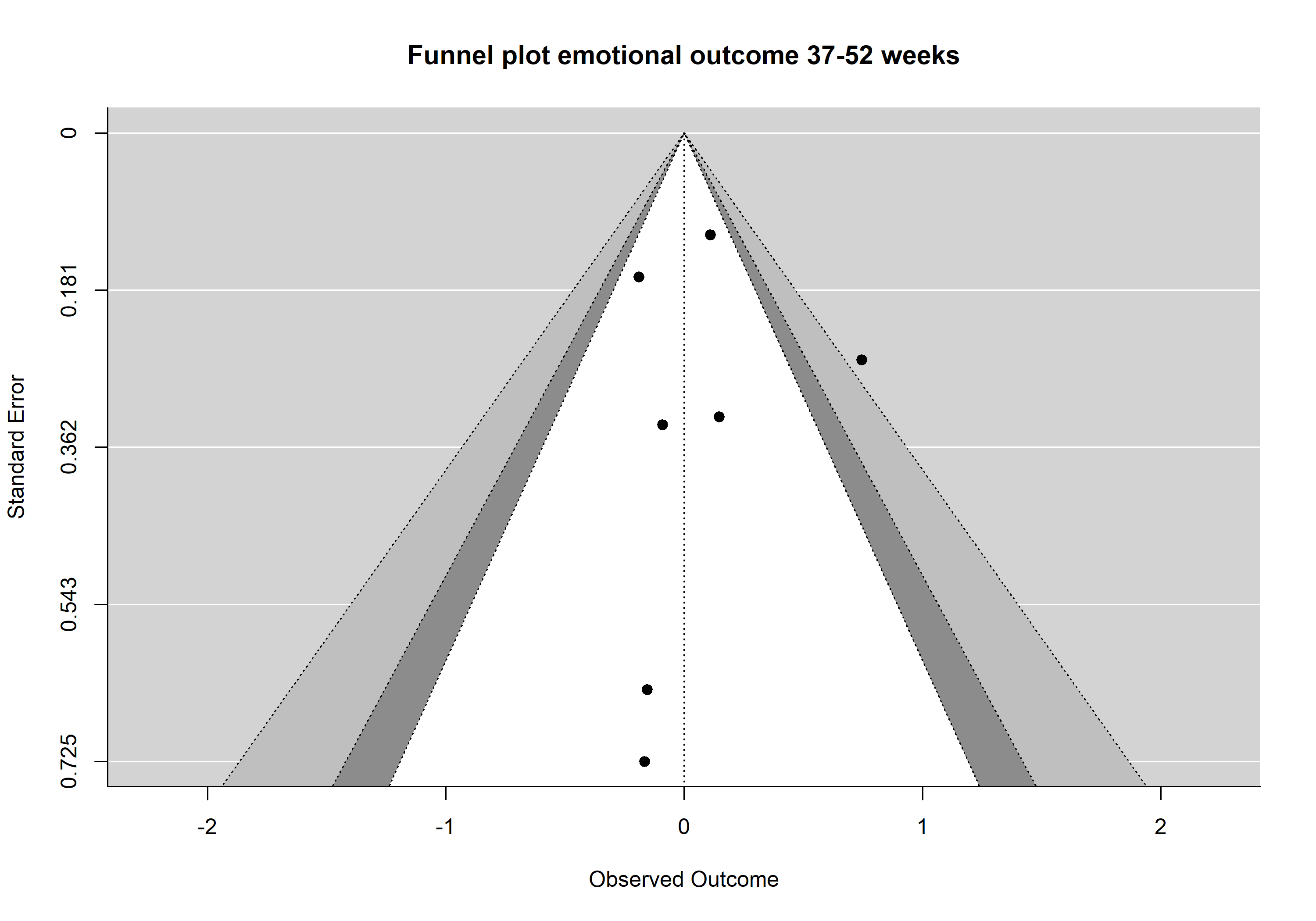 |
| --- | --- |

**Analysis with effect size RR (achieving change ≥1 MID)**

Number of studies combined: k = 7

Number of observations: 481; Number of events: 176

| **Study (k = 7)** | **SPC**  **#1MID** | | **SPC**  **Total** | **UC**  **#1MID** | | **UC**  **Total** | **RR**  **(> 1MID)** | | **95% CI** | | **weight (random,**  **in %)** | |
| --- | --- | --- | --- | --- | --- | --- | --- | --- | --- | --- | --- | --- |
| Bakitas *et al* 2009^55^ | 8 | | 26 | 11 | | 31 | 0.87 | | 0.41 to 1.83 | | 8.5 | |
| Bakitas *et al* 2015^56^ | 16 | | 29 | 14 | | 28 | 1.10 | | 0.67 to 1.81 | | 19.3 | |
| Bassi *et al* 202^38^ | 2 | | 14 | 1 | | 19 | 2.71 | | 0.27 to 27.05 | | 0.9 | |
| Greer *et al* 2022^36^ | 15 | | 40 | 16 | | 39 | 0.91 | | 0.53 to 1.58 | | 15.6 | |
| Kluger *et al* 2020^41^ | 43 | | 87 | 44 | | 85 | 0.95 | | 0.71 to 1.28 | | 54.1 | |
| Tattersall *et al* 2014^46^ | 1 | | 21 | 3 | | 29 | 0.46 | | 0.05 to 4.12 | | 1.0 | |
| Woo *et al* 2019^48^ | 1 | | 19 | 1 | | 14 | 0.74 | | 0.05 to 10.80 | | 0.7 | |
|  | |  | | |  | | |  | |  | |  |
| ***Meta-analysis*** | | **RR** | | | **95% CI** | | | ***t*** | | ***p*** | |  |
| Random effects model | | 0.97 | | | 0.84 to 1.12 | | | -0.560 | | 0.593 | |  |
|  | |  | | |  | | |  | |  | |  |
| ***Heterogeneity*** | |  | | |  | | | ***Q (df)*** | | ***p*** | |  |
| *τ²* | | 0.00 | | | 0.00 to 0.18 | | | 1.66 (6) | | 0.948 | |  |
| *I²* | | 0.0% | | | 0.0 to 70.8% | | |  | |  | |  |
| *H* | | 1.00 | | | 1.00 to 1.85 | | |  | |  | |  |

**Forest plot of RR effect size for the QoL outcome 37 to 52 weeks**


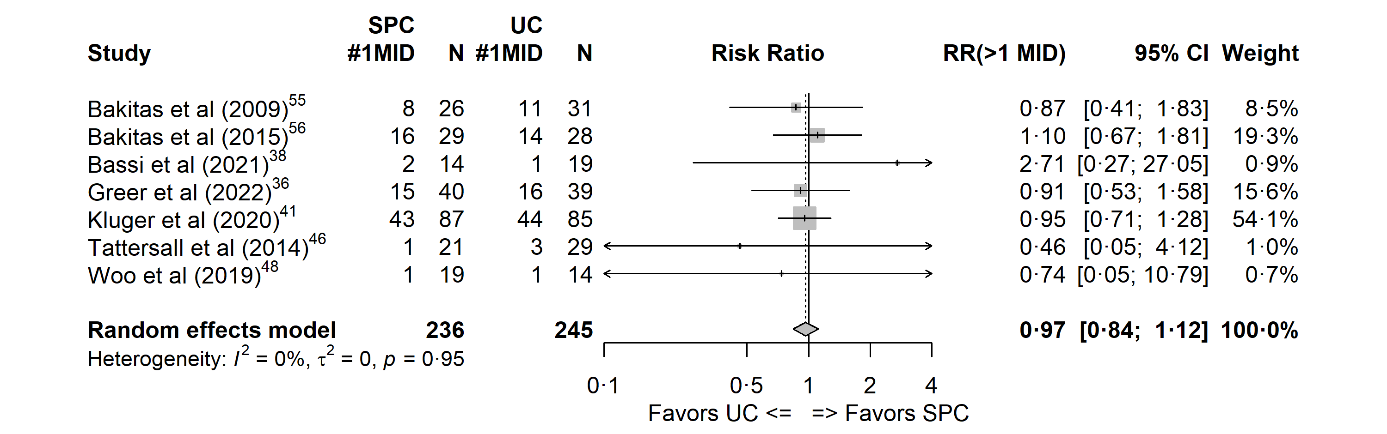


The risk ratio of RR = 0.97 (95% CI = 0.84 to 1.12) translates into a point estimate of no increased probability of experiencing a change in emotional wellbeing of at least 1 MID size with SPC. This is statistically not significant.

**Meta-regression: Univariate meta-regression analyses with covariates**

Since this meta-regression has a low number of included studies, results are not interpreted according to statistical significance and are presented for descriptive purposes only.

| *k = 7* | **Regression** | | | | | **Heterogeneity** | | | **Test of moderators** | |
| --- | --- | --- | --- | --- | --- | --- | --- | --- | --- | --- |
|  | *b* | *SE* | *t* | *p* | 95% CI | *I²* | *Q* | *p* | *F* | *p* |
| ***Attrition (in %)*** |  |  |  |  |  |  |  |  |  |  |
| Intercept | 0.37 | 0.26 | 1.391 | 0.223 | - | 38 | 6.9 | 0.231 | 1.315 | 0.303 |
| Attrition (in %) | -0.01 | 0.01 | -1.147 | 0.303 | -0.03; -0.01 |  |  |  |  |  |
| ***% advanced disease*** |  |  |  |  |  |  |  |  |  |  |
| Intercept | -0.37 | 0.44 | -0.845 | 0.460 | - | 58 | 7.4 | **0.060** | 1.490 | 0.310 |
| % advanced disease | 0.01 | 0.01 | 1.221 | 0.310 | -0.01; 0.03 |  |  |  |  |  |
| ***Disease group (ref: Cancer)*** |  |  |  |  |  |  |  |  |  |  |
| Intercept | -0.01 | 0.13 | -0.061 | 0.954 | - | 32 | 6.3 | 0.276 | 2.700 | 0.161 |
| Non-cancer | 0.41 | 0.25 | 1.643 | 0.161 | -0.23; 1.06 |  |  |  |  |  |
| ***RoB2 score (ref: low risk)*** |  |  |  |  |  |  |  |  |  |  |
| Intercept | 0.12 | 0.23 | 0.539 | 0.613 | - | 50 | 9.5 | 0.089 | 0.014 | 0.911 |
| RoB2: Some risk | -0.03 | 0.29 | -0.118 | 0.911 | -0.78; 0.71 |  |  |  |  |  |
| RoB2: High risk | - | - | - | - | - |  |  |  |  |  |
| ***Service composition score*** |  |  |  |  |  |  |  |  |  |  |
| Intercept | -0.40 | 0.30 | -1.348 | 0.236 | - | 19 | 5.7 | 0.333 | 3.081 | 0.140 |
| Service composition score | 0.05 | 0.03 | 1.755 | 0.140 | -0.02; 0.13 |  |  |  |  |  |
| ***Setting (ref: multiple settings)*** |  |  |  |  |  |  |  |  |  |  |
| Intercept | 0.13 | 0.18 | 0.713 | 0.515 | - | 65 | 9.6 | **0.047** | 0.079 | 0.926 |
| Inpatient consulting model | -0.29 | 0.77 | -0.378 | 0.724 | -2.44; 1.85 |  |  |  |  |  |
| Home or hospital outreach | -0.06 | 0.40 | -0.158 | 0.882 | -1.17; 1.05 |  |  |  |  |  |
| ***Type of intervention (ref: SPC)*** |  |  |  |  |  |  |  |  |  |  |
| Intercept | -0.14 | 0.79 | -0.176 | 0.648 | - | 48 | 31.8 | 0.011 | 1.023 | 0.409 |
| Early SPC | -0.03 | 0.20 | -0.140 | 0.898 | -0.67; 0.62 |  |  |  |  |  |
| Integrated collaborative care | 0.42 | 0.34 | 1.227 | 0.412 | -0.57; 0.25 |  |  |  |  |  |
| Nurse-led palliative care | 0.09 | 0.40 | 0.235 | 0.278 | -0.29; 0.94 |  |  |  |  |  |
| ***Year*** |  |  |  |  |  |  |  |  |  |  |
| Intercept | -124.02 | 65.6 | -1.890 | 0.117 | - | 9 | 5.5 | 0.362 | 3.578 | 0.117 |
| Year | 0.06 | 0.03 | 1.892 | 0.117 | -0.02; 0.15 |  |  |  |  |  |

.

**Bubble plots of univariate meta-regression analyses**

| **Attrition** | *F*(1,5) = 1.315  *p* = 0.303 | 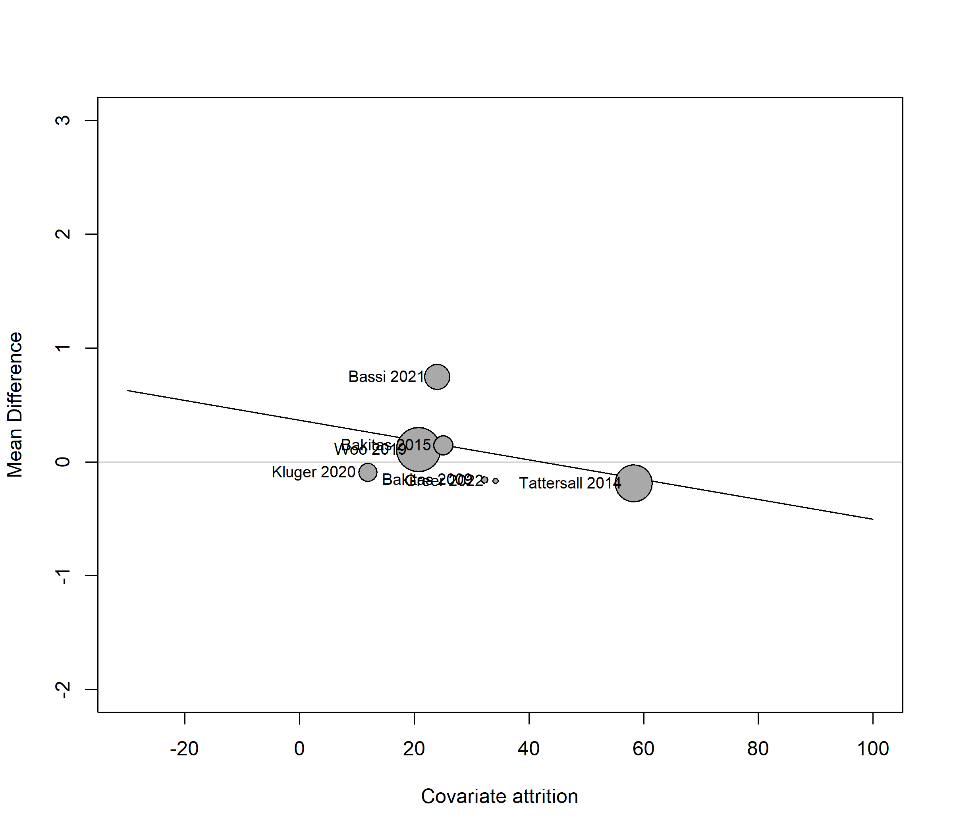 |
| --- | --- | --- |
| **% advanced disease** | *F*(1,3) = 1.490  *p* = 0.310 | 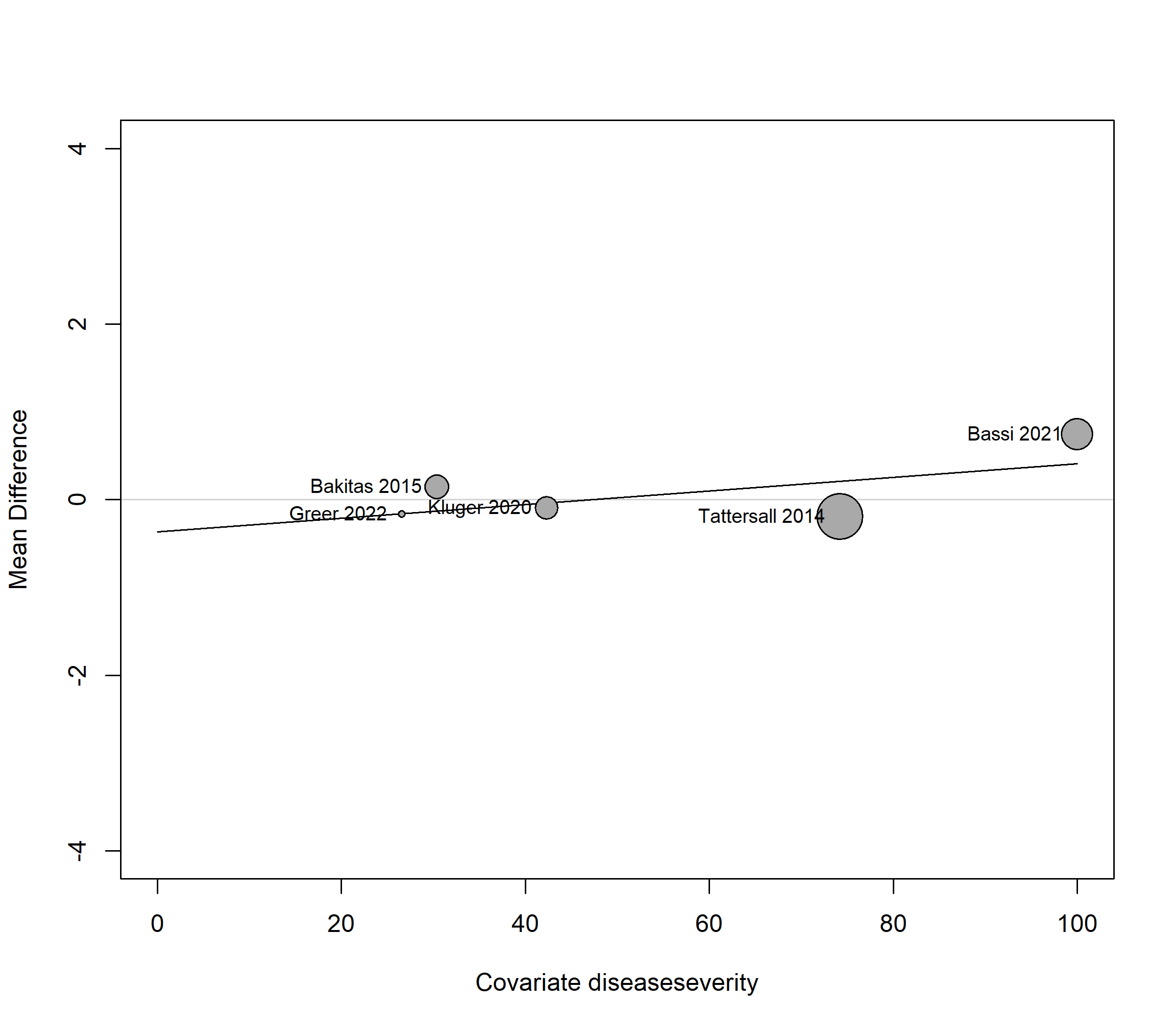 |
| **Disease group** | *F*(1,5) = 2.700  *p* = 0.161 | 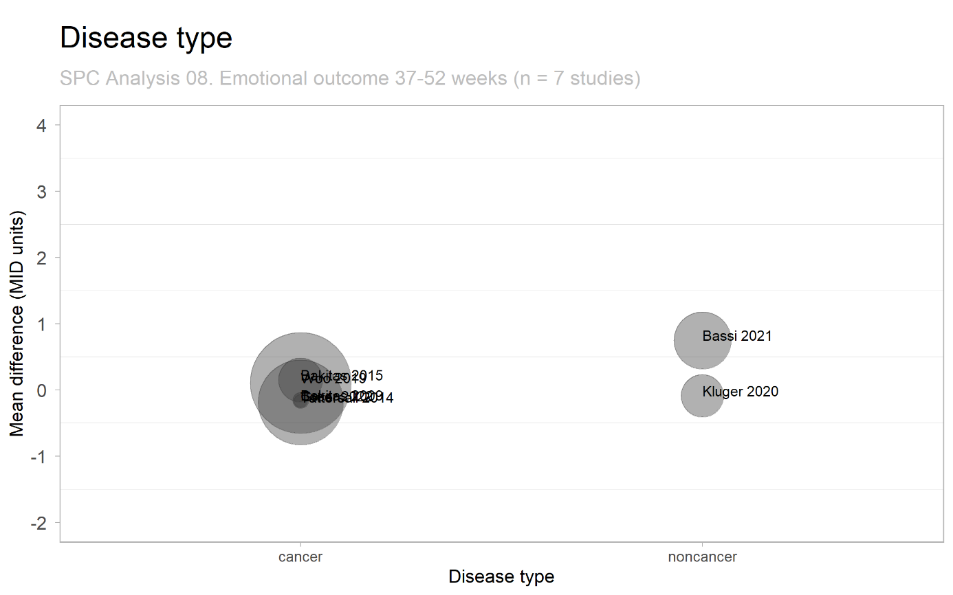 |
| **RoB2 score** | *F*(1,5) = 0.014  *p* = 0.911 | 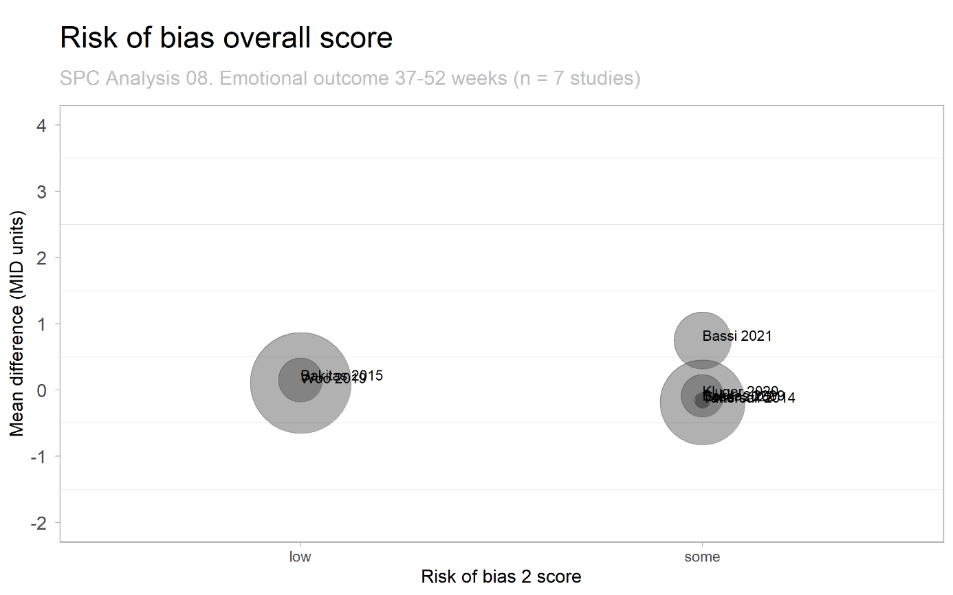 |
| **Service composition score** | *F*(1,5) = 3.081  *p* = 0.140 | 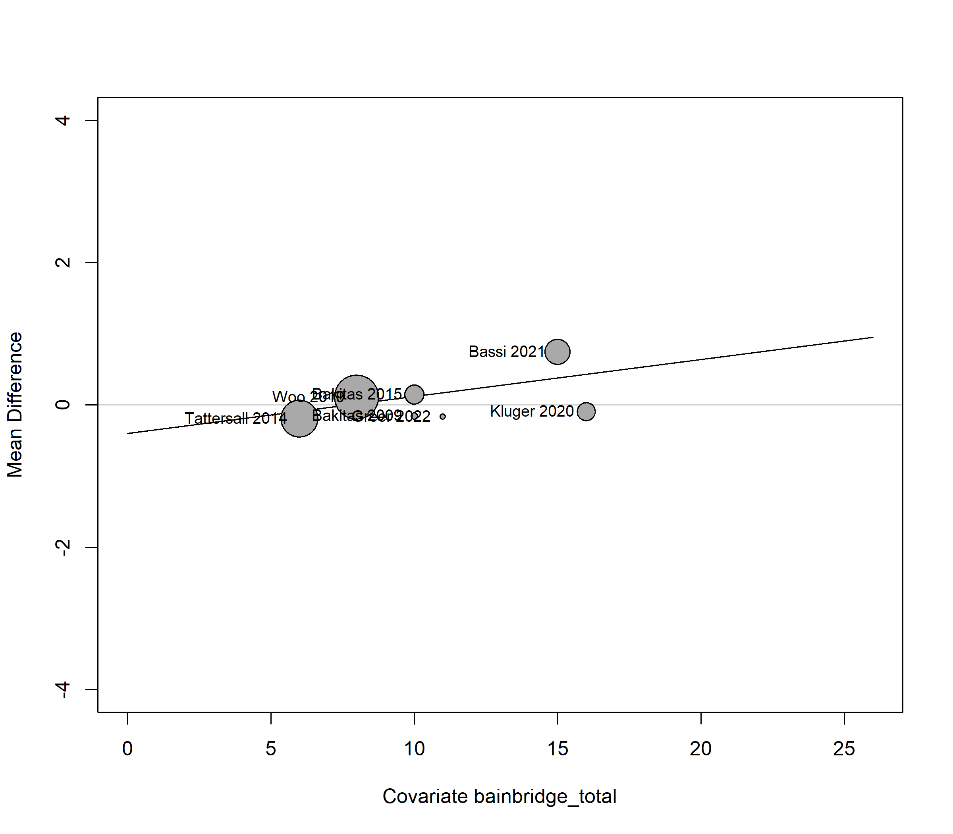 |
| **Setting** | *F*(2,4) = 0.079  *p* = 0.926 | 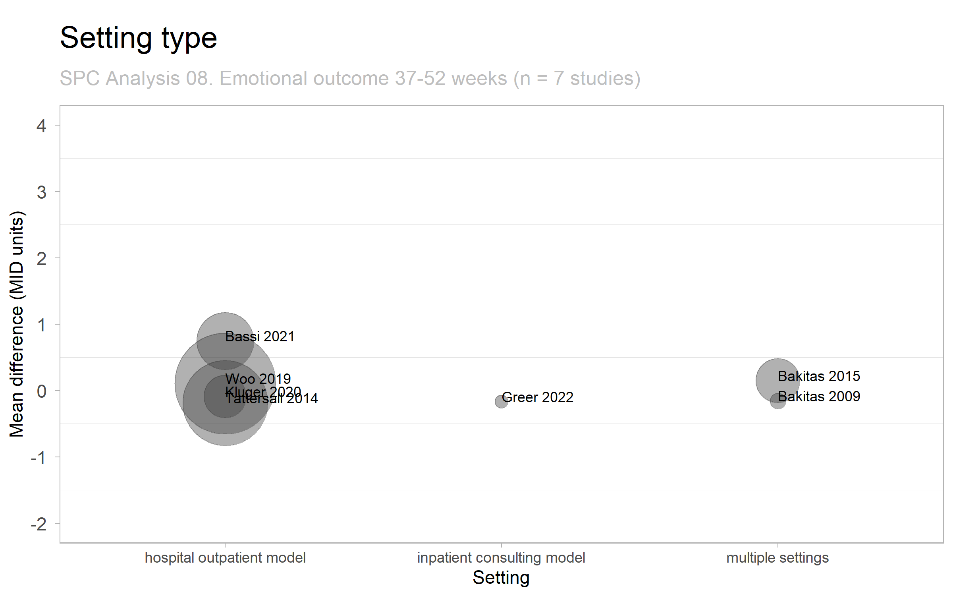 |
| **Type of intervention** | *F*(3,3) = 0.551  *p* = 0.682 | 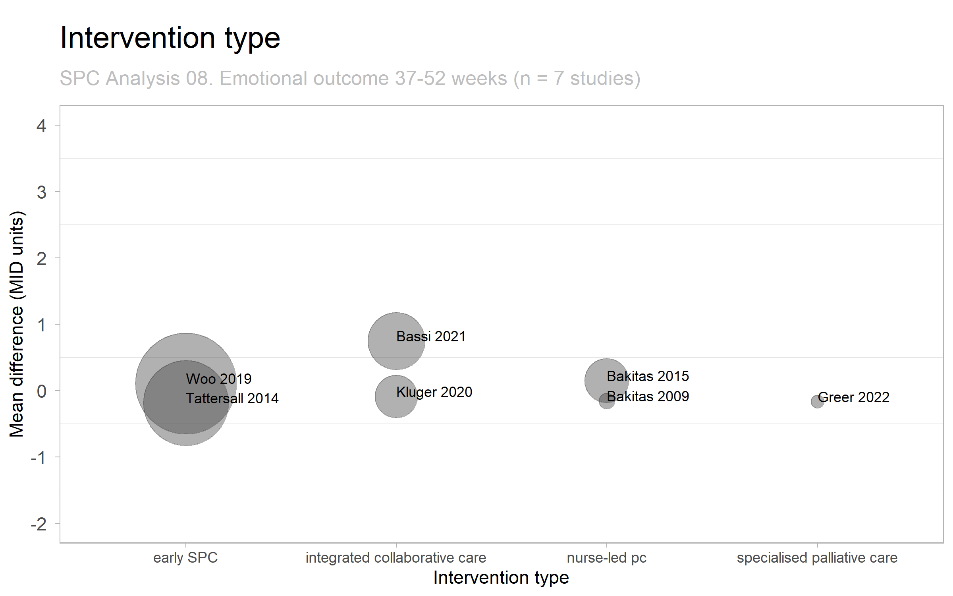 |
| **Year** | *F*(1,5) = 3.578  *p* = 0.117 | 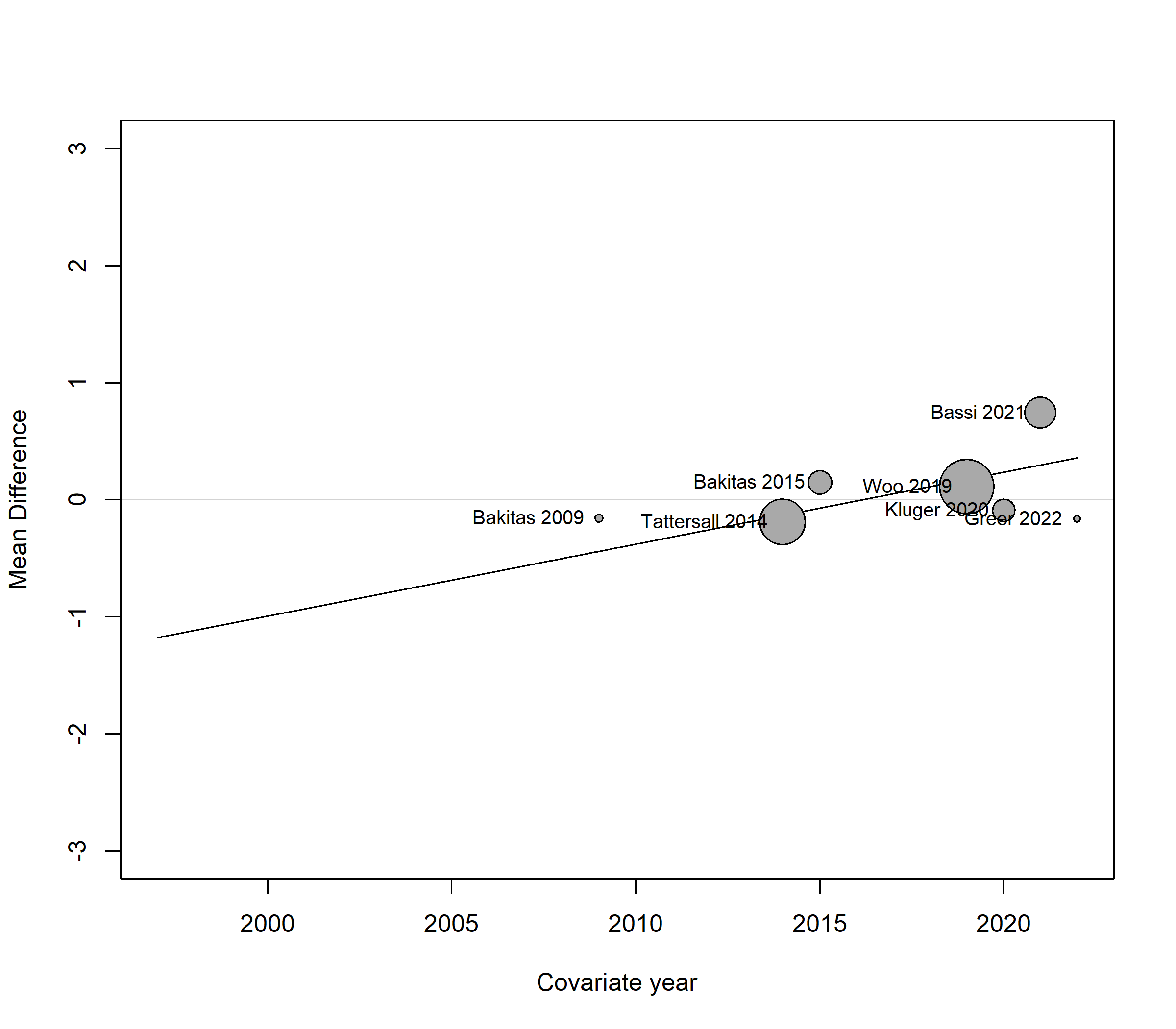 |
