# Supplementary material for: Benefits of specialist palliative care by identifying active ingredients of service composition, structure, and delivery model: A systematic review with meta-analysis and meta-regression
Source: PLoS Med. 2024 Aug 2;21(8):e1004436. doi: 10.1371/journal.pmed.1004436 (PMC11329153; doi:10.1371/journal.pmed.1004436)
Supplement: S10 Appendix — (DOCX) [file pmed.1004436.s010.docx]

**Benefits of specialist palliative care by identifying active ingredients of service composition, structure, and delivery model: A systematic review with meta-analysis and meta-regression**

**S10 Appendix**

Miriam J. Johnson, Leah Rutherford, Anisha Sunny, Sophie Pask, Susanne de Wolf-Linder, Fliss E. M. Murtagh, Christina Ramsenthaler

[hycr22@hyms.ac.uk](mailto:hycr22@hyms.ac.uk)

# Sensitivity analysis with different MIDs

## Fig A and Table A: Quality of life at 12 to 16 weeks using the best MID

**Analysis with effect size SMD (MID units)**

| **Study (k = 34)** | **MD (MID)** | **95% CI** | **weight (random, in %)** |  |
| --- | --- | --- | --- | --- |
| Bakitas *et al* 2009^55^ | 0.74 | 0.04 to 1.44 | 3.5 |  |
| Bakitas *et al* 2015^56^ | 0.03 | -0.66 to 0.72 | 3.6 |  |
| Bakitas *et al* 2020^57^ | 0.34 | -0.66 to 1.34 | 3.1 |  |
| Bekelman *et al* 2018^58^ | 0.36 | -0.69 to 1.41 | 3.0 |  |
| Benthien *et al* 2020^59^ | -0.18 | -0.93 to 0.57 | 3.5 |  |
| Brims *et al* 2019^51^ | 0.39 | -1.47 to 2.24 | 1.8 |  |
| do Carmo *et al* 2017^33^ | 1.97 | -2.13 to 6.06 | 0.6 |  |
| Edmonds *et al* 2010^60^ | 1.05 | 0.25 to 1.85 | 3.4 |  |
| El-Jahwari *et al* 2016^34^ | 1.58 | -0.54 to 3.69 | 1.6 |  |
| El-Jahwari *et al* 2021^35^ | 0.79 | -0.23 to 1.80 | 3.0 |  |
| Evans *et al* 2021^61^ | 1.01 | 0.32 to 1.70 | 3.6 |  |
| Eychmüller *et al* 2021^40^ | 0.01 | -0.30 to 0.32 | 4.0 |  |
| Franciosi *et al* 2019^62^ | 0.20 | -0.53 to 0.94 | 3.5 |  |
| Gao *et al* 2020^63^ | 0.46 | -0.22 to 1.13 | 3.6 |  |
| Given *et al* 2002^64^ | 0.50 | -0.29 to 1.29 | 3.4 |  |
| Goldstein *et al* 2022^52^ | 4.28 | 4.01 to 4.55 | 4.1 |  |
| Greer *et al* 2022^36^ | -0.26 | -1.26 to 0.73 | 3.1 |  |
| Groenvold *et al* 2017^65^ | 0.74 | -0.35 to 1.76 | 3.0 |  |
| Hoek *et al* 2017^66^ | 0.20 | -1.58 to 1.99 | 1.9 |  |
| Liu *et al* 2022^32^ | 0.26 | -0.35 to 0.87 | 3.7 |  |
| Maltoni *et al* 2016^42^ | 0.53 | -0.27 to 1.33 | 3.4 |  |
| Nottelmann *et al* 2021^43^ | 0.00 | -1.58 to 1.58 | 2.2 |  |
| Patil *et al* 2021^67^ | 0.11 | -1.13 to 1.36 | 2.7 |  |
| Rogers *et al* 2017^68^ | 0.28 | -1.35 to 1.91 | 2.1 |  |
| Scarpi *et al* 2019^44^ | 0.47 | -0.74 to 1.69 | 2.7 |  |
| Sidebottom *et al* 2015^37^ | 0.75 | 0.05 to 1.46 | 3.5 |  |
| Slama *et al* 2020^45^ | 0.32 | -1.24 to 1.88 | 2.2 |  |
| Tattersall *et al* 2014^46^ | -0.02 | -0.14 to 0.10 | 4.2 |  |
| Temel *et al* 2010^47^ | 1.84 | -0.61 to 4.29 | 1.3 |  |
| Temel *et al* 2020^69^ | 0.60 | -0.12 to 1.32 | 3.5 |  |
| Vanbutsele *et al* 2020^70^ | 1.82 | 0.45 to 3.19 | 2.5 |  |
| Wong *et al* 2016^53^ | 1.16 | -0.05 to 2.37 | 2.7 |  |
| Woo *et al* 2019^48^ | 0.48 | -0.81 to 1.76 | 2.6 |  |
| Zimmermann *et al* 2014^49^ | 0.91 | 0.29 to 1.53 | 3.7 |  |
|  |  |  |  |  |
| ***Meta-analysis*** | **SMD (MID)** | **95% CI** | ***t*** | ***p*** |
| Random effects model | 0.66 | 0.35 to 0.98 | 4.310 | **<0.001** |
|  |  |  |  |  |
| ***Heterogeneity*** |  |  | ***Q (df)*** | ***p*** |
| *τ²* | 0.72 | 0.33 to 1.02 | 857.01 (33) | **<0.001** |
| *I²* | 96% | 95.3 to 96.8% |  |  |
| *H* | 5.10 | 4.63 to 5.61 |  |  |

**Forest plot**


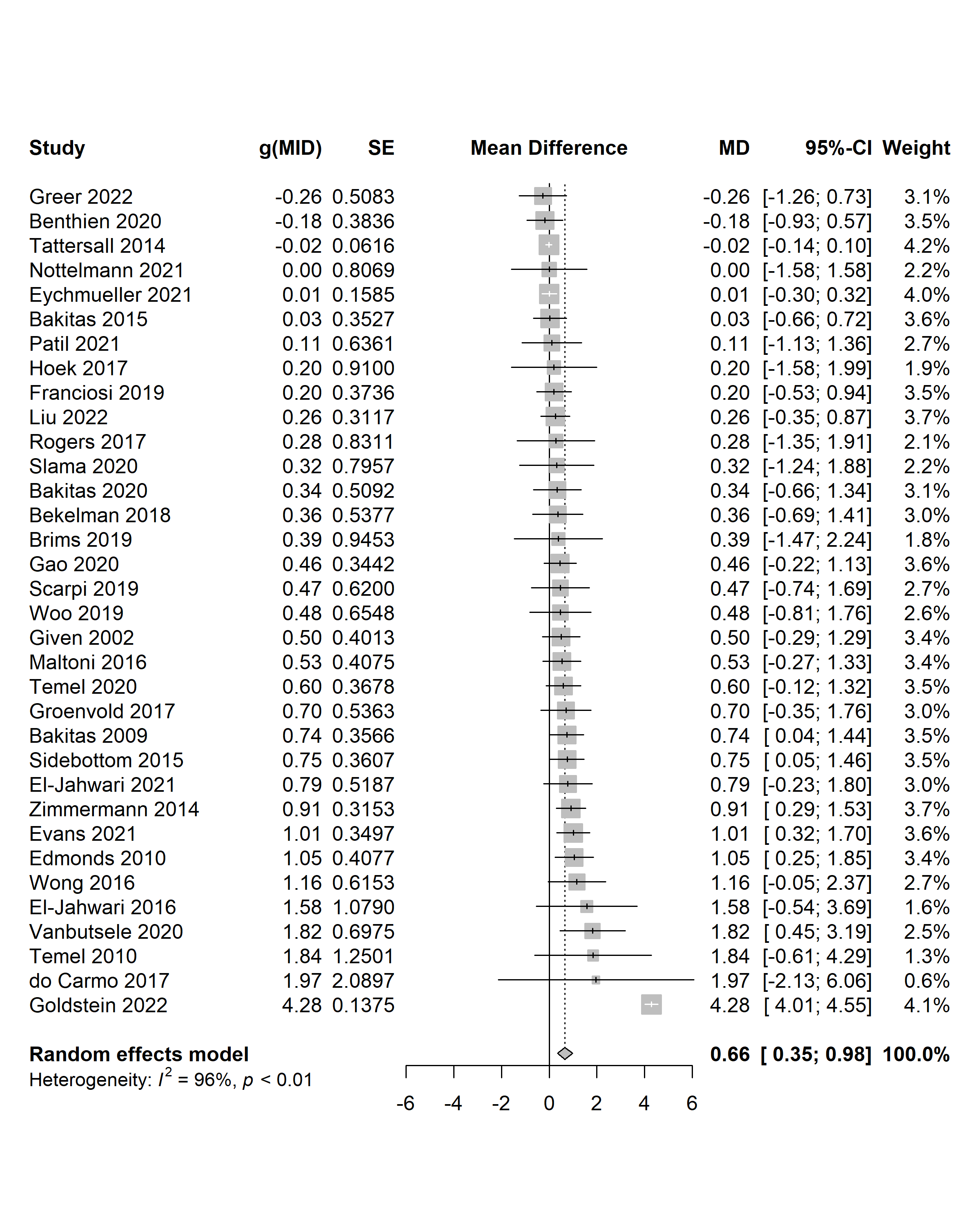


**Publication bias**

Egger’s enhanced funnel plot

| Linear regression test of funnel plot asymmetry  Intercept: 0.273  95% CI: -2.891 to 3.437  *t*(33) = 0.169, *p* = 0.867 | 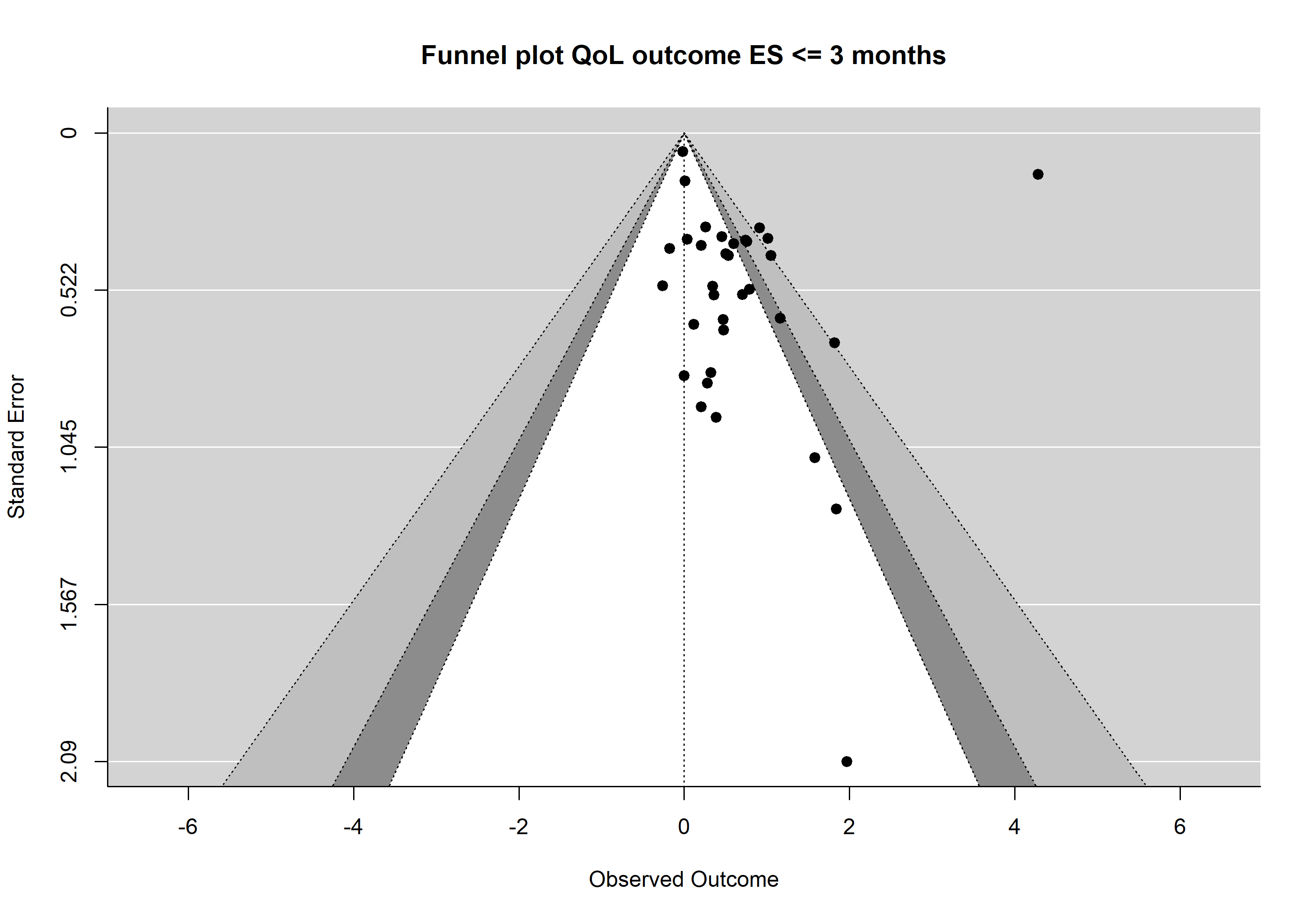 |
| --- | --- |

**Analysis with effect size RR (achieving change ≥1 MID)**

Number of studies combined: k = 34

Number of observations: 5473; Number of events: 2158

| **Study (k = 34)** | **RR** | **95% CI** | **weight (random, in %)** |  |
| --- | --- | --- | --- | --- |
| Bakitas *et al* 2009^55^ | 1.27 | 0.91 to 1.78 | 3.8 |  |
| Bakitas *et al* 2015^56^ | 1.10 | 0.66 to 1.83 | 2.6 |  |
| Bakitas *et al* 2020^57^ | 1.08 | 0.82 to 1.43 | 4.2 |  |
| Bekelman *et al* 2018^58^ | 1.08 | 0.82 to 1.43 | 4.2 |  |
| Benthien *et al* 2020^59^ | 0.94 | 0.69 to 1.30 | 3.9 |  |
| Brims *et al* 2019^51^ | 1.05 | 0.68 to 1.63 | 3.0 |  |
| do Carmo *et al* 2017^33^ | 1.27 | 0.48 to 3.33 | 1.1 |  |
| Edmonds *et al* 2010^60^ | 2.52 | 0.78 to 8.13 | 0.8 |  |
| El-Jahwari *et al* 2016^34^ | 1.35 | 0.64 to 2.85 | 1.6 |  |
| El-Jahwari *et al* 2021^35^ | 1.30 | 0.80 to 2.12 | 2.8 |  |
| Evans *et al* 2021^61^ | 2.09 | 0.59 to 7.38 | 0.7 |  |
| Eychmüller *et al* 2021^40^ | 1.11 | 0.47 to 2.63 | 1.3 |  |
| Franciosi *et al* 2019^62^ | 1.07 | 0.73 to 1.56 | 3.4 |  |
| Gao *et al* 2020^63^ | 1.13 | 0.89 to 1.43 | 4.5 |  |
| Given *et al* 2002^64^ | 1.33 | 0.85 to 2.07 | 3.0 |  |
| Goldstein *et al* 2022^52^ | 27.40 | 8.12 to 92.51 | 0.7 |  |
| Greer *et al* 2022^36^ | 0.85 | 0.52 to 1.37 | 2.8 |  |
| Groenvold *et al* 2017^65^ | 1.14 | 0.89 to 1.47 | 4.4 |  |
| Hoek *et al* 2017^66^ | 1.17 | 0.54 to 2.50 | 1.6 |  |
| Liu *et al* 2022^32^ | 1.16 | 0.75 to 1.80 | 3.0 |  |
| Maltoni *et al* 2016^42^ | 1.08 | 0.61 to 1.89 | 2.3 |  |
| Nottelmann *et al* 2021^43^ | 0.99 | 0.78 to 1.26 | 4.5 |  |
| Patil *et al* 2021^67^ | 0.97 | 0.66 to 1.42 | 3.4 |  |
| Rogers *et al* 2017^68^ | 1.03 | 0.83 to 1.26 | 4.8 |  |
| Scarpi *et al* 2019^44^ | 1.20 | 0.78 to 1.85 | 3.1 |  |
| Sidebottom *et al* 2015^37^ | 1.21 | 0.94 to 1.56 | 4.4 |  |
| Slama *et al* 2020^45^ | 1.05 | 0.64 to 1.72 | 2.7 |  |
| Tattersall *et al* 2014^46^ | - | - | - |  |
| Temel *et al* 2010^47^ | 2.15 | 1.26 to 3.69 | 2.5 |  |
| Temel *et al* 2020^69^ | 1.32 | 0.93 to 1.89 | 3.6 |  |
| Vanbutsele *et al* 2020^70^ | 1.45 | 0.99 to 2.09 | 3.5 |  |
| Wong *et al* 2016^53^ | 1.38 | 0.90 to 2.10 | 3.2 |  |
| Woo *et al* 2019^48^ | 1.09 | 0.85 to 1.40 | 4.5 |  |
| Zimmermann *et al* 2014^49^ | 1.37 | 1.02 to 1.85 | 4.1 |  |
|  |  |  |  |  |
| ***Meta-analysis*** | **RR** | **95% CI** | ***t*** | ***p*** |
| Random effects model | 1.20 | 1.07 to 1.35 | 3.150 | **0.004** |
|  |  |  |  |  |
| ***Heterogeneity*** |  |  | ***Q (df)*** | ***p*** |
| *τ²* | 0.06 | 0.00 to 0.28 | 46.93 (32) | **0.040** |
| *I²* | 31.8% | 0.0 to 55.6% |  |  |
| *H* | 1.21 | 1.00 to 1.50 |  |  |

**Forest plot of RR effect size for the QoL outcome at 12 weeks**


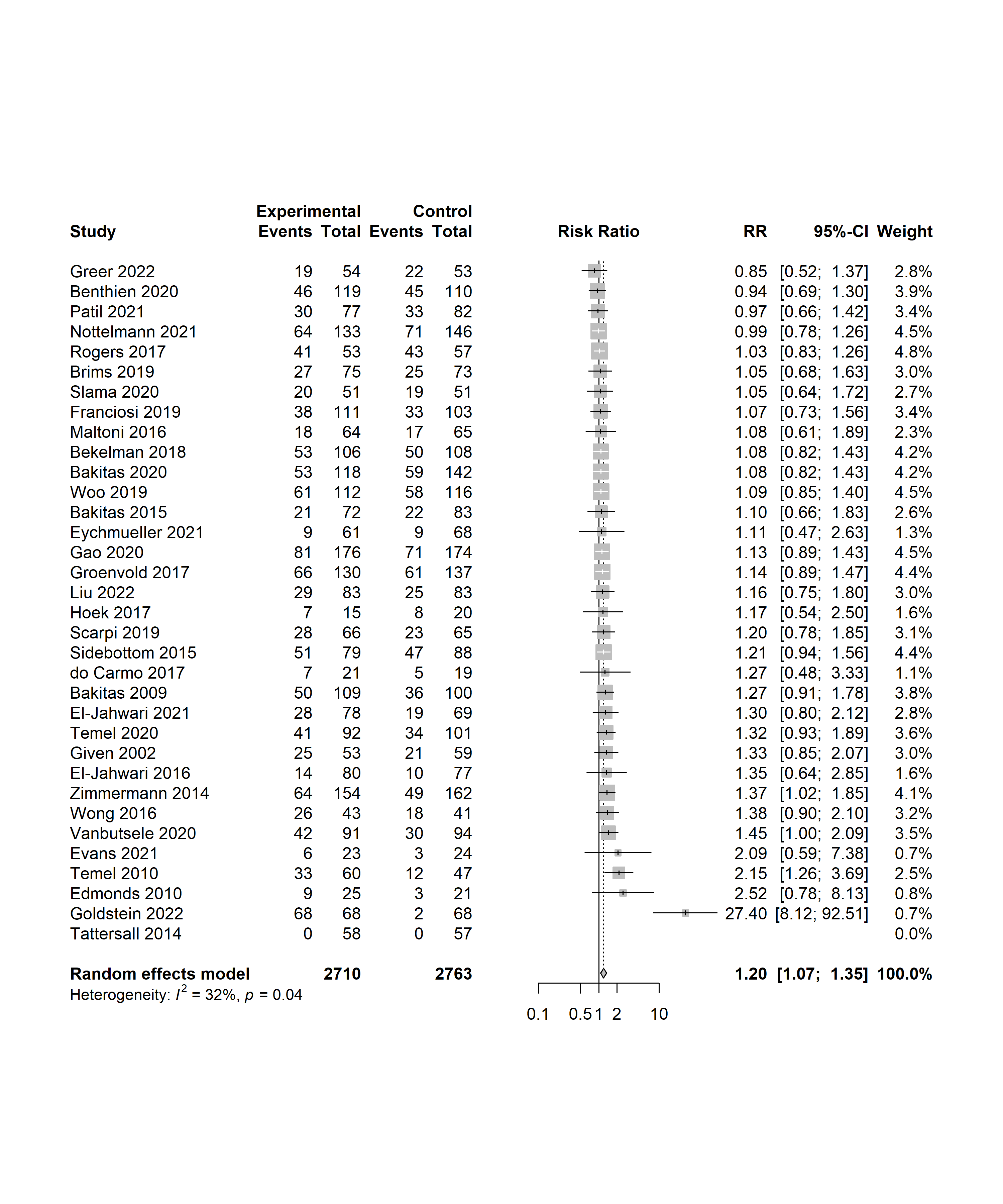


The risk ratio of RR = 1.20 (95% CI = 1.07 to 1.35) translates into a point estimate of a 20% increased probability of experiencing a change in QOL of at least 1 MID size with SPC. This is statistically significant.

The number needed to treat is calculated as follows (*p_CG_* is the baseline risk of experiencing change of at least 1 MID in the control group):

$$\frac{1}{p_{CG}\cdot(1-RR)}=\frac{1}{\frac{983}{2763}\cdot0.20}=14.05 \to14$$

The NNT is 14, meaning that 14 people need to be treated with SPC in order for one person to have a change in QOL at 12 weeks of at least 1 MID.

## Fig B and Table B: Emotional wellbeing at 12 to 16 weeks using the best MID

**Analysis with effect size SMD (MID units)**

| **Study (k = 23)** | **MD (MID)** | **95% CI** | **weight (random, in %)** |  |
| --- | --- | --- | --- | --- |
| Bakitas *et al* 2009^55^ | -0.13 | -0.88 to 0.63 | 4.6 |  |
| Bakitas *et al* 2015^56^ | 0.37 | -0.26 to 0.99 | 4.8 |  |
| Bakitas *et al* 2020^57^ | 0.14 | -0.54 to 0.83 | 4.7 |  |
| Bekelman *et al* 2018^58^ | 0.32 | -0.11 to 0.75 | 5.0 |  |
| Brims *et al* 2019^51^ | 0.00 | -0.87 to 0.87 | 4.4 |  |
| do Carmo *et al* 2017^33^ | 2.46 | -0.76 to 5.68 | 1.4 |  |
| Edmonds *et al* 2010^60^ | -0.11 | -0.62 to 0.39 | 5.0 |  |
| El-Jahwari *et al* 2016^34^ | 1.10 | 0.28 to 1.91 | 4.5 |  |
| El-Jahwari *et al* 2021^35^ | 1.44 | 0.69 to 2.20 | 4.6 |  |
| Evans *et al* 2021^61^ | 0.35 | -0.27 to 0.96 | 4.8 |  |
| Eychmüller *et al* 2021^40^ | 0.01 | -0.30 to 0.32 | 5.1 |  |
| Gao *et al* 2020^63^ | 0.33 | -0.48 to 1.13 | 4.5 |  |
| Given *et al* 2002^64^ | 0.17 | -1.56 to 1.89 | 3.0 |  |
| Greer *et al* 2022^36^ | -0.39 | -1.35 to 0.56 | 4.3 |  |
| Groenvold *et al* 2017^65^ | 0.78 | -0.08 to 1.63 | 4.4 |  |
| Hoek *et al* 2017^66^ | -0.02 | -1.78 to 1.74 | 2.9 |  |
| Liu *et al* 2022^32^ | 4.84 | 4.08 to 5.60 | 4.6 |  |
| Rogers *et al* 2017^68^ | 0.00 | - | 0.0 |  |
| Sidebottom *et al* 2015^37^ | 0.29 | 0.01 to 0.56 | 5.2 |  |
| Slama *et al* 2020^45^ | 0.18 | -0.96 to 1.31 | 4.0 |  |
| Tattersall *et al* 2014^46^ | -0.22 | -0.44 to 0.00 | 5.2 |  |
| Temel *et al* 2020^69^ | 0.61 | 0.05 to 1.18 | 4.9 |  |
| Woo *et al* 2019^48^ | 0.00 | -1.65 to 1.65 | 3.1 |  |
| Zimmermann *et al* 2014^49^ | 0.40 | 0.04 to 0.76 | 5.1 |  |
|  |  |  |  |  |
| ***Meta-analysis*** | **SMD (MID)** | **95% CI** | ***t*** | ***p*** |
| Random effects model | 0.56 | 0.04 to 0.98 | 2.270 | **0.033** |
|  |  |  |  |  |
| ***Heterogeneity*** |  |  | ***Q (df)*** | ***p*** |
| *τ²* | 0.99 | 0.51 to 2.17 | 184.83 (22) | **<0.001** |
| *I²* | 88% | 84.5 to 91.4% |  |  |
| *H* | 2.90 | 2.46 to 3.42 |  |  |

**Forest plot**


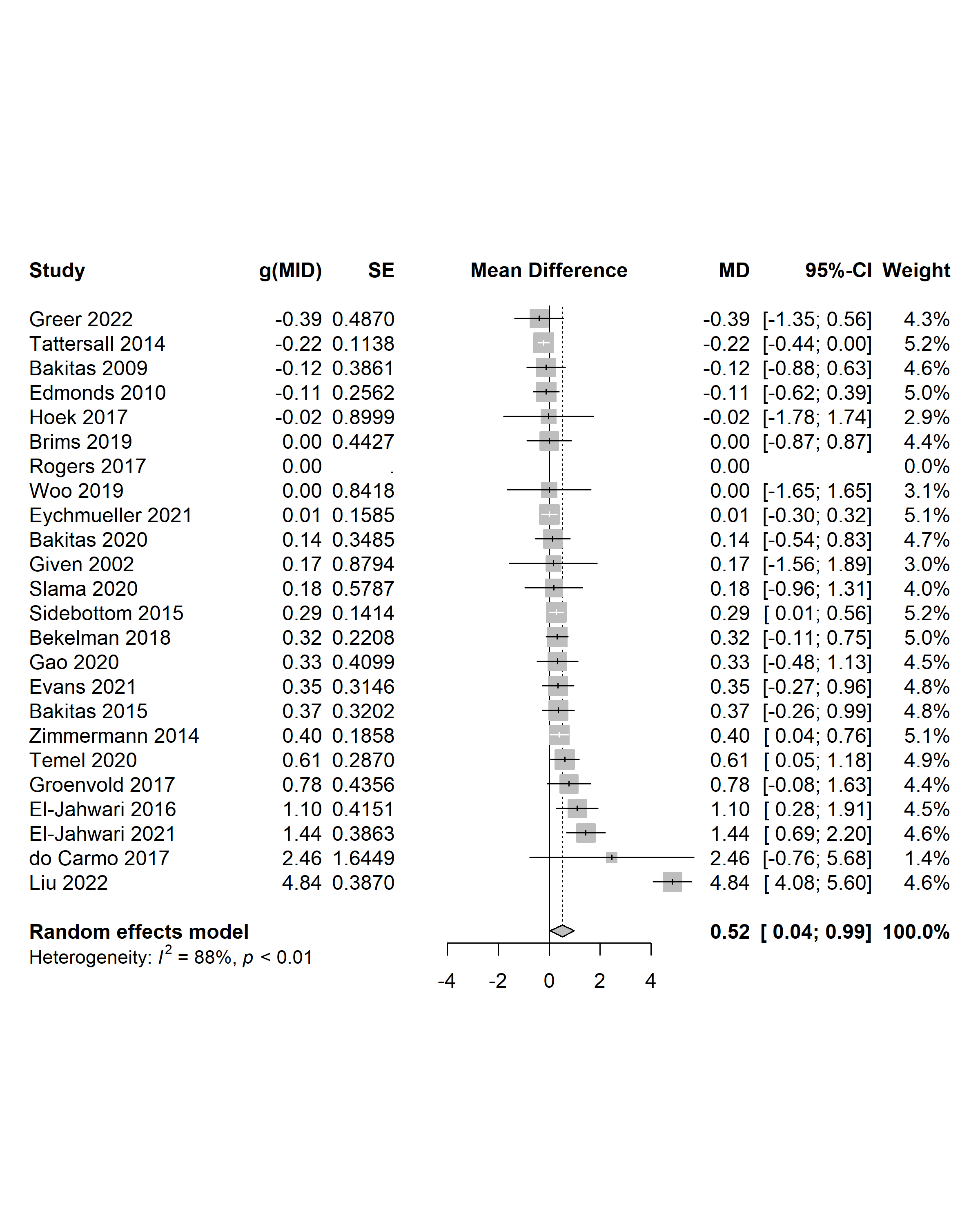


**Publication bias**

Egger’s enhanced funnel plot

| Linear regression test of funnel plot asymmetry  Intercept: 0.273  95% CI: -2.891 to 3.437  *t*(22) = 0.169, *p* = 0.867 | 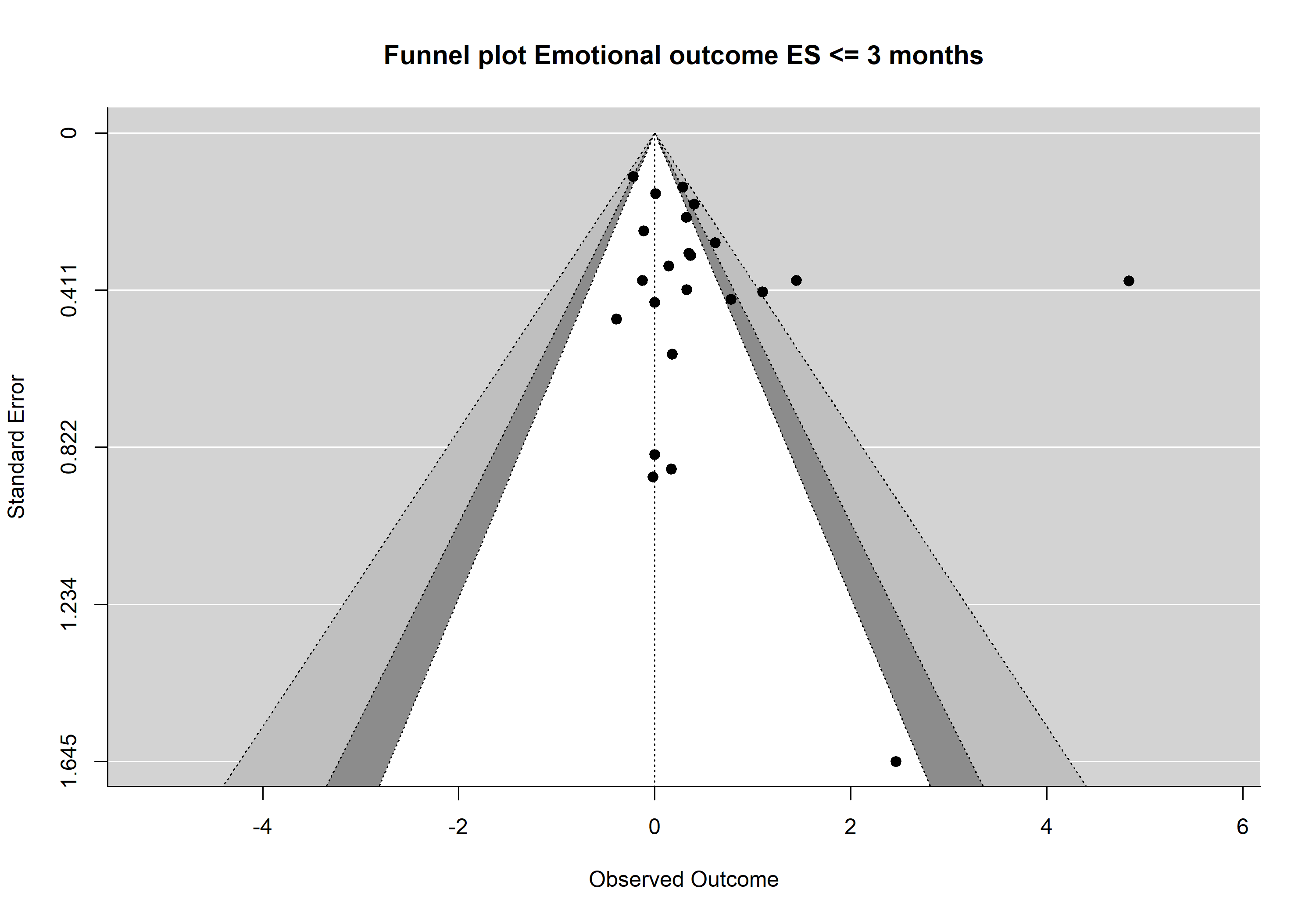 |
| --- | --- |

**Analysis with effect size RR (achieving change ≥1 MID)**

Number of studies combined: k = 23

Number of observations: 3765; Number of events: 1344

| **Study (k = 27)** | **RR** | **95% CI** | **weight (random, in %)** |  |
| --- | --- | --- | --- | --- |
| Bakitas *et al* 2009^55^ | 0.94 | 0.68 to 1.29 | 6.6 |  |
| Bakitas *et al* 2015^56^ | 1.18 | 0.86 to 1.62 | 6.5 |  |
| Bakitas *et al* 2020^57^ | 1.18 | 0.77 to 1.42 | 6.8 |  |
| Bekelman *et al* 2018^58^ | 1.27 | 0.85 to 1.88 | 5.1 |  |
| Brims *et al* 2019^51^ | 1.05 | 0.60 to 1.84 | 3.1 |  |
| do Carmo *et al* 2017^33^ | 0.05 | 0.27 to 2.07 | 1.1 |  |
| Edmonds *et al* 2010^60^ | 0.75 | 0.13 to 5.16 | 0.4 |  |
| El-Jahwari *et al* 2016^34^ | 1.81 | 0.97 to 2.45 | 4.1 |  |
| El-Jahwari *et al* 2021^35^ | 2.54 | 0.27 to 4.97 | 2.3 |  |
| Evans *et al* 2021^61^ | 1.51 | 0.35 to 3.13 | 1.0 |  |
| Eychmüller *et al* 2021^40^ | 1.04 | 0.47 to 2.63 | 1.6 |  |
| Gao *et al* 2020^63^ | 1.11 | 0.86 to 1.43 | 8.0 |  |
| Given *et al* 2002^64^ | 1.03 | 0.70 to 1.52 | 5.3 |  |
| Greer *et al* 2022^36^ | 0.88 | 0.53 to 1.47 | 3.6 |  |
| Groenvold *et al* 2017^65^ | 1.19 | 0.93 to 1.53 | 8.2 |  |
| Hoek *et al* 2017^66^ | 1.02 | 0.50 to 2.06 | 2.2 |  |
| Liu *et al* 2022^32^ | 2.19 | 1.72 to 2.79 | 8.3 |  |
| Rogers *et al* 2017^68^ | - | - | 0.0 |  |
| Sidebottom *et al* 2015^37^ | 1.50 | 0.92 to 2.44 | 3.9 |  |
| Slama *et al* 2020^45^ | 1.11 | 0.67 to 1.84 | 3.7 |  |
| Tattersall *et al* 2014^46^ | 0.33 | 0.07 to 1.56 | 0.5 |  |
| Temel *et al* 2020^69^ | 1.30 | 0.94 to 1.80 | 6.4 |  |
| Woo *et al* 2019^48^ | 1.08 | 0.79 to 1.48 | 6.6 |  |
| Zimmermann *et al* 2014^49^ | 1.49 | 0.99 to 2.25 | 4.9 |  |
|  |  |  |  |  |
| ***Meta-analysis*** | **RR** | **95% CI** | ***t*** | ***p*** |
| Random effects model | 1.23 | 1.08 to 1.39 | 3.470 | **0.002** |
|  |  |  |  |  |
| ***Heterogeneity*** |  |  | ***Q (df)*** | ***p*** |
| *τ²* | 0.03 | 0.00 to 0.11 | 40.86 (22) | **0.009** |
| *I²* | 46.2% | 12.2 to 67.0% |  |  |
| *H* | 1.36 | 1.07 to 1.74 |  |  |

**Forest plot of RR effect size for the QoL outcome at 12 weeks**


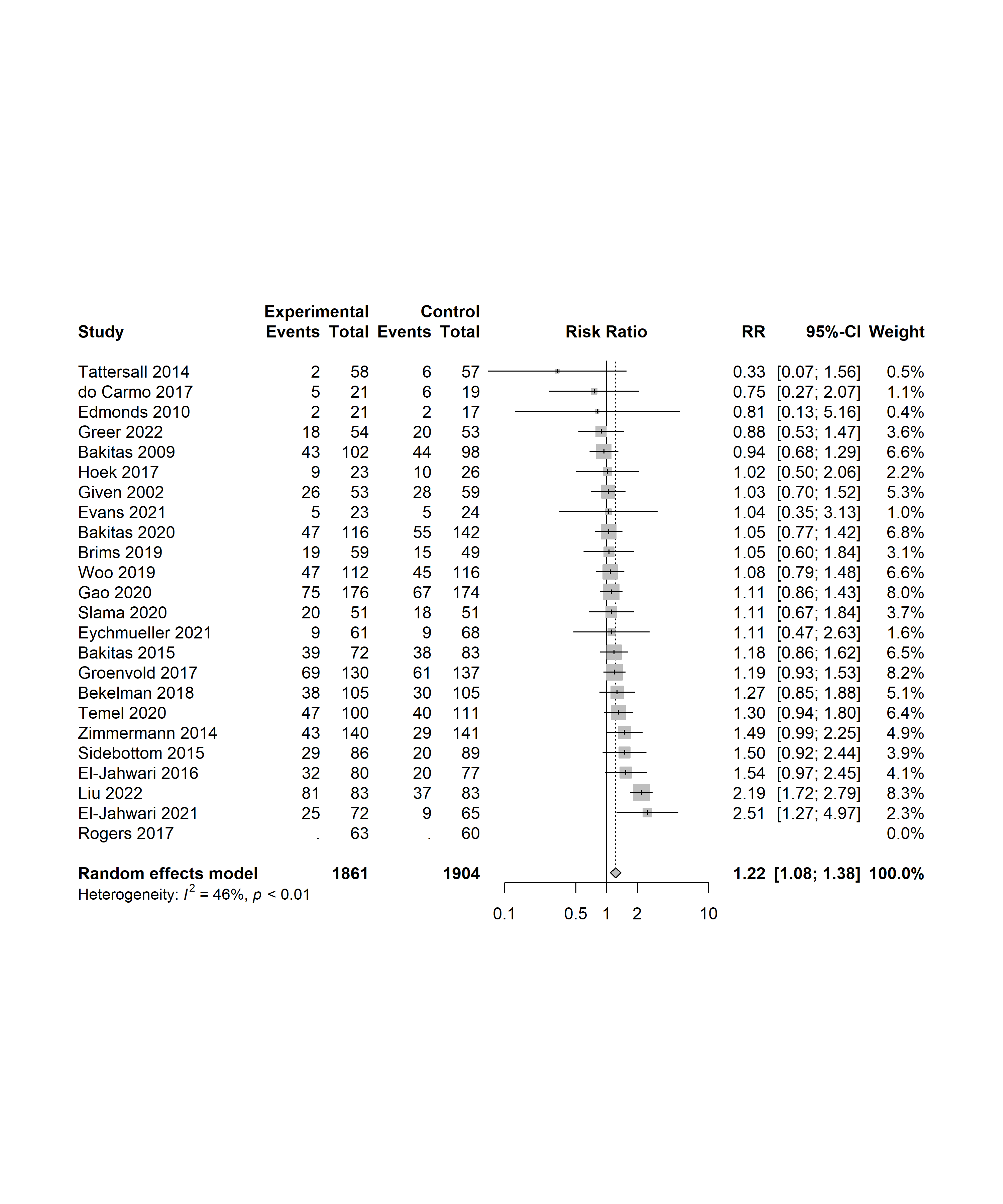


The risk ratio of RR = 1.22 (95% CI = 1.08 to 1.38) translates into a point estimate of a 22% increased probability of experiencing a change in QOL of at least 1 MID size with SPC. This is statistically significant.

The number needed to treat is calculated as follows (*p_CG_* is the baseline risk of experiencing change of at least 1 MID in the control group):

$$\frac{1}{p_{CG}\cdot(1-RR)}=\frac{1}{\frac{614}{1904}\cdot0.22}=14.094 \to14$$

The NNT is 14, meaning that 14 people need to be treated with SPC in order for one person to have a change in emotional wellbeing at 12 weeks of at least 1 MID.

## Fig C and Table C: Quality of life at 13 to 36 weeks

**Analysis with effect size SMD (MID units)**

| **Study (k = 35)** | **MD (MID)** | **95% CI** | **weight (random, in %)** |  |
| --- | --- | --- | --- | --- |
| Bakitas *et al* 2009^55^ | 0.02 | -0.77 to 0.82 | 3.3 |  |
| Bakitas *et al* 2015^56^ | -0.07 | -0.67 to 0.54 | 3.5 |  |
| Bakitas *et al* 2020^57^ | 0.36 | -0.67 to 1.39 | 2.9 |  |
| Bassi *et al* 2021^38^ | 0.70 | 0.44 to 0.96 | 3.9 |  |
| Bekelman *et al* 2018^58^ | 0.52 | -0.88 to 1.92 | 2.4 |  |
| Bekelman *et al* 2022^39^ | 0.85 | 0.30 to 1.41 | 3.6 |  |
| Benthien *et al* 2020^59^ | 0.37 | -0.57 to 1.32 | 3.1 |  |
| Braennstroem *et al* 2014^50^ | 0.86 | -0.14 to 1.86 | 3.0 |  |
| Brims *et al* 2019^51^ | -0.32 | -2.28 to 1.64 | 1.7 |  |
| do Carmo *et al* 2017^33^ | 5.23 | -0.35 to 10.81 | 0.3 |  |
| Edmonds *et al* 2010^60^ | -0.85 | -3.09 to 1.39 | 1.4 |  |
| El-Jahwari *et al* 2016^34^ | 0.78 | -1.19 to 2.75 | 1.7 |  |
| El-Jahwari *et al* 2021^35^ | 0.54 | -0.01 to 1.09 | 3.6 |  |
| Evans *et al* 2021^61^ | 0.71 | 0.12 to 1.30 | 3.6 |  |
| Eychmueller *et al* 2021^40^ | -0.32 | -1.26 to 0.61 | 3.1 |  |
| Franciosi *et al* 2019^62^ | 0.20 | -0.53 to 0.94 | 3.4 |  |
| Gao *et al* 2020^63^ | 0.46 | -0.22 to 1.13 | 3.4 |  |
| Given *et al* 2002^64^ | 0.50 | -0.29 to 1.29 | 3.3 |  |
| Goldstein *et al* 2022^52^ | 4.28 | 4.01 to 4.55 | 3.9 |  |
| Greer *et al* 2022^52^ | -0.01 | -1.22 to 1.20 | 2.6 |  |
| Hoek *et al* 2017^36^ | -1.33 | -3.45 to 0.80 | 1.5 |  |
| Kluger *et al* 2020^65^ | 0.39 | 0.03 to 0.74 | 3.8 |  |
| Maltoni *et al* 2016^66^ | 0.53 | -0.27 to 1.33 | 3.3 |  |
| Nottelmann *et al* 2021^41^ | 0.00 | -1.58 to 1.58 | 2.1 |  |
| Patil *et al* 2021^32^ | -0.33 | -1.43 to 0.76 | 2.8 |  |
| Rogers *et al* 2014^27^ | 1.26 | -0.58 to 3.10 | 1.8 |  |
| Scarpi *et al* 2019^43^ | 0.39 | -0.58 to 1.36 | 3.0 |  |
| Sidebottom *et al* 2015^67^ | 0.28 | -0.29 to 0.85 | 3.6 |  |
| Slama *et al* 2020^68^ | -0.11 | -2.08 to 1.85 | 1.7 |  |
| Tattersall *et al* 2014^44^ | -0.17 | -0.32 to -0.02 | 4.0 |  |
| Temel *et al* 2010^37^ | 1.84 | -0.61 to 4.29 | 1.3 |  |
| Temel *et al* 2020^69^ | 0.60 | -0.12 to 1.32 | 3.4 |  |
| Vanbutsele *et al* 2020^45^ | 1.93 | 0.65 to 3.21 | 2.5 |  |
| Woo *et al* 2019^46^ | 0.15 | -0.39 to 0.68 | 3.6 |  |
| Zimmermann *et al* 2014^47^ | 0.71 | 0.32 to 1.11 | 3.8 |  |
|  |  |  |  |  |
| ***Meta-analysis*** | **SMD (MID)** | **95% CI** | ***t*** | ***p*** |
| Random effects model | 0.53 | 0.20 to 0.87 | 3.230 | **0.003** |
|  |  |  |  |  |
| ***Heterogeneity*** |  |  | ***Q (df)*** | ***p*** |
| *τ²* | 0.75 | 0.39 to 1.33 | 852.26 (34) | **<0.001** |
| *I²* | 96.0% | 95.2 to 96.7% |  |  |
| *H* | 5.01 | 4.55 to 5.51 |  |  |

**Forest plot**


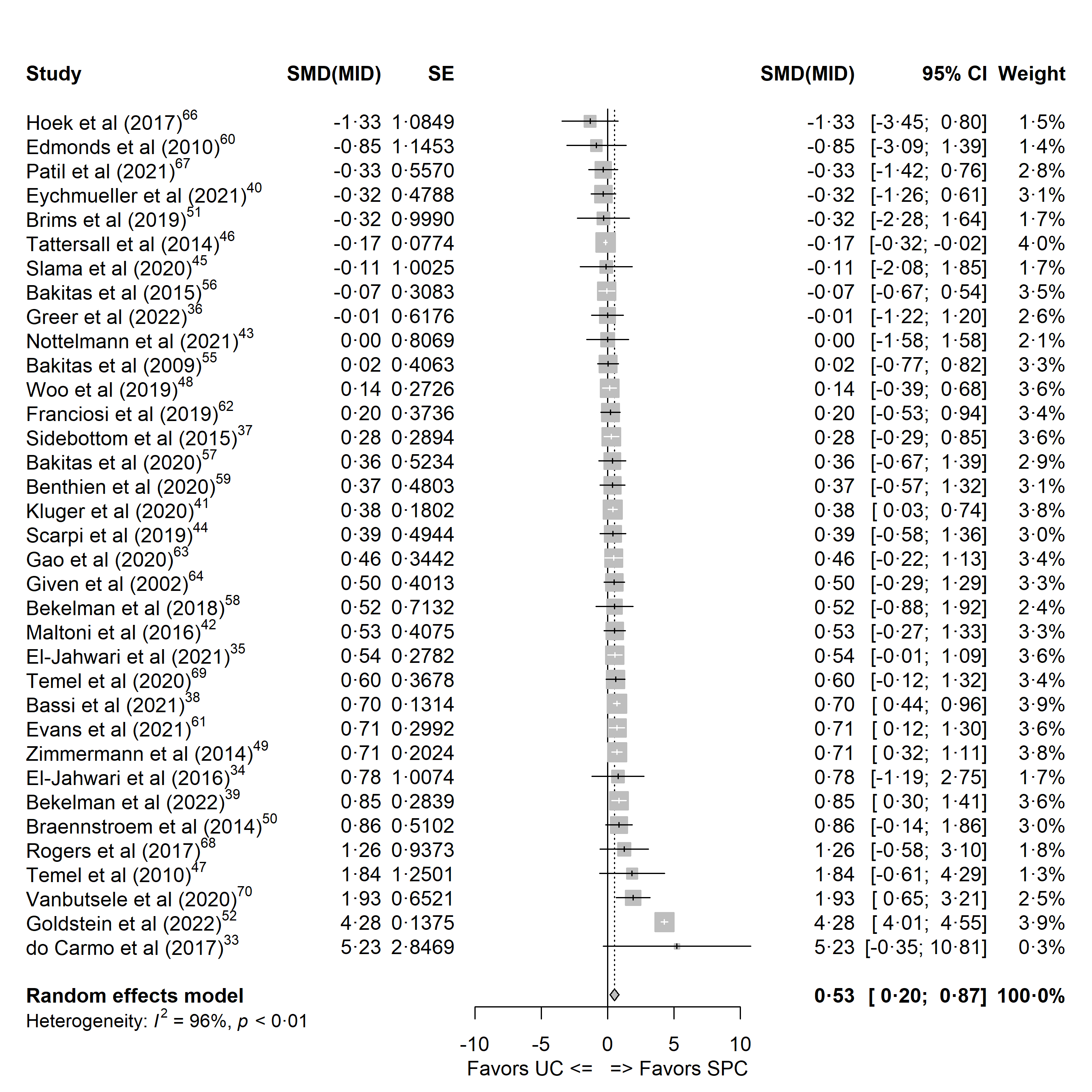


**Publication bias**

Egger’s enhanced funnel plot

| Linear regression test of funnel plot asymmetry  Intercept: -0.213  95% CI: -2.819 to 2.393  *t*(34) = -0.160, *p* = 0.874 | 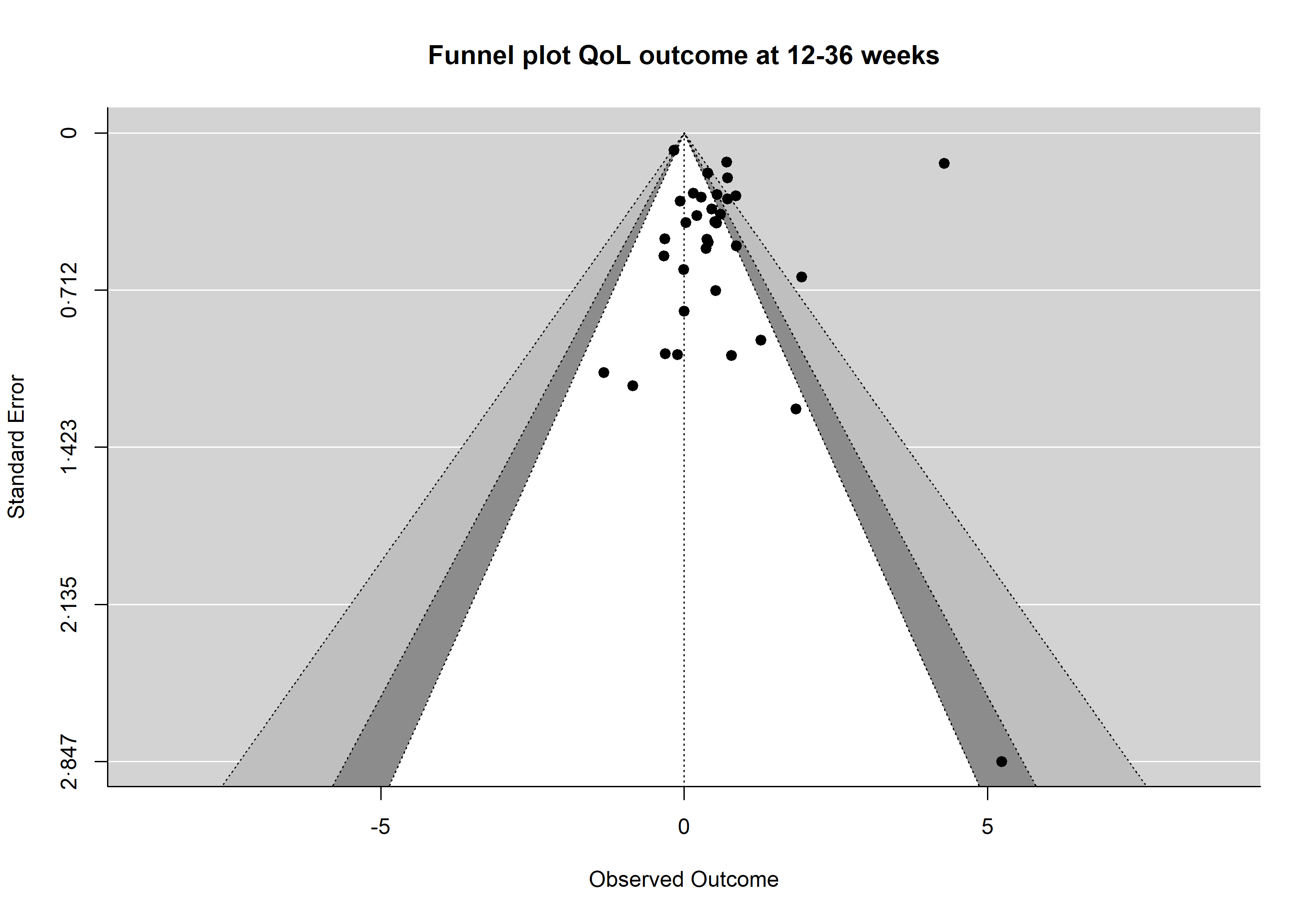 |
| --- | --- |

**Analysis with effect size RR (achieving change ≥1 MID)**

Number of studies combined: k = 35

Number of observations: 4822; Number of events: 1922

| **Study (k = 35)** | **RR** | **95% CI** | **weight (random, in %)** |  |
| --- | --- | --- | --- | --- |
| Bakitas *et al* 2009^55^ | 0.97 | 0.65 to 1.43 | 3.5 |  |
| Bakitas *et al* 2015^56^ | 0.97 | 0.64 to 1.46 | 3.3 |  |
| Bakitas *et al* 2020^57^ | 1.08 | 0.83 to 1.39 | 4.1 |  |
| Bassi *et al* 2021^38^ | 2.71 | 0.27 to 27.05 | 0.3 |  |
| Bekelman *et al* 2018^58^ | 1.07 | 0.83 to 1.39 | 4.1 |  |
| Bekelman *et al* 2022^39^ | 1.44 | 1.07 to 1.94 | 3.9 |  |
| Benthien *et al* 2020^59^ | 1.14 | 0.76 to 1.71 | 3.4 |  |
| Braennstroem *et al* 2014^50^ | 1.43 | 0.86 to 2.36 | 2.9 |  |
| Brims *et al* 2019^51^ | 0.92 | 0.59 to 1.43 | 3.2 |  |
| do Carmo *et al* 2017^33^ | 2.33 | 0.74 to 7.38 | 1.1 |  |
| Edmonds *et al* 2010^60^ | 0.73 | 0.24 to 2.24 | 1.1 |  |
| El-Jahwari *et al* 2016^34^ | 1.11 | 0.78 to 1.60 | 3.6 |  |
| El-Jahwari *et al* 2021^35^ | 1.42 | 0.88 to 2.31 | 3.0 |  |
| Evans *et al* 2021^61^ | 2.09 | 0.59 to 7.38 | 1.0 |  |
| Eychmueller *et al* 2021^40^ | 0.91 | 0.62 to 1.35 | 3.5 |  |
| Franciosi *et al* 2019^62^ | 1.07 | 0.73 to 1.56 | 3.5 |  |
| Gao *et al* 2020^63^ | 1.13 | 0.89 to 1.43 | 4.2 |  |
| Given *et al* 2002^64^ | 1.33 | 0.85 to 2.07 | 3.2 |  |
| Goldstein *et al* 2022^52^ | 27.40 | 8.12 to 92.51 | 1.0 |  |
| Greer *et al* 2022^52^ | 0.98 | 0.54 to 1.77 | 2.5 |  |
| Hoek *et al* 2017^36^ | 0.82 | 0.29 to 2.30 | 1.3 |  |
| Kluger *et al* 2020^65^ | 2.15 | 1.15 to 4.02 | 2.4 |  |
| Maltoni *et al* 2016^66^ | 1.08 | 0.61 to 1.89 | 2.6 |  |
| Nottelmann *et al* 2021^41^ | 0.99 | 0.78 to 1.26 | 4.2 |  |
| Patil *et al* 2021^32^ | 0.75 | 0.40 to 1.41 | 2.4 |  |
| Rogers *et al* 2014^27^ | 1.14 | 0.91 to 1.42 | 4.3 |  |
| Scarpi *et al* 2019^43^ | 1.22 | 0.77 to 1.94 | 3.1 |  |
| Sidebottom *et al* 2015^67^ | 1.11 | 0.84 to 1.47 | 4.0 |  |
| Slama *et al* 2020^68^ | 0.98 | 0.67 to 1.43 | 3.5 |  |
| Tattersall *et al* 2014^44^ | 0.68 | 0.06 to 7.21 | 0.3 |  |
| Temel *et al* 2010^37^ | 2.15 | 1.26 to 3.69 | 2.8 |  |
| Temel *et al* 2020^69^ | 1.32 | 0.93 to 1.89 | 3.6 |  |
| Vanbutsele *et al* 2020^45^ | 1.70 | 1.06 to 2.74 | 3.1 |  |
| Woo *et al* 2019^46^ | 0.94 | 0.56 to 1.62 | 2.7 |  |
| Zimmermann *et al* 2014^47^ | 1.85 | 1.22 to 2.83 | 3.3 |  |
|  |  |  |  |  |
| ***Meta-analysis*** | **RR** | **95% CI** | ***t*** | ***p*** |
| Random effects model | 1.22 | 1.06 to 1.40 | 2.810 | **0.008** |
|  |  |  |  |  |
| ***Heterogeneity*** |  |  | ***Q (df)*** | ***p*** |
| *τ²* | 0.10 | 0.02 to 0.35 | 60.76 (34) | **0.003** |
| *I²* | 44.0% | 16.4 to 62.5% |  |  |
| *H* | 1.34 | 1.09 to 1.63 |  |  |

**Forest plot of RR effect size for the QoL outcome 12 to 36 weeks**


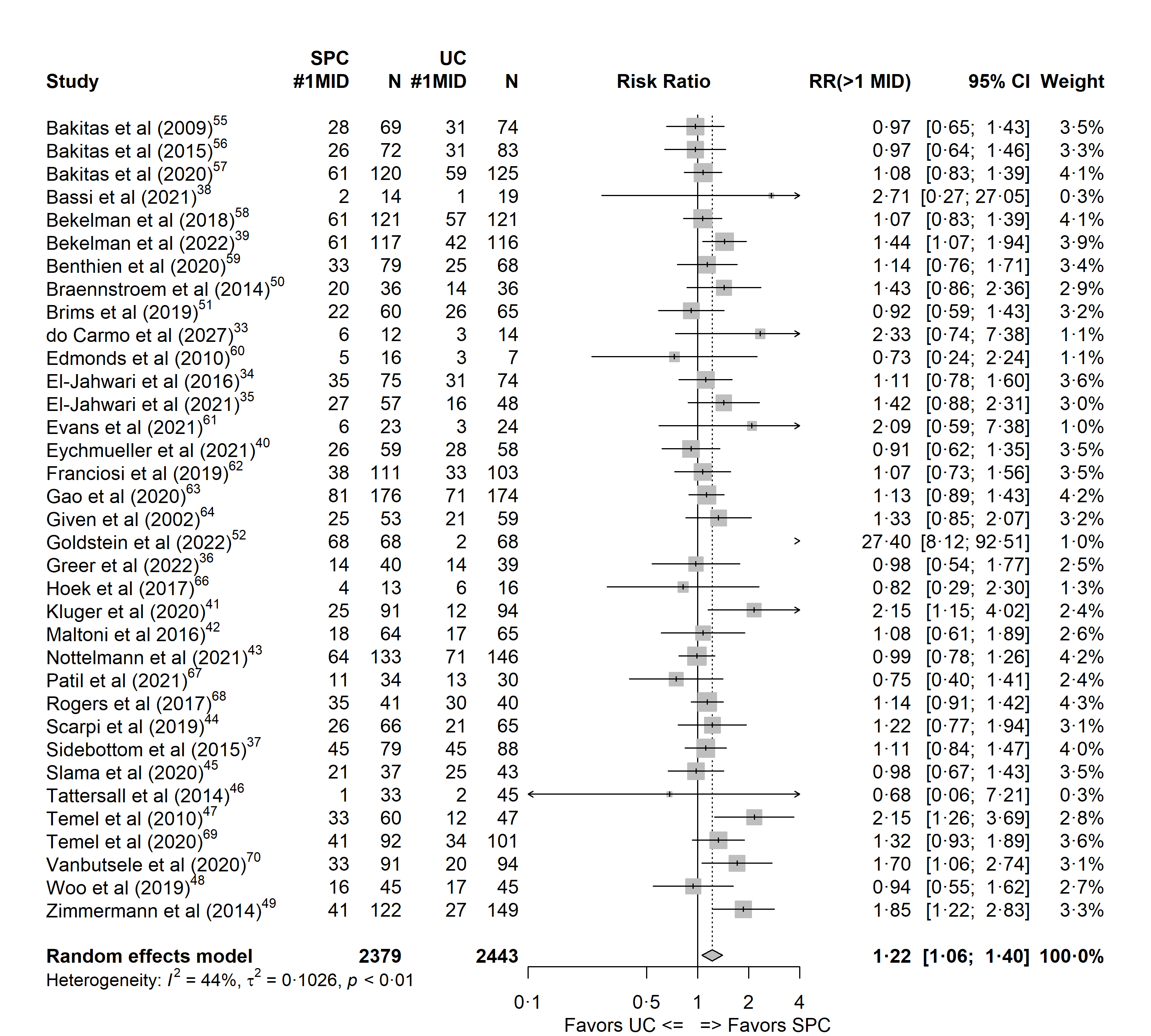


The risk ratio of RR = 1.22 (95% CI = 1.06 to 1.40) translates into a point estimate of a 22% increased probability of experiencing a change in QOL of at least 1 MID size with SPC. This is statistically significant.

The number needed to treat is calculated as follows (*p_CG_* is the baseline risk of experiencing change of at least 1 MID in the control group):

$$\frac{1}{p_{CG}\cdot(1-RR)}=\frac{1}{\frac{863}{2443}\cdot0.22}=12.867 \to13$$

The NNT is 13, meaning that 13 people need to be treated with SPC in order for one person to have a change in QOL at 12 to 36 weeks of at least 1 MID.

**Meta-regression: Univariate meta-regression analyses with covariates**

| *k = 35* | **Regression** | | | | | **Heterogeneity** | | | **Test of moderators** | |
| --- | --- | --- | --- | --- | --- | --- | --- | --- | --- | --- |
|  | *b* | *SE* | *t* | *p* | 95% CI | *I²* | *Q* | *p* | *F* | *p* |
| ***Attrition (in %)*** |  |  |  |  |  |  |  |  |  |  |
| Intercept | 0.69 | 0.13 | 5.132 | **0.001** | - | 22 | 40.2 | 0.151 | 7.887 | **0.008** |
| Attrition (in %) | -0.01 | 0.00 | -2.808 | **0.008** | -0.02; -0.00 |  |  |  |  |  |
| ***% advanced disease*** |  |  |  |  |  |  |  |  |  |  |
| Intercept | 0.41 | 0.24 | 1.708 | 0.105 | - | 54 | 57.6 | **0.001** | 0.103 | 0.752 |
| % advanced disease | -0.00 | 0.00 | -0.321 | 0.752 | -0.01; 0.01 |  |  |  |  |  |
| ***Disease group (ref: Cancer)*** |  |  |  |  |  |  |  |  |  |  |
| Intercept | 0.29 | 0.20 | 1.452 | 0.156 | - | 87 | 580.7 | **0.000** | 3.990 | 0.054 |
| Non-cancer | 0.65 | 0.33 | 1.997 | 0.054 | -0.01; 1.32 |  |  |  |  |  |
| ***RoB2 score (ref: low risk)*** |  |  |  |  |  |  |  |  |  |  |
| Intercept | 0.54 | 0.42 | 1.299 | 0.203 | - | 90 | 841.9 | **0.000** | 0.385 | 0.684 |
| RoB2: Some risk | 0.10 | 0.48 | 0.217 | 0.829 | -0.87; 1.07 |  |  |  |  |  |
| RoB2: High risk | -0.24 | 0.52 | -0.451 | 0.655 | -1.30; 0.83 |  |  |  |  |  |
| ***Service composition score*** |  |  |  |  |  |  |  |  |  |  |
| Intercept | -0.30 | 0.68 | -0.452 | 0.654 | - | 87 | 632.8 | **0.000** | 1.640 | 0.209 |
| Service composition score | 0.07 | 0.05 | 1.281 | 0.209 | -0.04; 0.18 |  |  |  |  |  |
| ***Setting (ref: multiple settings)*** |  |  |  |  |  |  |  |  |  |  |
| Intercept | 0.35 | 0.26 | 1.346 | 0.188 | - | 90 | 840.3 | **0.000** | 0.526 | 0.596 |
| Inpatient consulting model | 0.16 | 0.55 | 0.293 | 0.772 | -0.96; 1.28 |  |  |  |  |  |
| Home or hospital outreach | 0.37 | 0.36 | 1.024 | 0.314 | -0.36; 1.09 |  |  |  |  |  |
| ***Type of intervention (ref: SPC)*** |  |  |  |  |  |  |  |  |  |  |
| Intercept | 0.94 | 0.37 | 2.565 | **0.015** | - | 87 | 321.8 | **0.000** | 0.768 | 0.521 |
| Early SPC | -0.58 | 0.45 | -1.277 | 0.211 | -1.50; 0.34 |  |  |  |  |  |
| Integrated collaborative care | -0.30 | 0.49 | -0.603 | 0.551 | -1.30; 0.70 |  |  |  |  |  |
| Nurse-led palliative care | -0.75 | 0.59 | -1.265 | 0.215 | -1.95; 0.46 |  |  |  |  |  |
| ***Year*** |  |  |  |  |  |  |  |  |  |  |
| Intercept | -0.12 | 0.68 | -0.175 | 0.863 | - | 87 | 605.5 | **0.000** | 0.975 | 0.331 |
| Year | 0.04 | 0.03 | 0.987 | 0.331 | -0.04; 0.11 |  |  |  |  |  |

.

**Bubble plots of univariate meta-regression analyses**

| **Attrition** | *F*(1,32) = 7.887  *p* = **0.008** | 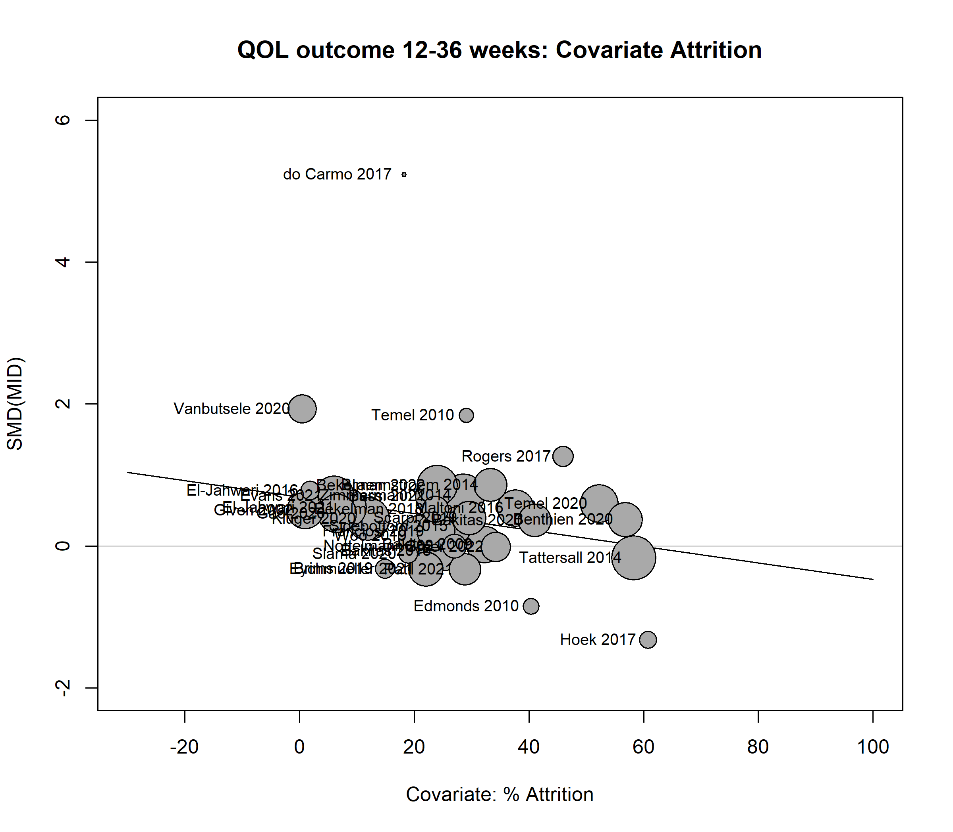 |
| --- | --- | --- |
| **% advanced disease** | *F*(1,18) = 0.103  *p* = 0.752 | 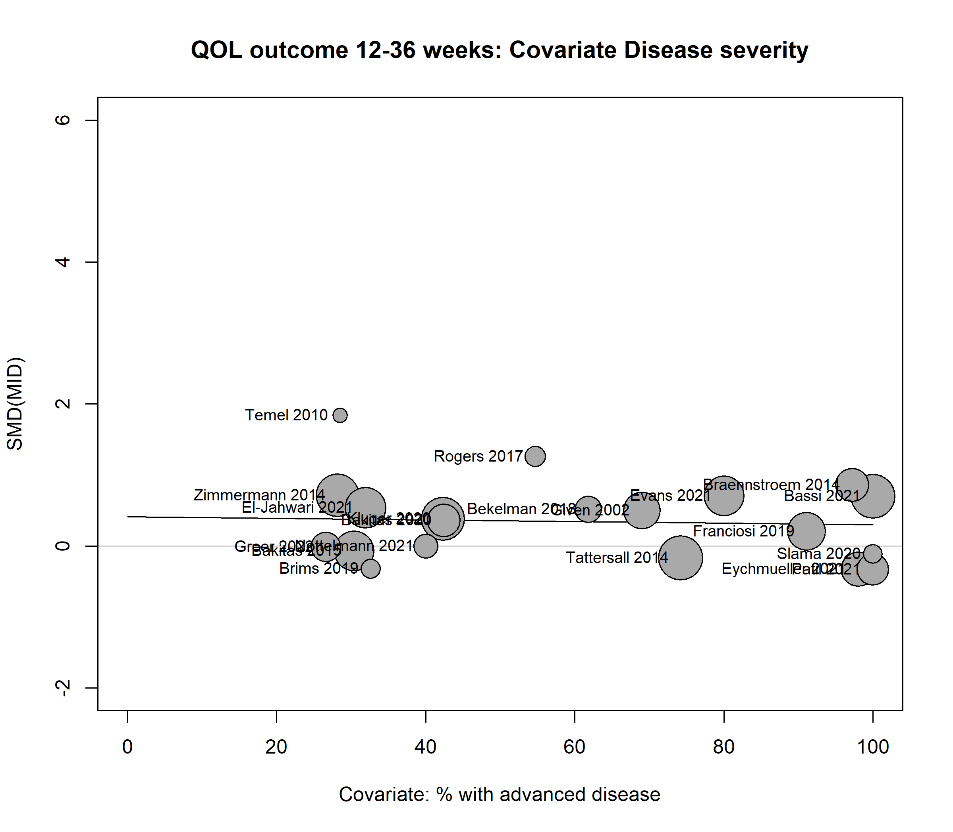 |
| **Disease group** | *F*(1,33) = 3.990  *p* = **0.054** | 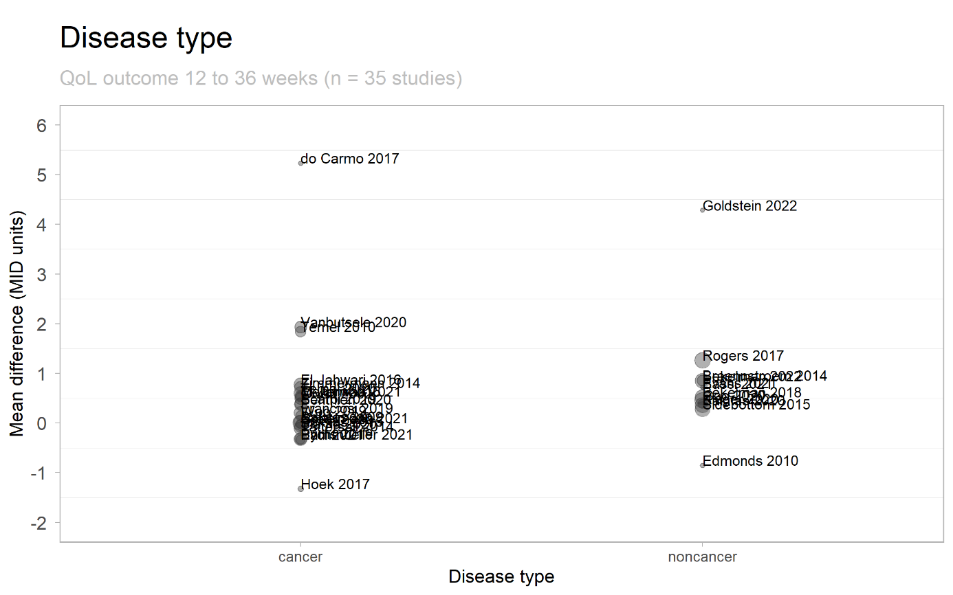 |
| **RoB2 score** | *F*(2,32) = 0.385  *p* = 0.684 | 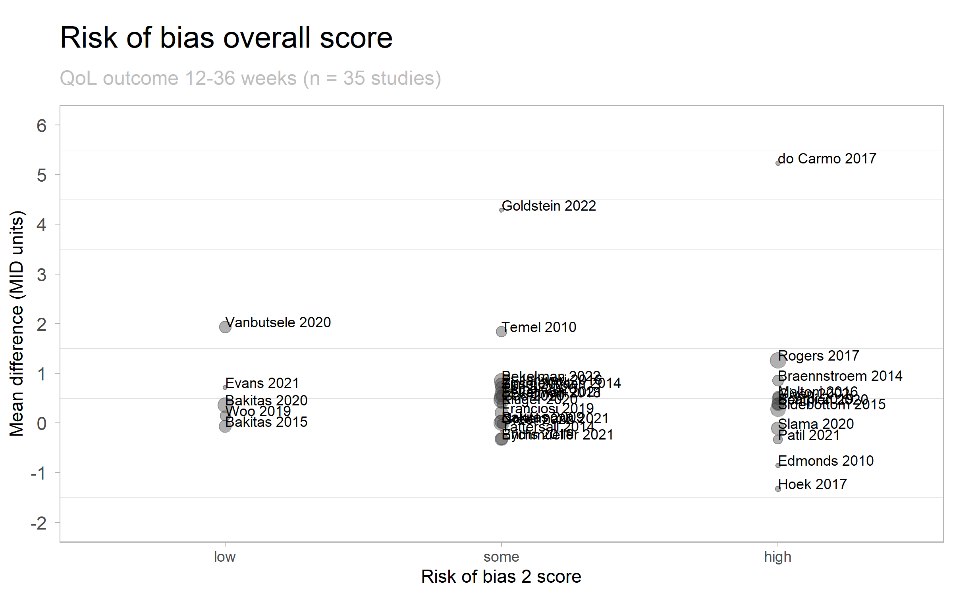 |
| **Service composition score** | *F*(1,33) = 1.640  *p* = 0.209 | 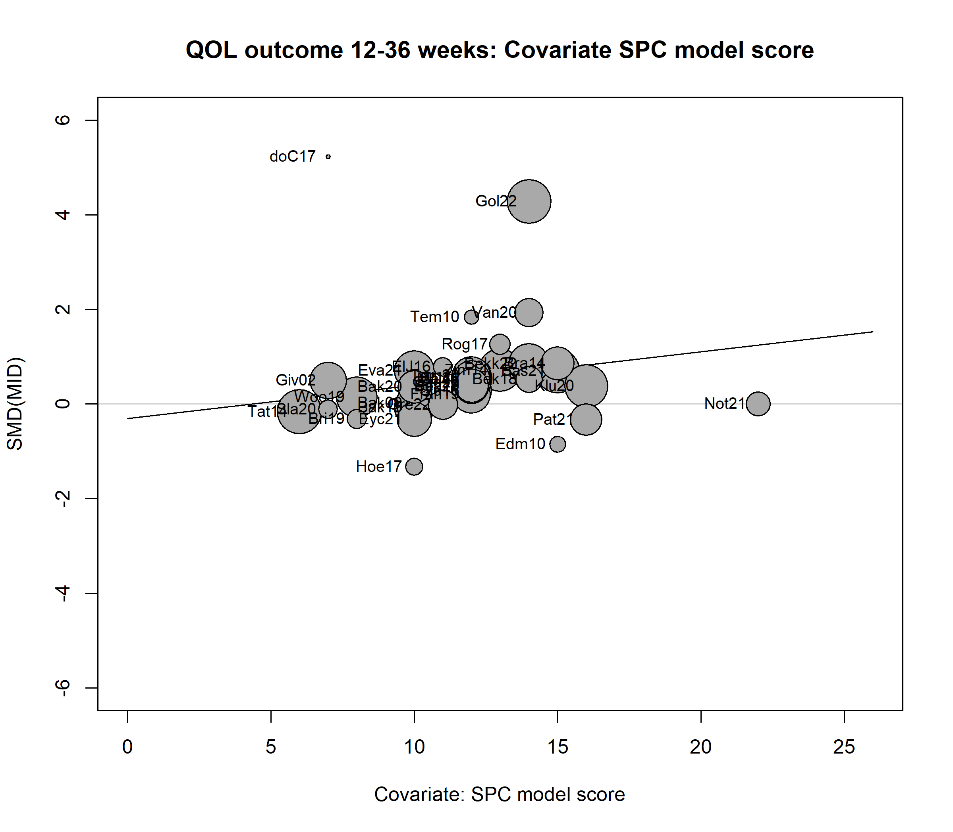 |
| **Setting** | *F*(2,32) = 0.526  *p* = 0.596 | 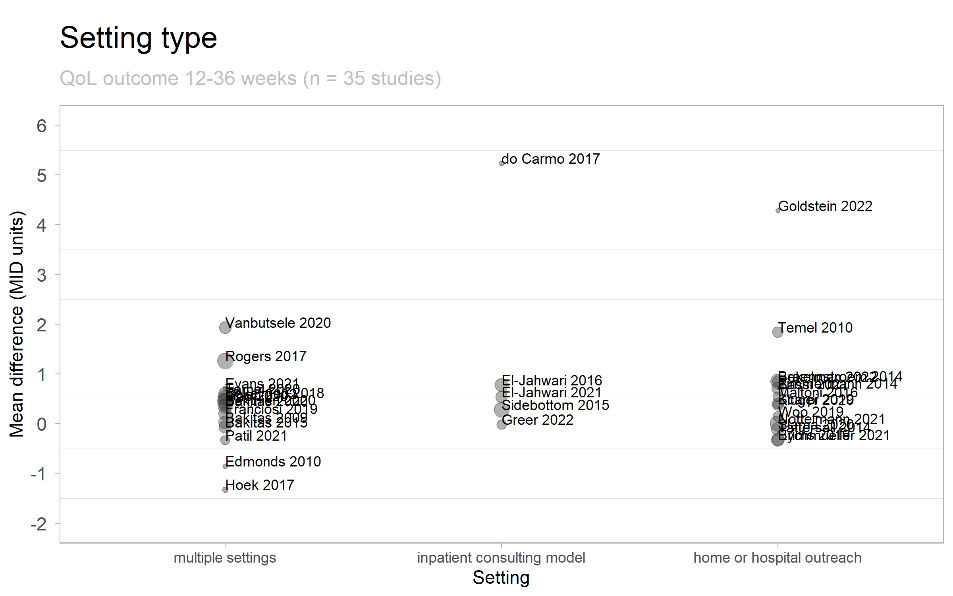 |
| **Type of intervention** | *F*(3,31) = 0.768  *p* = 0.521 | 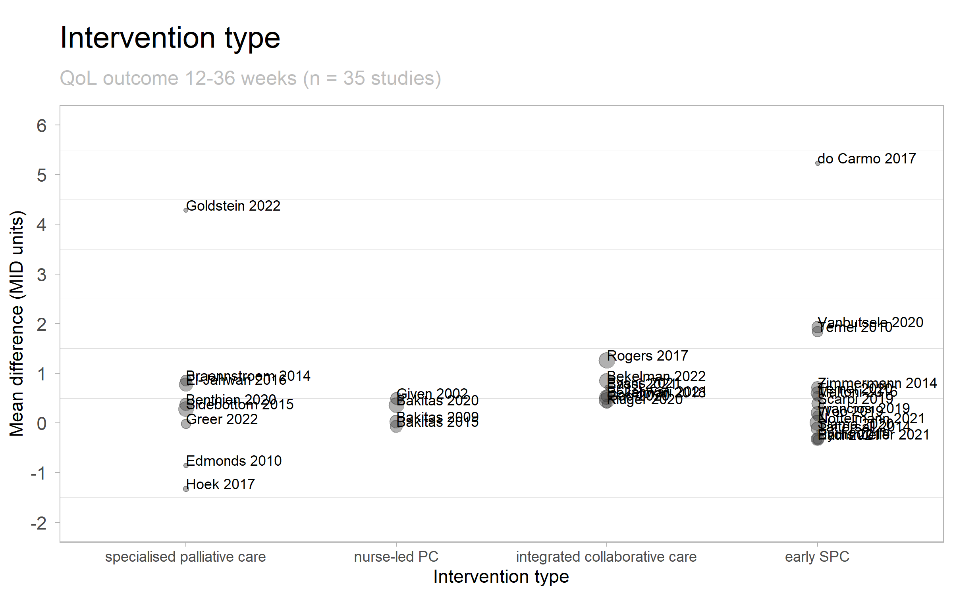 |
| **Year** | *F*(1,33) = 0.975  *p* = 0.331 | 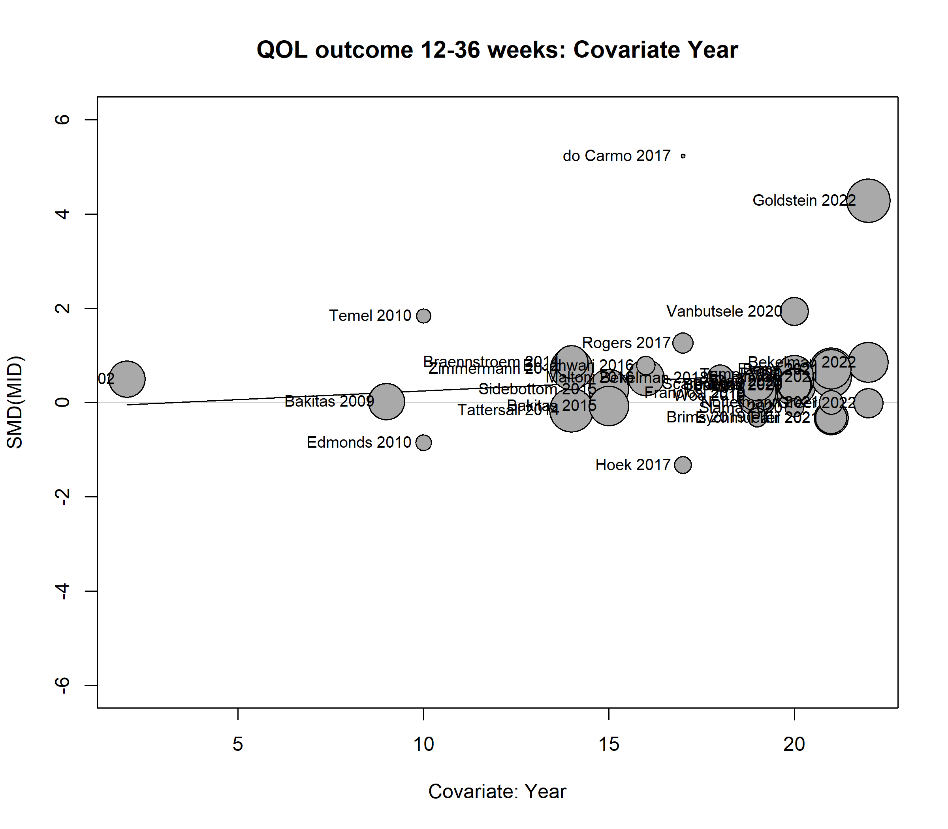 |

## Fig D and Table D: Emotional wellbeing at 13 to 36 weeks

**Analysis with effect size SMD (MID units)**

| **Study (k = 22)** | **MD (MID)** | **95% CI** | **weight (random, in %)** |  |
| --- | --- | --- | --- | --- |
| Bakitas *et al* 2009^55^ | 0.25 | -0.63 to 1.13 | 2.8 |  |
| Bakitas *et al* 2015^56^ | 0.37 | -0.26 to 0.99 | 4.3 |  |
| Bakitas *et al* 2020^57^ | 0.23 | -0.43 to 0.89 | 4.0 |  |
| Bassi *et al* 2021^38^ | 0.46 | 0.35 to 0.56 | 9.3 |  |
| Bekelman *et al* 2018^58^ | 0.28 | -0.15 to 0.71 | 5.8 |  |
| Brims *et al* 2019^51^ | 0.40 | -0.55 to 1.35 | 7.5 |  |
| do Carmo *et al* 2017^33^ | -0.35 | -0.95 to 0.24 | 4.2 |  |
| Edmonds *et al* 2010^60^ | -0.11 | -0.62 to 0.39 | 5.0 |  |
| El-Jahwari *et al* 2016^34^ | 0.30 | 0.03 to 0.58 | 7.5 |  |
| El-Jahwari *et al* 2021^35^ | 1.29 | 0.19 to 2.39 | 1.8 |  |
| Evans *et al* 2021^61^ | 0.35 | -0.27 to 0.96 | 4.0 |  |
| Eychmueller *et al* 2021^40^ | -0.28 | -0.67 to 0.12 | 6.1 |  |
| Gao *et al* 2020^63^ | 0.33 | -0.48 to 1.13 | 3.1 |  |
| Greer *et al* 2022^36^ | -0.75 | -1.92 to 0.42 | 1.6 |  |
| Hoek *et al* 2017^36^ | -1.80 | -4.09 to 0.48 | 0.5 |  |
| Kluger *et al* 2020^65^ | 0.35 | -0.28 to 0.96 | 4.1 |  |
| Rogers *et al* 2014^27^ | 0.76 | -0.75 to 2.26 | 1.0 |  |
| Sidebottom *et al* 2015^67^ | 0.14 | -0.14 to 0.43 | 7.5 |  |
| Slama *et al* 2020^68^ | 0.25 | -1.27 to 1.77 | 1.0 |  |
| Tattersall *et al* 2014^44^ | -0.51 | -0.76 to -0.25 | 7.9 |  |
| Temel *et al* 2020^69^ | 0.77 | -0.03 to 1.58 | 2.9 |  |
| Woo *et al* 2019^46^ | 0.01 | -0.10 to 0.13 | 9.2 |  |
|  |  |  |  |  |
| ***Meta-analysis*** | **SMD (MID)** | **95% CI** | ***t*** | ***p*** |
| Random effects model | 0.12 | -0.06 to 0.29 | 1.400 | 0.176 |
|  |  |  |  |  |
| ***Heterogeneity*** |  |  | ***Q (df)*** | ***p*** |
| *τ²* | 0.07 | 0.02 to 0.32 | 90.00 (21) | **<0.001** |
| *I²* | 76.7% | 65.0 to 84.5% |  |  |
| *H* | 2.07 | 1.69 to 2.54 |  |  |

**Forest plot**


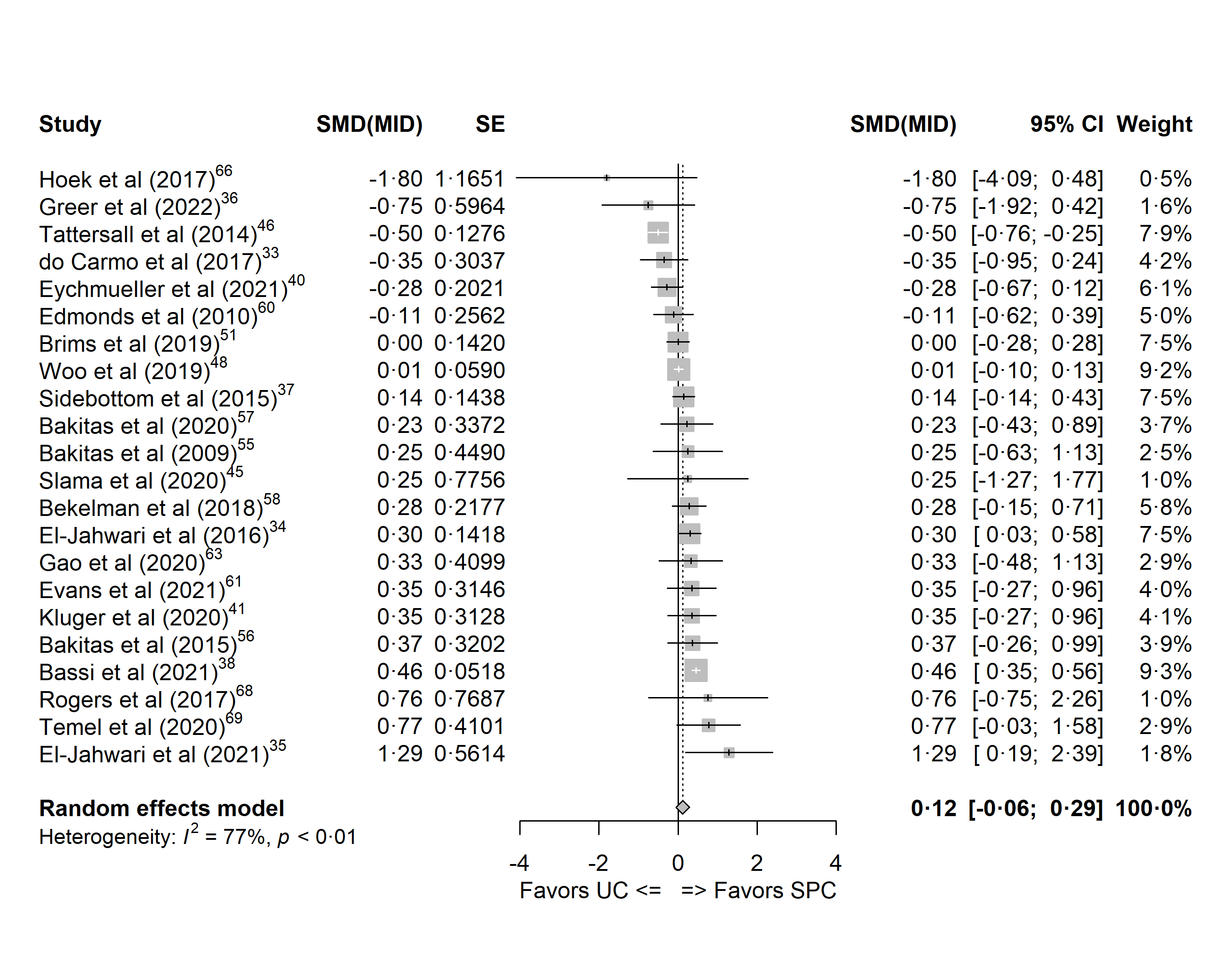


**Publication bias**

Egger’s enhanced funnel plot

| Linear regression test of funnel plot asymmetry  Intercept: -0.473  95% CI: -1.750 to 0.803  *t*(21) = -0.727, *p* = 0.476 | 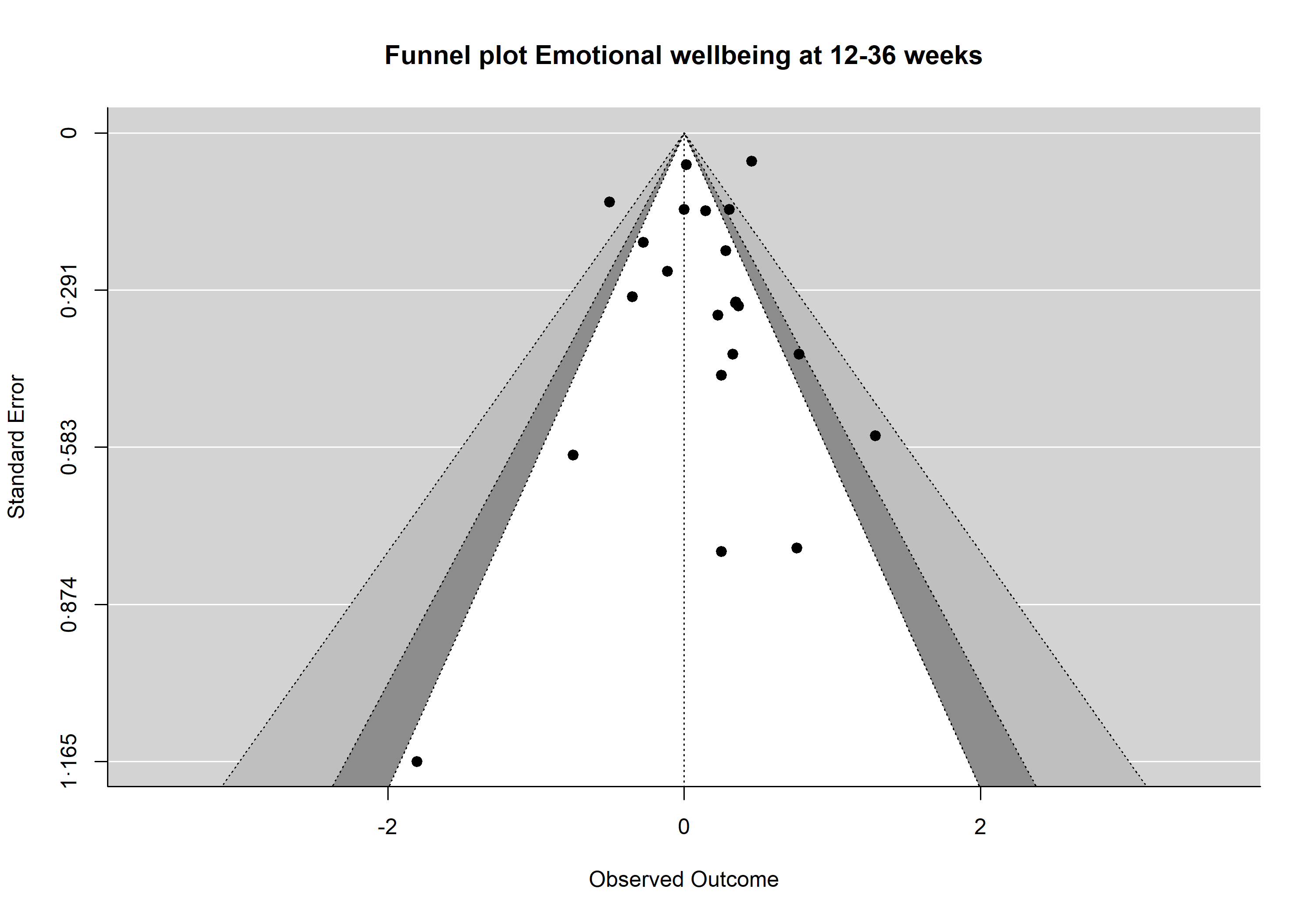 |
| --- | --- |

**Analysis with effect size RR (achieving change ≥1 MID)**

Number of studies combined: k = 22

Number of observations: 2728; Number of events: 910

| **Study (k = 22)** | **RR** | **95% CI** | **weight (random, in %)** |  |
| --- | --- | --- | --- | --- |
| Bakitas *et al* 2009^55^ | 1.09 | 0.74; 1.60 | 6.9 |  |
| Bakitas *et al* 2015^56^ | 1.18 | 0.86; 1.62 | 10.2 |  |
| Bakitas *et al* 2020^57^ | 1.08 | 0.75; 1.54 | 7.9 |  |
| Bassi *et al* 2021^38^ | 1.36 | 0.09; 19.89 | 0.1 |  |
| Bekelman *et al* 2018^58^ | 1.20 | 0.84; 1.70 | 8.1 |  |
| Brims *et al* 2019^51^ | 0.90 | 0.36; 2.25 | 1.2 |  |
| do Carmo *et al* 2017^33^ | 1.17 | 0.08; 16.72 | 0.1 |  |
| Edmonds *et al* 2010^60^ | 0.81 | 0.17; 5.16 | 0.3 |  |
| El-Jahwari *et al* 2016^34^ | 1.60 | 0.71; 3.64 | 1.5 |  |
| El-Jahwari *et al* 2021^35^ | 1.38 | 0.96; 1.99 | 7.6 |  |
| Evans *et al* 2021^61^ | 1.04 | 0.35; 3.13 | 0.8 |  |
| Eychmueller *et al* 2021^40^ | 0.88 | 0.39; 2.02 | 1.5 |  |
| Gao *et al* 2020^63^ | 1.11 | 0.86; 1.43 | 15.7 |  |
| Greer *et al* 2022^36^ | 0.72 | 0.45; 1.17 | 4.4 |  |
| Hoek *et al* 2017^36^ | 0.62 | 0.24; 1.59 | 1.1 |  |
| Kluger *et al* 2020^65^ | 1.14 | 0.82; 1.57 | 9.8 |  |
| Rogers *et al* 2014^27^ | 1.18 | 0.77; 1.80 | 5.7 |  |
| Sidebottom *et al* 2015^67^ | 1.26 | 0.79; 2.02 | 4.6 |  |
| Slama *et al* 2020^68^ | 1.10 | 0.67; 1.80 | 4.1 |  |
| Tattersall *et al* 2014^44^ | 0.27 | 0.03; 2.23 | 0.2 |  |
| Temel *et al* 2020^69^ | 1.34 | 0.93; 1.92 | 7.8 |  |
| Woo *et al* 2019^46^ | 1.00 | 0.06; 15.50 | 0.1 |  |
|  |  |  |  |  |
| ***Meta-analysis*** | **RR** | **95% CI** | ***t*** | ***p*** |
| Random effects model | 1.13 | 1.05 to 1.22 | 3.410 | **0.003** |
|  |  |  |  |  |
| ***Heterogeneity*** |  |  | ***Q (df)*** | ***p*** |
| *τ²* | 0.00 | 0.00 to 0.00 | 10.58 (21) | 0.970 |
| *I²* | 0.0% | 0.0 to 46.2% |  |  |
| *H* | 1.00 | 1.00 to 1.36 |  |  |

**Forest plot of RR effect size for the QoL outcome 12 to 36 weeks**


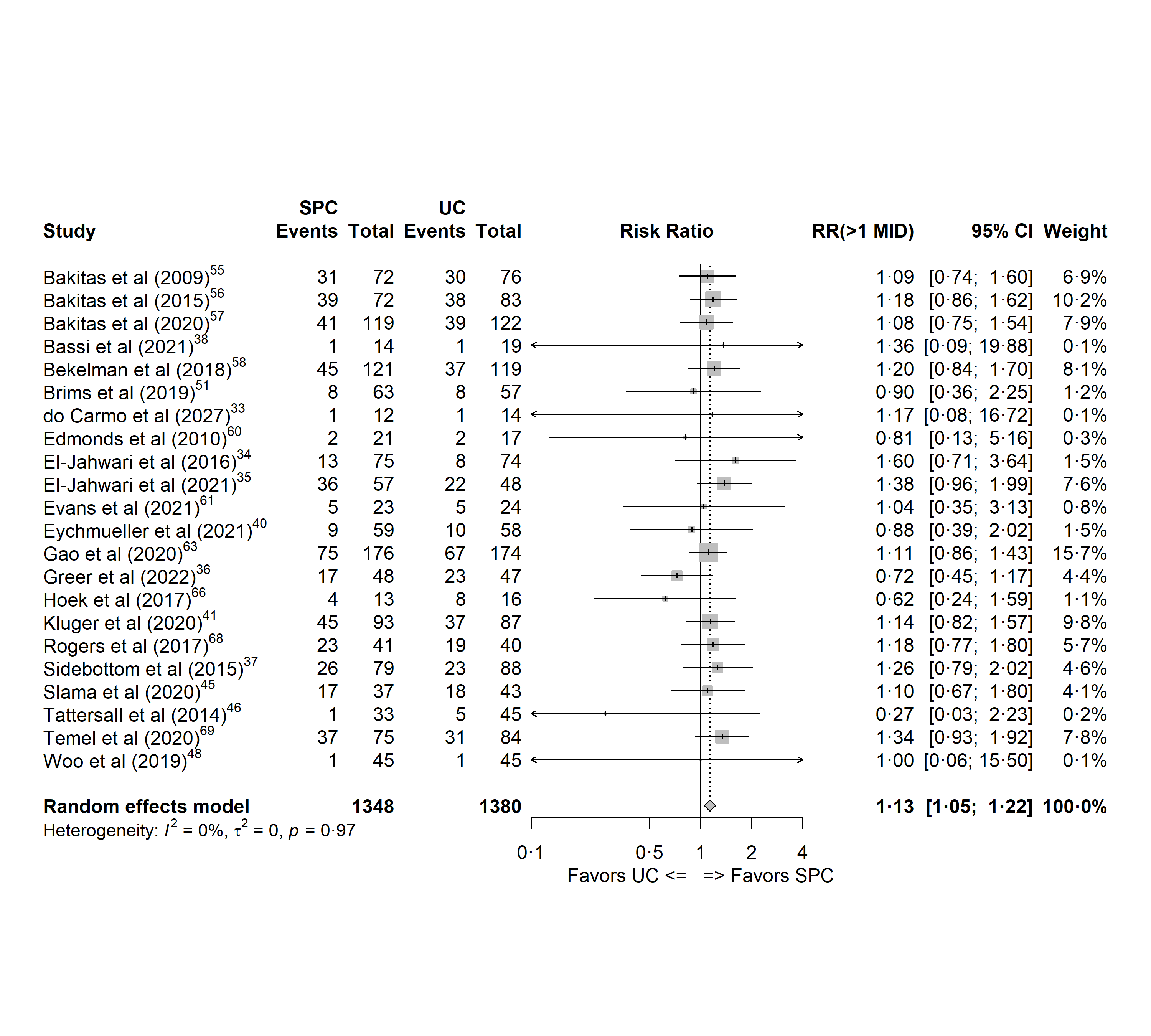


The risk ratio of RR = 1.13 (95% CI = 1.05 to 1.22) translates into a point estimate of a 13% increased probability of experiencing a change in QOL of at least 1 MID size with SPC. This is statistically significant.

The number needed to treat is calculated as follows (*p_CG_* is the baseline risk of experiencing change of at least 1 MID in the control group):

$$\frac{1}{p_{CG}\cdot(1-RR)}=\frac{1}{\frac{430}{1380}\cdot0.13}=24.687 \to25$$

The NNT is 25, meaning that 25 people need to be treated with SPC in order for one person to have a change in emotional wellbeing at 12 to 36 weeks of at least 1 MID.

**Meta-regression: Univariate meta-regression analyses with covariates**

| *k = 22* | **Regression** | | | | | **Heterogeneity** | | | **Test of moderators** | |
| --- | --- | --- | --- | --- | --- | --- | --- | --- | --- | --- |
|  | *b* | *SE* | *t* | *p* | 95% CI | *I²* | *Q* | *p* | *F* | *p* |
| ***Attrition (in %)*** |  |  |  |  |  |  |  |  |  |  |
| Intercept | 0.37 | 0.15 | 2.530 | **0.020** | - | 63 | 73.6 | **0.000** | 4.216 | 0.053 |
| Attrition (in %) | -0.01 | 0.01 | -2.053 | 0.053 | -0.02; -0.00 |  |  |  |  |  |
| ***% advanced disease*** |  |  |  |  |  |  |  |  |  |  |
| Intercept | 0.26 | 0.33 | 0.799 | 0.441 | - | 71 | 55.5 | **0.000** | 0.153 | 0.703 |
| % advanced disease | -0.00 | 0.00 | -0.392 | 0.703 | -0.01; 0.01 |  |  |  |  |  |
| ***Disease group (ref: Cancer)*** |  |  |  |  |  |  |  |  |  |  |
| Intercept | -0.00 | 0.10 | -0.081 | 0.936 | - | 57 | 46.9 | **0.001** | 3.266 | 0.086 |
| Non-cancer | 0.28 | 0.16 | 1.807 | 0.086 | -0.04; 0.61 |  |  |  |  |  |
| ***RoB2 score (ref: low risk)*** |  |  |  |  |  |  |  |  |  |  |
| Intercept | 0.09 | 0.10 | 0.984 | 0.337 | - | 67 | 76.9 | **0.000** | 0.441 | 0.650 |
| RoB2: Some risk | -0.16 | 0.20 | -0.843 | 0.410 | -0.57; 0.24 |  |  |  |  |  |
| RoB2: High risk | -0.06 | 0.15 | -0.435 | 0.669 | -0.37; 0.24 |  |  |  |  |  |
| ***Service composition score*** |  |  |  |  |  |  |  |  |  |  |
| Intercept | -0.68 | 0.21 | -3.234 | **0.004** | - | 28 | 30.4 | 0.063 | 15.370 | **0.001** |
| Service composition score | 0.07 | 0.02 | 3.920 | **0.001** | 0.03; 0.11 |  |  |  |  |  |
| ***Setting (ref: multiple settings)*** |  |  |  |  |  |  |  |  |  |  |
| Intercept | 0.26 | 0.15 | 1.756 | 0.095 | - | 72 | 89.6 | **0.000** | 0.843 | 0.446 |
| Inpatient consulting model | -0.13 | 0.23 | -0.562 | 0.580 | -0.62; 0.36 |  |  |  |  |  |
| Home or hospital outreach | -0.25 | 0.20 | -1.296 | 0.210 | -0.66; 0.16 |  |  |  |  |  |
| ***Type of intervention (ref: SPC)*** |  |  |  |  |  |  |  |  |  |  |
| Intercept | 0.09 | 0.13 | 0.686 | 0.502 | - | 37 | 30.9 | **0.029** | 4.771 | **0.013** |
| Early SPC | -0.22 | 0.16 | -1.346 | 0.195 | -0.57; 0.12 |  |  |  |  |  |
| Integrated collaborative care | 0.34 | 0.18 | 1.911 | 0.072 | -0.04; 0.71 |  |  |  |  |  |
| Nurse-led palliative care | 0.20 | 0.27 | 0.740 | 0.469 | -0.36; 0.76 |  |  |  |  |  |
| ***Year*** |  |  |  |  |  |  |  |  |  |  |
| Intercept | -0.44 | 0.45 | -0.993 | 0.333 | - | 67 | 61.1 | **0.000** | 1.612 | 0.219 |
| Year | 0.03 | 0.03 | 1.296 | 0.219 | -0.02; 0.08 |  |  |  |  |  |

.

**Bubble plots of univariate meta-regression analyses**

| **Attrition** | *F*(1,20) = 4.216  *p* = 0.053 | 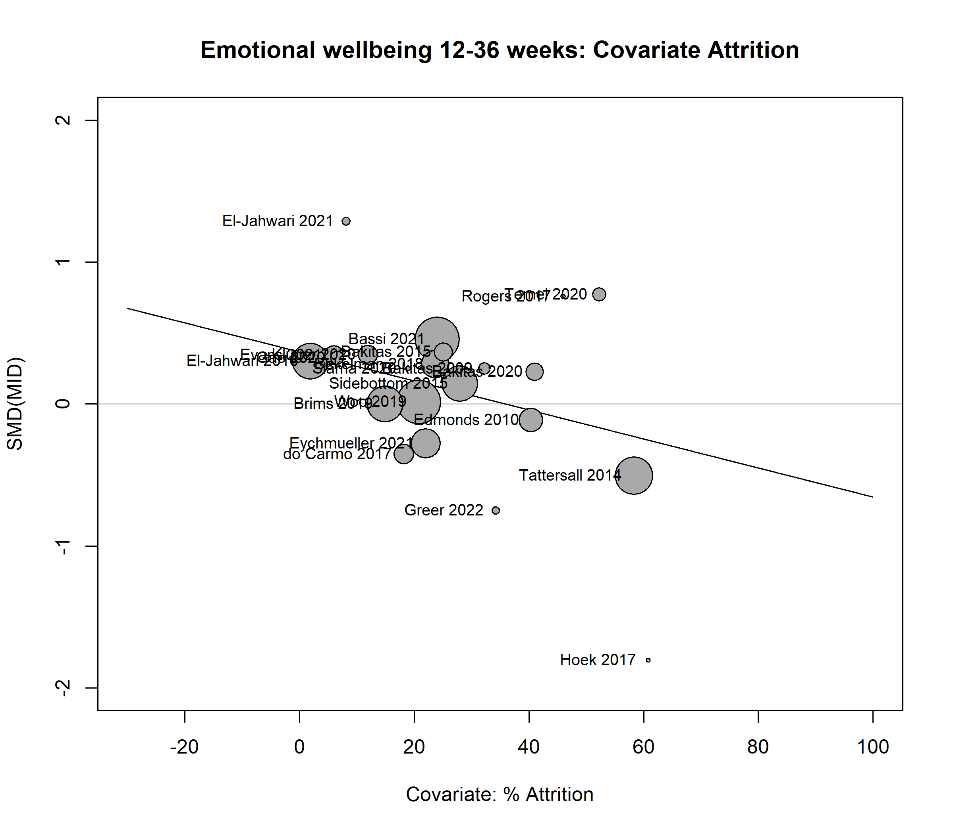 |
| --- | --- | --- |
| **% advanced disease** | *F*(1,11) = 0.153  *p* = 0.703 | 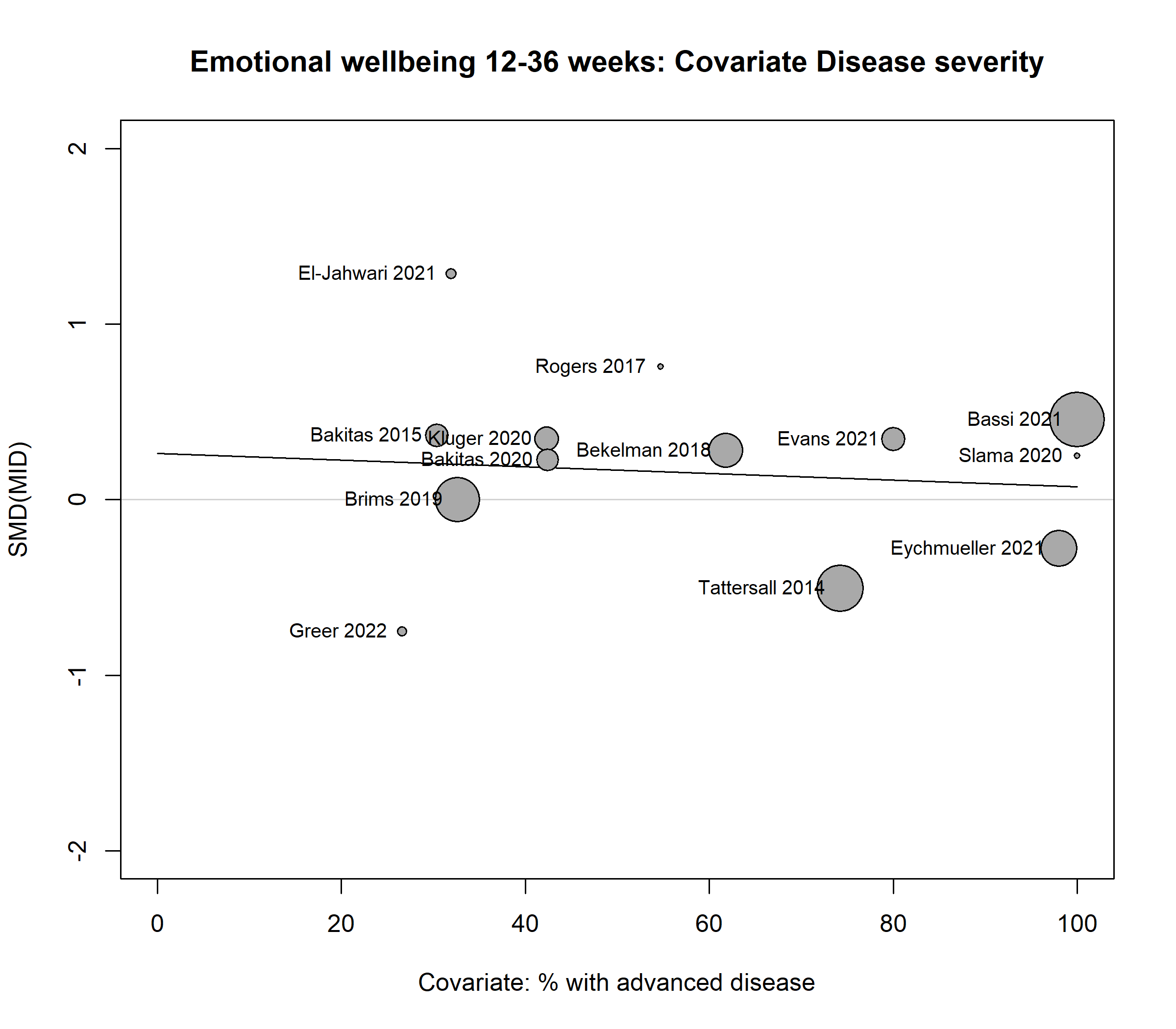 |
| **Disease group** | *F*(1,20) = 3.266  *p* = 0.086 | 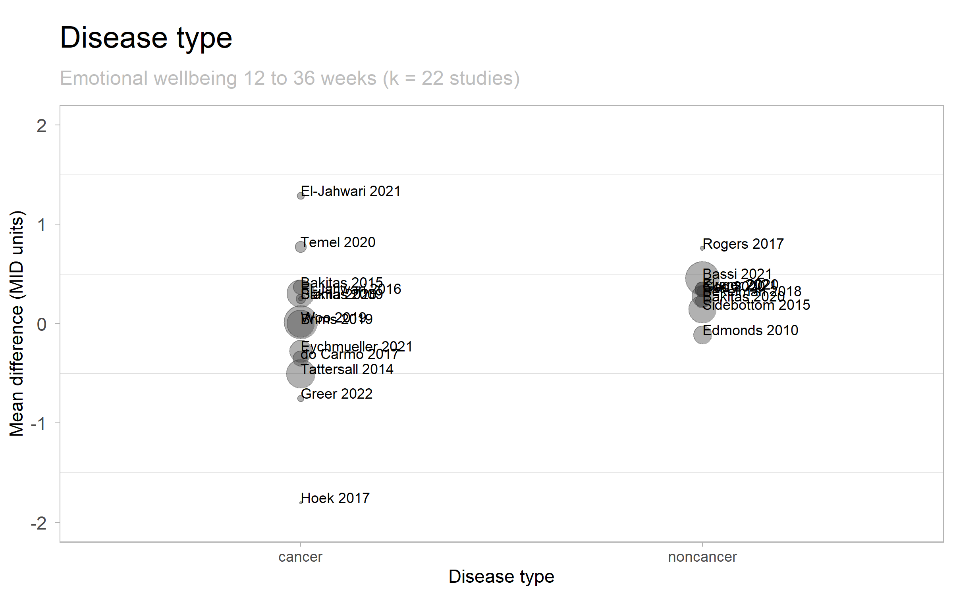 |
| **RoB2 score** | *F*(2,19) = 0.441  *p* = 0.650 | 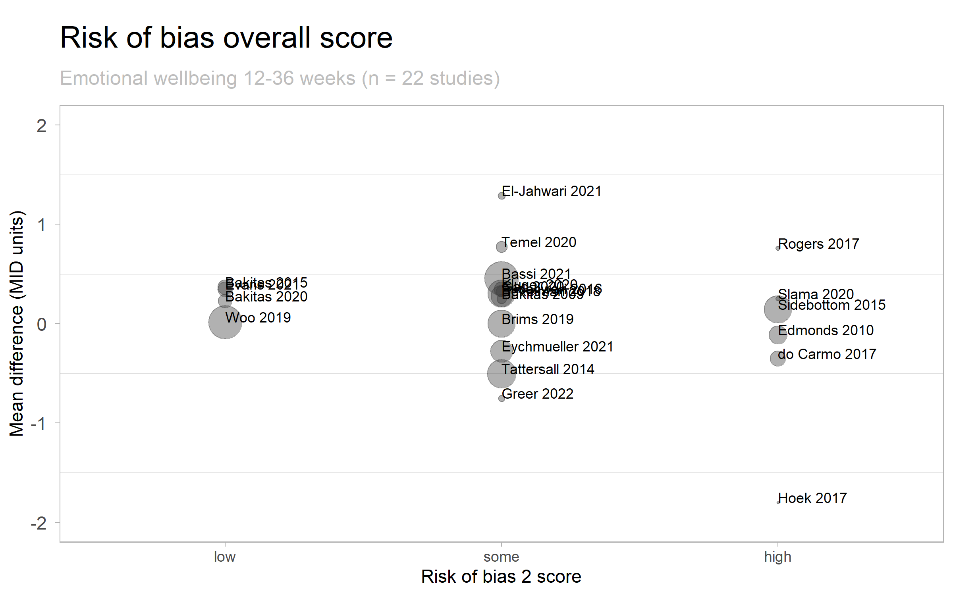 |
| **Service composition score** | *F*(1,20) = 15.370  *p* = **0.001** | 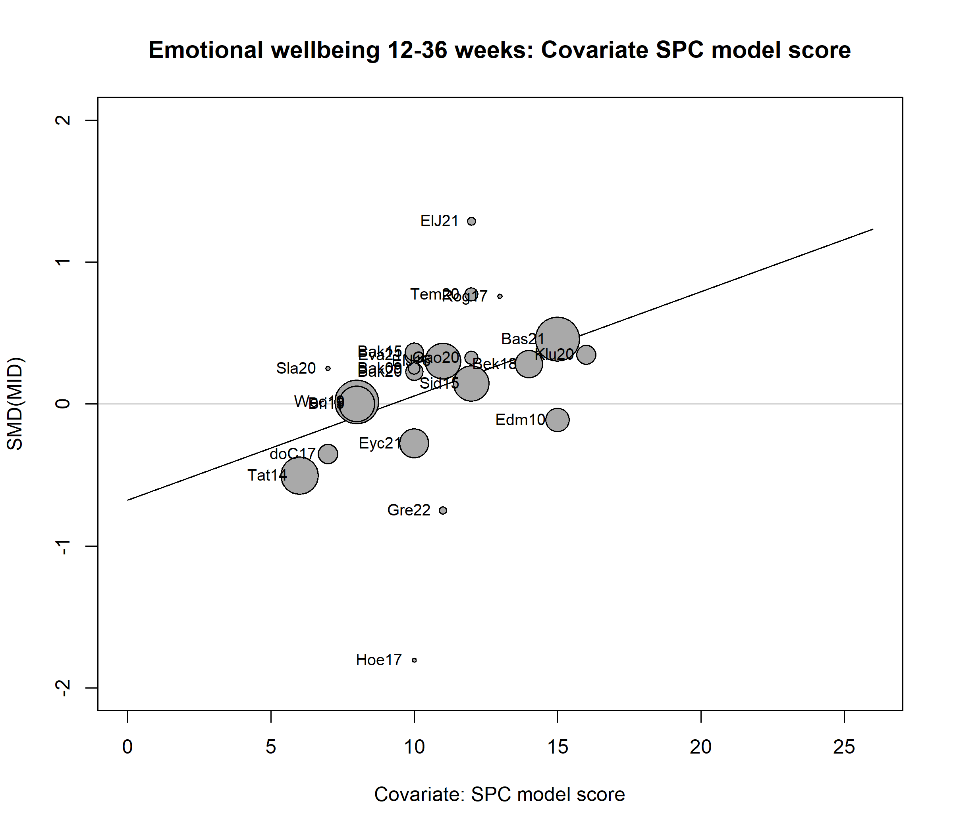 |
| **Setting** | *F*(2,19) = 0.843  *p* = 0.446 | 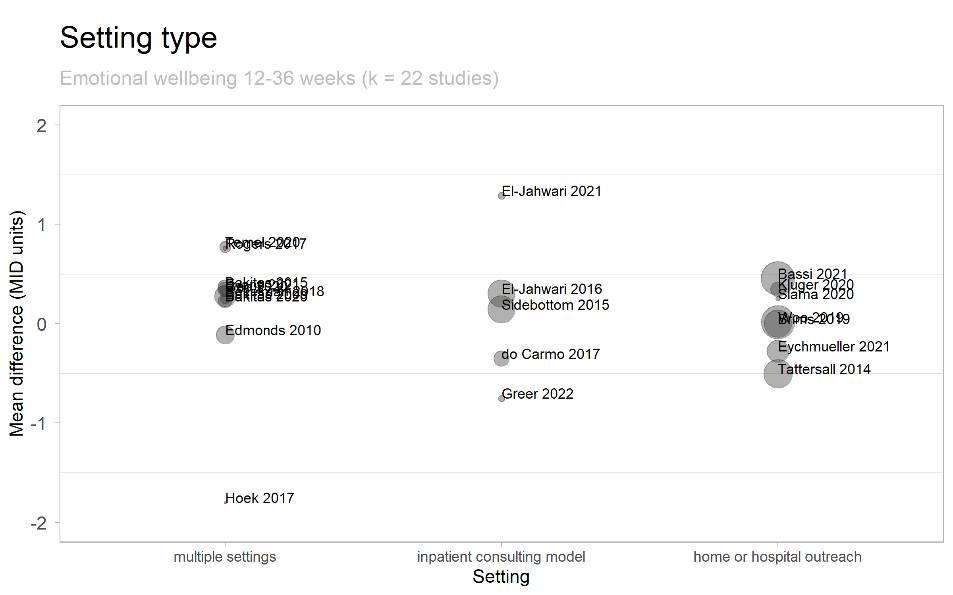 |
| **Type of intervention** | *F*(3,18) = 4.771  *p* = **0.013** | 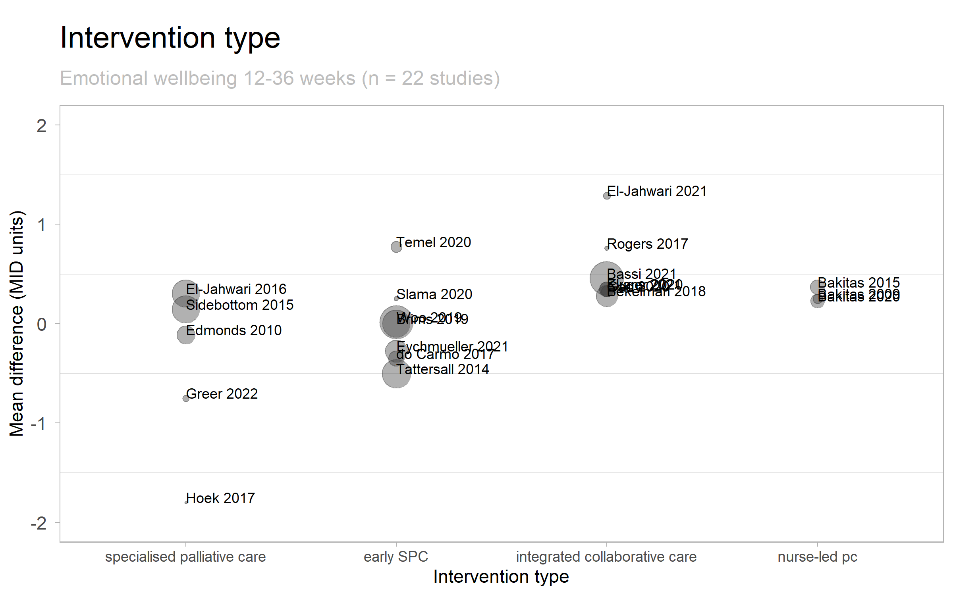 |
| **Year** | *F*(1,20) = 1.612  *p* = 0.219 | 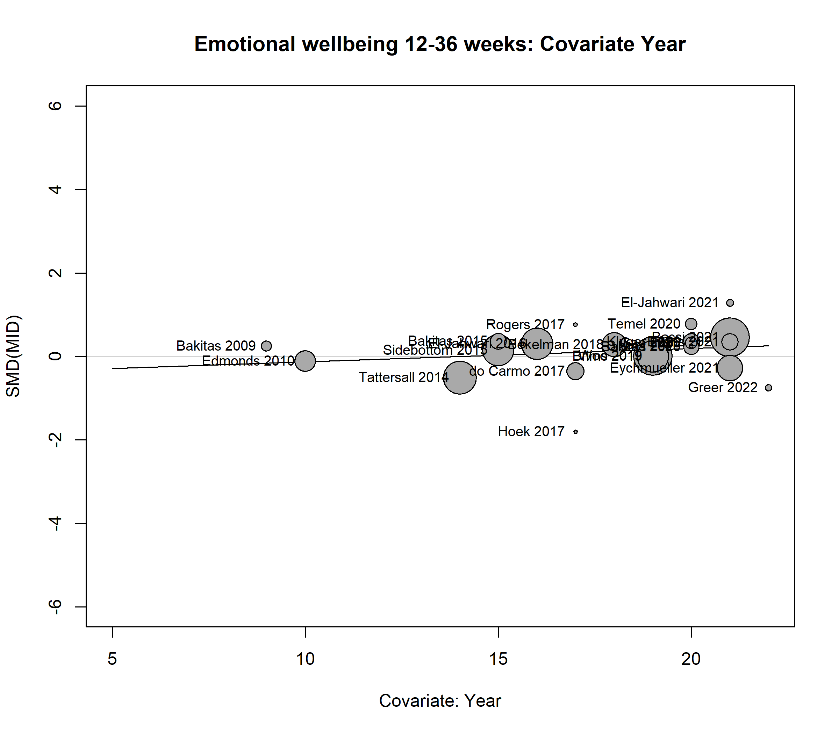 |
