# Supplementary material for: Benefits of specialist palliative care by identifying active ingredients of service composition, structure, and delivery model: A systematic review with meta-analysis and meta-regression
Source: PLoS Med. 2024 Aug 2;21(8):e1004436. doi: 10.1371/journal.pmed.1004436 (PMC11329153; doi:10.1371/journal.pmed.1004436)
Supplement: S11 Appendix — (DOCX) [file pmed.1004436.s011.docx]

**Benefits of specialist palliative care by identifying active ingredients of service composition, structure, and delivery model: A systematic review with meta-analysis and meta-regression**

**S11 Appendix**

Miriam J. Johnson, Leah Rutherford, Anisha Sunny, Sophie Pask, Susanne de Wolf-Linder, Fliss E. M. Murtagh, Christina Ramsenthaler

[hycr22@hyms.ac.uk](mailto:hycr22@hyms.ac.uk)

**PRISMA checklist**

# Table A: PRISMA Checklist

| **Section and Topic** | **Item #** | **Checklist item** | **Location where item is reported** |
| --- | --- | --- | --- |
| **TITLE** | | |  |
| Title | 1 | Identify the report as a systematic review. | Title specifies this as a meta-analysis |
| **ABSTRACT** | | |  |
| Abstract | 2 | See the PRISMA 2020 for Abstracts checklist. | Items included as per PLOSMed and PRISMA checklist guidelines |
| **INTRODUCTION** | | |  |
| Rationale | 3 | Describe the rationale for the review in the context of existing knowledge. | Rationale given and need for review motivated in introduction, paragraph 3. Extensive review of existing evidence and prior systematic reviews and meta-analyses in Supplement 1 and 2. |
| Objectives | 4 | Provide an explicit statement of the objective(s) or question(s) the review addresses. | Objectives are given at the end of the introduction section of the manuscript, paragraph 4. |
| **METHODS** | | |  |
| Eligibility criteria | 5 | Specify the inclusion and exclusion criteria for the review and how studies were grouped for the syntheses. | Please see Table 1. |
| Information sources | 6 | Specify all databases, registers, websites, organisations, reference lists and other sources searched or consulted to identify studies. Specify the date when each source was last searched or consulted. | Please see Table 1. Additional information is given on PROSPERO where this review was registered (no: CRD42021292371). Information on the searches and the full search strategy can be found in Supplement 3, an abbreviated search strategy and databases are listed in Table 1. |
| Search strategy | 7 | Present the full search strategies for all databases, registers and websites, including any filters and limits used. | Information on the searches and the full search strategy can be found in Supplement 3. An abbreviated search strategy and databases are listed in Table 1. |
| Selection process | 8 | Specify the methods used to decide whether a study met the inclusion criteria of the review, including how many reviewers screened each record and each report retrieved, whether they worked independently, and if applicable, details of automation tools used in the process. | Please see Table 1 and methods section, paragraphs and subsections in “Search strategy and selection criteria” and “Eligibility criteria and study selection”. |
| Data collection process | 9 | Specify the methods used to collect data from reports, including how many reviewers collected data from each report, whether they worked independently, any processes for obtaining or confirming data from study investigators, and if applicable, details of automation tools used in the process. | Please see methods section, paragraph “Data extraction and risk of bias assessment” and Table 1. |
| Data items | 10a | List and define all outcomes for which data were sought. Specify whether all results that were compatible with each outcome domain in each study were sought (e.g. for all measures, time points, analyses), and if not, the methods used to decide which results to collect. | This is detailed in the manuscript in the methods section “Data analysis”, first paragraph, and in Table 1 as well as in Supplement 4 (extended protocol for the analysis). |
|  | 10b | List and define all other variables for which data were sought (e.g. participant and intervention characteristics, funding sources). Describe any assumptions made about any missing or unclear information. | This is detailed in the manuscript in the methods section “Data analysis”, first paragraph, and in Table 1 as well as in Supplement 4 (extended protocol for the analysis). |
| Study risk of bias assessment | 11 | Specify the methods used to assess risk of bias in the included studies, including details of the tool(s) used, how many reviewers assessed each study and whether they worked independently, and if applicable, details of automation tools used in the process. | Please see paragraph “Data extraction and risk of bias assessment” in manuscript and Table 1. |
| Effect measures | 12 | Specify for each outcome the effect measure(s) (e.g. risk ratio, mean difference) used in the synthesis or presentation of results. | Please see paragraph “Data analysis” in manuscript and Supplement 4 (extended protocol for the analysis). |
| Synthesis methods | 13a | Describe the processes used to decide which studies were eligible for each synthesis (e.g. tabulating the study intervention characteristics and comparing against the planned groups for each synthesis (item #5)). | Please see the corresponding paragraphs in the manuscript “Data analysis” and the one before, “Classifications of components of specialist palliative care intervention models” and Supplement 4 (extended protocol for the analysis).  A full list of excluded full-texts with reasons for exclusion is available in Supplement 6.  Full data extraction tables are available in Supplement 7. |
|  | 13b | Describe any methods required to prepare the data for presentation or synthesis, such as handling of missing summary statistics, or data conversions. |  |
|  | 13c | Describe any methods used to tabulate or visually display results of individual studies and syntheses. |  |
|  | 13d | Describe any methods used to synthesize results and provide a rationale for the choice(s). If meta-analysis was performed, describe the model(s), method(s) to identify the presence and extent of statistical heterogeneity, and software package(s) used. |  |
|  | 13e | Describe any methods used to explore possible causes of heterogeneity among study results (e.g. subgroup analysis, meta-regression). |  |
|  | 13f | Describe any sensitivity analyses conducted to assess robustness of the synthesized results. |  |
| Reporting bias assessment | 14 | Describe any methods used to assess risk of bias due to missing results in a synthesis (arising from reporting biases). | Please see the appropriate paragraph “Data extraction and risk of bias assessment”. |
| Certainty assessment | 15 | Describe any methods used to assess certainty (or confidence) in the body of evidence for an outcome. | Please see the appropriate paragraph “Data extraction and risk of bias assessment”. |
| **RESULTS** | | |  |
| Study selection | 16a | Describe the results of the search and selection process, from the number of records identified in the search to the number of studies included in the review, ideally using a flow diagram. | A full PRISMA flowchart is provided as Figure 1 in the manuscript. |
|  | 16b | Cite studies that might appear to meet the inclusion criteria, but which were excluded, and explain why they were excluded. | A list of excluded fulltexts with reasons for exclusion is available in Supplement 6. |
| Study characteristics | 17 | Cite each included study and present its characteristics. | Full study characteristics are presented in the first to fourth paragraph of the Results section and in Supplement 7. |
| Risk of bias in studies | 18 | Present assessments of risk of bias for each included study. | Please see Supplement 8 for GRADE table and risk of bias table. |
| Results of individual studies | 19 | For all outcomes, present, for each study: (a) summary statistics for each group (where appropriate) and (b) an effect estimate and its precision (e.g. confidence/credible interval), ideally using structured tables or plots. | For the main endpoint, this is provided as Figure 2a and 2b for the two outcomes (a) quality of life and (b) emotional wellbeing. A full set of all analyses with all effect sizes for all endpoints is provided in Supplement 9. |
| Results of syntheses | 20a | For each synthesis, briefly summarise the characteristics and risk of bias among contributing studies. | Please see risk of bias in results section, paragraph four, and in Supplement 8. |
|  | 20b | Present results of all statistical syntheses conducted. If meta-analysis was done, present for each the summary estimate and its precision (e.g. confidence/credible interval) and measures of statistical heterogeneity. If comparing groups, describe the direction of the effect. | For the main endpoint, this is provided as Figure 2a and 2b for the two outcomes (a) quality of life and (b) emotional wellbeing. A full set of all analyses with all effect sizes for all endpoints is provided in Supplement 9. |
|  | 20c | Present results of all investigations of possible causes of heterogeneity among study results. | For the main endpoint, this is provided as Figure 2a and 2b for the two outcomes (a) quality of life and (b) emotional wellbeing. A full set of all analyses with all effect sizes for all endpoints is provided in Supplement 9.  Results of all meta-regressions to investigate heterogeneity are presented in Supplement 9. |
|  | 20d | Present results of all sensitivity analyses conducted to assess the robustness of the synthesized results. | Sensitivity analysis are included as findings in the results section (paragraph 5 onwards) and are presented in full in Supplement 10. |
| Reporting biases | 21 | Present assessments of risk of bias due to missing results (arising from reporting biases) for each synthesis assessed. | Please see Supplement 8, eFigure 1 for details. |
| Certainty of evidence | 22 | Present assessments of certainty (or confidence) in the body of evidence for each outcome assessed. | Please see Supplement 8, eFigure 1 for details. |
| **DISCUSSION** | | |  |
| Discussion | 23a | Provide a general interpretation of the results in the context of other evidence. | Please see discussion section, first and following paragraph. |
|  | 23b | Discuss any limitations of the evidence included in the review. | Please see discussion section, sixth and seventh paragraphs. |
|  | 23c | Discuss any limitations of the review processes used. | Please see discussion section, sixth and seventh paragraphs. |
|  | 23d | Discuss implications of the results for practice, policy, and future research. | Please see discussion section, 9^th^ and 10th paragraph. |
| **OTHER INFORMATION** | | |  |
| Registration and protocol | 24a | Provide registration information for the review, including register name and registration number, or state that the review was not registered. | Registered on PROSPERO: CRD42021292371 |
|  | 24b | Indicate where the review protocol can be accessed, or state that a protocol was not prepared. | On PROSPERO, detailed analysis protocol in Supplement 4. |
|  | 24c | Describe and explain any amendments to information provided at registration or in the protocol. | No amendments. |
| Support | 25 | Describe sources of financial or non-financial support for the review, and the role of the funders or sponsors in the review. | Manuscript, Declarations. |
| Competing interests | 26 | Declare any competing interests of review authors. | No competing interests, please see section on declarations. |
| Availability of data, code and other materials | 27 | Report which of the following are publicly available and where they can be found: template data collection forms; data extracted from included studies; data used for all analyses; analytic code; any other materials used in the review. | All available on <https://osf.io/h8pmz/>  Please see section on declarations in the manuscript. |

*From:*  Page MJ, McKenzie JE, Bossuyt PM, Boutron I, Hoffmann TC, Mulrow CD, et al. The PRISMA 2020 statement: an updated guideline for reporting systematic reviews. BMJ 2021;372:n71. doi: 10.1136/bmj.n71

For more information, visit: <http://www.prisma-statement.org/>
