# Supplementary material for: Benefits of specialist palliative care by identifying active ingredients of service composition, structure, and delivery model: A systematic review with meta-analysis and meta-regression
Source: PLoS Med. 2024 Aug 2;21(8):e1004436. doi: 10.1371/journal.pmed.1004436 (PMC11329153; doi:10.1371/journal.pmed.1004436)
Supplement: Attachment — Submitted filename: Replyletter_SPCMetaanalysis_PLOSMed_R1 mj.docx [file pmed.1004436.s012.docx]

Editorial team and external peer reviewers

Dr Alexandra Schaefer, PhD

PLOS Medicine

May 1^st^, 2024

Dear Dr Schaefer,

Dear Reviewers,

Re: **PMEDICINE-D-24-00814R1 “Does specialist palliative care provide additional benefits? A meta-analysis with meta-regression to identify active ingredients of service composition, service structure, and delivery model”**

Many thanks for your time in reviewing the above manuscript, and for your comments which have helped us to hopefully improve the manuscript. We enclose the revised manuscript for your consideration, and outline below how we have addressed each point.

*ACADEMIC EDITOR COMMENTS
I agree that it's an interesting approach to an important topic. The areas that I don't think are covered are the context of who is dying where around the world of what, and then contextualizing the results in the discussion with that in mind. It's possible that the trials have been done effectively in populations where there's almost a pre-selection of potential to benefit from palliative care services - and that's where it's important to look at settings, age groups, conditions that people are dying from. The countries and regions of the world are not provided, and it seems likely that most of this evidence is from high-income countries with a handful represented, even before thinking about the different settings in which people die. Caution must therefore be exercised in interpretation and generalization.*

**Reply:**

Thank you for raising this issue. We agree that we had omitted information on country of origin of included studies. We have amended Supplemental File S7, eTable 7.1, now presenting countries in which the included studies were done. We have also added this information to the description of included studies in the results section on page 12, line 236 ff. In terms of countries, 16 RCTs were done in the USA, 4 in Italy, 3 in Denmark or the United Kingdom, 2 each in Australia or China, and 1 each in Belgium, Brazil, Canada, the Czech Republic, India, South Korea, Sweden, Switzerland, and The Netherlands. Overall, this confirms the suspicion that the majority of trials was done in countries with a well-resourced health care system in which specialist palliative care is part of mainstream healthcare. However, with a few studies from countries like Brazil, South Korea, and China, there are also trials included from countries in which specialist palliative care has only recently been established or is yet to be established as part of the healthcare system. In terms of age groups and diseases, we present this information in Supplemental File S7, also presenting information (if reported) on the proportion of non-white participants. We describe the included studies’ sample composition in the results section, line 256 ff and alert to the fact that the proportion of older participants and the proportion of non-cancer conditions is quite high. We added the limitation of generalizability to low-/ middle-income countries and the possible underrepresentation of disadvantaged and underserved groups as a limitation in the discussion section (line 361 ff).

EDITORIAL COMMENTS
*The Editorial team agrees that your study addresses an important topic, but we feel that the study could be more specific about how the study (perhaps including the study design) adds to existing knowledge and, for example, how it highlights a gap in current service provision. Please consider our input as you revise your manuscript.*

**Reply:**

Thank you for your comment. To also answer the reviewer #3’s comments, we have strengthened the introduction section, particularly focusing on the gap in the literature and how our approach to meta-analysis will help close this gap. We have likewise tried to strengthen the discussion section by making the clinical message clearer. Please see the highlighted changes in both the introduction and discussion section.

Requests from the editors:
 ****Please note: not all will apply to your paper, but please check each item carefully

GENERAL COMMENTS

1) Please cite the reference numbers in square brackets. Citations should be preceding punctuation.*

**Reply**:

We have cited the reference numbers in square brackets and made sure that citations precede punctuation marks.

*COMPETING INTEREST

All authors must declare their relevant competing interests per the PLOS policy, which can be seen here:* [*https://eur03.safelinks.protection.outlook.com/?url=https%3A%2F%2Fjournals.plos.org%2Fplosmedicine%2Fs%2Fcompeting-interests&data=05%7C02%7Cchristina.ramsenthaler%40kcl.ac.uk%7C734b8fd20b5e477572ce08dc5b03cff0%7C8370cf1416f34c16b83c724071654356%7C0%7C0%7C638485319131093410%7CUnknown%7CTWFpbGZsb3d8eyJWIjoiMC4wLjAwMDAiLCJQIjoiV2luMzIiLCJBTiI6Ik1haWwiLCJXVCI6Mn0%3D%7C0%7C%7C%7C&sdata=rJ2JgNQPugx6%2FO%2BYIrMaBkocut7bgSliJC41VP%2BR%2FIQ%3D&reserved=0*](https://journals.plos.org/plosmedicine/s/competing-interests) *For authors with ties to industry, please indicate whether any of the interests has a financial stake in the results of the current study.*

**Reply:**

We have rephrased the competing interests statement following the guidance (line 413 ff).

*ABSTRACT

1) Please report your abstract according to PRISMA for Abstracts, following the PLOS Medicine abstract structure (Background, Methods and Findings, Conclusions).*

**Reply**:

We believe that the submitted abstract has already been presented in this format. We formatted the abstract so that it satisfies the journal’s and PRISMA guidelines.

*2) PLOS Medicine requests that main results are quantified with 95% CIs as well as p values. When reporting p values please report as p<0.001 and where higher as the exact p value p=0.002, for example. For the purposes of transparent data reporting, if not including the aforementioned please clearly state the reasons why not. When a p value is given, please specify the statistical test used to determine it.*

**Reply**:

We believe that the submitted manuscript already used the correct format as specified. However, we have revised the formatting in the abstract to comply with guidelines. All p values in the meta-analysis originate from the random-effects models as detailed in the data analysis section (line 201 ff).

*3) Throughout, suggest reporting statistical information as follows to improve clarity for the reader “22% (95% CI [13%,28%]; p</=)”. Please be sure to define all numerical values at first use. Please amend throughout the abstract and main manuscript. Please note the use of commas to separate upper and lower bounds, as opposed to hyphens as these can be confused with reporting of negative values.*

**Reply:**

We have used this format and revised how we present the confidence intervals in the abstract and main body of text.

*4) Please ensure that all numbers presented in the abstract are present and identical to numbers presented in the main manuscript text.*

**Reply:**

We have checked our manuscript and made sure that numbers align.

*5) Please provide the dates of search, data sources, number of studies included, types of study designs included, eligibility criteria, and synthesis/appraisal methods.*

**Reply:**

We have detailed these methods in the methods section and in Table 1.

*6) Please define all abbreviations including those for statistical reporting at first use.*

**Reply:**

We have made sure to define all abbreviations in the text at first use. We have also added a list of abbreviations at the end of the manuscript. We have checked that all abbreviations used in tables and figures are explained as well.

*7) In the last sentence of the Abstract Methods and Findings section, please describe the main limitation(s) of the study's methodology.*

**Reply:**

We have added a sentence in the abstract to this effect (line 58 f).

*AUTHOR SUMMARY
At this stage, we ask that you include a short, non-technical Author Summary of your research to make findings accessible to a wide audience that includes both scientists and non-scientists. The Author Summary should immediately follow the Abstract in your revised manuscript. This text is subject to editorial change and should be distinct from the scientific abstract. Ideally each sub-heading should contain 2-3 single sentence, concise bullet points containing the most salient points from your study. In the final bullet point of ‘What Do These Findings Mean?’, please include the main limitations of the study in non-technical language. Please see our author guidelines for more information:* [*https://eur03.safelinks.protection.outlook.com/?url=https%3A%2F%2Fjournals.plos.org%2Fplosmedicine%2Fs%2Frevising-your-manuscript%23loc-author-summary&data=05%7C02%7Cchristina.ramsenthaler%40kcl.ac.uk%7C734b8fd20b5e477572ce08dc5b03cff0%7C8370cf1416f34c16b83c724071654356%7C0%7C0%7C638485319131101377%7CUnknown%7CTWFpbGZsb3d8eyJWIjoiMC4wLjAwMDAiLCJQIjoiV2luMzIiLCJBTiI6Ik1haWwiLCJXVCI6Mn0%3D%7C0%7C%7C%7C&sdata=JNi%2FNyVWKRkew456UrZ2VLLFOJa0ZKw2hMHIMI9tMgA%3D&reserved=0*](https://journals.plos.org/plosmedicine/s/revising-your-manuscript#loc-author-summary)

**Reply**:

We have added the author summary in lay language (line 74 ff).

*INTRODUCTION
Please address past research and explain the need for and potential importance of your study. Indicate whether your study is novel and how you determined that. If there has been a systematic review of the evidence related to your study (or you have conducted one), please refer to and reference that review and indicate whether it supports the need for your study.*

**Reply**:

We have revised the introduction section, also trying to address the reviewers’ comments, and have strengthened the section on why we think our meta-analysis is addressing the current gap in the literature. We have also included a wider review of the evidence in the introduction section.

*METHODS AND RESULTS
1) PLOS Medicine requests that main results are quantified with 95% CIs as well as p values. We suggest reporting statistical information as detailed above – see under ABSTRACT.*

**Reply:**

We have quantified all results with 95% confidence intervals and p values throughout the abstract and main body of text.

*2) Please present numerators and denominators for percentages (at least in the Tables [not necessarily each time they're mentioned]).*

**Reply**:

This comment applies to some results and to data in figures. In figures, numerators and denominators for calculating relative risks are given throughout. When presenting percentages in the results, we also present numerators and denominators as well. Descriptive data regarding sample characteristics of included studies is also present in Supplemental File S7.

*3) Please report your study according to the PRISMA guidelines provided at the EQUATOR site. Please add the following statement, or similar, to the Methods: "This study is reported as per the Preferred Reporting Items for Systematic Reviews and Meta-Analyses (PRISMA) guideline (S1 Checklist)."*

**Reply:**

We have revised the sentence at the beginning of the methods section (line 167 f).

*4) Please provide the completed PRISMA checklist as Supporting Information. When completing the checklist, please use section and paragraph numbers, rather than page numbers.*

**Reply:**

We have added the completed PRISMA checklist as Supplemental File S10.

*DISCUSSION
Please present and organize the Discussion as follows: a short, clear summary of the article's findings; what the study adds to existing research and where and why the results may differ from previous research; strengths and limitations of the study; implications and next steps for research, clinical practice, and/or public policy; one-paragraph conclusion (no subheading).*

**Reply:**

We had originally laid out the discussion as such. However, we have added subheadings (apart from the conclusion) and edited slightly to make sure we have structured the text in a clearer way (also answering reviewer #3’s comment).

*FIGURES AND TABLES
1) Please provide titles and legends for all figures and tables (including those in Supporting Information files).*

**Reply:**

We have added all titles and legends to the figures and tables in the main body of text. We did compile all supplemental files into one appendix files which has a table of contents listing all information and tables.

*2) Please define all abbreviations used in each figure/table (including those in Supporting Information files).*

**Reply:**

We have taken care to do so. We also have included a complete list of abbreviations at the end of the manuscript.

*3) Please consider avoiding the use of red and green in order to make your figure more accessible to those with color blindness.*

**Reply**:

All our figures, also those in the appendix, are in black/white or in grey scale. The GRADE table showing the scorings of individual papers, however, uses green and red color. This is because we used the Cochrane library template and cannot change these colours (Supplement S8).

*SUPPLEMENTARY MATERIAL
1) For supplementary figures and tables, please see the general comments under TABLES and FIGURES and amend accordingly.*

**Reply:**

We have amended as mandated.

*2) We suggest reporting statistical information as detailed above – see under ABSTRACT. Please be sure to define all numerical values.*

**Reply**:

We have reported as mandated.

*3) As for the main manuscript, please indicate whether analyses are adjusted to help facilitate transparent data reporting please also detail the factors adjusted for and present the unadjusted analyses for comparison. If not, please clearly state the reasons why not.*

**Reply**:

We believe that this statement does not apply to our type of analysis.

*4) Please cite your Supporting Information as outlined here:* [*https://eur03.safelinks.protection.outlook.com/?url=https%3A%2F%2Fjournals.plos.org%2Fplosmedicine%2Fs%2Fsupporting-information&data=05%7C02%7Cchristina.ramsenthaler%40kcl.ac.uk%7C734b8fd20b5e477572ce08dc5b03cff0%7C8370cf1416f34c16b83c724071654356%7C0%7C0%7C638485319131107955%7CUnknown%7CTWFpbGZsb3d8eyJWIjoiMC4wLjAwMDAiLCJQIjoiV2luMzIiLCJBTiI6Ik1haWwiLCJXVCI6Mn0%3D%7C0%7C%7C%7C&sdata=xUtJVK%2FzykCG%2BH3lRryLXx00U3Mvc7LwyJhy%2FLP64ds%3D&reserved=0*](https://journals.plos.org/plosmedicine/s/supporting-information)

**Reply**:

We have amended the citation of supporting information in the manuscript as outlined in the guidance.

*REFERENCES
1) PLOS uses the numbered citation (citation-sequence) method and first six authors, et al.*

*2) Please ensure that journal name abbreviations match those found in the National Center for Biotechnology Information (NCBI) databases (*[*https://eur03.safelinks.protection.outlook.com/?url=http%3A%2F%2Fwww.ncbi.nlm.nih.gov%2Fnlmcatalog%2Fjournals&data=05%7C02%7Cchristina.ramsenthaler%40kcl.ac.uk%7C734b8fd20b5e477572ce08dc5b03cff0%7C8370cf1416f34c16b83c724071654356%7C0%7C0%7C638485319131114853%7CUnknown%7CTWFpbGZsb3d8eyJWIjoiMC4wLjAwMDAiLCJQIjoiV2luMzIiLCJBTiI6Ik1haWwiLCJXVCI6Mn0%3D%7C0%7C%7C%7C&sdata=nZz6iOrCBzVnMUI8RaVGcDxDwWfD5Oglx1YQIWTEs%2Bw%3D&reserved=0*](http://www.ncbi.nlm.nih.gov/nlmcatalog/journals)*), and are appropriately formatted and capitalised.*

*3) Where website addresses are cited, please specify the date of access (e.g. [accessed: 16/09/2023]).*

*4) Please also see* [*https://eur03.safelinks.protection.outlook.com/?url=https%3A%2F%2Fjournals.plos.org%2Fplosmedicine%2Fs%2Fsubmission-guidelines%23loc-references&data=05%7C02%7Cchristina.ramsenthaler%40kcl.ac.uk%7C734b8fd20b5e477572ce08dc5b03cff0%7C8370cf1416f34c16b83c724071654356%7C0%7C0%7C638485319131120389%7CUnknown%7CTWFpbGZsb3d8eyJWIjoiMC4wLjAwMDAiLCJQIjoiV2luMzIiLCJBTiI6Ik1haWwiLCJXVCI6Mn0%3D%7C0%7C%7C%7C&sdata=%2BrQ70giU7GGd%2BvUclHgVeHHkTE0OMMoB9NFrb5DbfvE%3D&reserved=0*](https://journals.plos.org/plosmedicine/s/submission-guidelines#loc-references) *for further details on reference formatting.*

**Reply:**

We have amended the citation methods in the manuscript to fit the journal’s style.

Comments from the reviewers:

*Reviewer #1: This is a novel approach to addressing the challenges of doing meta-analyses of widely heterogenous studies and is important to do in order to demonstrate the benefits of early integrated specialist palliative care, with multidisciplinary components. The results are clinically meaningful and relevant in these times of fiscal scarcity and fierce competition for resources.
My review is hampered by my lack of statistical expertise and I would recommend that it be thoroughly reviewed by someone with such expertise before accepting my recommendation.
From the clinical perspective and being knowledgeable about the topic I think it is well-written, makes sense, and I think important to be published.
I have no suggestions for improvement from a grammatical or language perspective.*

**Reply**:

Thank you for your comment. We really appreciate the positive feedback and confirmation of our main message.

*Reviewer #2: Firstly, I would like to thank the authors for their work in putting this manuscript together. I appreciate the enormity of the task that has been undertaken. The authors aimed to assess the impact of special palliative care on patient reported outcomes of quality of life and emotional wellbeing. I have mainly focused on the statistical analysis. Below are some queries:*

**Reply**:

Thank you for your appreciative comment and for acknowledging the size of the review.

*Methods: The main text doesn't appear to mention distribution based methods at all, only anchor based (page 8, line 124).*

**Reply**:

Thank you for noticing this omission. We have added the use of distribution-based methods in the section on line 205.

*Search date restriction: Could the authors please provide reasoning for this restriction and the impact it may have had on the identification of other studies of relevance before this date.*

**Reply**:

We agree that a clarification of the rationale for this decision is helpful. There are quite a few older systematic reviews that have also failed to synthesize effects of earlier studies, failing to do so because of poor conduct of these studies. One of our inclusion criteria was for trials to have an a priori sample size calculation on the primary endpoint and we have also found that most studies before the 2000s do not satisfy this criterion.

Service models of specialist palliative care have become part of mainstream healthcare only in recent years. For most countries in Europe and even countries like the United States, service models and their active ingredients have been developed from the 2000s onwards. We have added the following explanation to the text: “We restricted the search to synthesize effects for contemporary service models”. We believe that the restriction is more a strength than a limitation since including earlier evidence might jeopardize the generalizability and validity of extrapolating effects from early, pioneer studies to a situation where most specialist palliative care services are part of mainstream health care.

*Study selection: Information on how studies were screened is lacking information, e.g. what software was used. I would also suggest including study selection in the header "eligibility" as this will aid readability of the manuscript.*

**Reply**:

Thank you for noticing this omission. We have added more detail to this section as requested and have also added the software (Rayyan) that was used for screening (line 179 ff).

*DSL method is used and given that the majority of analyses include 20+ studies, this is probably OK. However, I would recommend the authors use a Hartung-Knapp correction to assess the impact on the analyses. Additionally, as some analyses include <20 studies, I feel this correction should be applied in those analyses. I would recommend a sensitivity analysis conducting both methods and using the most conservative of the two methods (i.e., the method with the widest confidence intervals) for each analysis.*

**Reply**:

Thank you for this suggestion for a sensitivity analysis. Having used both the meta and metafor packages in R, we had already run all analyses using the Hartung-Knapp correction but failed to detail this in the methods (or rather gave the wrong approach). We have therefore corrected the random-effects model (line 219).

*Forest plots: where there is heterogeneity, please include prediction intervals.*

**Reply:**

Thank you for your suggestion. Where there was heterogeneity, we have added this to the forest plots.

*Anchor based MID: What were the thresholds considered to qualify the MID? Did the authors consider the correlation between the anchor and the outcome? These details should be added to the report for the assessment of the validity of the anchors.*

**Reply**:

Thank you for your suggestion. We are afraid that at this point, it is not possible for us to do this for every outcome measure considered in this review. All references that were used to derive the MIDs are detailed Supplement S5. For many outcome measures, more than one anchor-based MID was reported. We included studies testing specialist palliative care intervention in cancer, non-cancer, and mixed samples. We tried to use the most appropriate and conservative estimate for the MID for that outcome measure in the specific population we could find. We believe that documenting this level of detail would constitute another review in its own right. We acknowledge this as a limitation in both the abstract and the limitation section (line 358 ff).

*Distribution based MID: what method was used to calculate the distribution based MID? There is detail in the supplementary material regarding the calculation of the SMD but no information appears to be present for method of calculating MID. What SD did the authors use for the calculation of the MID? Could the authors please provide this information.*

**Reply:**

Thank you for comment. As detailed in our reply to the previous comment, we did not calculate the MIDs ourselves. We used the most conservative estimate for the MID reported from studies determining the MID in similar populations to the samples included in the reviewed studies. In Supplement S8, we detail the MID, the methods by which it was determined and we include a reference to the study or review from which the MID used in our analysis for that particular outcome measure was derived.

*Subgroup analysis: While the authors have conducted meta-regression, I would like to ask if they had/have considered using subgroup analyses? I think it would be important to understand the direction of effects in some of the subgroups. For example, cancer/non-cancer/mixed. Did the authors consider and run these analyses? I think it would show some beneficial information and provide further clarification as to whether there are any differences between these subgroups in terms of quality of life and emotional well-being.*

**Reply:**

Thank you for your comment. We had indeed thought about using subgroup analyses, but opted for meta-regression for several reasons. We included a mixture of categorical and metric independent variables, ranging from year of publication of included studies to the service composition score (essentially a weighted integer sum score of elements and professionals present or delivering the SPC service). It would have only been possible to model metric independent variables by splitting these scores into groups, a practice which can distort results when a data-driven method is used. In the absence of a known score for capturing service composition, structure, and delivery method, we felt unable to convert the service score into a categorical variable.

Second, we ran all meta-regressions involving categorical independent variables using dummy coding to a reference category (usually choosing the group with the expected minimum effect as the reference category for ordinal contrasts across groups). Although computationally different and using dummy coding instead of effect coding, meta-regression of categorically scored independent variables will give similar results as a subgroup analysis (under the ALM). However, we refrained from running post-hoc contrasts on these coefficients to control type I error rate and by trusting that the coefficients could be interpreted within the dummy coding approach as differences to the reference category. Meta-regression in this regard was used as a technique allowing the modelling of these two types of independent variables to the outcome SMD(MID).

However, we also appreciate the comment regarding presentation of results and we have tried to revise Table 2 to make it clearer which group or which type of score was associated with a higher effect size.

*.Table 2: I think you should include a measure of heterogeneity (preferably tau) in this table as the results alone could be misleading. I appreciate these are in the supplementary information but I feel there is a need to be clear about the level of statistical heterogeneity in the main text as well.*

**Reply**:

Thank you for your suggestion. We agree to add a measure of heterogeneity to the table. To keep with how information is presented in the main body of text, we have opted for I^2^, presenting the I^2^ values both for the meta-analysis in MID units and the RR meta-analysis.

*Reviewer #3: Dear Authors,
The topic is really interesting, important and useful for the health sector, but to be published, it needs important advice:*

**Reply**:

Thank you for your appreciative review.

*TITLE:
The title needs to be better rewritten, avoiding the repetition of the word "service". The title needs to show the content of the article.*

**Reply**:

Thank you for your comment. We have deleted the second word “service” in the title. We believe that our title covers all the elements that are in our review – we have done a meta-analysis to determine the overall effect of specialist palliative care, and we analyzed how this effect is moderated by service composition, structure, and delivery model with the score used being illustrated in Table 3 of our review.

*SUMMARY:
Separate the items Methods and Results. In Methods, specify the type of study carried out and improve the writing, which is a little confusing.
In Results, we summarize the main results of the study.*

**Reply**:

Thank you for suggesting making the abstract clearer. While it is against the journal’s editorial guidelines to separate the methods and findings, we appreciate that we did not separate these aspects into paragraphs. We have amended the abstract as to your suggestion, separating the methods and findings into paragraphs, and also revised the study design and the results (line 33 ff).

*Summary Conclusions:
It is necessary to respond to the objectives of the study and be in tune with the Conclusions presented at the end of the study.*

**Reply**:

Thank you for suggesting aligning the conclusion with the objectives. We have revised our conclusion section and hopefully now make the main message clearer (line 61 ff).

*INTRODUCTION;
Please review the text to make it clearer, less confusing and specific to the title.
Make clear in the Introduction, the Question (question) and the Importance of the research*

**Reply**:

We have revised the introduction as per your suggestions. First, have added detail to the review of literature, focusing on the gap in the current literature and why it is important to close this gap. We have revised the last paragraph of the introduction and clarified the review’s question (line 143 ff and line 157 ff).

*METHODS:
Explain the Type of Search. Check whether all recommendations on the PRISMA 2020 list have been met.*

**Reply**:

The type of search, databases, access dates, and an abbreviated search strategy are given in Table 1. The full detail with a complete search strategy for our systematic search is provided in Supplement S3. We had checked and uploaded the completed PRISMA checklist but have now included this checklist as an additional appendix (S11) as well.

*In Line 106, Data extraction and vision risk assessment, the authors say that a data deletion spreadsheet was developed and tested. I asked: was it created and tested where, how and by whom? please quote.* *Line 111, Please improve the wording of the sentence related to the risk of bias.
All text regarding Data Analysis and Precise Review Results. It's confusing.*

**Reply**:

Thank you for your suggestion. We have revised the paragraph starting line 106 in the original manuscript, now detailing further how data extraction was accomplished in line 186 ff. We also have added the detail requested who did what. We have also revised line 111 (now line 191).

We are sorry that the text regarding data analysis seemed confusing to the reviewer. We acknowledge that we used novel methodology and that a lot of detail is packed into this section. However, reviewer #2 did not request changes and seemed to be able to follow our description of the analytical methods. We are afraid that to revise this text, we would need more specific guidance as to the aspects deemed confusing.

*CONCLUSION:
In conclusion, it is necessary to respond to the objectives, which are: Estimate the summary effect of specialized palliative care in all environments on quality of life and emotional well-being and identify the ideal model of service provision. The Conclusion that is in the SUMMARY must be, in short, the same that is here at the end.*

**Reply**:

We have extensively revised the discussion and conclusion as per your suggestion. We have aligned the paragraph in the summary with the last paragraph (conclusion) in our manuscript, also answering our objectives.

General journal requests:

*1) Please ensure that the paper adheres to the PLOS Data Availability Policy (see* [*https://eur03.safelinks.protection.outlook.com/?url=http%3A%2F%2Fjournals.plos.org%2Fplosmedicine%2Fs%2Fdata-availability&data=05%7C02%7Cchristina.ramsenthaler%40kcl.ac.uk%7C734b8fd20b5e477572ce08dc5b03cff0%7C8370cf1416f34c16b83c724071654356%7C0%7C0%7C638485319131125851%7CUnknown%7CTWFpbGZsb3d8eyJWIjoiMC4wLjAwMDAiLCJQIjoiV2luMzIiLCJBTiI6Ik1haWwiLCJXVCI6Mn0%3D%7C0%7C%7C%7C&sdata=aQBWlusIwBihC9%2BYZARlzar0TkYlbTkf%2F0I3cMk8A9c%3D&reserved=0)*](http://journals.plos.org/plosmedicine/s/data-availability)*, which requires that all data underlying the study's findings be provided in a repository or as Supporting Information. For data residing with a third party, authors are required to provide instructions with contact information for obtaining the data. PLOS journals do not allow statements supported by "data not shown" or "unpublished results." For such statements, authors must provide supporting data or cite public sources that include it.*

**Reply**:

The data are fully accessible with analysis scripts being documented in full on the Open Science Framework website already detailed in the manuscript.

*2) In addition, we request that you upload any figures associated with your paper as individual TIF or EPS files with 300dpi resolution at resubmission; please read our figure guidelines for more information on our requirements:* [*https://eur03.safelinks.protection.outlook.com/?url=http%3A%2F%2Fjournals.plos.org%2Fplosmedicine%2Fs%2Ffigures&data=05%7C02%7Cchristina.ramsenthaler%40kcl.ac.uk%7C734b8fd20b5e477572ce08dc5b03cff0%7C8370cf1416f34c16b83c724071654356%7C0%7C0%7C638485319131131197%7CUnknown%7CTWFpbGZsb3d8eyJWIjoiMC4wLjAwMDAiLCJQIjoiV2luMzIiLCJBTiI6Ik1haWwiLCJXVCI6Mn0%3D%7C0%7C%7C%7C&sdata=v%2FrF5LbD7nmfbljE%2BZRbs0bJrpalowN21jaxxgmdGk0%3D&reserved=0*](http://journals.plos.org/plosmedicine/s/figures)*. While revising your submission, please upload your figure files to the PACE digital diagnostic tool,* [*https://eur03.safelinks.protection.outlook.com/?url=https%3A%2F%2Fpacev2.apexcovantage.com%2F&data=05%7C02%7Cchristina.ramsenthaler%40kcl.ac.uk%7C734b8fd20b5e477572ce08dc5b03cff0%7C8370cf1416f34c16b83c724071654356%7C0%7C0%7C638485319131136174%7CUnknown%7CTWFpbGZsb3d8eyJWIjoiMC4wLjAwMDAiLCJQIjoiV2luMzIiLCJBTiI6Ik1haWwiLCJXVCI6Mn0%3D%7C0%7C%7C%7C&sdata=ae%2FJIxmbjZaYFcKpuxz3ScwIqkbbbideKzHqmcAHN1Y%3D&reserved=0*](https://pacev2.apexcovantage.com/)*. PACE helps ensure that figures meet PLOS requirements. To use PACE, you must first register as a user. Then, login and navigate to the UPLOAD tab, where you will find detailed instructions on how to use the tool. If you encounter any issues or have any questions when using PACE, please email us at* [*PLOSMedicine@plos.org*](mailto:PLOSMedicine@plos.org)*.*

**Reply**:

We have uploaded the figures using the detailed file type.

*3) We ask every co-author listed on the manuscript to fill in a contributing author statement, making sure to declare all competing interests. If any of the co-authors have not filled in the statement, we will remind them to do so when the paper is revised. If all statements are not completed in a timely fashion this could hold up the re-review process. If new competing interests are declared later in the revision process, this may also hold up the submission. Should there be a problem getting one of your co-authors to fill in a statement we will be in contact.  YOU MUST NOT ADD OR REMOVE AUTHORS UNLESS YOU HAVE ALERTED THE EDITOR HANDLING THE MANUSCRIPT TO THE CHANGE AND THEY SPECIFICALLY HAVE AGREED TO IT. You can see our competing interests policy here:* [*https://eur03.safelinks.protection.outlook.com/?url=http%3A%2F%2Fjournals.plos.org%2Fplosmedicine%2Fs%2Fcompeting-interests&data=05%7C02%7Cchristina.ramsenthaler%40kcl.ac.uk%7C734b8fd20b5e477572ce08dc5b03cff0%7C8370cf1416f34c16b83c724071654356%7C0%7C0%7C638485319131141549%7CUnknown%7CTWFpbGZsb3d8eyJWIjoiMC4wLjAwMDAiLCJQIjoiV2luMzIiLCJBTiI6Ik1haWwiLCJXVCI6Mn0%3D%7C0%7C%7C%7C&sdata=QfghujdplFSZebyePZojSdqfLUEZPOW3NS%2BiUWUuxxY%3D&reserved=0*](http://journals.plos.org/plosmedicine/s/competing-interests)*.*

**Reply**:

All authors have completed this information.
