# Supplementary material for: Benefits of specialist palliative care by identifying active ingredients of service composition, structure, and delivery model: A systematic review with meta-analysis and meta-regression
Source: PLoS Med. 2024 Aug 2;21(8):e1004436. doi: 10.1371/journal.pmed.1004436 (PMC11329153; doi:10.1371/journal.pmed.1004436)
Supplement: Attachment — Submitted filename: Replyletter_SPCMetaanalysis_PLOSMed_R3.docx [file pmed.1004436.s013.docx]

Editorial team and academic editor

Dr Alexandra Torsun, PhD

PLOS Medicine

June 6^th^, 2024

Dear Dr Tosun,

Dear Editors,

Re: **PMEDICINE-D-24-00814R2 “Does specialist palliative care provide additional benefits? A meta-analysis with meta-regression to identify active ingredients of service composition, structure, and delivery model”**

Many thanks for your time in reviewing the above manuscript, and for your comments which have helped us to hopefully improve the manuscript. We enclose the revised manuscript for your consideration, and outline below how we have addressed each point.

**Requests from Editors:**

*Please update the Financial Disclosure Statement on the online submission form with the information provided on lines 408-413.*

**Reply**: We have done so, updating the online submission form.

*ABSTRACT*

*1) l.33: Please change to: “We conducted a..”.*

**Reply**: Thank you for this suggestion. We have copied as suggested in line 33.

*2) l.39: Please temper claims of primacy of results by stating, "to our knowledge" or something similar (i.e. “The meta-analysis used, to our knowledge, novel methodology to convert outcomes into minimally clinically…”).*

**Reply**: Thank you for this suggestion. We have tempered the claims by adding “to our knowledge” in line 39.

*3) ll.45-46: “almost exclusively from high and middle-income countries” – Thank you for including this detail. We suggest including an exact number.*

**Reply**: Thank you. We have included the exact number “(n = 38 from high and middle-income countries)” in line 45.

*4) l.47: Please delete ‘with’ before the brackets.*

**Reply:** Thank you for noticing this superfluous word. We have deleted this in line 47.

*5) l.48: Please define “CI” at first use.*

**Reply**: Thank you. We have defined this in line 48.

*6) l.48: “effect size of 0.40 at 4 to 6 months”- in the table the time span is “at 13 to 36 weeks”, please revise and make sure this is consistently reported throughout.*

**Reply**: Thank you for alerting us to this change in presenting time frames/time points of our data. We have made changes throughout the manuscript in lines 48, 49, 52, 284, 307.

*7) ll.49-50: For clarity, please re-iterate the time frame this refers to.*

**Reply**: Thank you. We have re-iterated the time frame at ‘13 to 36 weeks’ in line 49.

*8) l.50: Please define “RR” at first use in the Abstract.*

**Reply**: Thank you. We have defined RR in line 50.

*9) l.50/l.51: In line 50, you mention "1 MID magnitude change" and provide a risk ratio, whereas in the following line you describe the results as "1 MID unit change" and the corresponding risk ratio is missing (RR 1.16; l.298). Please revise.*

**Reply**: Thank you for noticing this change in wording. We have changed “magnitude” to “unit” throughout, in line 50 and line 260.

*We have also noted that in the abstract you refer to the time frame as «4 to 6 months», whereas in the main text and Table 2 this time frame is referred to as «13 to 36 weeks» (which is not the same as 4 to 6 months) – please revise and use a consistent format (see comments above).*

**Reply**: Thank you for noticing this mixture in the presentation of time points. We have changed this throughout the manuscript to make it consistent (please see point (6)).

*10) l.56: “lower number-needed-to-treat (14)” – We feel the number 14 here is rather confusing without appropriate context. We suggest removing it.*

**Reply**: We agree with your assessment. We have removed this number from the statement.

*11) l.61: Please temper claims of primacy of results by stating, "to our knowledge" or something similar.*

**Reply**: We have done so in line 61.

*AUTHOR SUMMARY Thank you for providing the Author Summary. The Author Summary in its current format is rather long and needs to be shortened. It should contain only the most important details for each of the questions, in a condensed form. For example, the bullet points of “Why was this study done?”, could be condensed as follows (please note that the last bullet point belongs under the second question “What did the researchers do and find?”):*

*-	Specialist palliative care (SPC) services provide a complex intervention that addresses the holistic needs of individuals with life-limiting conditions and their families.*

*-	Different intervention models include a variation of different disciplines (doctors, nurses, psychologists, etc.), configurations (e.g., whether out-of-hours care is provided), and settings (hospital, hospice, community, etc.)*

*-* *The overall effectiveness of SPC on quality of life and emotional wellbeing is undetermined due to large variation in intervention models and heterogeneity in outcome measures used to measure quality of life and emotional wellbeing.*

*-	We systematically reviewed evidence for the effectiveness of SPC, to assess which intervention model components and configurations are most effective in improving quality of life or emotional wellbeing.*

*It may be helpful to review currently published articles for examples which can be found on our website here* [*https://eur03.safelinks.protection.outlook.com/?url=https%3A%2F%2Fjournals.plos.org%2Fplosmedicine%2F&data=05%7C02%7Cchristina.ramsenthaler%40kcl.ac.uk%7C8ad6c93f288c46a9dca708dc8186c65d%7C8370cf1416f34c16b83c724071654356%7C0%7C0%7C638527663050114353%7CUnknown%7CTWFpbGZsb3d8eyJWIjoiMC4wLjAwMDAiLCJQIjoiV2luMzIiLCJBTiI6Ik1haWwiLCJXVCI6Mn0%3D%7C0%7C%7C%7C&sdata=8ZRIBSpZVuUV9851XfH4Ru%2FWd%2B4jUAx9vszZ1PXGD7M%3D&reserved=0*](https://eur03.safelinks.protection.outlook.com/?url=https%3A%2F%2Fjournals.plos.org%2Fplosmedicine%2F&data=05%7C02%7Cchristina.ramsenthaler%40kcl.ac.uk%7C8ad6c93f288c46a9dca708dc8186c65d%7C8370cf1416f34c16b83c724071654356%7C0%7C0%7C638527663050114353%7CUnknown%7CTWFpbGZsb3d8eyJWIjoiMC4wLjAwMDAiLCJQIjoiV2luMzIiLCJBTiI6Ik1haWwiLCJXVCI6Mn0%3D%7C0%7C%7C%7C&sdata=8ZRIBSpZVuUV9851XfH4Ru%2FWd%2B4jUAx9vszZ1PXGD7M%3D&reserved=0)

**Reply**: We absolutely agree with your assessment of the original’s author summary being too long. We greatly appreciate you taking the time to rephrase the first section for us. We have copied this into the revision. We have also shortened all subsequent paragraphs and revised the ordering of points. Thank you for your help in shortening this summary. This is greatly appreciated.

*INTRODUCTION*

*l.160: Please temper claims of primacy of results by stating, "to our knowledge" or something similar.*

**Reply**: Thank you for your suggestion. We have added this to line 141 (the line numbers are lower due to shortening the author summary).

METHODS AND RESULTS

*1) In the Methods section, we suggest adding a brief explanation on the calculation of the number needed to treat (with reference to the Supplementary Appendix), together with the interpretation of the NNT.*

**Reply**: Thank you for your suggestion. We absolutely agree. We have added a brief explanation, referred to the appendix and provided an interpretation of the NNT in line 205ff.

*2) ll.172/173: We think it might be helpful to explain the data restriction choice in more detail, as you did in response to the reviewer comments. Please be sure to include a reference. For example: "Given that SPC service models have become part of mainstream healthcare only in recent years, and that for most countries in Europe or the United States, service models and their components have been developed since the 2000s, we restricted the search to synthesizing effects for contemporary service models.”*

**Reply**: Thank you for this suggestion. We have done so, also adding a reference in line 153ff.

*3) l.249: “RCTs by Liu et al [28] and Nottelmann et al [39] included the most diverse group.” – in terms of different professions involved? Please clarify.*

**Reply**: We agree. We included “of professions” in line 236.

*4) l.257: Please change to “populations with cancer”. We prefer the use of patient-centered language. Please revise accordingly throughout the main text.*

**Reply**: We agree with the use of patient-centered language. We have changed line 244 accordingly, also changing line 245, line 246 line 297.

*5) l.258: “Of these, 19 RCTs used mixed cancer populations.” – Please clarify what mixed cancer populations means (different types of cancer?).*

**Reply**: Thank you for noticing this potentially confusing statement. We have changed this to “19 RCTs used populations with different cancer diagnoses” in line 245.

*6) ll.259-261: “The mean age was 65.8 years…” – if possible, please provide standard deviation and interquartile ranges when presenting mean and median values.*

**Reply**: Unfortunately, as this mean age was calculated as a weighted average from the original included studies mean or median age, it is not possible to calculate a measure of spread from this aggregated data. While we could potentially calculate the weighted standard deviation from individual trial’s SD (if reported) and sample sizes provided for age, the resulting measure of spread would not be valid. Also, measures of spread are not reported in all individual studies. We appreciate the request for reporting measures of spread with any measures of location (as a professor of statistics I agree with this statement wholeheartedly!). However, I am afraid it is neither straightforward nor unbiased (or even possible) to do so in this instance.

We are sorry that we probably cannot follow this suggestion.

*7) l.271: Please define “UC” at first use.*

**Reply**: We have done so in line 258.

*8) l.275: Please define “NNT” at first use.*

**Reply**: We have done so in line 264.

*9) l.283: “The covariate consistently associated with the MD(MID) was the attrition..” – should this be “SMD”?*

**Reply**: Thank you so much for noticing this inconsistency (you really have an eagle’s eyes)! We have changed this to SMD(MID) in line 271.

*10) l.289: Please check whether you meant “SMD” instead of “MD”.*

**Reply**: Thank you once again. Indeed, we meant SMD. We have changed this in line 277.

*11) l.290: Please change to “(p=0.003)”.*

**Reply**: Thank you for noticing this. Something went wrong with the Search and Replace we ran when preparing the last submission. We have added as suggested in line 278.

*FIGURES AND TABLES*

*1) Figure 1: Please define “RCT” in the figure description or write in full in the figure.*

**Reply**: We have added “RCT” to the list of abbreviation in the figure’s description.

*2) Table 1: Please define “SPC” and “QoL” at first use.*

**Reply**: We have defined both abbreviations at first use in Table 1.

*3) Table 3: Please either define “SPC” in the title and “ACP” in the table or list both definition below the table.*

**Reply**: We have defined “ACP” as “Advance care planning” in both table headers. Of note, I only included two table headers to make this table more readable. Please remove the second table header when setting/layouting, it was just for ease of reading this table broken across two pages.

SPC is now also defined in the table’s title.

*DISCUSSION*

*1) Please remove any subheadings from the Discussion.*

**Reply**: We removed all subheadings from the discussion and put leading subphrases at the beginning of the paragraph starting the new subsection of the discussion to help the reader and structure the text.

*2) ll.316-319: “The NNT for one patient to have benefit of at least 1 MID improves markedly for both types of measures in studies with follow-up of between 3 and 6 months; QoL outcomes improve from 74 (<3 months) to 16 (4 to 6+ months), and emotional outcomes improve from 49 (<3 months) to 24 (4 to 6 months).” – in the second part of the statement, please reiterate that the numbers you are describing are the numbers needed to treat. In addition, we have noticed that the numbers presented here do not match the numbers presented in the abstract (ll.52-52). We are not sure where the numbers come from, please check and comment.*

**Reply**: Again, thank you for noticing this inconsistency. We have checked and included all the numbers reported in the tables and also re-iterated that this was the NNT we referred to throughout this paragraph (line 306 ff.)

*3) l.350: Please temper claims of primacy of results by stating, "to our knowledge" (or something similar), or removing the word “novel”.*

**Reply**: If possible, we would like to keep the word ‘novel’ in this section. We believe it really is ‘novel’ in the sense of (a) using a novel technique to meta-analyse findings from trials, and (b) providing for the first time a comprehensive and robust meta-analysis of all trials reporting on quality-of-life outcomes and emotional wellbeing outcomes in specialist palliative care. All prior meta-analyses (of which there have been few) were not able to combine all evidence into one summary measure. Therefore, despite the method of converting to MID units being used in other disciplines, it’s the first time this technique is used for meta-analysis in palliative care and it is the first time the technique is used, so a summary effect over all trials identified through the systematic review can be given. However, we do agree that in the context of this sentence, the suggestion to temper the claim is absolutely correct and we have done so (line 338/339).

*4) ll.350-352, please change to: “As a distinct strength of our work, our methodology, combining MIDs across different outcomes, allowed us to include more RCTs than previous meta-analyses and enabled robust conclusions regarding clinical importance of our findings.”*

**Reply**: See statement above. We would like to suggest using the tempering statement «As a distinct strength of our work, the - to our knowledge - novel methodology,…». Our explanation is provided in the answer above. We would also be happy to rephrase in a different way, but very much would like to keep the word «novel» in one form or other, if possible.

SUPPLEMENTARY MATERIAL

Thank you for providing the completed PRISMA checklist. We ask you to revise the checklist using section and paragraph numbers, when completing the checklist (i.e. please specify “Please see paragraph in manuscript”).

**Reply**: We have added all paragraphs and sections to the PRISMA checklist in the supplemental appendix.

*SOCIAL MEDIA*

*To help us extend the reach of your research, please provide any X (formerly known as Twitter) handle(s) that would be appropriate to tag, including your own, your co-authors’, your institution, funder, or lab. Please enter in the submission form any handles you wish to be included when we post about this paper.*

**Reply**: Thank you for suggesting this. We have already entered all X handles on the submission form.

**Comments from Reviewers:**

*Reviewer #2: The authors have provided detailed and well written responses. Where they could accommodate the reviewers they have, when they could not, they provide well justified arguments and clear reasoning. From a statistical standpoint they have conducted the meta-analysis using the best available data. Again, I would just like to show my appreciation to the work that has gone into this review.*

**Reply**: Thank you so much for your positive statement. We also would like to thank you for your time in reviewing the manuscript.

*Reviewer #3: The topic developed is interesting and useful for health professionals. In the previous review, I sent important comments and suggestions to the authors of the article. In the current review, I verify that the suggestions were considered and that the article was clear and easy for the reader to understand. The title is better and represents the content covered in the article. The details regarding the methods allow reproduction by other researchers. The conclusions respond to the objective of the article.*

**Reply**: Thank you so much for your positive statement. We also would like to thank you for your time in reviewing the manuscript.

**General Editorial Requests**

*1) We ask every co-author listed on the manuscript to fill in a contributing author statement. If any of the co-authors have not filled in the statement, we will remind them to do so when the paper is revised. If all statements are not completed in a timely fashion this could hold up the re-review process. Should there be a problem getting one of your co-authors to fill in a statement we will be in contact. YOU MUST NOT ADD OR REMOVE AUTHORS UNLESS YOU HAVE ALERTED THE EDITOR HANDLING THE MANUSCRIPT TO THE CHANGE AND THEY SPECIFICALLY HAVE AGREED TO IT.*

**Reply**: All authors have completed this information.

*2) Please ensure that the paper adheres to the PLOS Data Availability Policy (see https://eur03.safelinks.protection.outlook.com/?url=http%3A%2F%2Fjournals.plos.org%2Fplosmedicine%2Fs%2Fdata-availability&data=05%7C02%7Cchristina.ramsenthaler%40kcl.ac.uk%7C8ad6c93f288c46a9dca708dc8186c65d%7C8370cf1416f34c16b83c724071654356%7C0%7C0%7C638527663050121093%7CUnknown%7CTWFpbGZsb3d8eyJWIjoiMC4wLjAwMDAiLCJQIjoiV2luMzIiLCJBTiI6Ik1haWwiLCJXVCI6Mn0%3D%7C0%7C%7C%7C&sdata=yeTAEp8Nx%2BWA9vHUnvkTMHGJHzzIxLliccP%2FKryLljA%3D&reserved=0), which requires that all data underlying the study's findings be provided in a repository or as Supporting Information. For data residing with a third party, authors are required to provide instructions with contact information for obtaining the data. PLOS journals do not allow statements supported by "data not shown" or "unpublished results." For such statements, authors must provide supporting data or cite public sources that include it.*

**Reply**: We have done this using the following statement: “All data included were derived from publicly available documents cited in the references. All data, a data dictionary and full analysis scripts for R can be obtained on the Open Science Framework website for this project (<https://osf.io/h8pmz/>).”

3) Please review your reference list to ensure that it is complete and correct. If you have cited papers that have been retracted, please include the rationale for doing so in the manuscript text, or remove these references and replace them with relevant current references. Any changes to the reference list should be mentioned in the rebuttal letter that accompanies your revised manuscript.

**Reply**: We have reviewed the reference list and no cited papers have been retracted. We have made one relevant change to the list by adding reference [77] (please see comment on arguing the search limitation (time period) above). No further changes were made.

Thank you for the time and the incredible care taken when providing feedback to our manuscript. We greatly appreciate your feedback and suggestions.
