# Supplementary material for: Benefits of specialist palliative care by identifying active ingredients of service composition, structure, and delivery model: A systematic review with meta-analysis and meta-regression
Source: PLoS Med. 2024 Aug 2;21(8):e1004436. doi: 10.1371/journal.pmed.1004436 (PMC11329153; doi:10.1371/journal.pmed.1004436)
Supplement: Attachment — Submitted filename: Replyletter_SPCMetaanalysis_PLOSMed_R4.docx [file pmed.1004436.s014.docx]

Editorial team and academic editor

Alexandra Torsun PhD

PLOS Medicine

June 28^th^, 2024

Dear Dr Tosun,

Dear Editors,

Re: **PMEDICINE-D-24-00814R3 “Does specialist palliative care provide additional benefits? A meta-analysis with meta-regression to identify active ingredients of service composition, structure, and delivery model”**

We would like to thank you again for the time and precision with which you have reviewed our manuscript. Your comments have hopefully helped us to improve the manuscript further. We enclose the revised manuscript for your consideration, and outline below how we have addressed each point.

**Requests from Editors:**

*1) Please revise your title according to PLOS Medicine's style. Your title must be nondeclarative and not a question. It should begin with the main concept if possible. "Effect of" should be used only if causality can be inferred, i.e., for an RCT. Please place the study design ("A randomized controlled trial," "A retrospective study," "A modelling study," etc.) in the subtitle (i.e., after a colon). For example: Benefits of specialist palliative care by identifying active ingredients of service composition, structure, and delivery model: A systematic review with meta-analysis and meta-regression*

**Reply**: We have revised the title as per your suggestion. The title now is: “Benefits of specialist palliative care by identifying active ingredients of service composition, structure, and delivery model: A systematic review with meta-analysis and meta-regression”.

*2) Abstract: As you are reporting results for NNT in the Abstract, please mention this in the Methods section of the Abstract. For example: "We also calculated the number needed to treat (NNT)."*

**Reply**: Thank you for this suggestion. We have added this information to the abstract (line 40). This change necessitated shortening the abstract to keep it within the word count. We have shortened the information on additional searches starting in line 35 f.

*3) l.47: “…benefit was seen from four months’ follow-up..” – should this say three months, referring to the 13 (to 36) weeks? Please check and revise.*

**Reply**: Thank you for noticing this inconsistency. We have changed as suggested in this line.

*4) ll.61-64, please change to: “Using, to our knowledge, novel methods to combine different outcomes, we show clear evidence of moderate overall effect size for both quality-of-life and emotional wellbeing benefits from SPC, regardless of underlying condition, with multi-disciplinary, multi-component, and multi-setting models being most effective.”*

**Reply**: Thank you for your suggestion. We have copied this exact wording into the abstract in the indicated lines.

*5) l.86: Please remove the word “all”.*

**Reply:** Thank you for noticing this superfluous word. We have removed this as per your suggestion.

*6) l.105: Please create a new bullet point starting with “Honoring the complex and…”.*

**Reply**: Thank you. We have created a new bullet point at the indicated position.

*7) l.284: “at our primary endpoint of 13 to 38 weeks” – please change to ’36 weeks’.*

**Reply**: Again, thank you for noticing this inconsistency. We have revised as per your request.

*8) l.302ff: Please revise the Discussion for tense. Results and findings should be discussed in the past tense, e.g. “For QoL and emotional outcomes, statistically and clinically significant benefits were seen in trials with follow up for three months or more.”.*

**Reply**: Thank you for checking the manuscript for inconsistency in tense. We have revised the tense in both the results and the discussion sections, starting from line 285 onwards.

*9) ll.304-307, please change to: “The NNT for one patient to have benefit of at least 1 MID improved for both types of measures in studies with follow-up of between 3 and 6 months. The NNT for QoL outcomes improved from 69 (<3 months) to 15 (12 weeks) and 20 (13+ weeks), and the NNT for emotional outcomes improved from 46 (<3 months) to 19 (13 to 36 weeks).”*

**Reply**: Thank you. We have changed these lines as requested.

*10) Figure 1: You have included the definition of 'RCT' in the list of abbreviations for Figure 2 instead of Figure 1. Please revise (you may write 'RCT' in full in the flow diagram).*

**Reply**: Thank you for noticing this omission. We have written the word RCT in full and enclose a new, revised Figure 1.

Please note that we have changed a few smaller mistakes in the discussion section which can be seen in the marked up copy of the submission (for example, changed the number in line 320 which still had a mid-point dot instead of a decimal dot, or changed the wording in line 347 & 348 to enhance the readability of this sentence).

**Comments from Reviewers:**

There were no comments from reviewers to address.

**General Editorial Requests**

*1) We ask every co-author listed on the manuscript to fill in a contributing author statement. If any of the co-authors have not filled in the statement, we will remind them to do so when the paper is revised. If all statements are not completed in a timely fashion this could hold up the re-review process. Should there be a problem getting one of your co-authors to fill in a statement we will be in contact. YOU MUST NOT ADD OR REMOVE AUTHORS UNLESS YOU HAVE ALERTED THE EDITOR HANDLING THE MANUSCRIPT TO THE CHANGE AND THEY SPECIFICALLY HAVE AGREED TO IT.*

*2) Please ensure that the paper adheres to the PLOS Data Availability Policy (see https://eur03.safelinks.protection.outlook.com/?url=http%3A%2F%2Fjournals.plos.org%2Fplosmedicine%2Fs%2Fdata-availability&data=05%7C02%7Cchristina.ramsenthaler%40kcl.ac.uk%7Cf7b21d4da1aa4188ac2108dc91f510a4%7C8370cf1416f34c16b83c724071654356%7C0%7C0%7C638545728927328490%7CUnknown%7CTWFpbGZsb3d8eyJWIjoiMC4wLjAwMDAiLCJQIjoiV2luMzIiLCJBTiI6Ik1haWwiLCJXVCI6Mn0%3D%7C0%7C%7C%7C&sdata=zPzScPOmcxssaxqjCYA6fMtfTJTfINeNMv6rGsHfBAo%3D&reserved=0), which requires that all data underlying the study's findings be provided in a repository or as Supporting Information. For data residing with a third party, authors are required to provide instructions with contact information for obtaining the data. PLOS journals do not allow statements supported by "data not shown" or "unpublished results." For such statements, authors must provide supporting data or cite public sources that include it.*

*3) Please review your reference list to ensure that it is complete and correct. If you have cited papers that have been retracted, please include the rationale for doing so in the manuscript text, or remove these references and replace them with relevant current references. Any changes to the reference list should be mentioned in the rebuttal letter that accompanies your revised manuscript.*

**Reply**: These three standing request have already been addressed in our last revision, R3.

Once again, thank you for the time and the incredible care taken when providing feedback to our manuscript. We greatly appreciate your feedback and suggestions.
